# Short Sb···Sb Contacts in Polymorphic Tris(*p‐*Tolyl)Antimony: A Hallmark of Organoantimony(III) Solid‐State Chemistry?

**DOI:** 10.1002/anie.5894899

**Published:** 2026-08-12

**Authors:** Imogen Suh, Michael Ferguson, Filip Topić, Fu‐Qiang Gong, Mihails Arhangelskis, Hatem M. Titi, D. Scott Bohle, Vinko Nemec, Levente Kiss, Andrew J. Morris, Cristian Silvestru, Dominik Cinčić, Tomislav Friščić

**Affiliations:** ^1^ Department of Chemistry McGill University Montreal Canada; ^2^ School of Chemistry University of Birmingham Edgbaston UK; ^3^ Faculty of Chemistry University of Warsaw Warsaw Poland; ^4^ Department of Chemistry Faculty of Science University of Zagreb Zagreb Croatia; ^5^ Department of Chemistry Supramolecular Organic and Organometallic Chemistry Centre (SOOMCC) Faculty of Chemistry and Chemical Engineering Babeș‐Bolyai University Cluj‐Napoca Romania; ^6^ School of Metallurgy and Materials University of Birmingham Edgbaston UK

**Keywords:** antimony, crystal engineering, polymorphism, supramolecular, thermal analysis

## Abstract

Discovery of a polymorph of tris(*p‐*tolyl)antimony(III) reveals solid‐state supramolecular dimers connected through short, attractive Sb∙∙∙Sb contacts that, based on theoretical calculations and a systematic screen of the Cambridge Structural Database (CSD), could be an overlooked hallmark of the solid‐state and supramolecular chemistry of trivalent organoantimony(III) compounds. While the previously reported crystal structure of tris(*p‐*tolyl)antimony(III) is known to exhibit phenyl embrace motifs, resembling the arsenic or phosphorus analogs, the new polymorph exhibits supramolecular dimers characterized by direct Sb∙∙∙Sb contacts shorter than the sum of the van der Waals radii, and stabilized by a significant dimer dissociation energy of ca. −26 kJ mol^−1^ established by periodic density‐functional calculations. Theoretical analysis indicates that the contacts are attractive and dispersion‐dominated, with an overview of the CSD showing that such direct Sb···Sb contacts are not unique to this polymorphic form of tris(*p‐*tolyl)antimony(III) but appear to be a more general feature of trivalent organoantimony in the solid state. Specifically, the CSD shows that dimers based on short Sb···Sb contacts are found across a number of organoantimony structures, including some of the simplest representatives of the series, such as triphenyl‐ and trimethylantimony, for which the dimer interaction energy is herein calculated to be ca. −13 kJ mol^−1^.

## Graphical abstract

Discovery of polymorphism in solid tris(*p‐*tolyl)antimony(III) highlighted short, direct antimony‐antimony contacts, with interatomic separation shorter than the sum of the van der Waals radii, and calculated interaction energy of ca. 26 kJ mol^−1^ that includes a significant dispersion component. Analysis of the Cambridge Structural Database indicates that such contacts are found across a wide range of structures and present a general, but so far poorly explored hallmark of supramolecular chemistry of organoantimony in the solid state.

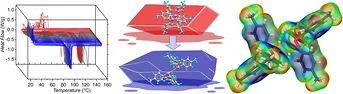

**Abbreviations**
CH_3_CN: acetonitrile
CSD: Cambridge Structural Database
DFT: density‐functional theory
DSC: differential scanning calorimetry
Et_2_O: diethyl ether
MAS: magic angle spinning
MBD: many‐body dispersion
NMR: nuclear magnetic resonance
*p‐*Tol: para‐tolyl‐
PXRD: powder x‐ray diffraction
Sb(*p‐*Tol)_3_
: tris(*para*‐tolyl)antimony(III)
SbCl_3_
: antimony trichloride
SSNMR: solid‐state nuclear magnetic resonance
VT‐PXRD: variable‐temperature powder x‐ray diffraction.
Δ*H*_m_
: enthalpy of melting

## 1 Introduction

Supramolecular chemistry of antimony has recently attracted significant attention in terms of fundamental studies, catalysis, and materials design, focusing on the role of antimony as a donor or acceptor in σ‐hole interactions, such as pnictogen and halogen bonding, respectively [1, 2, 3, 4, 5, 6, 7, 8, 9, 10, 11, 12, 13, 14, 15, 16, 17, 18]. Here, we describe short Sb···Sb contacts appearing in solid‐state structures of organoantimony(III) compounds, which came to our attention upon the discovery of polymorphism [19, 20, 21, 22, 23] in a long‐known [24, 25, 26, 27] organometallic compound, tris(*para*‐tolyl)antimony(III) (Sb(*p‐*Tol)_3_ [28] Figure 1a). Whereas the originally reported structure [28] of Sb(*p‐*Tol)_3_ (Form I) consists of supramolecular dimers based on the phenyl embrace motif (Figure 1a) [29], isostructural to P(*p‐*Tol)_3_ and As(*p‐*Tol)_3_ homologs [30, 31, 32, 33, 34], we now report the structure of a metastable polymorph (Form II) characterized by molecular dimers exhibiting short Sb···Sb contacts of just below 4 Å (Figure 1b), stabilized by a calculated interaction energy of ca. −26 kJ mol^−1^. Notably, a targeted CSD search reveals that such motifs occur in a small, but significant number of already reported structures of trivalent organoantimony compounds. Whereas short antimony‐antimony contacts, reported by Ashe in several thermochromic distibines and comprising antimony in the +2 oxidation state, attracted attention in the 1980s as a potential means to explain and even design thermochromism in such systems [35, 36, 37, 38, 39, 40, 41, 42], the present work presents a broader perspective and shows that such short, direct Sb···Sb contacts can be stabilizing, and appear in a wider range of organoantimony structures, potentially making them a general hallmark of supramolecular chemistry of organometallic antimony in the solid state.

**FIGURE 1:**
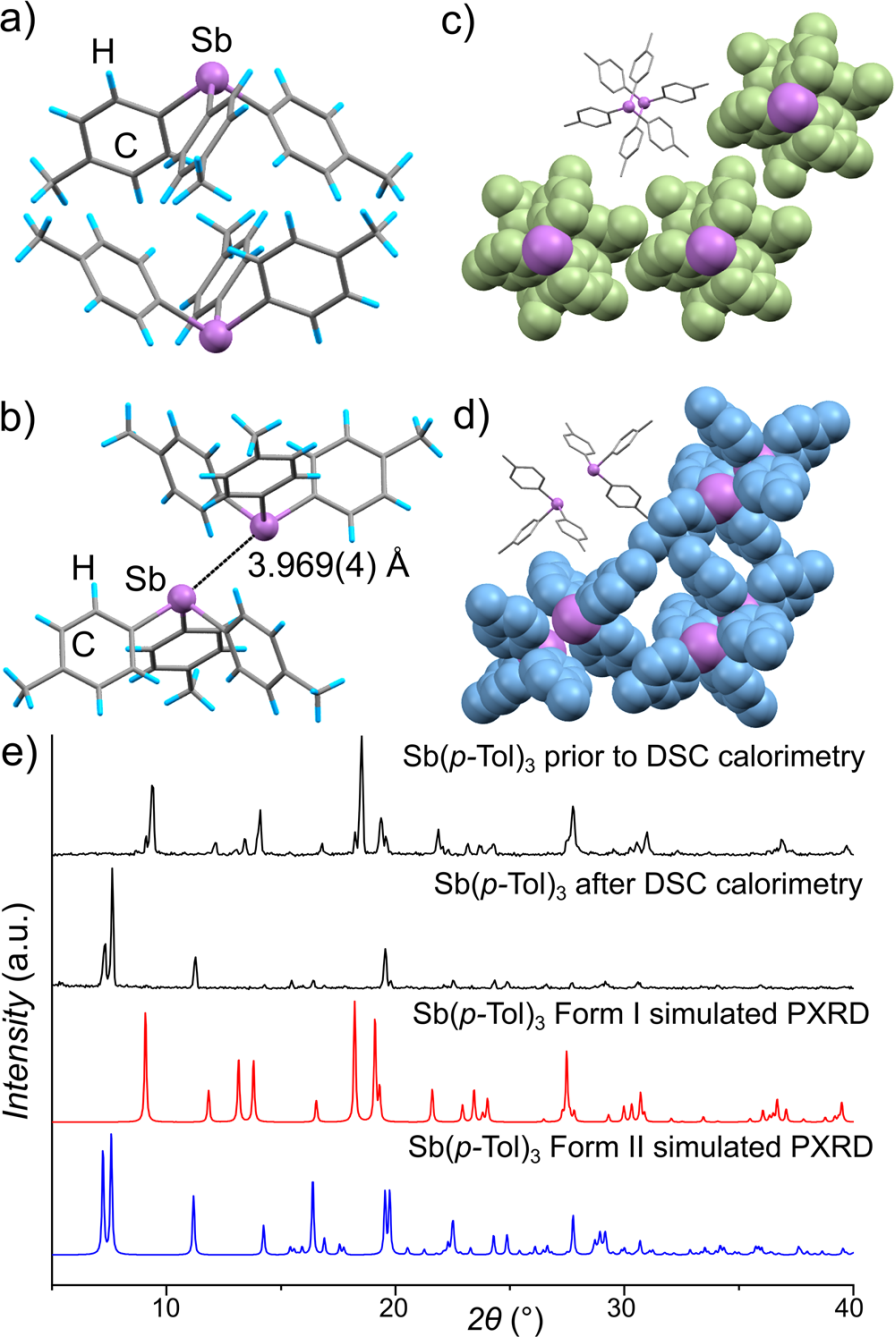
Fragments of the crystal structures of Sb(*p‐*Tol)_3_: (a) Form I showing a phenyl embrace motif. (b) Form II showing Sb···Sb short contacts (highlighted by a line); (c) rows of phenyl embrace dimers in the [002] plane of Form I, and (d) Sb···Sb dimers in the [020] plane of Form II. In panels (c) and (d), the hydrogen atoms are not shown for clarity. (e) Comparison of PXRD patterns for (top‐to‐bottom): Sb(*p‐*Tol)_3_ prior to DSC experiment; after a sequence of being cooled to −20°C and warmed to 150°C repeated three times; simulated for Sb(*p‐*Tol)_3_ Form I and simulated for Sb(*p‐*Tol)_3_ Form II.

## 2 Results and Discussion

Title compound was obtained by reaction of SbCl_3_ with *p*‐TolMgBr [43], purified by liquid‐liquid extraction using diethyl ether (Et_2_O) and water, followed by drying over anhydrous Na_2_SO_4_ and recrystallization from 2‐propanol (*i‐*PrOH). Crystallization from *i‐*PrOH yielded crystals matching the previously reported Form I, as indicated by powder X‐ray diffraction (PXRD). An unsuccessful attempt to obtain a halogen‐bonded cocrystal [14, 44] of Sb(*p‐*Tol)_3_ with 1,4‐diiodooctafluorobutane led, however, to diffraction‐quality crystals of a new, monoclinic polymorph of Sb(*p‐*Tol)_3_ (Form II, Figure 1b, Table 1). X‐ray crystal structure analysis showed that molecules in Form II are not associated via phenyl embrace motif but form short Sb···Sb contacts of 3.969(4) Å and stack in a head‐to‐tail fashion (see Supporting Information for further details of supramolecular interaction analysis). This separation is notably shorter than the sum of van der Waals radii according to Mantina et al. (4.12 Å, ca. 4% shorter), Batsanov (4.4 Å, ca. 10% shorter), Alvarez (4.94 Å, ca. 20% shorter) and, most recently, Charry and Tkatchenko (4.62 Å, ca. 14% shorter) [45, 46, 47, 48]. For comparison, we have also re‐determined the structure of Form I (Table 1), which was fully consistent with the original report. The differences in molecular assembly between the two forms are also reflected in Hirshfeld surfaces (see Supporting Information) [49, 50]. The structures of Form I and II also exhibit ring‐like dimers of C‐H···π bonds (see Supporting Information) and consist of staggered rows of supramolecular dimers based on either phenyl embrace (Form I) or Sb···Sb contacts (Form II) (Figure 1c,d), respectively.

**TABLE 1:** Selected crystallographic data for Sb(*p‐*Tol)_3_ Forms I and II.

Parameter	Form I^a^	Form II
Space group	*R* $\bar{3}$	*P*2_1_/*c*
*a* (Å)	12.8233(2)	11.7532(4)
*b* (Å)	12.8233(2)	6.4245(2)
*c* (Å)	20.1811(4)	24.6837(9)
*β* (°)	90	97.896(2)
*V* (Å^3^)	2873.92	1846.16
Voids volume (%)^b^	3.3	2.8

*Note*: Full crystallographic and general data are given in the Supporting Information.

^a^ Re‐determined structure of Sb(*p‐*Tol)_3_ Form I.

^b^ Fraction (%) of the unit cell volume occupied by voids accessible to a spherical probe 1 Å in diameter, calculated using MercuryCSD [51].

Subsequent attempts to deliberately synthesize Form II by milling in the presence of additives, crystal seeds, or solvents, as well as by solution crystal growth with and without the presence of the halogen bond donor, or pre‐made seeds of Form II, were not successful [52]. Instead, the next observation of Form II occurred upon differential scanning calorimetry (DSC) analysis of a sample of initially Form I. After three cycles of heating and cooling in a hermetically sealed aluminum pan, in the range from −20 to 150°C, at a rate of 5°C min^−1^, and with each heating or cooling segment separated by a one‐minute isothermal hold, PXRD analysis of a sample of Sb(*p*‐Tol)_3_ revealed the presence of Form II (Figure 1e). In an attempt to obtain Form II in larger amounts, freshly prepared samples of Form I were placed in one‐dram vials which were then submerged into an oil bath set at 140°C until the material was seen to be fully liquified (melting point of Form I was found to be 127‐129°C, see Supporting Information). The vials were removed from the oil bath, and the samples were allowed to resolidify upon spontaneous cooling to room temperature. The resulting solid material was ground using a mortar and pestle, with subsequent PXRD analysis indicating that all six samples still consisted of Form I (Figure 2a).

**FIGURE 2:**
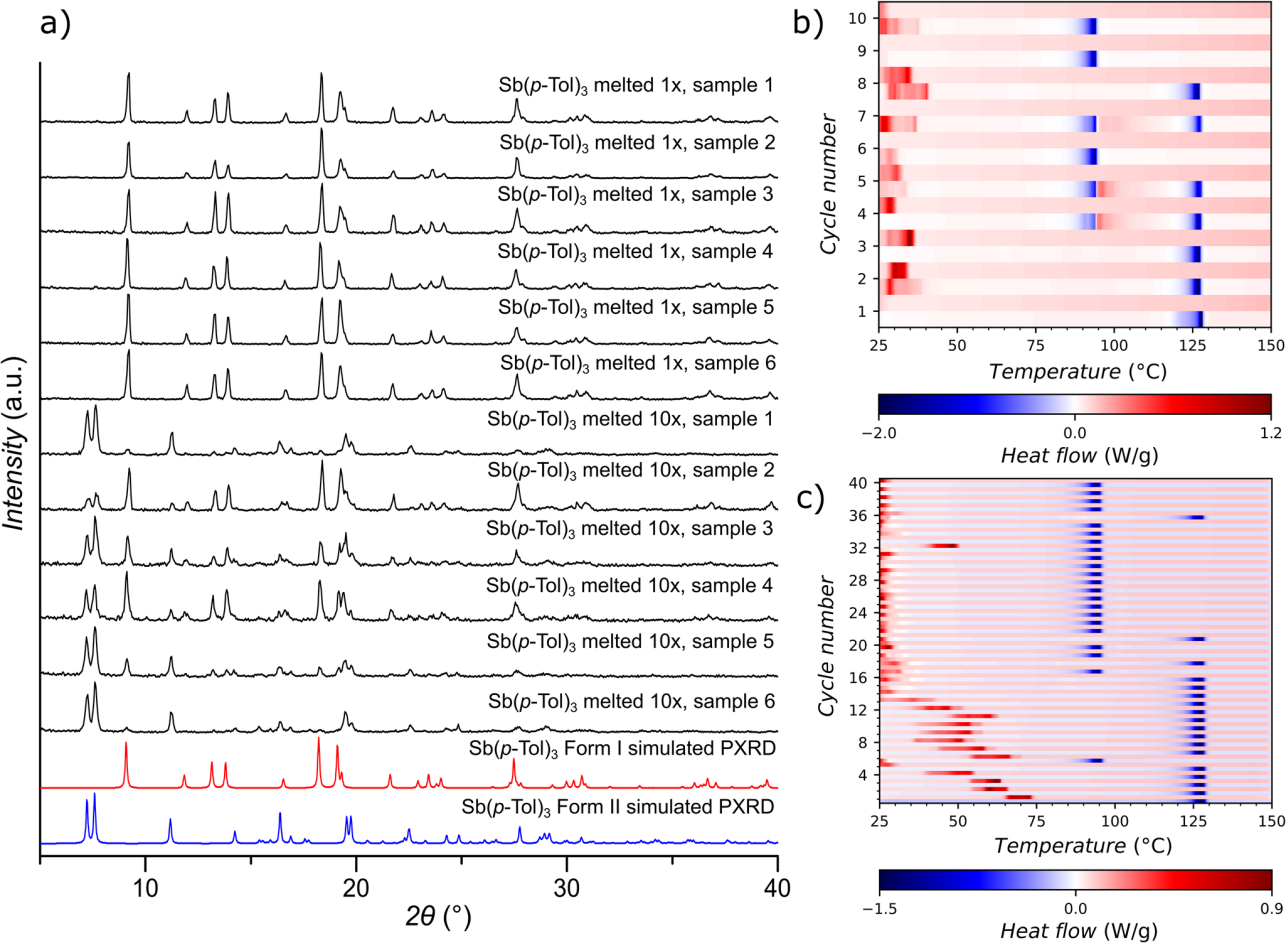
(a) Comparison of PXRD patterns of six samples of Sb(*p‐*Tol)_3_ that were each melted and solidified once prior to data collection, followed by PXRD patterns of another six samples of Sb(*p‐*Tol)_3_ that were each melted and solidified 10 times, along with calculated PXRD patterns for Form I and II. In each experiment the sample weight was ca. 15 mg. (b) Waterfall plot of tenfold DSC experiment (25–150°C, 5°C min^−1^) performed on Sb(*p‐*Tol)_3_ synthesized at McGill University. (c) Waterfall plot of fortyfold DSC experiment (25–150°C, 5°C min^−1^) performed at the University of Zagreb on Sb(*p‐*Tol)_3_ synthesized at Babeș‐Bolyai University.

The inability to obtain Form II by a simple melting and crystallization protocol led us to consider whether a more elaborate treatment, that is, performing melting and solidification more than once, could be effective for the synthesis of Form II. For that purpose, six freshly prepared samples of Sb(*p‐*Tol)_3_ Form I were heat‐cycled 10 times, with each sample being molten using an oil bath, left to solidify at room temperature over a period of 2 min, and then melted again. After the 10th cycle, each sample was collected, manually ground to a powder, and analyzed by PXRD, which revealed a noticeable presence of Form II (Figure 2a). While a pure sample of Form II was not obtained, these observations indicate that a repeatable appearance of Form II is achievable via multiple melting and solidification cycles. This was supported by an additional DSC experiment involving ten cycles of melting and cooling (Figure 2b, also Figures S39–S50). The first temperature ramp from 25°C to 150°C at a rate of 5°C min^−1^ (Figure S41) revealed an endothermic event with a maximum at 128°C, which was interpreted as the melting point of Form I. While the two subsequent cycles also showed the presence of only Form I, in almost all subsequent cycles a different endothermic event was also observed at 94°C, interpreted as the melting point of Form II (Figures S42–S50). This additional endotherm appeared either alone or in the presence of the endothermic event at 127°C, suggesting concomitant [22, 23] polymorphism. The propensity to obtain Form II by multiple melt recrystallization was also seen for samples synthesized and analyzed independently at Babeș‐Bolyai University, Romania, and University of Zagreb, Croatia (Figures 2c and S67–S81, for detailed description of DSC observations, see Supporting Information), and was also observed at higher (10°C min^−1^) or lower (2.5°C min^−1^) heating and cooling rates (Figures S74–S77 and S78–S81). The observed behavior might be reminiscent of 4‐nitrotoluene, reported to form a metastable polymorph upon repeated heating and cooling [53]. Propensity to form the metastable Sb(*p‐*Tol)_3_ polymorph is, however, more remarkable: for 4‐nitrotoluene the metastable form was reported to appear in ca. 20% cases, while in the case of Sb(*p‐*Tol)_3_ this number can be ∼60% or higher. While the reason for an apparent propensity of Sb(*p‐*Tol)_3_ to form the metastable polymorph upon extensive thermal cycling remains unclear [55], we also note that it does not appear to depend on the rate of sample heating or cooling, as DSC experiments at 2.5, 5, and 10°C min^−1^ all showed similar results (see Figures S70–S81). Sample purification history, however, was found to play a role; using a sample of crude Sb(*p‐*Tol)_3_ obtained directly from the synthetic procedure revealed Form II already in the first DSC cycle, whereas a sample of re‐crystallized Sb(*p‐*Tol)_3_, purified by additional recrystallizations from ethanol and hexanes, led to the appearance of Form II only in later DSC cycles and always as a minor component of a mixture with Form I (Figures S51–S60). The addition of impurities selected to mimic those likely to be found in the crude product, such as vacuum grease or silicon oil was also found to have an effect, with Form II not observed upon DSC thermal cycling in presence of the former (Figures S61–S66).

Accessibility of Form II enabled further characterization and comparison to Form I. Integrating the DSC signal for a sample of recrystallized Sb(*p‐*Tol)_3_ (see Supporting Information: Synthetic Procedures section for purification protocol, Figures S13, S14, S16, S51–S55) in which only the melting of Form I was observed gave the enthalpy of melting (Δ*H*_m_) for that polymorph as 28.0 kJ mol^−1^. Analogously, the Δ*H*_m_ for Form II was found to be 26.6 kJ mol^−1^, by integrating the endothermic signal in a DSC cycle of crude Sb(*p‐*Tol)_3_ (see Supporting Information: Synthetic Procedures section for purification protocol, Figures S56–S60), which exhibited only an endotherm at 91°C and was therefore considered to consist only of Form II. The measured Δ*H*_m_ values indicate that Form I should be more stable, being ca. 1.4 kJ mol^−1^ more exothermic. This is consistent with slurrying experiments, conducted on mixtures of Form I and II in a range of liquids (Figures S87 and S88): in all cases, except water, slurrying led to complete conversion to Form I. Slurrying with water led to incomplete conversion, likely due to poorer Sb(*p‐*Tol)_3_ solubility. The metastable nature of Form II was also evident from variable‐temperature PXRD (VT‐PXRD) monitoring of a mixture of the two polymorphs while heating from 40°C to 110°C. Rietveld analysis [56] revealed essentially complete transformation of the initial mixture into Form I upon heating (Figures 3a,b, larger images shown in Figures S25 and S26), with maximum transformation rate at ca. 63°C. Moreover, storing a sample of the two polymorphs at 60°C led to complete conversion to Form I after ca. 18 h. Finally, conversion to Form I was observed even upon attempted characterization of initially Form II by ^13^C{^1^H} magic angle spinning (MAS) solid‐state nuclear magnetic resonance (SSNMR) while inside the sample rotor (Figures S30–S32).

**FIGURE 3:**
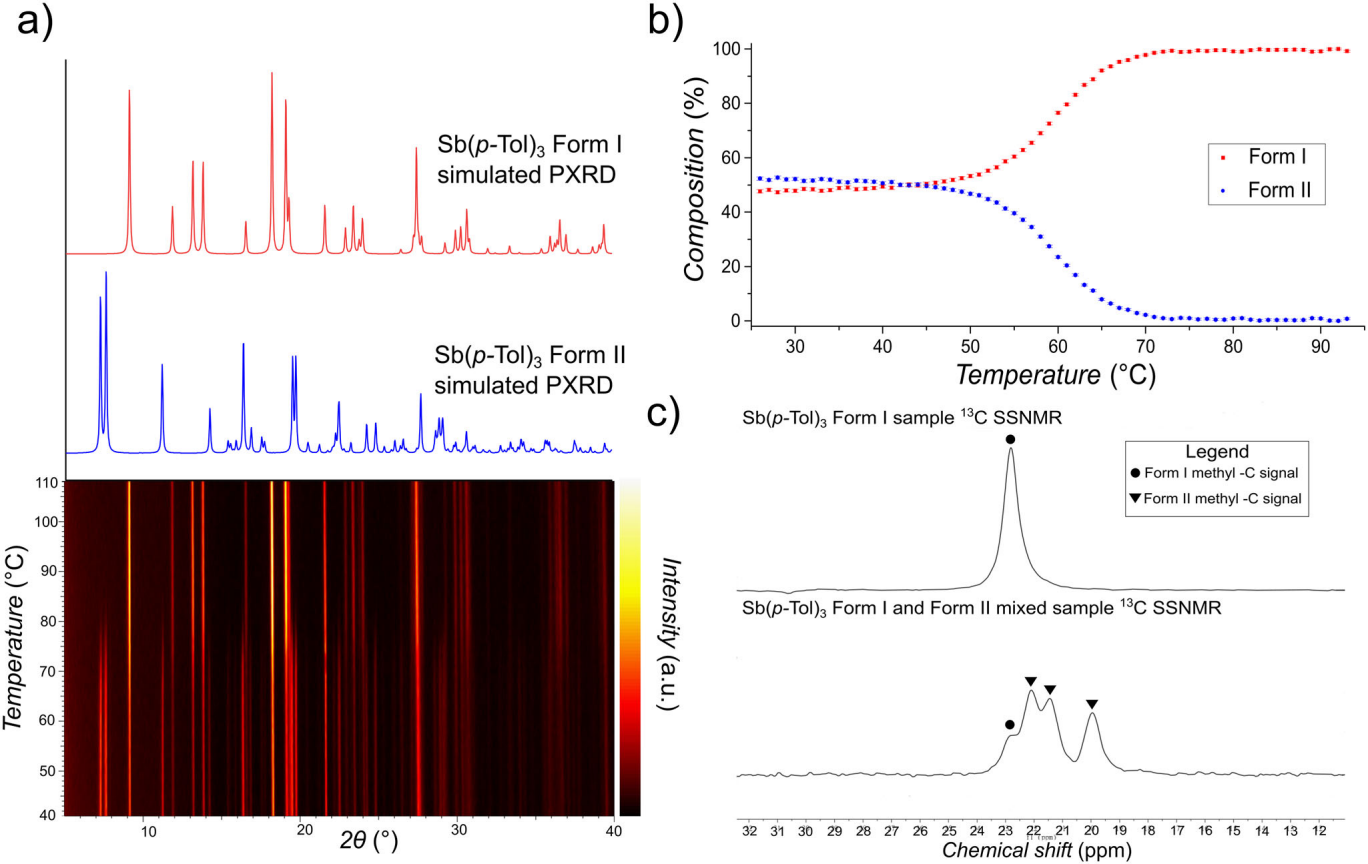
(a) Variable‐temperature PXRD pattern (heating rate 0.17°C min^−1^) showing conversion of a mixture of Forms I and II to Form I only; (b) profile of this transformation based on Rietveld refinement; (c) comparison of ^13^C{^1^H} SSNMR alkyl‐carbon region for a sample of Form I and a mixture of Form II and a Form I impurity developed during measurement. Full SSNMR spectra are provided in the Supporting Information.

The two polymorphs were characterized by Fourier‐transform infrared attenuated total reflectance (FTIR‐ATR), as well as by MAS‐SSNMR ^13^C{^1^H} spectroscopies (Figure 3c, also Figures S27–S34). The ^13^C{^1^H} SSNMR spectrum of Form I Sb(*p‐*Tol)_3_ reveals only one resonance that can be assigned to a methyl carbon signal, consistent with the crystal structure in which a three‐fold rotation axis renders each *p‐*tolyl group symmetrically equivalent. This contrasts with the SSNMR spectrum of a sample containing Form I and Form II, which shows three additional distinct methyl environments, consistent with three crystallographically non‐equivalent *p‐*tolyl groups in the asymmetric unit of Form II. Overall, the slurrying, ageing, VT‐PXRD and SSNMR experiments all show that Form II can convert to Form I at a range of temperatures prior to melting which, together with DSC measurements, suggests a monotropic relationship.

The instability of Form II with respect to Form I is notable when considering that calculated density for Form II (1.42 g cm^−3^) is higher than for Form I (1.37 g cm^−3^) [57, 58, 59]. In order to resolve this conundrum, and also evaluate the role of Sb···Sb interactions in Form II, the relative stability of Forms I and II was assessed by periodic density‐functional theory (DFT) calculations in CASTEP [60] (v25.1.2), using the LDA [61] and PBE [62] functionals, and the PBE functional with the addition of different semi‐empirical dispersion correction (SEDC) approaches: Grimme‐D3(0) [63], Grimme‐D3(BJ) [64], and Grimme‐D4 [65, 66, 67]. Variable‐cell geometry optimizations consistently showed that using the D3(0) dispersion correction resulted in a unit cell that was the closest to the experimental structure (see SI for full details on computational studies). This method also indicated that Form II should be 2.21 kJ mol^−1^ less stable than Form I, in good agreement with DSC results (1.4 kJ mol^−1^) and the observed tendency of Form II to convert into Form I. Notably, D3(BJ) and D4 approaches predicted Form II to be more stable, contradicting the experimental observations, and highlighting the importance of evaluating the dispersion correction when modeling supramolecular materials.

Theoretical analysis of the stability of selected supramolecular motifs indicated that the phenyl embrace in Form I was associated with a strong attractive dimer interaction energy of −79.9 kJ mol^−1^ per Sb(*p‐*Tol)_3_ pair, which is around −26.6 kJ mol^−1^ per pair of phenyl groups. In contrast, the dimer involving the short Sb···Sb contact in Form II shows an interaction energy of ca. −26.1 kJ mol^−1^ per pair of Sb(*p‐*Tol)_3_ molecules. Although the interaction energy for the Sb···Sb dimer appears smaller, it should be kept in mind that the phenyl embrace involves C‐H···π interactions involving six aromatic rings over an area larger than the intermolecular contact in Form II. From that perspective, the dimer interaction in Form II is notable and might be comparable in strength to some hydrogen and halogen bonds [68, 69].

While isolated dimers of molecules involving trivalent pnictogens have previously been modelled, such work has focused mostly on phosphorus and, in most cases, dealt with theoretical, gas‐phase models. As a notable exception, Moilanen et al. have performed gas‐phase modeling of a SbCl_3_ dimer (Sb···Sb contact of 3.78 Å) whose geometry was extracted from a solid‐state complex with 1,3,5‐triacetylbenzene [70]. Gas‐phase modeling of that isolated dimer showed that the Sb···Sb contact is repulsive by ca. 3 kJ mol^−1^ and is likely a result of crystal packing [71]. Indeed, the original solid‐state structure shows that this particular dimer is bridged, and likely to be held together, by a pair of oxygen atoms of from neighboring acetyl groups, with short (3.06 Å) Sb···O contacts. Similarly, theoretical modeling of isolated dimers by Zahn et al. found that Sb···Sb dimers of SbCl_3_ are not stable [72].

Consequently, as the prior literature does not appear to offer organoantimony models suitable for immediate interpretation of the nature of the Sb(*p‐*Tol)_3_ dimers in Form II, we conducted preliminary theoretical studies of the electrostatic surface potential (ESP) maps of the dimer and individual Sb(*p*‐Tol)_3_ molecules in the gas phase, bond critical point (BCP) analysis, residual charge density analysis, and explored the distance‐dependent interaction energy profile of the dimer. The analysis of the ESP map for a gas‐phase molecule of Sb(*p*‐Tol)_3_, in the conformation obtained from the Form II crystal structure, reveals an area of significant negative potential centered on the antimony atom, which is surrounded by areas of positive ESP ascribable to the σ‐hole regions of the three C‐Sb bonds (Figures 4a and S9). Moreover, areas of positive ESP on the antimony atom are adjacent to regions of positive ESP associated with *ortho*‐hydrogen atoms of the attached phenyl substituents.

**FIGURE 4:**
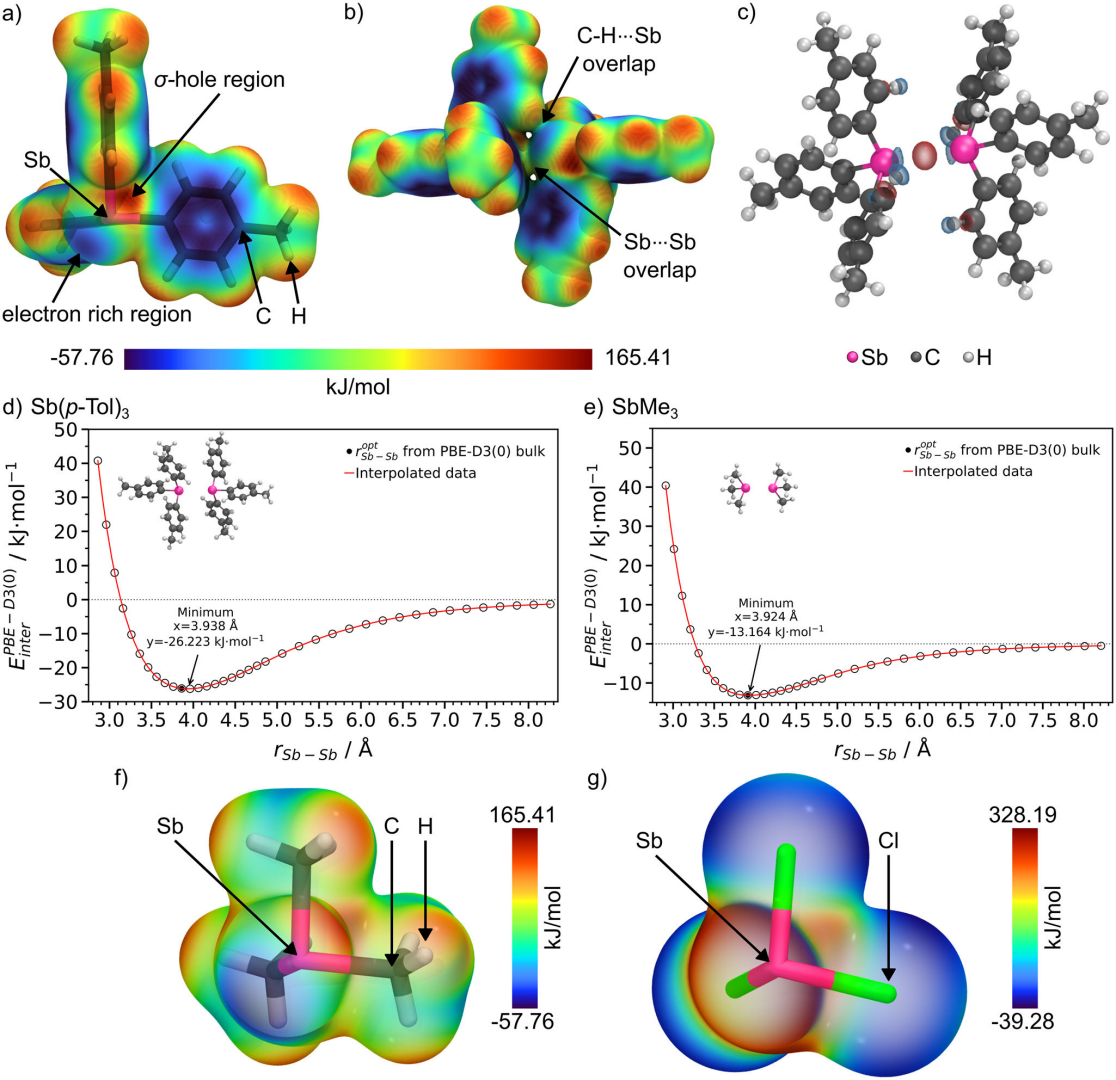
(a) ESP map for a single molecule of Sb(*p*‐Tol)_3_ with a translucent surface highlighting both the electron‐rich and *σ*‐hole regions on the Sb atom. (b) ESP map of a dimer of Sb(*p*‐Tol)_3_ in the Form II configuration which exhibits overlaps of the ESP map, suggesting possible CH···Sb and Sb···Sb contacts. (c) Render of electron density difference of Sb(*p*‐Tol)_3_ dimer from the Form II configuration with respect to the individual Sb(*p*‐Tol)_3_ molecules, where translucent red regions indicate an increase in electron density and translucent blue regions represent a decrease. Electron density regions are rendered at the ±5 × 10^−4^ e•a_0_^−3^ isosurfaces. (d) Interaction energy with respect to Sb‐Sb distance using the Form II dimer configuration of Sb(*p*‐Tol)_3_ from periodic DFT calculations (filled black dots). (e) Interaction energy with respect to Sb‐Sb distance using the dimer configuration of SbMe_3_ from periodic DFT calculations (filled black dots). Interaction energies (*E*_inter_) in panels (d) and (e) were obtained from single point energy calculations (black rings) with the PBE‐D3(0) method applied and smooth traces of the data were generated through cubic splines interpolation (red lines), permitting the optimal separation and accompanying energy minimum of the gas phase dimers to be determined. f) ESP map for a single molecule of SbMe_3_ with a translucent surface, and g) ESP map for a single molecule of SbCl_3_ with a translucent surface. All ESP maps are rendered at the 5 × 10^−3^ e•a_0_^−3^ isosurface

The ESP maps of the supramolecular dimer (Figures 4b and S9), again with a geometry as found in the structure of Form II, show for each Sb(*p*‐Tol)_3_ molecule a direct overlap between the area of negative ESP on the antimony atom with the areas of positive ESP from the adjacent molecule that are related to the antimony atom and to one of the *ortho*‐positioned C‐H groups. We note that such a dimer geometry, where the area of negative ESP of a pnictogen atom interacts with areas of positive ESP belonging to a pnictogen atom and an attached substituent of a neighboring molecule, is reminiscent to the geometries of model gas‐phase dimers of PH_2_Cl, PH_2_F, and related molecules, studied previously [73, 74, 75, 76]. While Politzer et al. [73] and Varadwaj et al. [74] interpreted such contacts focusing on electrostatic terms, Setiawan et al. [75] and Oliveira et al. [76] used coupled‐cluster level of theory and suggested a partial electron sharing character.

Analysis of the Sb(*p*‐Tol)_3_ dimer in Form II using the quantum theory of atoms in molecules (QTAIM) [77] approach was done using the Critic2 code [78, 79] to evaluate first‐order saddle points, that is, BCPs, and their associated bond paths [80, 81, 82, 83]. The analysis revealed that the highest electron density BCPs (0.068 electrons Å^−3^) are located between the antimony atoms of two neighboring molecules (see Figures S6–S9). Analogous calculations for the crystal structure of Form I showed that the BCPs of highest electron density in that structure are lower (0.045 electrons Å^−3^) than observed for the Form II Sb···Sb contacts and located between Sb(*p*‐Tol)_3_ molecules, corresponding to the six C‐H···π interactions in the phenyl embrace (see Supporting Information). As the existence of a BCP does not necessarily signify attraction, we also conducted residual electron density analysis (Figure 4c), which showed an area of enhanced electron density for the Sb(*p*‐Tol)_3_ dimer compared to individual molecules, located in the center of the supramolecular complex. At the same time, areas of decreased electron density were observed in the vicinity of both antimony atoms, as well as two of the *ortho*‐positioned C‐H groups. Such behavior lends itself to interpretation as a four‐center stabilizing interaction [72, 75].

We have also performed an analysis of the interaction energy profile with respect to the Sb···Sb separation, again keeping the conformation of individual molecules fixed. The interaction energy profile calculated using the PBE‐D3(0) approach revealed a minimum of −26.22 kJ mol^−1^ centered at 3.94 Å, which is ca. 0.1 Å longer than the Sb···Sb separation in the DFT‐optimized structure of Form II (Figure 4d). If the calculation is done using the PBE approach, but without any dispersion correction applied, the interaction energy again exhibits an attractive minimum (−5.44 kJ mol^−1^), now located at a 4.36 Å separation (Figure S4b). Using the PBE‐only profile, the interaction energy for the Sb···Sb separation found in the optimized structure of Form II is found to be −2.87 kJ mol^−1^. The comparison of PBE‐ and PBE‐D3(0)‐based calculations indicates that the interaction energy for Sb(*p*‐Tol)_3_ dimers is negative and dominated (ca. 90%) by dispersion.

It is well established that the magnitude of dispersion interactions grows with molecular size [84], although some exceptions have been noted [85]. To evaluate the effect of molecular size, we performed a brief investigation on the recently reported crystal structure of trimethylantimony (SbMe_3_), which also exhibits short Sb···Sb contacts (ca. 3.84 to 3.87 Å, depending on temperature) [44]. The structure of SbMe_3_ was subject to a variable‐cell optimization in CASTEP, with the PBE‐D3(0) method employed (see Supporting Information for details). From the resulting optimized structure, the dimer interaction energy was calculated to be −12.98 kJ mol^−1^ with a Sb···Sb distance of 3.81 Å. We subsequently calculated the interaction energy profile with respect to the Sb···Sb separation (Figure 4e). With the PBE‐D3(0) method applied, a minimum of −13.16 kJ mol^−1^ was observed for an Sb···Sb distance of ca. 3.92 Å, which is 0.12 Å longer than the optimized bulk structure. If the interaction energy versus distance profile is calculated using the PBE approach in the absence of a dispersion correction, an attractive minimum is again observed, this time of magnitude −6.17 kJ mol^−1^ and at an Sb···Sb distance of ca. 4.04 Å (Figure S5b). For the Sb···Sb separation identical to the one observed in the optimized bulk structure, the interaction energy based on the dispersion‐free PBE approach was found to be −5.57 kJ mol^−1^. Therefore, in the case of SbMe_3_, the dimer interaction energy again appears to be negative and dominated by dispersion interactions, but to a lesser extent (ca. 57%) than found in Sb(*p*‐Tol)_3_ Form II. These values are comparable to those recently found for modeling of halogen bonding to polycyclic aromatic hydrocarbon π‐systems [86]. We note that earlier studies of gas‐phase dimers of pnictogen‐based molecules in general, conducted by the Hey‐Hawkins group at the spin‐component scaled Møller‐Plesset and coupled‐cluster levels of theory, have also found a significant dispersion component [72].

The herein investigated organoantimony dimers found in Sb(*p*‐Tol)_3_ Form II and solid SbMe_3_ appear to be significantly stabilized on their own and not to be solely a result of crystal packing. This contrasts with previous studies which found that dimers based on short Sb···Sb contacts would not be stable. Those studies have, however, focused on dimers of SbCl_3_ [71, 72], a molecule in which the antimony atom exhibits an entirely positive ESP, which is different to Sb(*p*‐Tol)_3_ and SbMe_3_ (Figure 4f,g, also Figures S9 and S10), and likely to make any direct contacts between antimony atoms repulsive. A further, detailed understanding of this difference, as well as an analysis of the non‐dispersion factors that lead to stabilization of Sb···Sb‐connected dimers of Sb(*p*‐Tol)_3_ and SbMe_3_ would require a higher level of theory. Nevertheless, we note that the herein presented analysis can be related to a previous theoretical study by Klinkhammer and Pyykkö [87] of antimony···antimony contacts in Sb_2_H_4_, used as a model of thermochromic distibines such as 2,2',5,5'‐tetramethyldistibolyl [35, 36, 37, 38, 39, 40, 41, 42], who found that the dimers would likely be held by dispersion. Such a perspective suggests that the short Sb···Sb contacts observed in solid distibines might be of similar nature as those found in solid‐state dimers of Form II Sb(*p*‐Tol)_3_ and SbMe_3_.

## 3 Analysis of the Cambridge Structural Database (CSD)

The observation and calculated stability of the short Sb···Sb contacts in Form II led us to explore how common such a motif is in the known crystal structures of trivalent organoantimony compounds. For this, a broad search of the CSD [88] was designed to target inter‐ and intramolecular Sb···Sb contacts longer than the sum of covalent radii (2.80 Å) [89], but shorter than 5.12 Å, which is 1 Å greater than the sum of the van der Waals radii of two Sb atoms according to Mantina et al. (4.12 Å) [45, 46, 47, 48]. Whereas the most recent work indicates the sum of the van der Waals radii (Σ*r*_vdW_) is almost 5 Å, we decided to focus the analysis around the more conservative assessment of 4.12 Å, in that way addressing only the behavior of the shortest Sb···Sb contacts. The search recorded the distance between antimony centers (*d*_Sb···Sb_), as well as each of the angles formed by the Sb···Sb contact and the C‐Sb covalent bonds on each antimony atom. Labeling the two antimony atoms as Sb_i_ (where i = 1, 2), there are a total of six such angles, labeled *φ*_i,j_ (where i = 1, 2 and identifies the Sb atom, and j = 1, 2, or 3 and identifies a substituent on a particular Sb atom). The largest *φ*_i,j_ around each of the two antimony centers is designated *ϕ*_i_ (where i = 1 or 2, Figure 5). The search was designed not to include polymers and network solids. For structure re‐determinations, the database entries were first examined visually, after which only the first report found to possess full three‐dimensional atomic coordinates was included in the search results. Polymorphs were encountered in several cases, including triphenylantimony, and were all included in analysis (CSD codes CUDHOY, CUDHOY02, GAVKOF, GAVKOF01, QOSXIF, QOSXIF01, QOSXIF02, VENCOG, VENCOG02, WEKCEW, WEKCEW01, ZZZEHA01 and ZZZEHA02) [41, 90, 91, 92, 93, 94, 95, 96, 97, 98, 99]. Overall, from 533 structural entries, 74 were excluded due to being re‐determinations, residual polymeric structures, or structures not relevant to this search (e.g., very large cluster systems, see Supporting Information).

**FIGURE 5:**
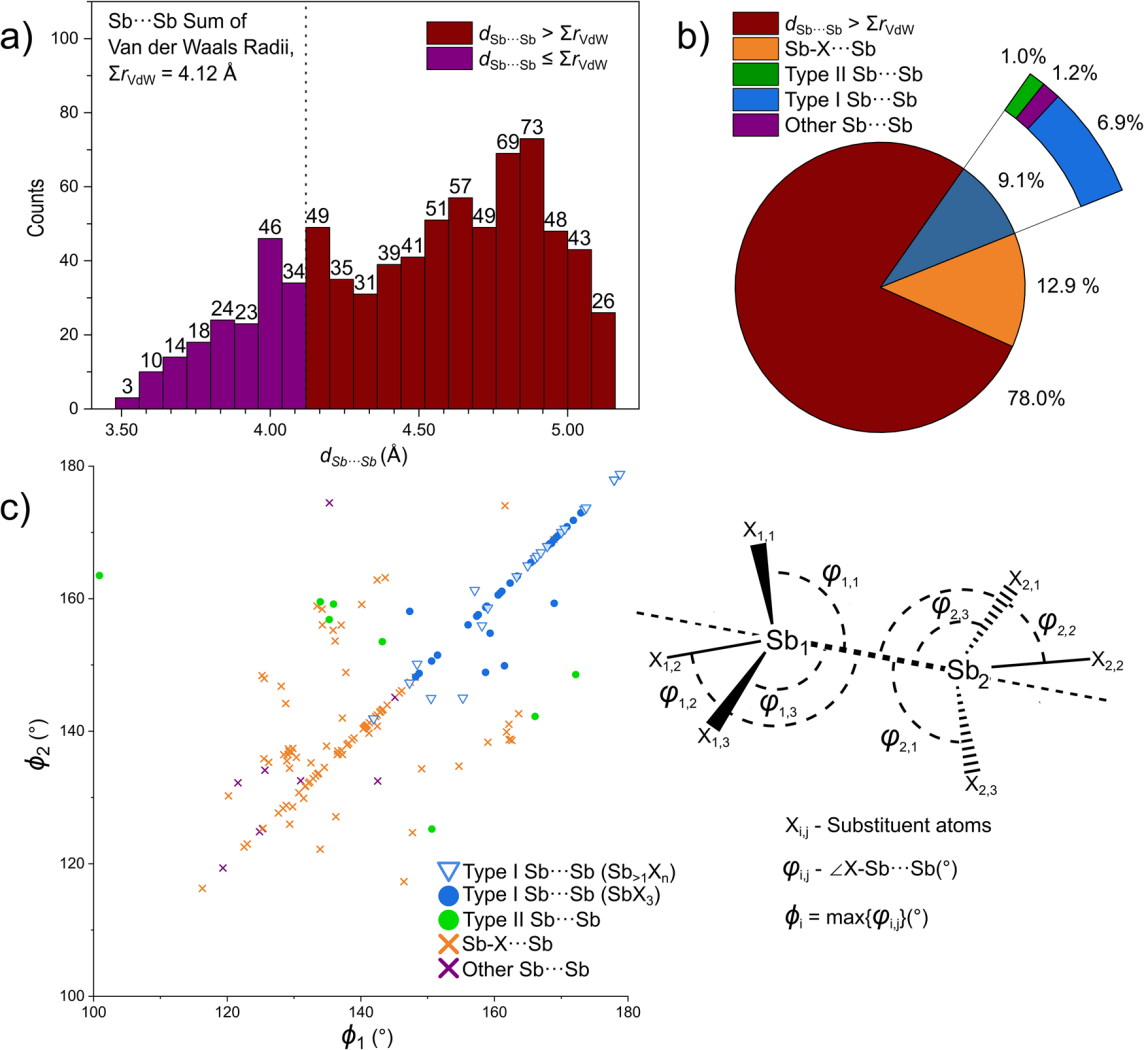
(a) A histogram of Sb···Sb distances below 5.12 Å (sum of *r*_vdW_ for a pair of Sb atoms + 1 Å), with the Σ*r*_vdW_ indicated by a dashed line; (b) distribution of different Sb···Sb pairs found in the CSD based on the herein proposed categorization: Type I, Type II, contacts that are a part of a Sb‐X···Sb (X = bridging atom) unit, as well as miscellaneous (“Other”) contacts with geometries that do not readily fit into other ones; (c) a scatterplot distribution of CSD entries exhibiting different types of Sb···Sb contacts, and a schematic illustrating the geometrical parameters *φ*_i,j_, *X*_i,j_, and Sb_i_. The entries are categorized by the largest angle *φ*_i,j_ for each Sb atom in a pair. For clarity, Sb···Sb pairs with 4.12 Å < *d* are omitted, and the plot area is constrained to 100°–180°, which additionally excludes one Sb‐X···Sb hit at (75°, 159°).

The search (Figure 5a, Table 2) yielded a total of 783 Sb···Sb pairs across 459 CSD entries, as sometimes more than one *d*_Sb···Sb_ contact was found per single crystal structure entry. Among the 783 hits, 172 were found to be contacts shorter than Σ*r*_vdW_ (4.12 Å), distributed over 123 unique CSD structure entries. Notably, there are 71 hits over 61 entries that exhibit contacts directly between pairs of antimony atoms, similar to the Sb···Sb contact seen in Form II Sb(*p*‐Tol)_3_. Of these, 21 pairs (over 17 entries) are short contacts resembling those noted by Ashe and co‐workers, i.e. involving Sb^II^ centers in distibines, or involving Sb^I^ centers in tristibanes. The remaining 101 hits, distributed over 65 entries, represent structures in which pairs of Sb atoms are bridged by a third atom, typically in Sb_2_X_2_ rings (X = chalcogen or halogen) with *ϕ*_i_ ∼140° (Figure 5b, Table 2) and ∠X···Sb‐X angle < 80°. While such bridged systems are not relevant for this study, we note that the appearance of short Sb···Sb contacts in sulfur‐bridged dithiocarbamates was previously discussed [100, 101].

**TABLE 2:** Overview of the results of the search of the CSD for structures exhibiting Sb···Sb contact lengths between pairs of antimony atoms (*d*_Sb···Sb_) that are smaller or 1 Å greater than the sum of the van der Waals radii according to Mantina et al. [45] (Σ*r*_vdW_, 4.12 Å).

*d*_Sb···Sb_ range	(Sb···Sb pairs/total Sb···Sb pairs | % of Sb···Sb pairs) [structures/total structures | % of structures]^a^			
> Σ*r*_vdW_	(611/783 | 78.0 %) [380/459 | 82.8%]			
≤ Σ*r*_vdW_	(172/783 | 21.8%) [123/459 | 26.8%]			
	Sb‐X···Sb (101/783 | 12.9%) [65/459 | 14.2%]	Sb···Sb (71/783 | 9.1%) [61/459 | 13.9%]		
		Type I (54/783 | 6.9%) [48/459 | 10.5%]	Type II (8/783 | 1.0%) [7/459 | 1.5%]	Other (9/783 | 1.2%) [8/459 | 1.7%]

*Note*: The results are shown as the number of Sb···Sb contacts (in parentheses) and structures (in square brackets) for a particular *d*_Sb···Sb_ range, compared with the total number of Sb···Sb contacts and structures, respectively.

^a^ As a single crystal structure entry can contain different types of Sb···Sb contacts, or several instances of a particular type of contact, the total sum of structures assigned to different contact types can exceed the total number of structures in the search.

The systematic analysis of geometries of supramolecular interactions other than hydrogen bonds is an active area of research [102]. To analyze the direct, short Sb···Sb contacts relevant to this study, we used a categorization analogous to the one used for halogen···halogen contacts [75, 76, 102, 103, 104]. Specifically, halogen···halogen contacts are typically categorized into Type I and Type II interactions, depending on the angle formed by the halogen···halogen interaction and the covalent bond between each halogen atom and its belonging substituent. To apply this kind of analysis to Sb···Sb contacts, we considered only the largest of three unique ∠Sb···Sb‐X angles {*φ*_i,j_} for each of the two Sb atoms in contact. Specifically, contacts accommodating the criterion *ϕ*_1_ ≅ *ϕ*_2_ ≅ 150°–180° were designated as Type I, while those with *ϕ*_1_ ≅ 150°–180° and *φ*_2,1_ ≅ *φ*_2,2_ ≅ *φ*_2,3_ ≅ 90°–150° were designated as Type II (Figure 5c). This proposed categorization is strictly based on geometry and, in principle, structures from each type could be consistent with interactions involving a σ‐hole on the pnictogen atom, sometimes termed stibium or pnictogen bonds [76, 105, 106]. The Type I contacts were observed most often (54 hits over 48 CSD entries) [35, 36, 39, 40, 41, 44, 92, 98, 107, 108, 109, 110, 111, 112, 113, 114, 115, 116, 117, 118, 119, 120, 121, 122, 123, 124, 125, 126, 127, 128, 129, 130, 131, 132, 133, 134, 135, 136, 137, 138, 139, 140, 141, 142, 143], while the Type II Sb···Sb contacts, as well as miscellaneous Sb···Sb short contacts that could not be readily assigned into Type I or Type II, were less abundant, with 8 [144, 145, 146, 147, 148] and 9 hits [149, 150, 151, 152, 153, 154, 155], respectively. The Type I contacts were observed frequently between symmetrically equivalent Sb atoms interacting directly opposite to a given substituent, with *ϕ*_1_ and *ϕ*_2_ being in the range c.a. 150°–180°, making the remaining *φ*_i,j_ close to 90° in most cases (Figure 5). Overall, the analysis of the CSD shows the formation of short, direct Sb···Sb contacts across a diversity of organoantimony(III) compounds, and can be seen in structures of some of the simplest members of the series, such as SbMe_3_ [44] or triphenylantimony [98] (see Figures S89–S93).

## 4 Conclusion

In summary, polymorphism in the organometallic compound Sb(*p*‐Tol)_3_ highlights the formation of supramolecular solid‐state dimers between organoantimony(III) centers characterized by a Sb···Sb distance shorter than the sum of van der Waals radii. Theoretical modeling indicates that the interaction energy for the Sb···Sb connected Sb(*p*‐Tol)_3_ dimer in Form II is in the range of ca. −26 kJ mol^−1^ and dominated by dispersion effects. Similar stabilization (−13 kJ mol^−1^), with a lesser dispersion contribution, is here also found for a similar supramolecular dimer from the recently reported crystal structure of SbMe_3_ [44]. Whereas the work of Ashe, and others, has previously associated short and direct antimony···antimony contacts with a small family of thermochromic organoantimony(II) species, that is, tetraorgano distibines [35, 36, 37, 38, 39, 40, 41, 42], the herein presented theoretical, experimental and database analysis suggests a broader perspective, in which the short, direct (i.e., with antimony atoms not bridged by other atoms) Sb···Sb contacts, in a way possibly analogous to Type I or Type II halogen···halogen interactions [76, 103, 104, 106], are a common occurrence in a greater range of organoantimony solids [156]. Moreover, we note that the short Sb···Sb contacts in distibines were proposed by Pyykkö [87] to be largely a result of van der Waals forces. This observation is consistent with the herein observed significant role of dispersion in stabilizing solid‐state Sb(*p*‐Tol)_3_ and SbMe_3_ dimers, and reinforces the proposed view of short Sb···Sb contacts as a general hallmark of trivalent organoantimony solids. That view is herein based on a conservative assessment of the van der Waals radius of antimony (2.06 Å), and will certainly be strengthened by considering the larger value (2.47 Å) for the radius, proposed by Alvarez and recently implemented by the CSD [45, 46, 47, 48, 88, 106]. While this work provides a preliminary analysis of the nature of short Sb···Sb contacts in organoantimony solids, further theoretical modeling is required to develop a clear understanding: a goal that is well‐aligned with ongoing discussions on the nature and terminology related to supramolecular interactions [157, 158] and could open opportunities in crystal engineering and reactivity that are not seen in the chemistry of arsenic or phosphorus homologs [1, 2, 3, 4, 5, 6, 7, 8, 9, 10, 11, 12, 13, 14, 15, 16, 17, 18].

We also highlight a route for the reproducible formation of the novel, metastable polymorph of Sb(*p‐*Tol)_3_, based on exposing samples of the compound to multiple cycles of melting and crystallization. While melt recrystallization was previously reported for accessing metastable solid forms, not accessible via solution [53, 159, 160, 161, 162, 163], systematic studies exploring this phenomenon, using samples and measurements at different laboratories, are not common. The reasons for the apparent promotion of Form II upon thermal cycling remain unclear, and might be related to a number of factors, including nucleation effects related to the sample environment (e.g., preferred nucleation of Form II from the melt, influence of container surface), as well as generation of impurities that interfere with the crystallization process or affect relative polymorph stability [164, 165]. The use and systematic investigation of thermal cycling to frequently generate a serendipitously observed metastable polymorph of a long‐known compound is of interest for developing polymorph screening [166, 167] and highlights the potential impact of thermal processing in late‐appearing polymorphism [165, 168]. We are currently exploring other factors that might be influencing the polymorphism of Sb(*p*‐Tol)_3_ upon thermal cycling, such as the choice of vessel material, and are exploring polymorphism of related organometallics as a means to identify further examples of short Sb···Sb motifs.

## Author Contributions

**Imogen Suh**: investigation, writing – original draft, writing – review and editing, visualization, methodology, formal analysis. **Michael Ferguson**: investigation, writing – original draft, writing – review and editing, validation, visualization, methodology, software, formal analysis. **Filip Topić**: investigation, formal analysis. **Fu‐Qiang Gong**: investigation, writing – original draft, writing – review and editing, methodology, validation, visualization, formal analysis. **Mihails Arhangelskis**: investigation, writing – original draft, writing – review and editing, funding acquisition, methodology, formal analysis. **Hatem M. Titi**: investigation, writing – original draft, writing – review and editing, visualization, formal analysis. **D. Scott Bohle**: investigation, writing – original draft, writing – review and editing, funding acquisition, supervision, conceptualization. **Vinko Nemec**: investigation, writing – original draft, writing – review and editing, formal analysis, methodology. **Levente Kiss**: investigation, writing – original draft, writing – review and editing, methodology. **Andrew J. Morris**: investigation, conceptualization, writing – original draft, writing – review and editing, methodology, validation, software, formal analysis, supervision, resources. **Cristian Silvestru**: supervision, resources, investigation, writing – original draft, writing – review and editing, conceptualization, funding acquisition. **Dominik Cinčić**: writing – original draft, writing – review and editing, visualization, investigation, conceptualization, funding acquisition, supervision, resources, formal analysis, methodology. **Tomislav Friščić**: conceptualization, writing – original draft, writing – review and editing, funding acquisition, data curation, supervision, resources, project administration, investigation, methodology, visualization.

## Conflicts of Interest

The authors declare no conflicts of interest.

## Supporting information



Additional details on experimental, computational, and crystallographic methods, as well as additional PXRD, DSC and mass‐spectrometry results are available as Supporting Information (SI) in pdf format. Crystallographic information files for our herein described re‐determination of Sb(*p*‐Tol)_3_ Form I and novel Form II are available as crystallographic information files (.cif) for download free of charge, and can also be accessed via the Cambridge Crystallographic Data Centre (CCDC, deposition codes 2175193 and 2175192 for Sb(*p‐*tol)_3_ Form I re‐determination, and Form II respectively). The authors have cited additional references within the Supporting Information.**Supporting File 1**: anie73176‐sup‐0001‐SuppMat.pdf.

**Supporting File 2**: anie73176‐sup‐0002‐cif.zip.

## Data Availability

The data that support the findings of this study are available in the Supporting Information of this article, and individual datasets are available on request from author.
