# Supplementary material for: Short Sb···Sb Contacts in Polymorphic Tris(p‐Tolyl)Antimony: A Hallmark of Organoantimony(III) Solid‐State Chemistry?
Source: Angew Chem Int Ed Engl. 2026 Aug 12;65(41):e5894899. doi: 10.1002/anie.5894899 (PMC13638092; doi:10.1002/anie.5894899)
Supplement: Supplementary file 1 [file ANIE-65-e5894899-s001.pdf]

Supplementary Information

Short Sb···Sb Contacts in Polymorphic Tris(*p*-tolyl)antimony: a Hallmark of Organoantimony(III) Solid-State Chemistry?

Imogen Suh,[a] Michael Ferguson,[b] Filip Topić,[a] Fu-Qiang Gong,[b] Mihails Arhangelskis,[c] Hatem M. Titi,[a] D. Scott Bohle,[a] Vinko Nemec,[d] Levente Kiss,[e] Andrew J. Morris,[f] Cristian Silvestru,[e] Dominik Cinčić,*[d] and Tomislav Friščić*[a,b]

a) Department of Chemistry, McGill University, 801 Sherbrooke St. W. H3A 0B8, Montreal, Canada
b) School of Chemistry, University of Birmingham, Edgbaston, B15 2TT, United Kingdom
c) Faculty of Chemistry, University of Warsaw, 1 Pasteura St., 02-093, Warsaw, Poland
d) Department of Chemistry, Faculty of Science, University of Zagreb, HR-10000, Zagreb, Croatia
e) Department of Chemistry, Supramolecular Organic and Organometallic Chemistry Centre (SOOMCC), Faculty of Chemistry and Chemical Engineering, Babeş-Bolyai University, Cluj-Napoca 400028, Romania
f) School of Metallurgy and Materials, University of Birmingham, Edgbaston, B15 2TT, United Kingdom

Table of Contents

Methods **2**
Analytical methodology 2
Synthetic procedures 22
Results **24**
Sb(*p*-Tol)$_3$ characterization 24
Single crystal analysis 29
Hirshfeld surface analysis of Form I Sb(*p*-Tol)$_3$ 31
Hirshfeld surface analysis of Form II Sb(*p*-Tol)$_3$ 32
Interaction maps of Form I and Form II Sb(*p*-Tol)$_3$ 33
Variable-Temperature Powder X-Ray Diffraction (VTPXRD) 34
Magic Angle Spinning Solid-State Nuclear Magnetic Resonance (MAS SSNMR) 36
Solid-state infrared spectroscopy 42
High-resolution Mass Spectrometry 43
Thermal Analysis 46
Before and after PXRDs of DSC samples 86
Before and after PXRDs of slurry experiments 91
Cambridge Structural Database structures 93
References **98**

Methods

Materials
All reagents used (antimony trichloride, *ReagentPlus*® 99%, magnesium turnings 98%) were ordered from Sigma Aldrich and used without further purification, with the exception of 4-bromotoluene, which was distilled under ~10 mmHg (or water aspirator) vacuum at 50 ˚C. All glassware used during synthesis was dried in a vacuum oven set to 125 ˚C overnight and used with exclusively SPS- dried solvents. Apiezon H grease and Sigma Aldrich silicone oil (CAS number 63148-62-9) were used during syntheses to seal glass joints and in oil baths respectively, and as additives for selected thermal cycling experiments.

Solution nuclear magnetic resonance (NMR) spectroscopy
Spectra were collected on: **1.** A Varian VNMRS 500 MHz spectrometer operating at 499.8 MHz (^{1}H) or 125.68 (^{13}C) (Figure S12, S16); **2.** A Varian Mercury 400 MHz spectrometer operating at 400.12 MHz (Figure S13, S14); **3.** A Bruker AVIII 500HD 500 MHz spectrometer operating at 500.30 MHz (Figure S15); **4.** NMR spectra for the samples originating from Babeș-Bolyai University, Romania, were measured on a Bruker Avance III 400 MHz spectrometer. For instruments **1**-**3**, the pulse program used was a standard 2-pulse (s2pul) with decoupling (^{1}H{^{13}C}, ^{13}C{^{1}H}).

Magic-angle spinning solid-state nuclear magnetic resonance (MAS-SSNMR) spectroscopy
^{13}C SSNMR spectra were collected on a Varian VNMRS 400 MHz spectrometer operating at 100.53 Hz, with 5 kHz sample spinning and a 50-millisecond acquisition time. Pulse program used was total antiphase non-CH cross polarization with total sideband suppression (TANCPXTOSS). Form II SSNMR samples universally had small traces of Form I present.

Differential scanning calorimetry (DSC)
DSC measurements were taken with a TA Instruments DSC 2500 (McGill University) or DSC 25 Instrument (University of Zagreb) in hermetically sealed aluminum pans.

Fourier-transform infrared attenuated total reflectance (FTIR-ATR) spectra
FTIR-ATR measurements were acquired on a Bruker Vertex 70 IR microscope instrument fitted with a Bruker Platinum ATR single-reflection diamond cell modular accessory with 32 scans and 16 background scans.

Single-crystal X-ray diffraction
The X-ray data for Form I and Form II were collected on a Bruker D8 Venture dual-source diffractometer equipped with a PHOTON II detector and an Oxford Cryostream 800 cooling system, using mirror-monochromated Cu*K*α radiation (λ = 1.54184 Å) from a microfocus source. Data was collected in a series of φ- and ω-scans. APEX3 software was used for data collection, integration and reduction.[1] Numerical absorption corrections were applied using SADABS-2016/2.[2]

Structures were solved by dual-space iterative methods using SHELXT[3] and refined by full-matrix least-squares on F^2 using all data with SHELXL[4] within the OLEX2[5] and/or WinGX[6] environment. For both structure models, some reflections were found to have been obscured by the beam stop and were omitted from the refinement. Hydrogen atoms were placed in calculated positions and treated as riding on the parent carbon atoms with isotropic displacement parameters 1.2 times (or 1.5 times in case of methyl groups) larger than the respective parent atoms. Extinction correction was applied to the structure of Form I. Crystal structure figures were generated using Mercury[7] and POV-Ray.[8]
CCDC 2175192 and 2175193 contain the supplementary crystallographic data for this paper. The data can be obtained free of charge from The Cambridge Crystallographic Data Centre via www.ccdc.cam.ac.uk/structures.

Cambridge Structural Database (CSD) search
The CSD search was done using Conquest Version 2026.1.0 and the CSD version 6.01 (Nov 2025) +1 update, for two fragments shown in Scheme S1, recording the Sb···Sb distances and X-Sb···Sb angles (green) for intermolecular contacts, as well as for intramolecular contacts in which antimony centers are separated by five or more bonds. The search was conducted both by each Sb atom set to be involved in three single bonds (Scheme S1a), as well as with only one defined single bond per Sb center (Scheme S1b). All searches were performed with exclusion of polymers.

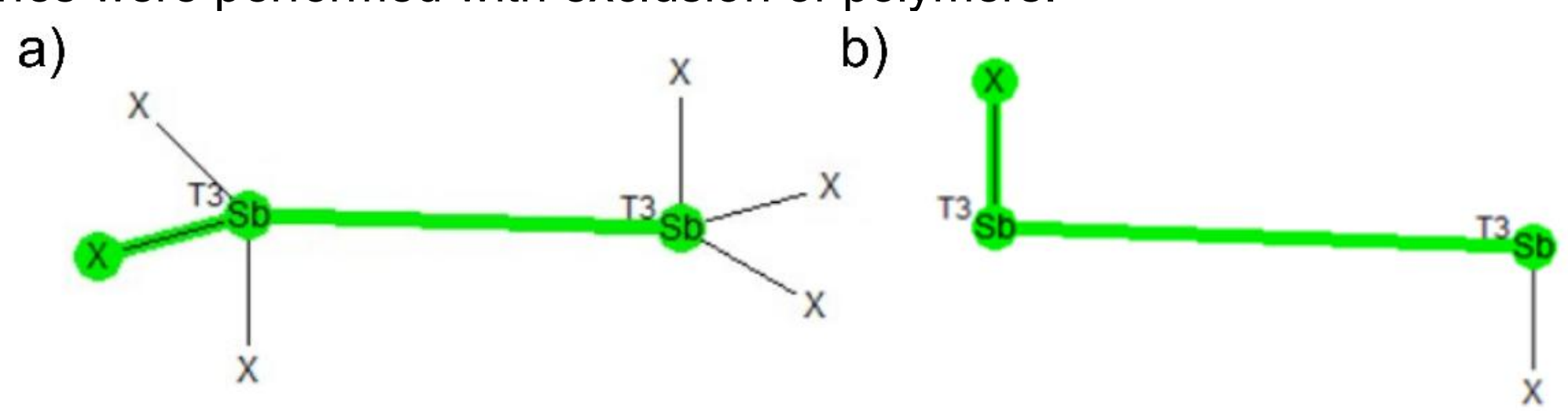

Scheme S1. Molecular fragments (X = any atom) used in the CSD searches.

The first search (Scheme S1a) yielded a total of 527 structures and, in cases where more than one structure was reported for a given chemical species, the entries were visually compared and, in case of structure redeterminations, only the first structure with 3-D coordinates was considered for analysis (excluded CSD codes: BOJFAH01, BOJFAH02, BOJFAH03, CEPDAC01, CEPDAC02, CIBKAZ01, CUDHOY01, DIKWOJ01, FOFVAX01, FOFVAX02, GALMIT01, GALMIT02, QESYIA01, TIWXOP01 to TIWXOP47 (47 entries), TPHDSB01, VENCOG01, WEKDUN01, YUDHIS01, ZAQRUE01, ZZZEHA03, ZZZEHA06, ZZZEHA08). Despite the search being conducted with exclusion of polymers, some of the entries were found to be polymeric, and were also excluded (CSD codes UCAGUD, UCAHAK, UCAGOX), as were structures based on very large clusters (CSD codes DUGFAQ, YIJFAC). Another, less restrictive search (Scheme S1b) along with visual inspection of the results indicated six further structures should be considered (CSD codes GOZCED, ISOYIY, NIBDUX, PANCUD, QAVJIG, SAYVIB). Out of these, the entry NIBDUX was excluded due to Sb atom being a part of a coordinated heterocycle. Overall, 74 entries were excluded, leading to a total of 459 analyzed entries.

Table S1. Crystallographic data for the crystal structures presented in this work.

Polymorph	Form I	Form II
CCDC Number	2175193	2175192
Formula	$C_{21}H_{21}Sb$	$C_{21}H_{21}Sb$
M_r	395.13	395.13
T (K)	298 (2)	253.0 (1)
Crystal system	trigonal	monoclinic
Space group	$R\overline{3}$	$P2_1/c$
a (Å)	12.8233(2)	11.7532(4)
b (Å)	12.8233(2)	6.4245(2)
c (Å)	20.1811(4)	24.6837(9)
α (°)	90	90
β (°)	90	97.896(2)
γ (°)	120	90
V (Å^3)	2873.92	1846.16
Z	6	4
ρ_{calc} (g cm^{-3})	1.370	1.422
μ (mm^{-1})	11.358	11.787
F(000)	1188	792
Crystal size (mm^3)	0.24 x 0.23 x 0.15	1.091 x 0.084 x 0.036
θ range for data collection (°)	19.122 – 144.816	9.744 – 144.864
Reflections collected	13429	10992
Independent reflections [R_{int}]	1244	3548
Data completeness (%)	97.7	97.2
Data/restraints/parameters	1244/0/69	3548/0/202
Goodness-of-fit on F^2	1.102	1.084
Final R for data with $I > 2\sigma(I)$	R_1 = 0.0393, wR_2 = 0.0886	R_1 = 0.0286, wR_2 = 0.0611
Final R for all data	R_1 = 0.0439, wR_2 = 0.0939	R_1 = 0.0365, wR_2 = 0.0665
Largest diff. peak/hole (e Å^{-3})	0.55/−0.70	0.60/−0.47
Extinction coefficient	0.0018 (3)	not modelled

Powder X-ray diffraction (PXRD)

PXRD patterns were collected from 4 to 40˚ 2θ, 0.5 seconds exposure with an interval of 0.05 2θ, using a Bruker D2 PHASER X-Ray diffractometer using a CuK_α (λ = 1.54 Å) radiation source, LYNXEYE detector and a Ni filter.

Variable temperature PXRD (VT-PXRD)

Variable temperature powder X-ray diffraction (VT-PXRD) experiments were performed on a Bruker D8 Advance diffractometer equipped with Anton Paar CHC plus+ chamber. The Bruker D8 Advance is also equipped with Ni-filtered CuK_α radiation, equipped with a LYNXEYE-XE linear position sensitive detector (Bruker AXS, Madison, WI). Diffractograms were collected in a stepwise fashion in a dry environment. After each collection (*ca.* 6 min), the temperature in the chamber was raised by 1 °C at a rate of 30 °C min^{-1} and a new collection started, in the range of 25 °C to 95 °C.

Rietveld refinement

Sequential Rietveld refinement[9] of the PXRD patterns collected during the VT-PXRD measurements was performed using TOPAS Academic v7.[10] The background was modelled with 8th order Chebyshev polynomial function, and peak shapes were described with a pseudo-Voigt function, combined with simple axial divergence model. The unit cell parameters of both polymorph phases were refined in each sequential step, in order to account for the shifting of diffraction peaks due to unit cell temperature expansion. Finally, the scale factors corresponding to the structures of both polymorphs were refined in order to account for the changes in the phase composition as a function of temperature (see also main text Figure 3, and SI Figure S25).

Hirshfeld surfaces and intermolecular interactions

Hirshfeld surfaces (HS), fingerprint plots, electrostatic surface potentials on the HS, and interaction energies between neighboring molecules were calculated using CrystalExplorer 17.[11,12] Input files were prepared in CrystalExplorer 17.[11,12] Wavefunctions were calculated using density-functional theory (DFT) with the STO-3G[13,14] basis set, and B3LYP[15-18] method to model exchange potential, running on an object-oriented system, Tonto.[19] Interaction energy calculations use Grimme's D2 dispersion correction.[20] Images of the calculated surfaces and interaction dimers were rendered in CrystalExplorer 17.[11,12] Hirshfeld surfaces are quantified with the parameter d_{norm}, (Equation S1). Terms which define d_{norm}, d_{e} and d_{i}, are defined as the distance from a given point on the HS to the closest atom exterior to the HS in the case of d_{e}, or interior to the HS in the case of d_{i}. These are first normalized relative to the sum of van der Waals radii (Equation S2).

Equation S1 $$\boldsymbol{d_{norm}} = \boldsymbol{d_{|e|}} + \boldsymbol{d_{|i|}}$$

Equation S2 $$\boldsymbol{d_{|e|}} = \frac{\boldsymbol{d_e} - \boldsymbol{r_{VdW}}}{\boldsymbol{r_{VdW}}};\ \boldsymbol{d_{|i|}} = \frac{\boldsymbol{d_i} - \boldsymbol{r_{VdW}}}{\boldsymbol{r_{VdW}}}$$

Periodic DFT calculations

Periodic DFT calculations were performed on structures of the herein reported Form I, Form II, and the originally reported Form I, found in the Cambridge Structural Database[21] (CSD) with reference code: TPTOSB01,[22] using the plane-wave code CASTEP (v25.1.2).[23] Input files were generated using the cif2cell program.[24] Structures containing R- symmetry were converted to the associated primitive setting to minimize the volume of the simulation cell, and therefore the number of atoms, to maximize the efficiency of the calculations. Converted structures are completely equivalent to those in the conventional space group settings. Core regions of electron density were described by On-The-Fly ultrasoft pseudopotentials from the CASTEP library, with the plane wave cut-off energy truncated to 1200 eV. The first Brillouin zone was sampled using regularly spaced Monkhorst-Pack[25] grids with *k*-point spacings of 0.07 2π $Å^{-1}$. The exchange-correlation term was described using the Perdew-Burke-Ernzerhof (PBE) functional.[26] Three different semi-empirical dispersion correction (SEDC) schemes were evaluated, namely: Grimme-D3(0),[27] Grimme-D3(BJ),[28] and Grimme-D4.[29-31] Results from the dispersion-corrected calculations were benchmarked against calculations using the local density approximation (LDA) description of exchange-correlation,[32] and PBE without dispersion corrections applied. Structures underwent variable cell optimizations with the following convergence criteria: self-consistent field calculation, electronic energy change = 1×10^{-10} eV•$atom^{-1}$; energy = 1×10^{-6} eV; force = 1×10^{-2} eV•$Å^{-1;}$ stress = 2×10^{-2} GPa; and displacement 5×10^{-4} Å.

Evaluation of dispersion corrections

To determine the most appropriate dispersion correction for this system, the change in cell parameters (*a*, *b*, *c*, *α*, *β*, *γ*) and change to the cell volume (*V*) were analysed for each polymorphic form, Tables S2-S4, Figure S1. Additionally, the relative stability of each polymorph was evaluated by calculating the cohesive energy ($E_{cohesive}$) for each form (Tables S5-9, Figures S2-3). The cohesive energy was calculated as;

Equation S3 $$E_{cohesive} = \frac{E_{bulk} - (N \cdot E_{iso})}{N}$$

Where E_{bulk} is the energy of the optimized crystalline bulk system, *N* is the number of Sb(*p*-Tol)$_3$ molecules in the bulk system and E_{iso} is the energy of an isolated molecule of Sb(*p*-Tol)$_3$ optimized in the gas phase. To obtain E_{iso}, a single molecule of Sb(*p*-Tol)$_3$ underwent a fixed cell optimization in a cubic cell of side length of 30 Å, following the same convergence criteria as used for the bulk system optimizations.

Table S2. Comparison of cell parameters for herein re-determined Sb(p-Tol)$_3$ Form I structure before and after DFT optimization with PBE, PBE-D3(0), PBE-D3(BJ), PBE-D4, and LDA methods. Values in parentheses denote the percentage change from the experimental cell.

Unit cell paramete r	Exp	DFT optimized				
		**PBE**	**PBE D3(0)**	**PBE D3(BJ)**	**PBE D4**	**LDA**
a / Å	10.0033	10.5575 (+5.74 %)	9.8229 (−1.80 %)	9.7531 (−2.50 %)	9.7966 (−2.07 %)	9.4960 (−5.07 %)
b / Å	10.0033	10.5575 (+5.74 %)	9.8229 (−1.80 %)	9.7531 (−2.50 %)	9.7966 (−2.07 %)	9.4960 (−5.07 %)
c / Å	10.0033	10.5575 (+5.74 %)	9.8229 (−1.80 %)	9.7531 (−2.50 %)	9.7966 (−2.07 %)	9.4960 (−5.07 %)
α / °	79.7300	78.7842 (−1.19%)	80.7808 (+1.32%)	80.7325 (+1.26 %)	80.6013 (+1.09 %)	80.5208 (+0.99 %)
β / °	79.7300	78.7842 (−1.19%)	80.7808 (+1.32 %)	80.7325 (+1.26 %)	80.6013 (+1.09 %)	80.5208 (+0.99 %)
γ / °	79.7300	78.7842 (−1.19%)	80.7808 (+1.32 %)	80.7325 (+1.26 %)	80.6013 (+1.09 %)	80.5208 (+0.99 %)
V / Å^3	957.9700	1123.4734 (+17.28 %)	914.6226 (−4.52 %)	894.9346 (−6.58 %)	906.0674 (−5.42 %)	824.6889 (−13.91 %)

Table S3. Comparison of cell parameters for Sb(p-Tol)$_3$ Form II before and after DFT optimization with PBE, PBE-D3(0), PBE-D3(BJ), PBE-D4, and LDA methods. Values in parentheses denote the percentage change from the experimental cell.

Unit cell Paramete r	Exp	DFT optimized				
		**PBE**	**PBE D3(0)**	**PBE D3(BJ)**	**PBE D4**	**LDA**
a / Å	11.7532	12.7171 (+8.20 %)	11.5616 (−1.63 %)	11.4009 (−3.00 %)	11.4878 (−2.26 %)	10.9038 (−7.23 %)
b / Å	6.4245	7.0252 (+9.35 %)	6.3310 (−1.45 %)	6.1843 (−3.74 %)	6.2466 (−2.77 %)	5.9475 (−7.43 %)
c / Å	24.6837	25.7257 (+4.22 %)	24.5911 (−0.38 %)	24.7989 (+0.47 %)	24.6808 (−0.01 %)	24.6918 (+0.03 %)
α / °	90.0000	90.0000 (--)	90.0000 (--)	90.0000 (--)	90.0000 (--)	90.0000 (--)
β / °	97.9000	97.6576 (−0.25%)	97.8850 (−0.02 %)	96.9018 (−1.02 %)	97.1050 (−0.81 %)	96.7662 (−1.16 %)
γ / °	90.0000	90.0000 (--)	90.0000 (--)	90.0000 (--)	90.0000 (--)	90.0000 (--)
V / Å^3	1846.160 0	2277.8411 (+23.38 %)	1782.9741 (−3.42 %)	1735.8226 (−5.98 %)	1757.4780 (−4.80 %)	1590.1075 (−13.87 %)

Table S4. Comparison of cell parameters for Sb(p-Tol)$_3$ for the originally reported Form I structure (CCDC code TPTOSB01) before and after DFT optimization with PBE, PBE-D3(0), PBE-D3(BJ), PBE-D4, and LDA methods. Values in parentheses denote the percentage change from the experimental cell.

Unit cell parameter	Exp	DFT optimized				
		**PBE**	**PBE D3(0)**	**PBE D3(BJ)**	**PBE D4**	**LDA**
a / Å	9.8561	10.6495 (+8.05 %)	9.8191 (−0.38 %)	9.7462 (−1.11 %)	9.7866 (−0.71 %)	9.4932 (−3.68 %)
b / Å	9.8561	10.6495 (+8.05 %)	9.8191 (−0.38 %)	9.7462 (−1.11 %)	9.7866 (−0.71 %)	9.4932 (−3.68 %)
c / Å	9.8561	10.6495 (+8.05 %)	9.8191 (−0.38 %)	9.7462 (−1.11 %)	9.7866 (−0.71 %)	9.4932 (−3.68 %)
α / °	80.0400	77.1688 (−3.59 %)	81.0184 (+1.22%)	80.9804 (+1.17 %)	80.9906 (+1.19 %)	80.6438 (+0.75 %)
β / °	80.0400	77.1688 (−3.59 %)	81.0184 (+1.22%)	80.9804 (+1.17 %)	80.9906 (+1.19 %)	80.6438 (+0.75 %)
γ / °	80.0400	77.1688 (−3.59 %)	81.0184 (+1.22%)	80.9804 (+1.17 %)	80.9906 (+1.19 %)	80.6438 (+0.75 %)
V / Å^3	918.7550	1129.1094 (+22.91 %)	915.1761 (−0.38 %)	894.7048 (−2.61 %)	905.9208 (−1.39 %)	824.7377 (−10.22 %)

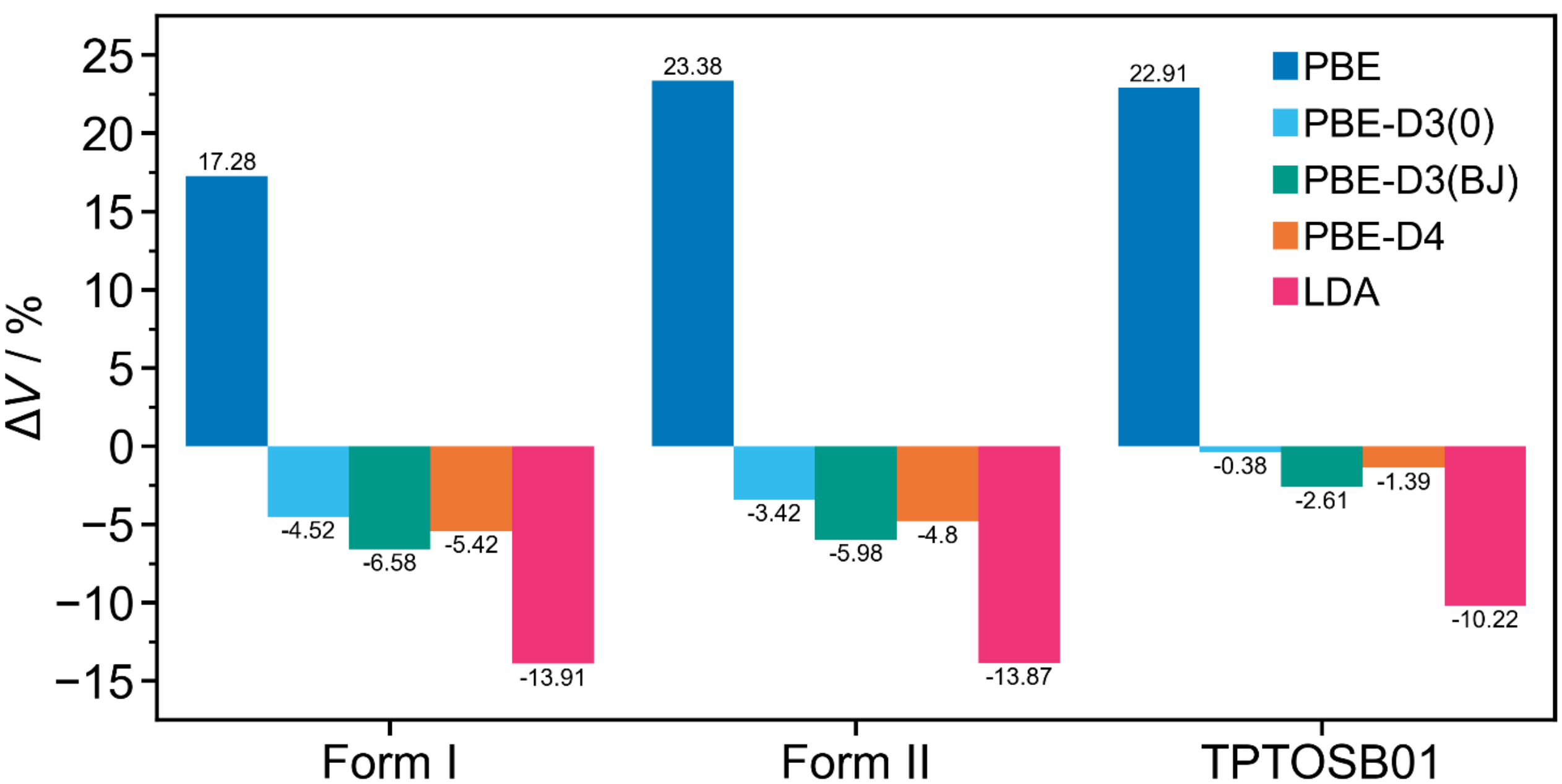

Figure S1. Comparison of the percentage change of cell volume (ΔV) for Sb(p-Tol)$_3$ for herein re-determined Form I, Form II, and originally reported Form I structure (CCDC code TPTOSB01) when using PBE (blue), PBE-D3(0) (cyan), PBE-D3(BJ) (green), PBE-D4 (orange), and LDA (magenta) methods.

Table S5. Cohesive energies for Sb(p-Tol)$_3$ for herein re-determined Form I, Form II, and originally reported Form I structure (CCDC code TPTOSB01) using PBE. E_{iso} was calculated to be −6048.133726 eV.

Structure	E_{bulk} / eV	N	N•E_{iso} / eV	$E_{cohesive}$ / eV	$E_{cohesive}$ / kJ•mol^{-1}
Form I	−12096.8697	2	−12095.2675	−0.3011	−29.05
Form II	−24193.4959	4	−24192.5349	−0.2402	−23.18
TPTOSB01	−12069.8614	2	−12095.2675	−0.2969	−28.65

Table S6. Cohesive energies for Sb(p-Tol)$_3$ for herein re-determined Form I, Form II, and originally reported Form I structure (CCDC code TPTOSB01) using PBE-D3(0). E_{iso} was calculated to be −6048.760862 eV.

Structure	E_{bulk} / eV	N	N•E_{iso} / eV	$E_{cohesive}$ / eV	$E_{cohesive}$ / kJ•mol^{-1}
Form I	−12100.8332	2	−12097.5217	−1.6557	−159.75
Form II	−24201.5749	4	−24195.0434	−1.6329	−157.55
TPTOSB01	−12100.8275	2	−12097.5217	−1.6529	−159.48

Table S7. Cohesive energies for Sb(p-Tol)$_3$ for herein re-determined Form I, Form II, and originally reported Form I structure (CCDC code TPTOSB01) using PBE-D3(BJ). E_{iso} was calculated to be −6049.758601 eV.

Structure	E_{bulk} / eV	N	N•E_{iso} / eV	$E_{cohesive}$ / eV	$E_{cohesive}$ / kJ•mol^{-1}
Form I	−12102.8018	2	−12099.5172	−1.6423	−158.46
Form II	−24205.6438	4	−24199.0344	−1.6524	−159.43
TPTOSB01	−12102.8004	2	−12099.5172	−1.6416	−158.39

Table S8. Cohesive energies for Sb(p-Tol)$_3$ for herein re-determined Form I, Form II, and originally reported Form I structure (CCDC code TPTOSB01) using PBE-D4. E_{iso} was calculated to be −6049.815833 eV.

Structure	E_{bulk} / eV	N	N•E_{iso} / eV	$E_{cohesive}$ / eV	$E_{cohesive}$ / kJ•mol^{-1}
Form I	−12102.6677	2	−12099.6317	−1.5180	−146.47
Form II	−24205.4107	4	−24199.2633	−1.5346	−148.07
TPTOSB01	−12102.6617	2	−12099.6317	−1.5150	−146.18

Table S9. Cohesive energies for Sb(*p*-Tol)$_3$ for herein re-determined Form I, Form II, and originally reported Form I structure (CCDC code TPTOSB01) using LDA. E_{iso} was calculated to be −6048.122565 eV.

Structure	E_{bulk} / eV	N	$N \cdot E_{iso}$ / eV	$E_{cohesive}$ / eV	$E_{cohesive}$ / kJ•mol^{-1}
Form I	−12099.0907	2	−12096.2451	−1.4228	−137.28
Form II	−24198.2387	4	−24192.4903	−1.4371	−138.66
TPTOSB01	−12099.0864	2	−12096.2451	−1.4206	−137.07

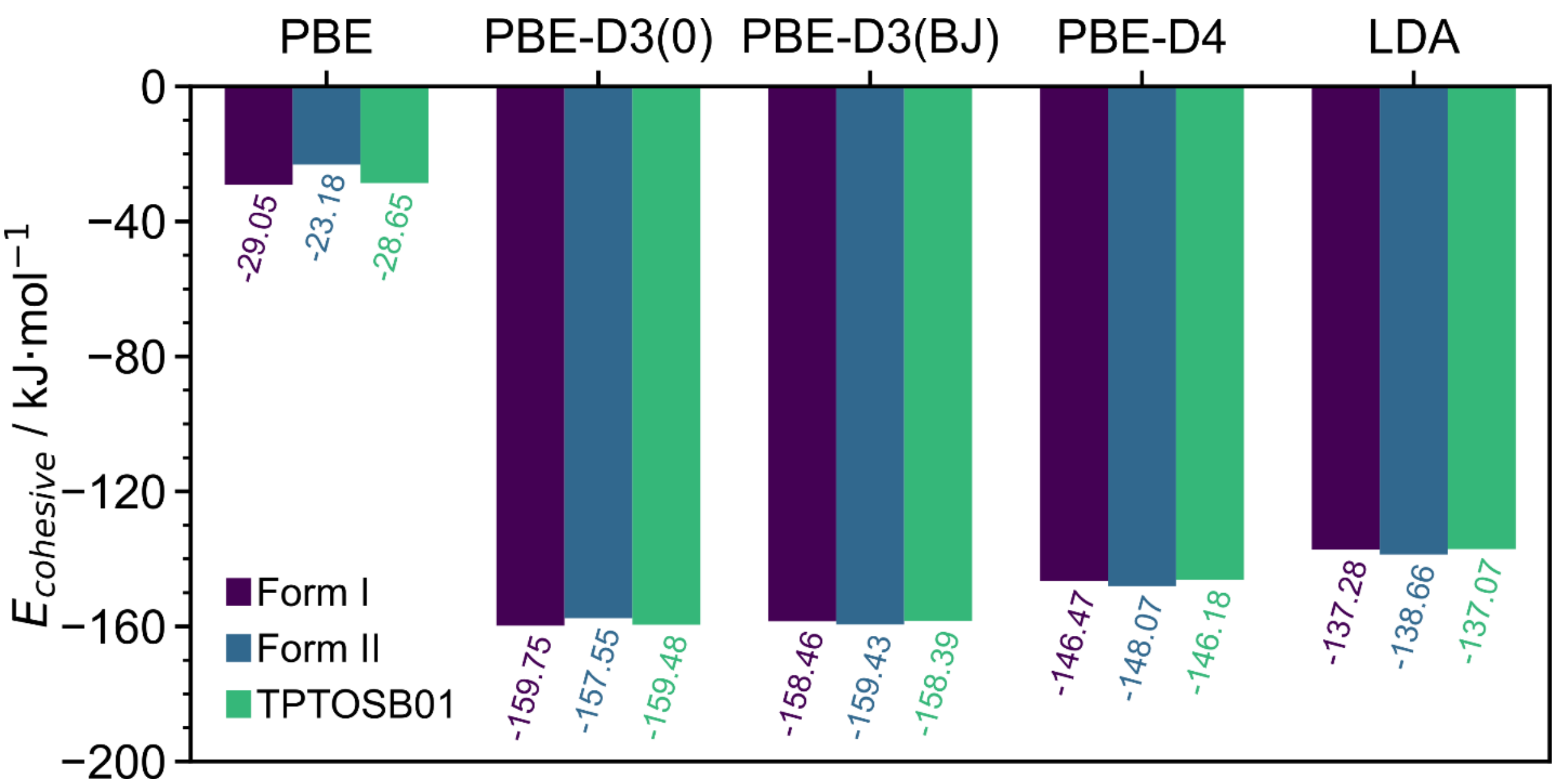

Figure S2. Comparison of the cohesive energies calculated for herein re-determined Sb(*p*-Tol)$_3$ Form I (purple), Form II (blue), and originally reported Form I structure (CCDC code TPTOSB01, green) when using PBE, PBE-D3(0), PBE-D3(BJ), PBE-D4, and LDA methods.

The formation of crystalline Sb(*p*-Tol)$_3$ is thermodynamically favorable, regardless of the dispersion correction used, or the resultant crystalline form. Using PBE without the implementation of an SEDC scheme, Form II is predicted to be less stable than Form I but the ca. 20 % expansion of the cell compared to experiment indicates that PBE alone is not sufficient to properly describe the system. When PBE-D3(BJ), PBE-D4, or LDA are used to account for dispersion interactions, Form II is predicted to be the most stable form of Sb(*p*-Tol)$_3$, which contrasts experimental observations. However, with D3(0) as the dispersion correction, Form II is predicted to be approximately 2.2 kJ•mol^{-1} less stable than Form I, in excellent agreement with the results of DSC analyses (see Differential Scanning Calorimetry (DSC) section).

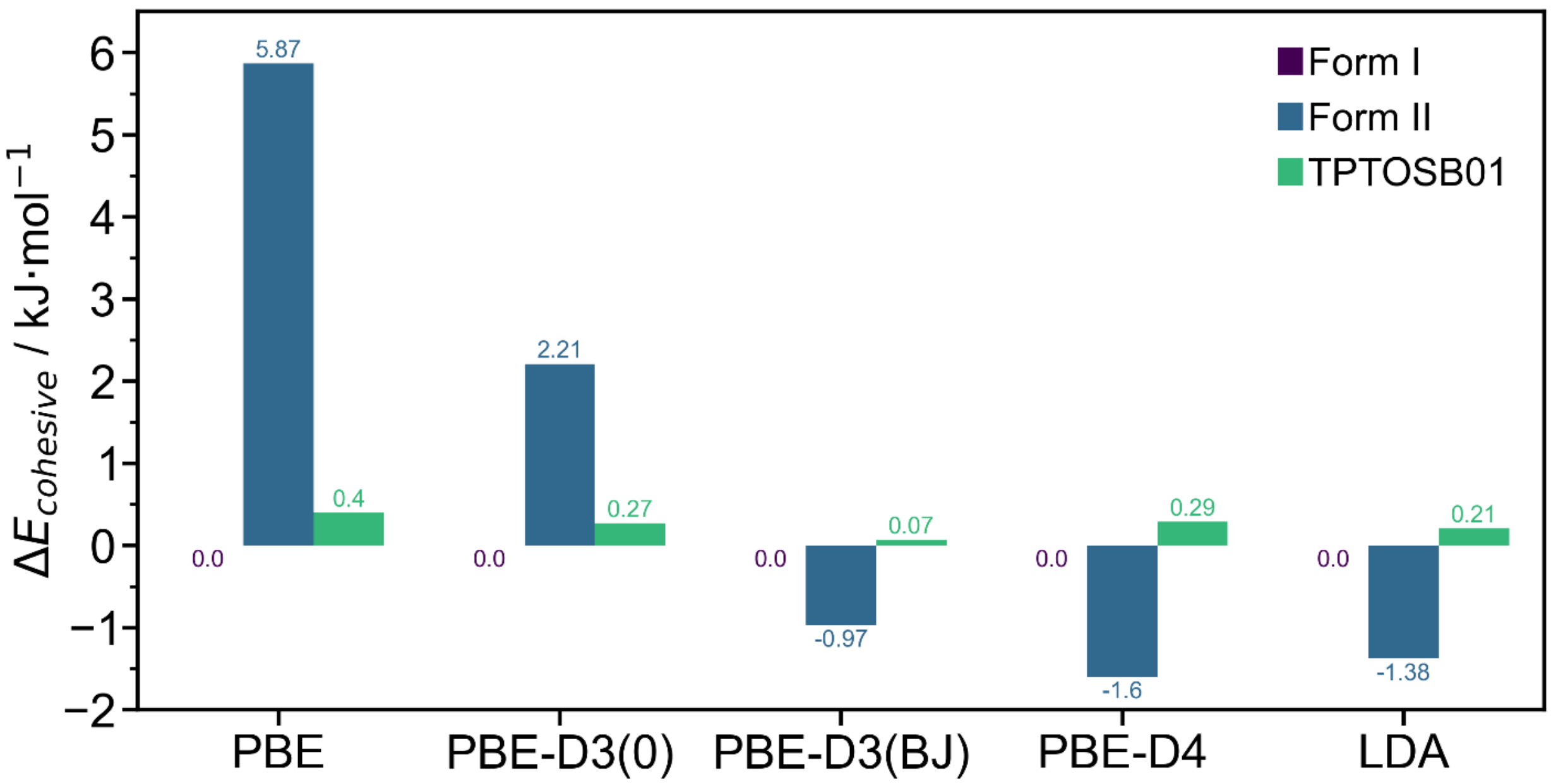

Figure S3. Comparison of the relative cohesive energy for herein re-determined Sb(*p*-Tol)$_3$ Form I (purple), Form II (blue), and originally reported Form I structure (CCDC code TPTOSB01, green) when using PBE, PBE-D3(0), PBE-D3(BJ), PBE-D4, and LDA methods. The relative cohesive energy is calculated with respect to the value obtained for the herein collected Form I structure.

Molecular dimer interaction energies were calculated with the D3(0) dispersion correction applied, given the agreement between experimental data and results of calculations with the D3(0) correction. The dimer interaction energies were calculated by placing the dimer fragments or individual molecules in a cubic simulation cell of 30 Å dimension. Molecular geometries from the DFT-optimized crystal structures were used in these calculations. Single-point energy calculations were then performed for the dimers, as well as individual molecules of Sb(*p*-Tol)$_3$, and dimer interaction energies were calculated as a difference between the electronic energy of the dimer and individual molecules (Table S10).

Table S10. Results of dimer interaction energy calculations.

Dimer motif	molecular energies / eV			Interaction energy / eV	Interaction energy / kJ•mol^{-1}
	Dimer	Molecule 1	Molecule 2		
Embrace (Form I)	−12098.3296	−6048.7507	−6048.7507	−0.8282	−79.91
Sb···Sb interaction (Form II)	−12098.7400	−6048.7347	−6048.7347	−0.2705	−26.10

The Form II dimer interaction energy was then evaluated with respect to the Sb···Sb distance ($r_{Sb\cdots Sb}$). To alter $r_{Sb\cdots Sb}$, the unit vector between the two Sb atoms was calculated and, subsequently, one of the molecules was translated along the given to the desired separation. The dimer interaction was evaluated using a series of single-point energy calculations, maintaining the PBE-D3(0) methodology, of the dimer configuration with $r_{Sb\text{-}Sb}$ in the range of 2.861 Å to 4.861 Å (*i.e.* ±1.0 Å) in steps of 0.1 Å, with respect to the optimized Sb···Sb distance (r_{Sb-Sb}^{opt}) of 3.861 Å (Figure S4a). After observing an initial energy well, $r_{Sb\text{-}Sb}$ values were increased from 4.861 Å to 8.261 Å, (i.e. up to r_{Sb-Sb}^{opt} +4.4 Å) in steps of 0.2 Å. The optimal Sb···Sb distance of the dimer in the gas phase is comparable to that of the bulk phase with only a minor increase (0.1 Å) observed. Additionally, the dimer interaction energy is found to be ca. 0.12 kJ•mol^{-1} more stable than observed at r_{Sb-Sb}^{opt}. We ascribe these differences to the lack of dispersion interactions between dimers that would be present in the bulk solid system.

To estimate the influence of dispersion interactions on the dimer interaction, we repeated the dimer interaction energy calculations using the exact same configurations but without the semi-empirical dispersion correction term (Figure S4b). Without the D3(0) correction applied, the optimal distance between the two Sb(*p*-Tol)$_3$ molecules increases by 0.5 Å to 4.361 Å, which would be expected given the 22 % volume expansion observed in the bulk system optimizations (Figure S1). An attractive energy well is still observed for the calculations without the dispersion corrections, albeit much shallower than observed with PBE-D3(0). At the preferred Sb···Sb distance the dimer interaction energy is approximately -5.44 kJ•mol^{-1}, which suggests that it is not solely dispersion interactions that hold the two Sb(*p*-Tol)$_3$ molecules together.

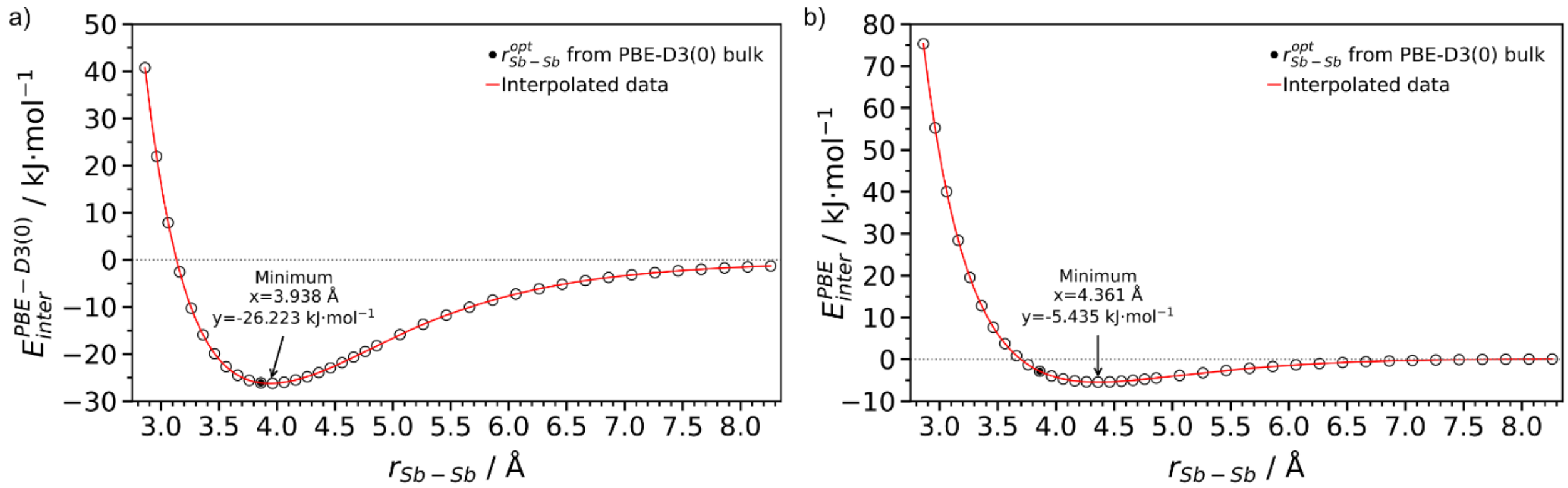

Figure S4. Interaction energy profiles of an Sb(p-Tol)$_3$ Form II dimer with respect to the PBE-D3(0) optimised structure (filled black dots) as the Sb···Sb distance ($r_{\text{Sb-Sb}}$) is changed. Interaction energies (E_{inter}, black rings) were obtained from single-point energy calculations using the a) PBE-D3(0) and b) PBE methods and were interpolated with a cubic splines approach (red lines).

Evaluating the dimer interaction energies at r_{Sb-Sb}^{opt} shows that when the dispersion correction is turned off, the magnitude of the attraction is reduced to ca. 3 kJ•mol^{-1}, which represents a ca. 90% reduction compared to the PBE-D3(0) method (Tables S10, S11).

Table S11. Results of Form II dimer interaction energy calculations at r_{Sb-Sb}^{opt} with the PBE method applied.

	molecular energies / eV			Interaction energy / eV	Interaction energy / kJ•mol^{-1}
	Dimer	Molecule 1	Molecule 2		
PBE	−12096.2293	−6048.0998	−6048.0998	−0.0298	−2.87

Effect of molecular size on Sb···Sb dimer interaction energy

Generally, the magnitude of dispersion interactions is accepted to increase with molecular size.[33] Therefore, we decided probe this effect by changing the ligands on the Sb atoms from p-tolyl groups to methyl groups. The crystal structure of $SbMe_3$ which was recently elucidated by Bujak *et al.* also exhibits short Sb···Sb contacts ranging from ca. 3.87 Å to ca. 3.84 Å, depending on temperature of the X-ray diffraction experiment.[34] To evaluate the Sb···Sb contact, the bulk $SbMe_3$ structure (CSD[21] refcode: GALMIT[34]) was optimized using CASTEP with the PBE-D3(0) method employed. The resulting crystal structure remained consistent, exhibiting a small (< 3%) contraction in cell volume (Table S12).

Table S12. Comparison of cell parameters for $SbMe_3$ for the originally reported structure (CSD code GALMIT)[34] before and after DFT optimization with PBE-D3(0).

Unit cell parameter	Exp	PBE D3(0)	Difference / %
a / Å	6.1203	6.0638	−0.92
b / Å	11.2342	11.1183	−1.03
c / Å	8.5536	8.4716	−0.96
α / °	90.0000	90.0000	--
β / °	96.8027	96.8712	+0.07
γ / °	90.0000	90.0000	--
V / Å^3	583.9729	567.0372	−2.90

From the optimized cell, r_{Sb-Sb}^{opt} was found to be 3.810 Å, within the combined van der Waals radii of two Sb atoms. The configuration of a dimer was extracted from the optimized cell and placed in a cubic box of side length equal to 30 Å to calculate the dimer interaction energy. Similar to the analysis performed for Sb(p-Tol)$_3$, the dimer interaction energy was calculated for the configuration extracted from the bulk crystal (Table S13), and across a range of Sb-Sb distances from 3.710 Å (i.e. r_{Sb-Sb}^{opt} - 1.0 Å) up to 8.210 Å (i.e. r_{Sb-Sb}^{opt} + 4.4 Å) using both PBE and PBE-D3(0) methods (Figure S5).

Table S13. Results of $SbMe_3$ dimer interaction energy calculations at r_{Sb-Sb}^{opt} with PBE-D3(0) and PBE methods applied.

Method	molecular energies / eV			Interaction energy / eV	Interaction energy / kJ•mol^{-1}
	Dimer	Molecule 1	Molecule 2		
PBE-D3(0)	−6037.5533	−3018.7094	−3018.7094	−0.1346	−12.98
PBE	−6037.3149	−3018.6286	−3018.6286	−0.0577	−5.57

Evaluation of the dimer interaction energies at r_{Sb-Sb}^{opt} reveals an attractive interaction of 12.98 kJ•mol^{-1} when the D3(0) dispersion correction is applied (Table S13). When calculated without the dispersion correction term the magnitude of the attractive interaction is reduced to 5.57 kJ•mol^{-1}. This suggests that dispersion interactions account for approximately 57 % of the interaction in the case of $SbMe_3$, which is consistent with previous calculations of dimer interactions for a series of small molecules pnictogen dimers.[35]

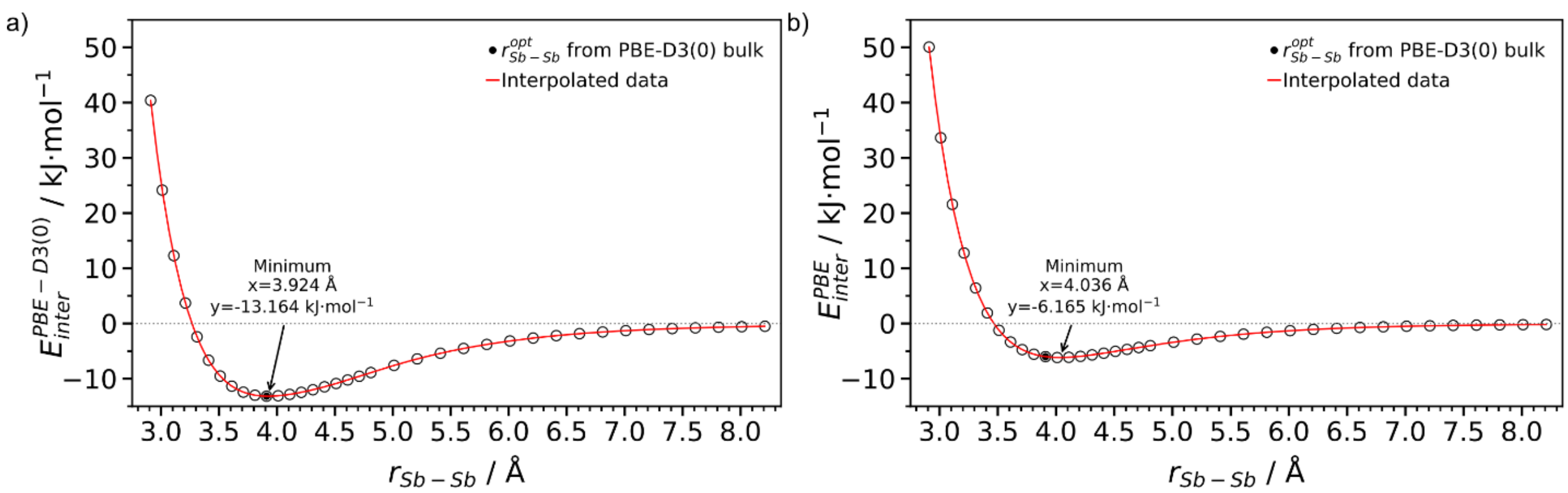

Figure S5. Interaction energy profiles of an $SbMe_3$ dimer with respect to the PBE-D3(0) optimised structure (filled black dots) as the Sb···Sb distance ($r_{Sb\text{-}Sb}$) is changed. Interaction energies (E_{inter}, black rings) were obtained from single-point energy calculations using the a) PBE-D3(0) and b) PBE methods and were interpolated with a cubic splines approach (red lines).

When evaluating the interaction energy with respect to $r_{Sb\text{-}Sb}$ for $SbMe_3$ dimers, similar observations can be made when comparing to Sb(*p*-Tol)$_3$ dimers. Specifically, when evaluated using PBE-D3(0), the optimal distance of the dimer in the phase is very similar to r^{opt}_{Sb-Sb}, with only a ca. 0.12 Å increase observed, which is accompanied by an increase in the interaction energy of ca. 0.2 kJ•mol^{-1} (Figure S5a). When calculated with PBE, i.e. without the D3(0) dispersion correction (Figure S5b), the optimal distance between the two $SbMe_3$ molecules increases by ca. 0.23 Å with an associated interaction energy of 6.17 kJ•mol^{-1}, approximately 0.60 kJ•mol^{-1} greater than the energy calculated at r^{opt}_{Sb-Sb}. Consistent with the results for Sb(*p*-Tol)$_3$, this analysis suggests dispersion interactions are not solely responsible for holding together dimers of $SbMe_3$.

Bond critical point (BCP) analysis

To gain a better understanding of the intermolecular interactions between different Sb(*p*-Tol)$_3$ molecules in the herein determined Forms I and II, the quantum theory of atoms in molecules (QTAIM) was employed to identify critical points based on the electron density topology of the crystal structure.[36] In particular, first-order saddle points (BCPs), as well as the associated bond paths were examined to characterize the intramolecular interactions between Sb(*p*-Tol)$_3$ molecules.[37-39] For Form I and Form II, the post-optimization electron densities computed by CASTEP, were stored in individual binary checkpoint (.check) files, and were converted into three-dimensional volume data (.cube) files using the C2x[40] code. The resultant cube files were then directly processed using the critic2[41,42] code for the postprocessing and identification of critical points within each crystal structure, in which the polyharmonic spline method (SMOOTHRHO) is employed to interpolate the electron density across a uniform grid in the structural space, enabling the calculation of the electron density derivatives.[43]

In the case of Form I, no BCPs were observed between the Sb atoms of two in the phenyl-embrace motif, which we ascribe to the large Sb-Sb distance between the two molecules within the phenyl-embrace motif. Instead, the BCPs that are highest in electron density (0.045 electrons·Å^{-3}) are observed from aromatic protons to aromatic carbon atoms of neighboring molecules, indicating that there are C-H···π interactions between two Sb(*p*-Tol)$_3$ molecules in the phenyl-embrace motif found in Form I (Figure S4a). In contrast, the BCPs of highest electron density (0.068 electrons·Å^{-3}) found in dimers of Sb(*p*-Tol)$_3$ in Form II are observed between Sb atoms of different molecules (Figure S4b).

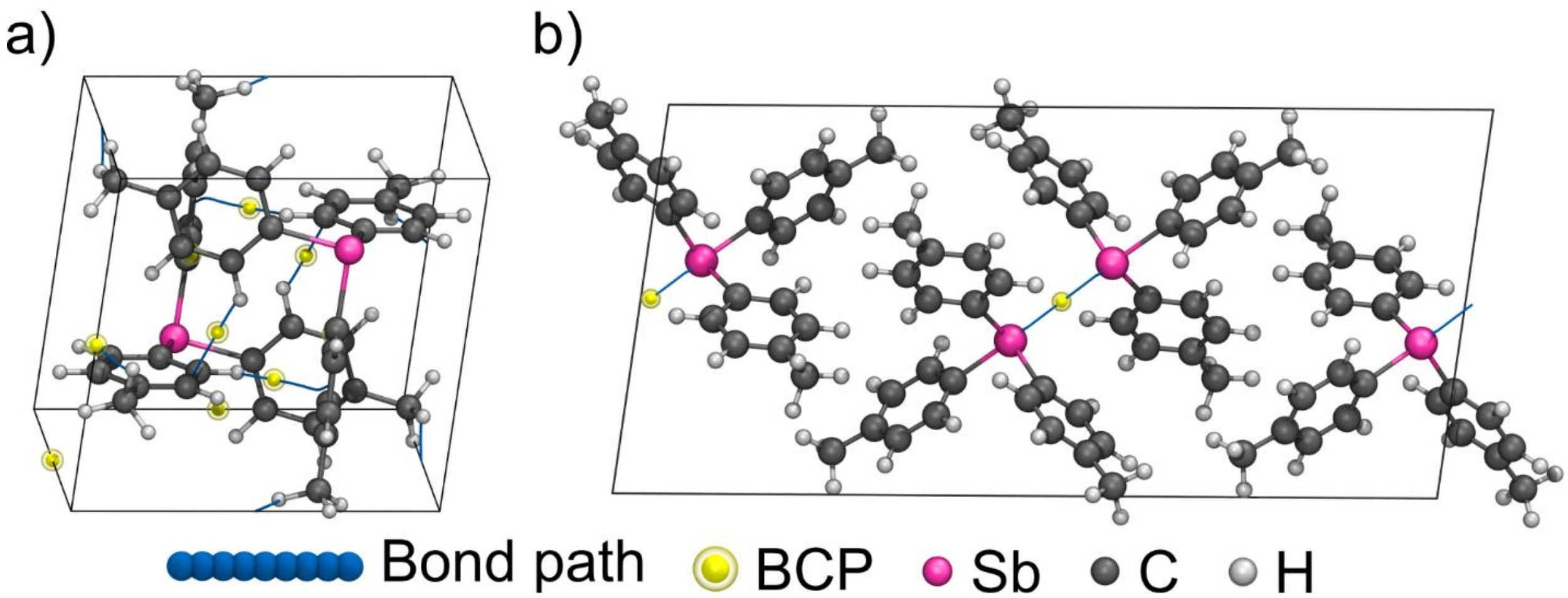

Figure S6. The highest electron density at bond critical points (BCPs) between different dimer units in Form I and II: a) the configuration of Form I shows BCPs from aromatic protons and aromatic carbons with an electron density of 0.045 electrons Å^{-3}; b) the configuration of Form II shows BCPs between Sb atoms with an electron density of 0.068 electrons Å^{-3}.

To further illustrate electron density distribution between antimony atoms, a plane defined by the two nearest neighbour Sb atoms and a carbon atom that is covalently bonded to Sb (Figures S5a and S6a) upon which the 2D electron density was projected for each Form I and Form II (Figures S5b and S6b, respectively).

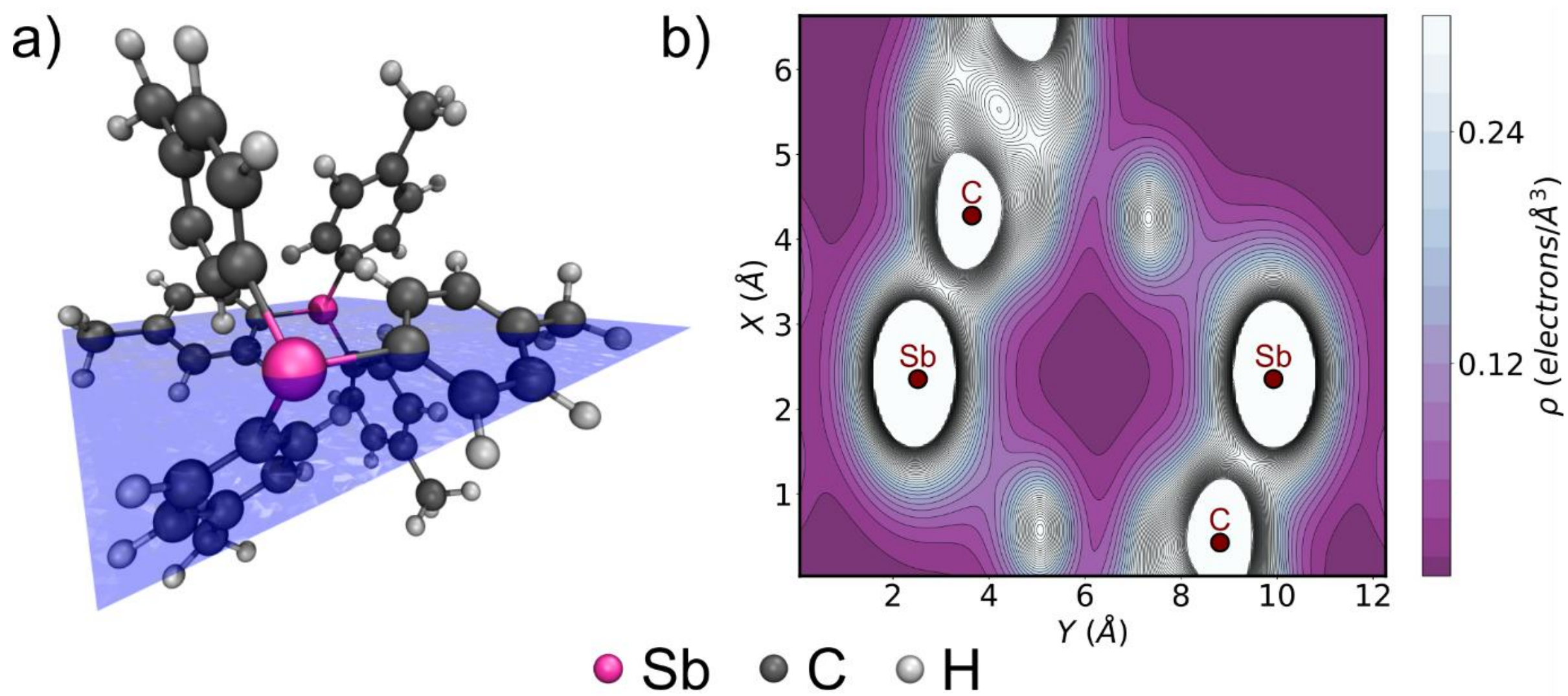

Figure S7. a) The configuration of Form I along with the plane used (blue) for electron density distribution analysis; b) a contour map showing the electron density distribution on that plane with zero electron density evident between Sb atoms.

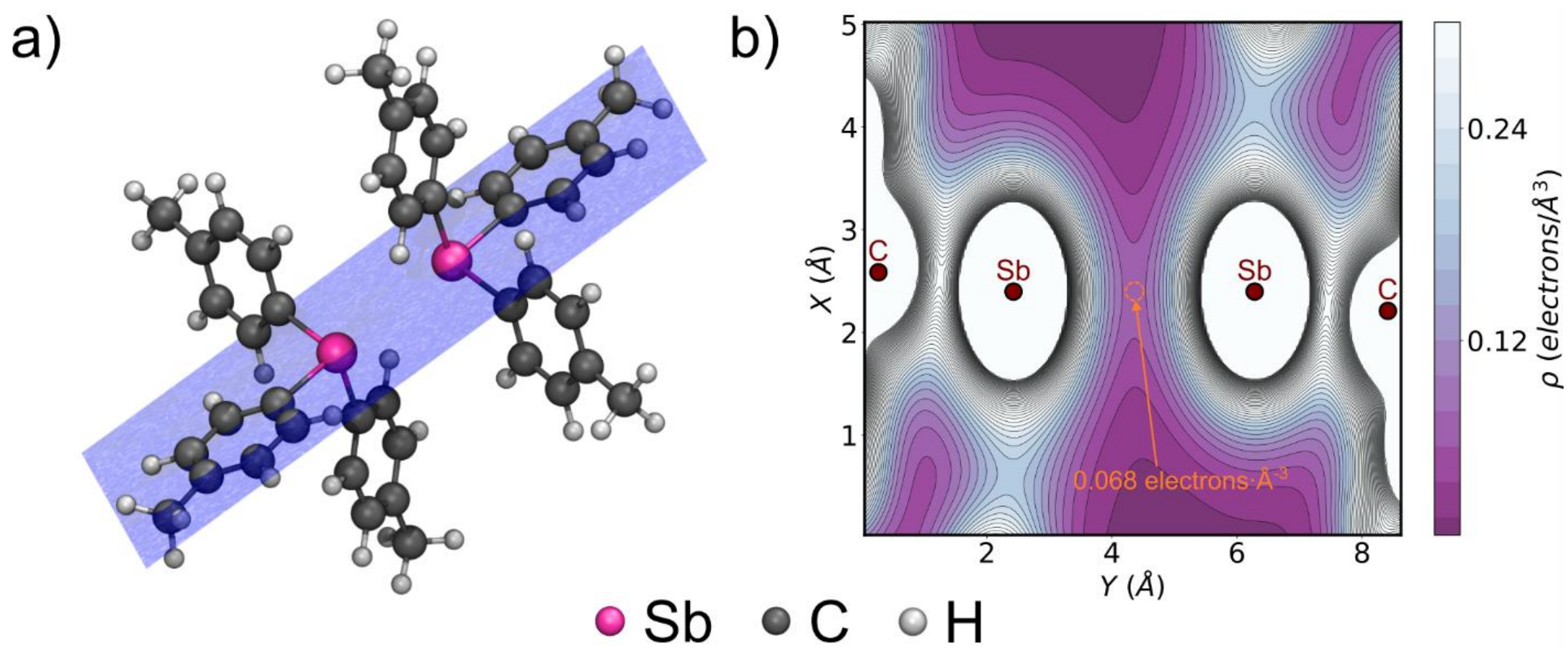

Figure S8. (a) The configuration of Form II along with the plane used (blue) for electron density distribution analysis; (b) a contour map showing the electron density distribution on that plane with the BCP between the two Sb atoms at 0.068 electrons·Å^{-3} highlighted by the orange dashed circle.

Molecular DFT calculations

Molecular DFT calculations were performed on individual molecules, as well as supramolecular Sb···Sb connected dimers, using geometries from Form II structure following DFT optimization using the PBE-D3(0) approach, using the ORCA v6.1.0 program.[44-46] All calculations employed the def2-TZVPP basis set[47,48] with an effective core potential applied for the Sb atoms.[49] Following the results from periodic DFT calculations the PBE functional[26] and D3(0) method[27] were applied to describe the exchange-correlation and semi-empirical dispersion terms, respectively. For self-consistent field calculations, the tolerance for electronic energy change was set to 1×10^{-10} E_h. For structure optimizations the following criteria defined convergence: energy = 5×10^{-6} E_h; force = 3×10^{-4} $E_h\bullet a_0^{-1}$; and displacement 3×10^{-5} a_0. To ensure that the Sb···Sb distance and relative orientation of the molecules in the dimer remained consistent with that obtained from periodic DFT calculations, the positions of the Sb atom and the three adjacent C atoms, covalently bonded to Sb, on each molecule were fixed. All other degrees of freedom were allowed to optimize freely.

Electrostatic surface potential (ESP) mapping

ESP maps were calculated for both the monomer and dimer configurations found in the Form II crystal structure, following geometry optimization and population analysis (Figure S9) and were visualized in VMD (v1.9.4a55)[50] at the 0.005 $e \bullet a_0^{-3}$ isosurface level which approximately corresponds to the solvent-excluded surface (or the Connolly surface).[51] ESP maps were also calculated for the monomers of the $SbMe_3$ and $SbCl_3$ following geometry optimization and population analysis, and were visualized in VMD at the 0.005 $e \bullet a_0^{-3}$ level (Figure S10).

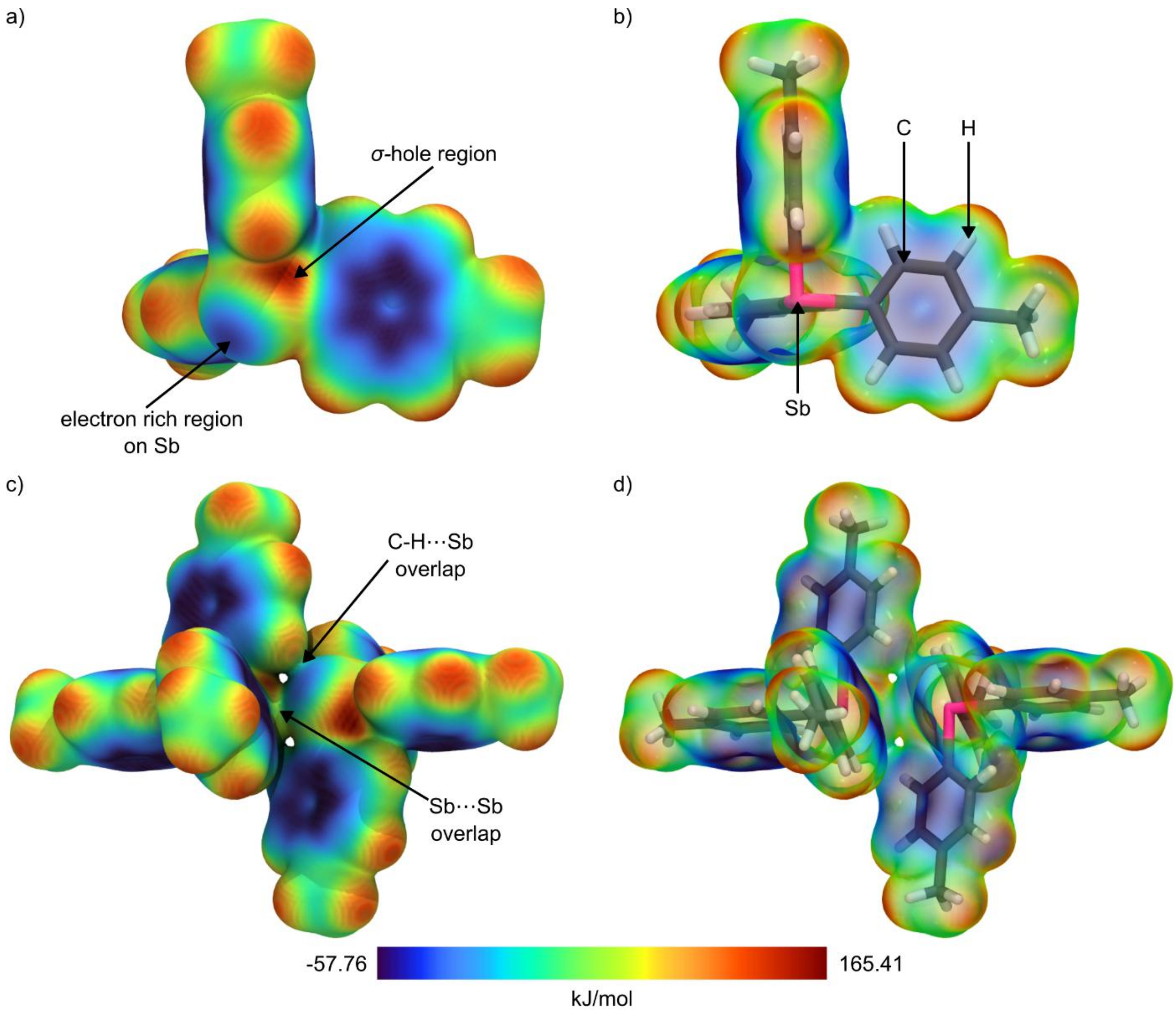

Figure S9. ESP maps for: a single molecule of Sb(*p*-Tol)$_3$ with a) an opaque surface which highlights both the electron-rich and *σ*-hole regions on the Sb atom and b) a translucent surface to show the atomic configuration; a dimer of Sb(*p*-Tol)$_3$ in configuration from Form II with c) an opaque surface which highlights ESP map overlaps which suggest CH···Sb and Sb···Sb contacts, and d) a translucent surface to show the arrangement of atoms. ESP maps are rendered at the 0.005 $e \bullet a_0^{-3}$ isosurface.

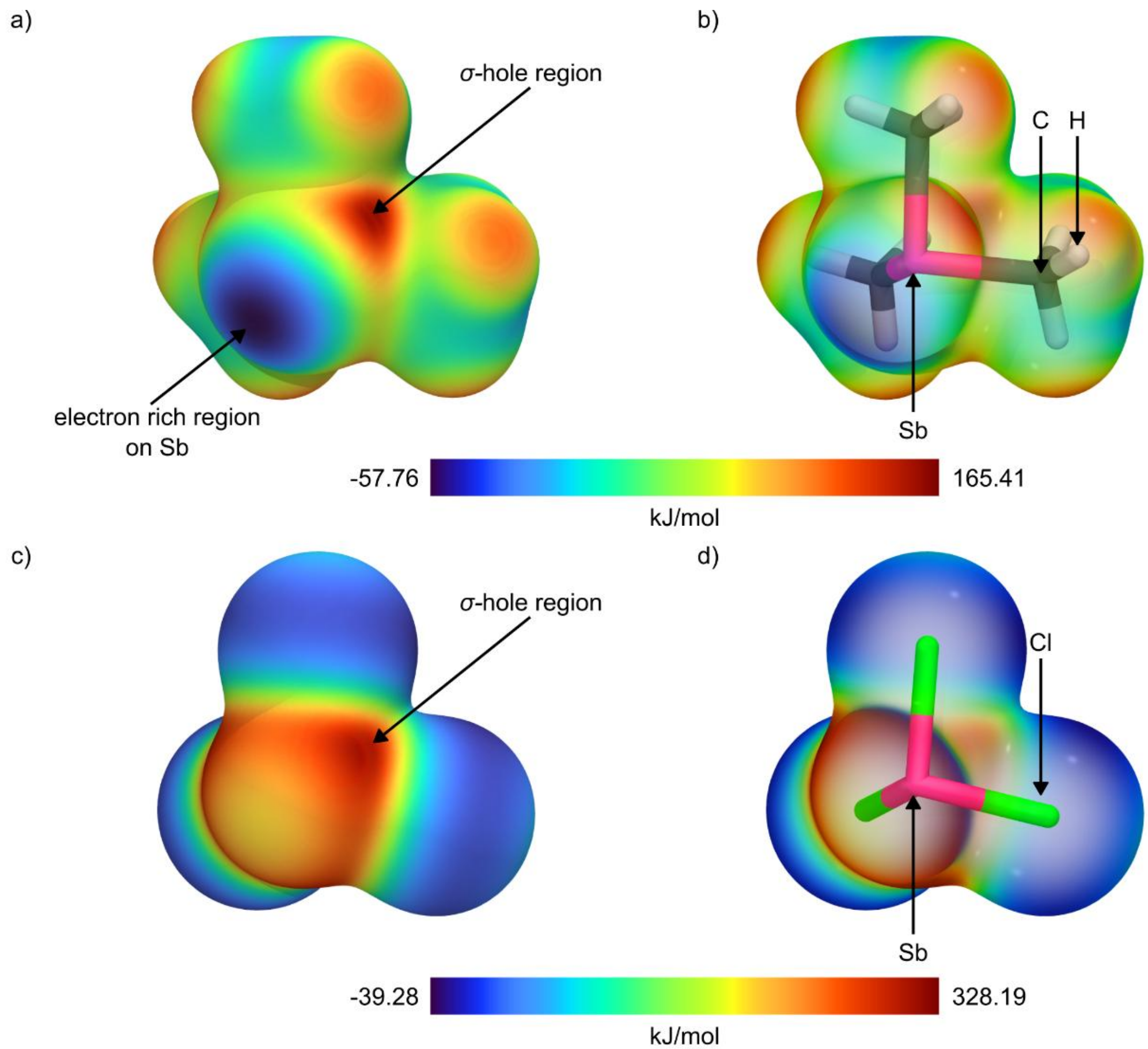

Figure S10. ESP maps for a single molecule of a) $SbMe_3$ with an opaque surface highlighting both the electron-rich and the σ-hole regions on the Sb atom, b) $SbMe_3$ with a translucent surface to illustrate the arrangement of atoms, c) $SbCl_3$ with an opaque surface which highlights the σ-hole region on the Sb atom, and d) $SbCl_3$ with a translucent surface to show arrangement of atoms. ESP maps are rendered at the 0.005 $e \bullet a_0^{-3}$ isosurface.

Density difference calculations

The electron density difference, $\Delta\rho(\mathbf{r})$, between the Sb(*p*-Tol)$_3$ dimer, in the Form II configuration, and the two individual molecules were calculated using equation S4, where all densities were determined using the geometry of the full system.

$$\textit{Equation S4} \quad \Delta\rho(\boldsymbol{r}) = \rho(\boldsymbol{r})_{dimer} - \rho(\boldsymbol{r})_{molecule_a} - \rho(\boldsymbol{r})_{molecule_b}$$

Density differences were rendered using VMD[50] at isosurface levels of $\pm 5\times10^{-4}$ $e\bullet a_0^{-3}$ (Figure S11), where positive regions (translucent red) and negative regions (translucent blue) indicate areas of electron accumulation and depletion, respectively.

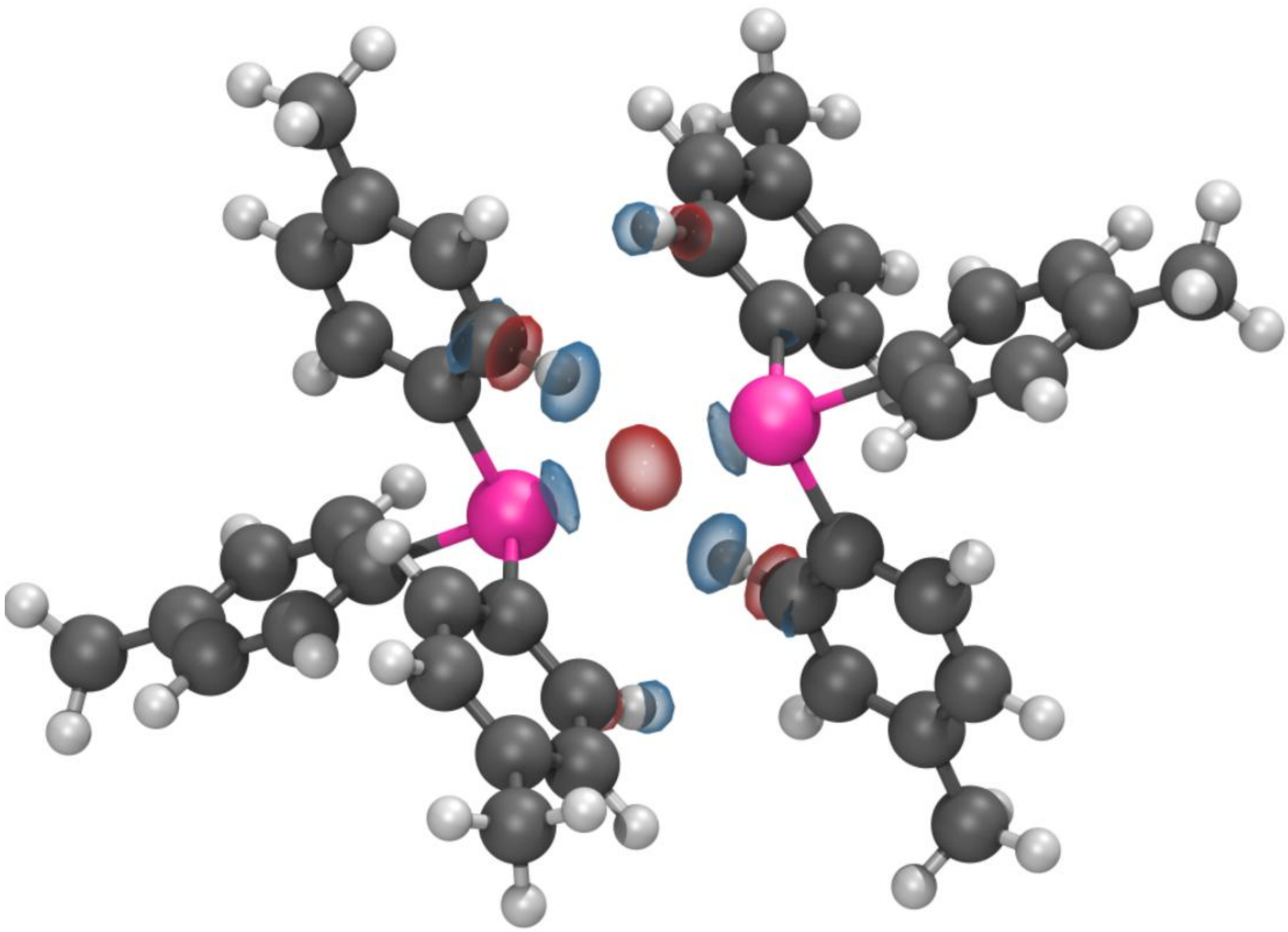

Figure S11. Render of electron density difference of Sb(*p*-Tol)$_3$ dimer from the Form II configuration with respect to the individual Sb(*p*-Tol)$_3$ molecules, where translucent red regions indicate an increase in electron density and translucent blue regions represent a decrease. Electron density regions are rendered at $\pm 5\times10^{-4}$ $e\bullet a_0^{-3}$ isosurfaces.

Synthetic Procedures

Synthesis of Sb(*p*-Tol)$_3$ (McGill University)

A three-neck, 250 mL round-bottom flask charged with 4-bromotoluene (5.196g, 30.4 mmol) and magnesium turnings (746 mg, 30.7 mmol) was evacuated and refilled three times with nitrogen. Next, ca. 100 mL of diethyl ether was added with constant stirring. The flask was then put under positive pressure of nitrogen for the addition of ca. 0.5 mg solid I_2 to activate the magnesium turnings, with cooling by immersion in a water bath. After thirty minutes, a slight colour change was observed. At this point a solution of $SbCl_3$ (2.310g, 30.4 mmol) in ca. 100 mL diethyl ether was added dropwise to the Grignard via a pressure-equalized addition funnel. The apparatus was allowed to warm the sample to room temperature following the addition of all the $SbCl_3$ solution and was allowed to stir for 18h. For workup, a 4 M aqueous NH_4Cl solution was used to quench any unreacted Grignard reagent, and the resulting biphasic mixture was filtered over cotton into a separation funnel for liquid-liquid extraction. The ether layer was rinsed three times with 4 M aqueous NH_4Cl, the ether layer dried over Na_2SO_4, filtered again over a fine frit loaded with celite and then evaporated using a rotary evaporator to produce a crystalline solid (3.211 g). Of this, 1.602 g was set aside (referred to as "crude Sb(*p*-Tol)$_3$"). The remaining material (1.605 g) was purified by dissolution in gently boiling isopropanol and placed in a −10 ˚C freezer, to produce colorless crystals that were dried *in vacuo* and represent the default, bulk material used in the study, referred to as "Sb(*p*-Tol)$_3$". A batch of this material, (ca. 400 mg) was also purified by sequential re-crystallization from EtOH and hexanes, producing colorless crystals that were rinsed with cold EtOH and cold hexanes over a frit, before being placed in a vacuum oven set to 80 ˚C overnight. The sample was then left to dry under dynamic vacuum in a vacuum desiccator charged with anhydrous P_4O_{10} for a week, and referred to as "re-crystallized Sb(*p*-Tol)$_3$". Repeated syntheses in which all material is re-crystallized once from EtOH (yielding samples designated "Sb(*p*-Tol)$_3$") have provided yields in the range 78-82%, after drying *in vacuo*.

^{1}H NMR: (Varian 400.12 MHz, 25˚C, C_6D_6) δ 7.50 – 7.44 (d, 6H, J^3_{HH}= 7.62Hz), 7.00 – 6.93 (d, 6H, J^3_{HH} = 7.46Hz), 2.05 (s, 9H) ppm.

^{13}C(^{1}H) NMR: (Varian 125.68 MHz, 25˚C, DCM-D_2,) δ 138.8, 136.4 135.3, 130.0, 21.4 ppm.

^{13}C(^{1}H) MAS SSNMR Form I: (Varian 400 MHz) δ 139.0, 137.1, 133.2, 130.0, 22.8 ppm.

^{13}C(^{1}H) MAS SSNMR Form II: (Varian 400 MHz) δ 138.9, 137.3, 135.2, 130.0, 22.8, 22.1, 21.4, 20.0 ppm.

Synthesis of Sb(*p*-Tol)$_3$ (Babeș-Bolyai University)

To a THF$_{anh}$ solution of the *p*-TolMgBr (12.315 g, 72 mmol, 20% excess in 50 ml) prepared from *p*-TolBr and Mg (2.100 g, 86.4 mmol, activated with 2.676 g 12.4 mmol, 1,2-$Br_2C_2H_4$) a THF solution of $SbCl_3$ (20 mmol, in 50 ml) is added dropwise at reflux, for 15 min., then the reflux is continued for 1 h. The obtained dark solution is stirred at r.t. for 3 days followed by quenching with 10 ml of water added dropwise. The mixture is diluted with the addition of 50 ml water and extracted with CH_2Cl_2 (4 x 25 ml). The combined organic phase is washed with water (2 x 25 ml), dried on anhydrous Na_2SO_4, filtered and the solvents are evaporated at reduced pressure. The colorless solid obtained is further dried in a rotary evaporator (40 °C, 5 mbar) for 3 hours, resulting in 7.12 g (90 % yield). M.p. 124-125 °C (120-123°C in Wada[52])

^{1}H NMR (Bruker, 400 MHz, $CDCl_3$): δ = 7.38 (d, 2H, $^3J_{HH}$ = 7.8 Hz), 7.18 (d, 2H, $^3J_{HH}$ = 7.5 Hz), 2.38 (s, 3H) ppm.

^{13}C(^{1}H) NMR (Bruker, 101 MHz, $CDCl_3$) δ = 138.42, 136.29, 134.94, 129.79, 21.52 ppm.

Results

^{1}H NMR of normal Sb(*p*-Tol)$_3$ in CDCl$_3$

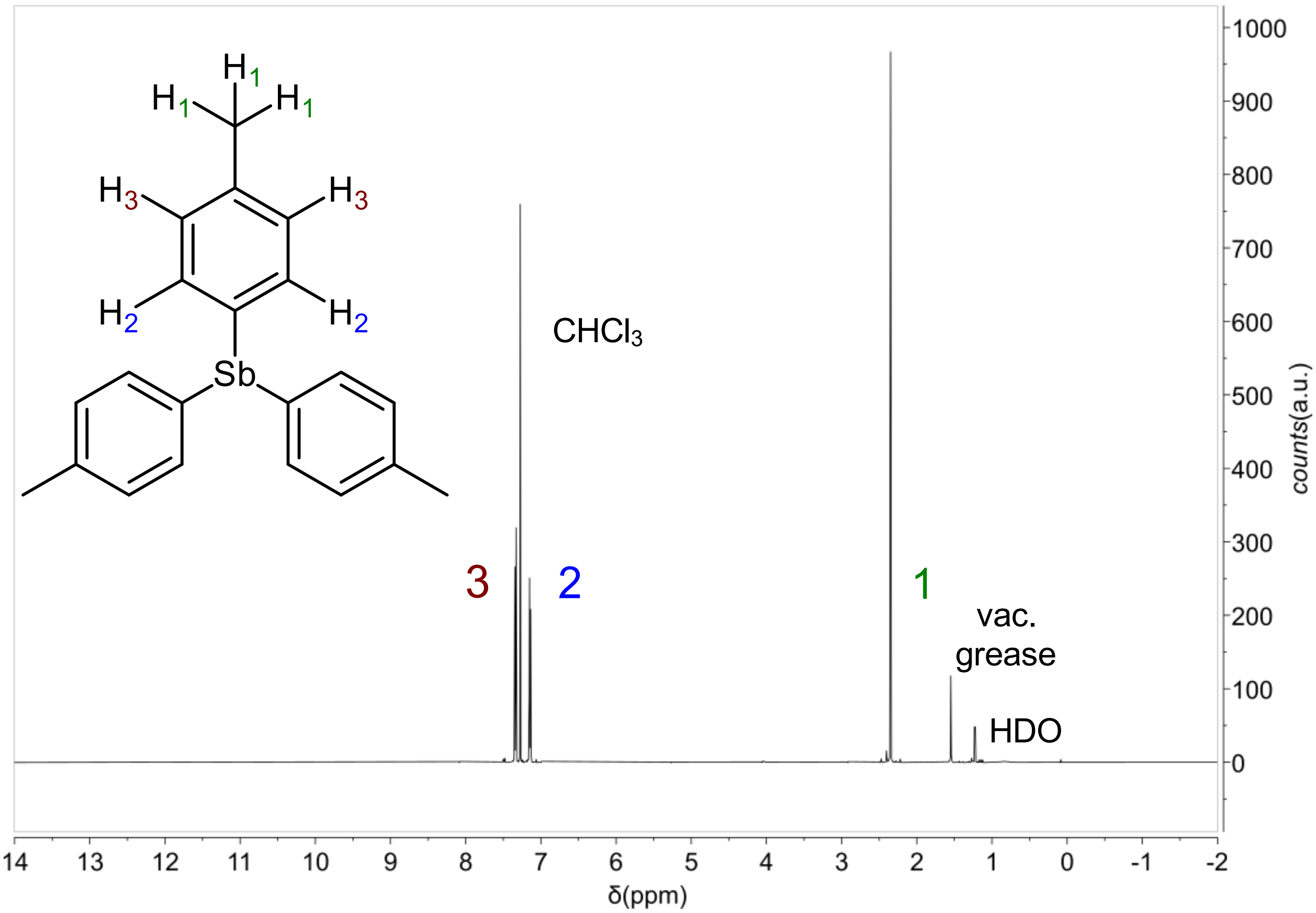

Figure S12. ^{1}H NMR spectrum of normal Sb(*p*-Tol)$_3$, collected on a Varian instrument operating at 499.78 MHz with 4 scans from 14 to −2 ppm. Shifts at 1.53 ppm correspond with traces of moisture in NMR solvent. Signals of suspect impurities of vacuum grease and/or silicon oil are highlighted.

^{1}H NMR of re-crystallized Sb(*p*-Tol)$_3$ in C_6D_6

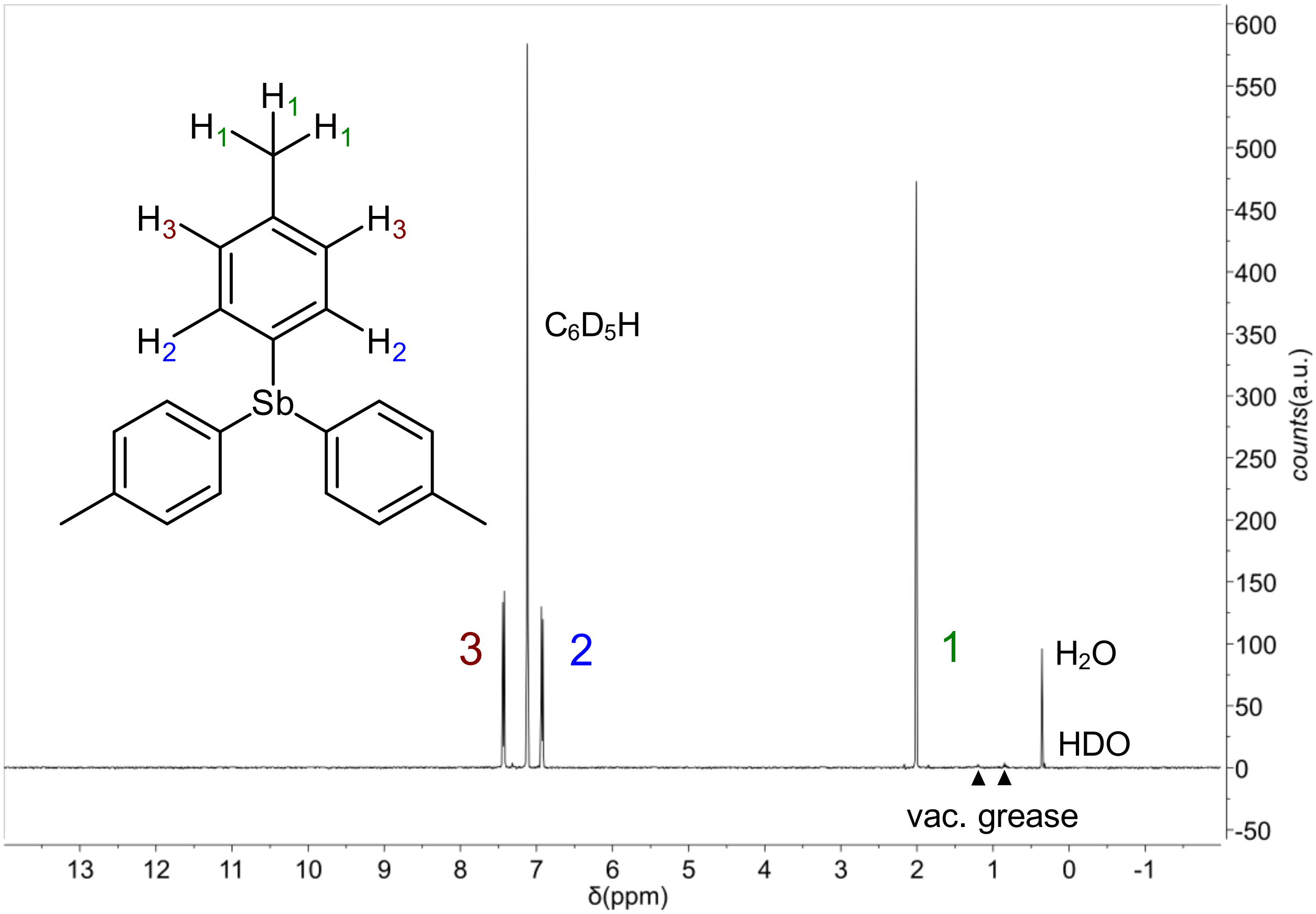

Figure S13. ^{1}H NMR spectrum of re-crystallized Sb(*p*-Tol)$_3$, collected on a Varian instrument operating at 400.12 MHz with 8 scans using a spectral width from 14 to −2 ppm. Signals at 0.40 and 0.36 ppm correspond to H_2O and HDO, respectively. Minor signals at 1.24 and 0.89 ppm are suspected to correspond to vacuum grease. Integrating the sharper peak at 0.89 ppm as a 6H -CH_3 peak for total molarity and multiplying by the molar mass of an arbitrary linear alkane of 25 carbon atoms chain length produces an estimated 0.07% of the NMR sample by mass. Apparent singlet at ca. 7.40 ppm with low intensity is the result of ^{13}C satellites from C_6D_5H and proton environment 3 overlapping.

^1H NMR integration of re-crystallized Sb(*p*-Tol)$_3$ in C_6D_6

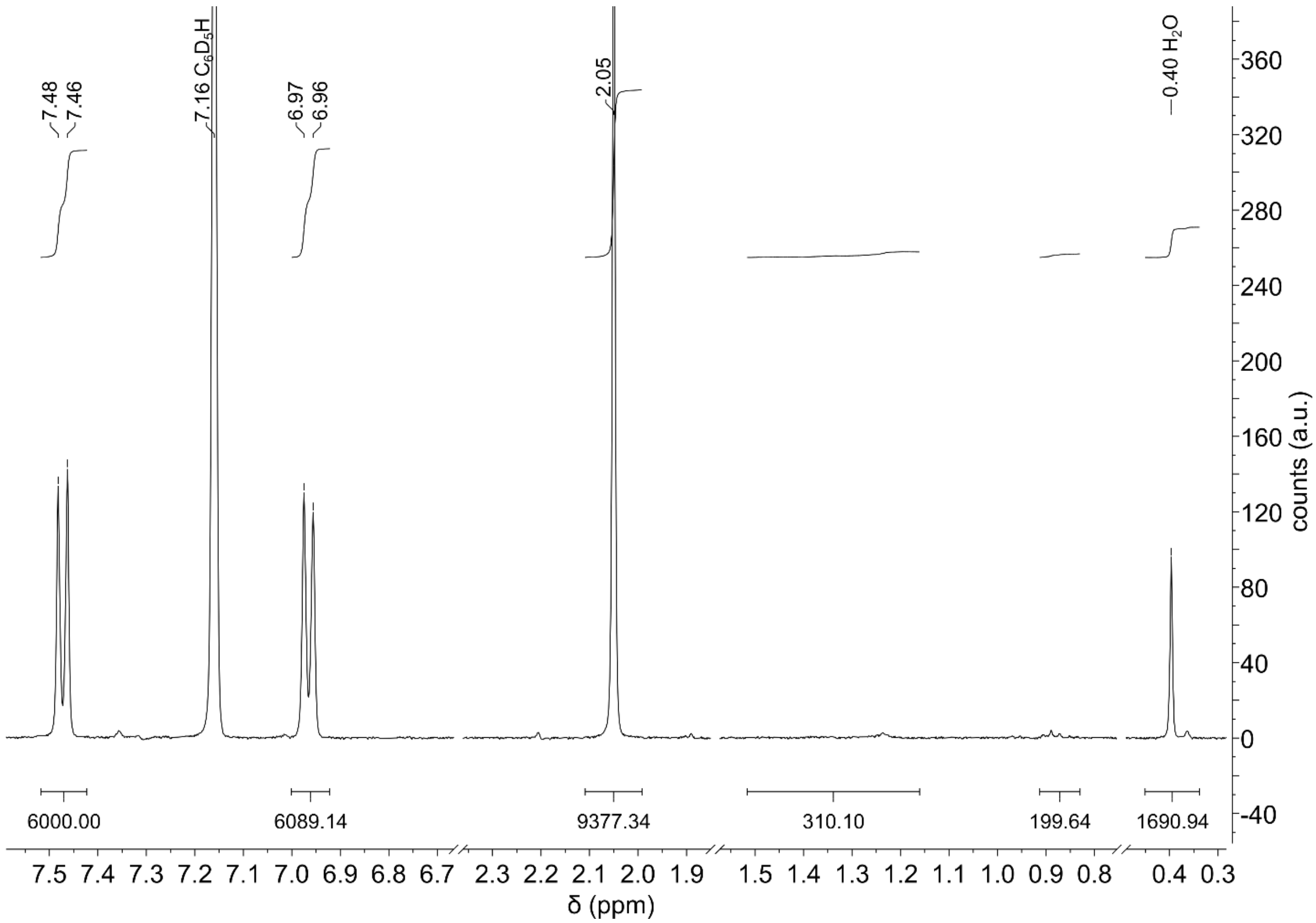

Figure S14. Enhanced view of integrals from ^1H NMR spectrum of re-crystallized Sb(*p*-Tol)$_3$, collected on a Varian instrument operating at 400.12 MHz with 8 scans using a spectral width from 14 to −2 ppm. Shifts at 0.40 and 0.36 ppm correspond to H_2O and HDO respectively. Empty sections of the spectrum are omitted for clarity, and integrals are normalized with the most downfield aromatic proton, normalized to number of protons multiplied by a factor of 1000 so that smaller integrals related to the presence of grease and of water are more easily compared.

^{1}H NMR of crude Sb(*p*-Tol)$_3$ in C_6D_6

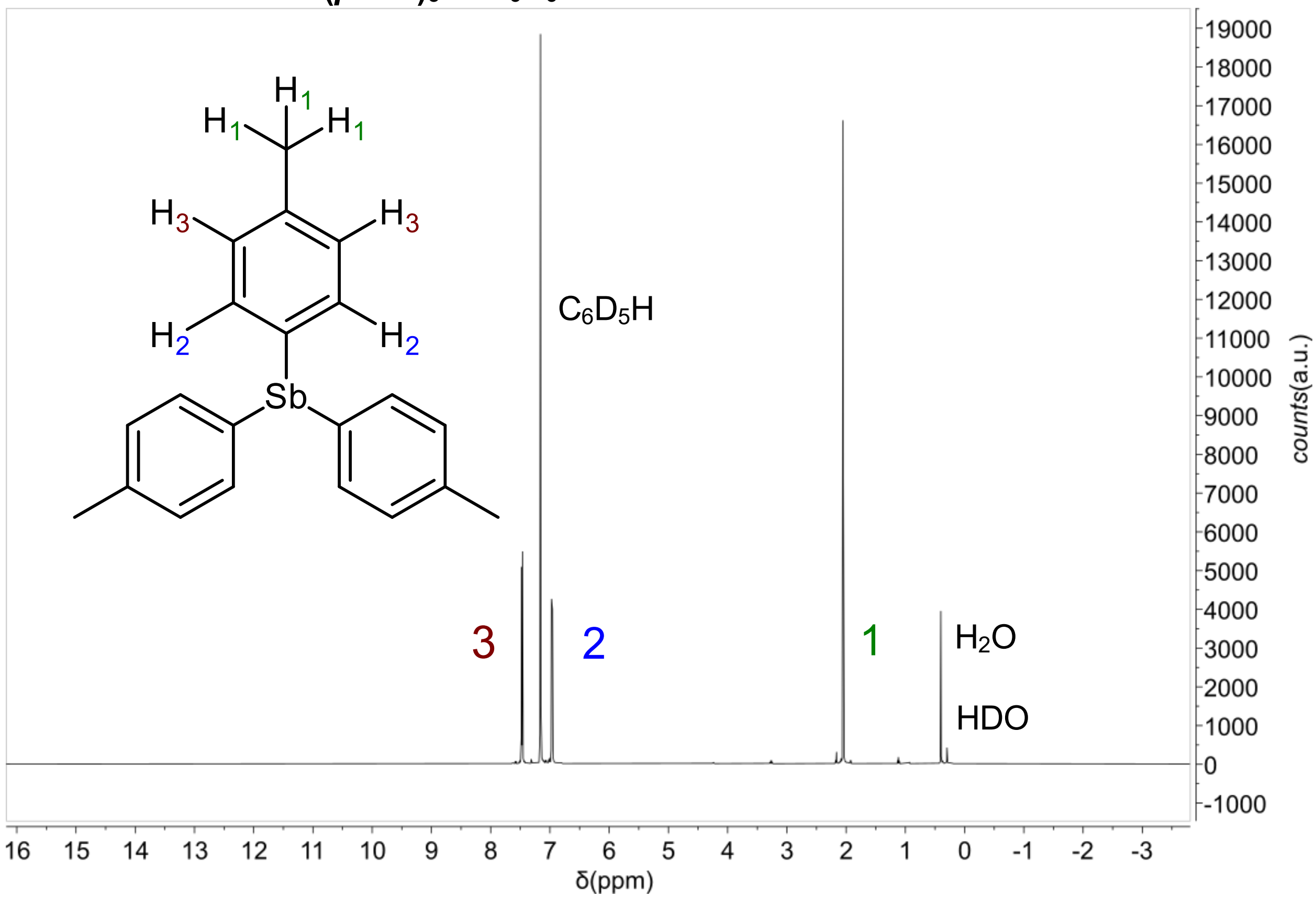

Figure S15. ^{1}H NMR spectrum of crude Sb(*p*-Tol)$_3$, collected on a Bruker AVIII500 HD instrument operating at 500.30 MHz with 16 scans using a spectral width from 16.2 to −3.8 ppm. Shifts at 0.40 and 0.36 ppm correspond with H_2O and HDO, respectively.

$^{13}C\{^1H\}$ NMR of re-crystallized $Sb(p\text{-}Tol)_3$ in CD_2Cl_2

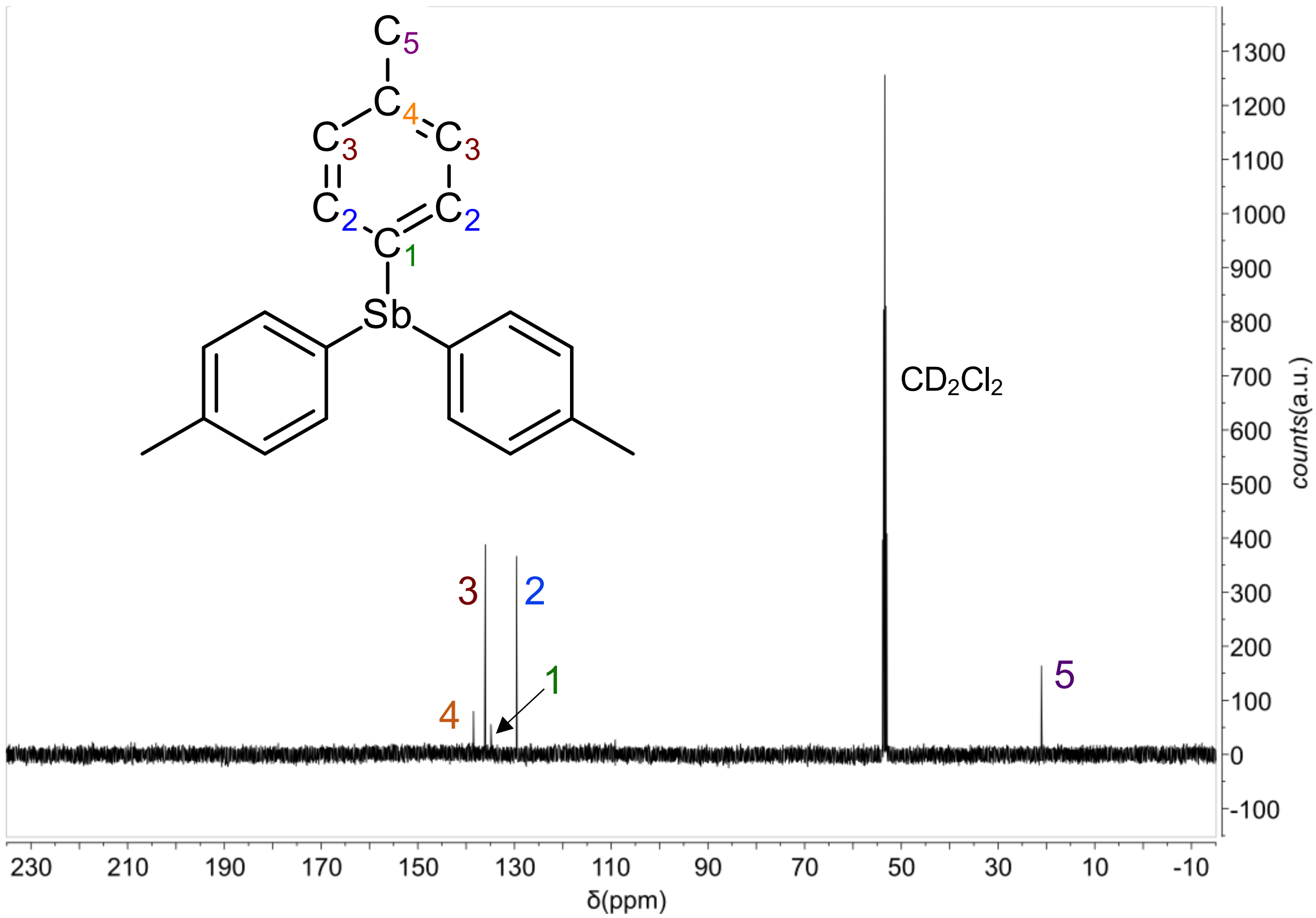

Figure S16. $^{13}C\{^1H\}$ NMR of re-crystallized $Sb(p\text{-}Tol)_3$ in CD_2Cl_2, collected on a Varian Mercury 400 MHz instrument operating at 125.68 MHz with 256 scans from 160 to −6 ppm.

Asymmetric Unit of Form I

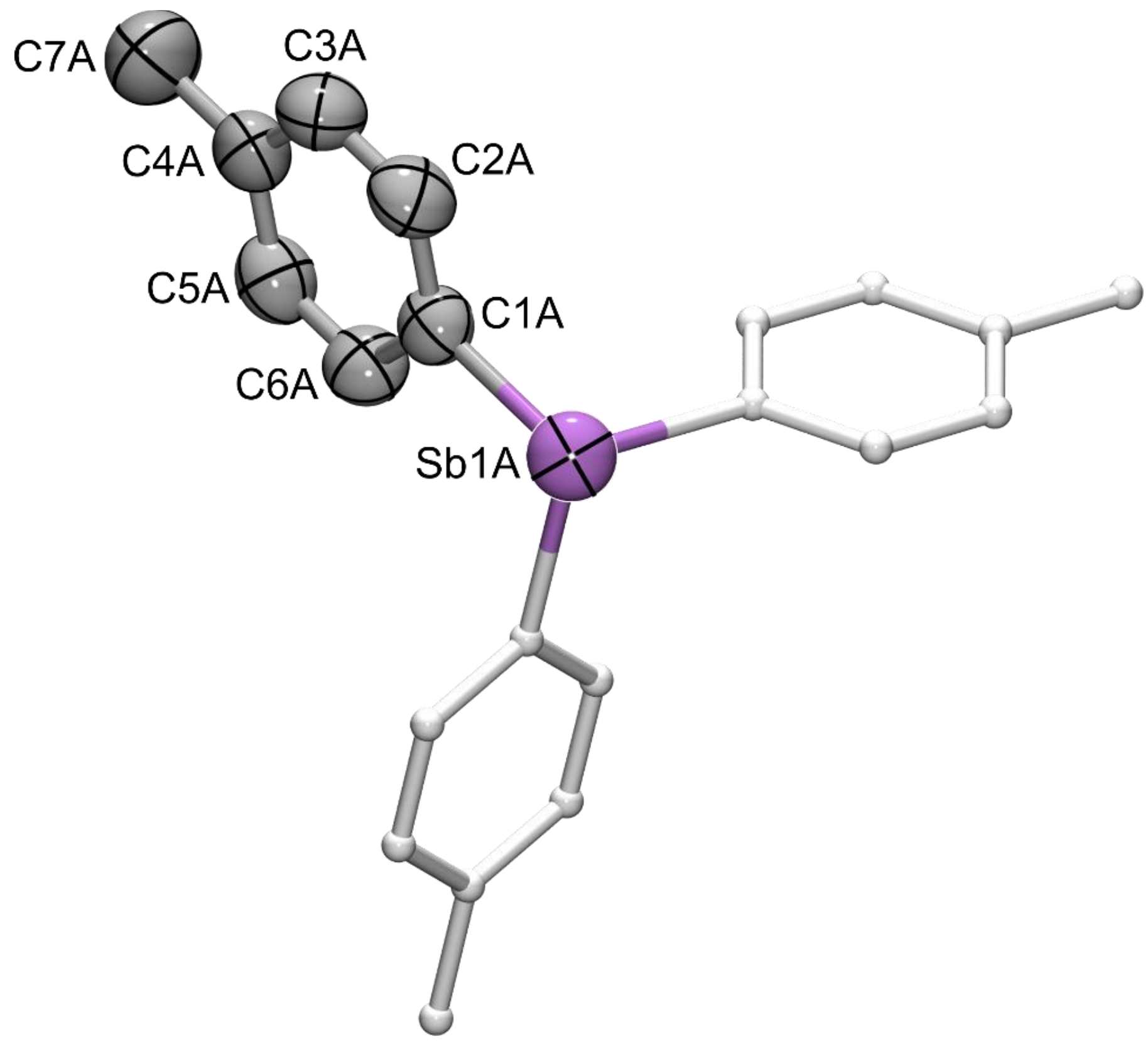

Figure S17. Asymmetric unit of Form I, collected at room temperature, showing the atom labelling scheme. Displacement ellipsoids are drawn at the 50 % probability level and the hydrogen atoms are omitted for clarity. The symmetry-dependent part molecule is shown in ball-and-stick model in light grey.

Asymmetric Unit of Form II

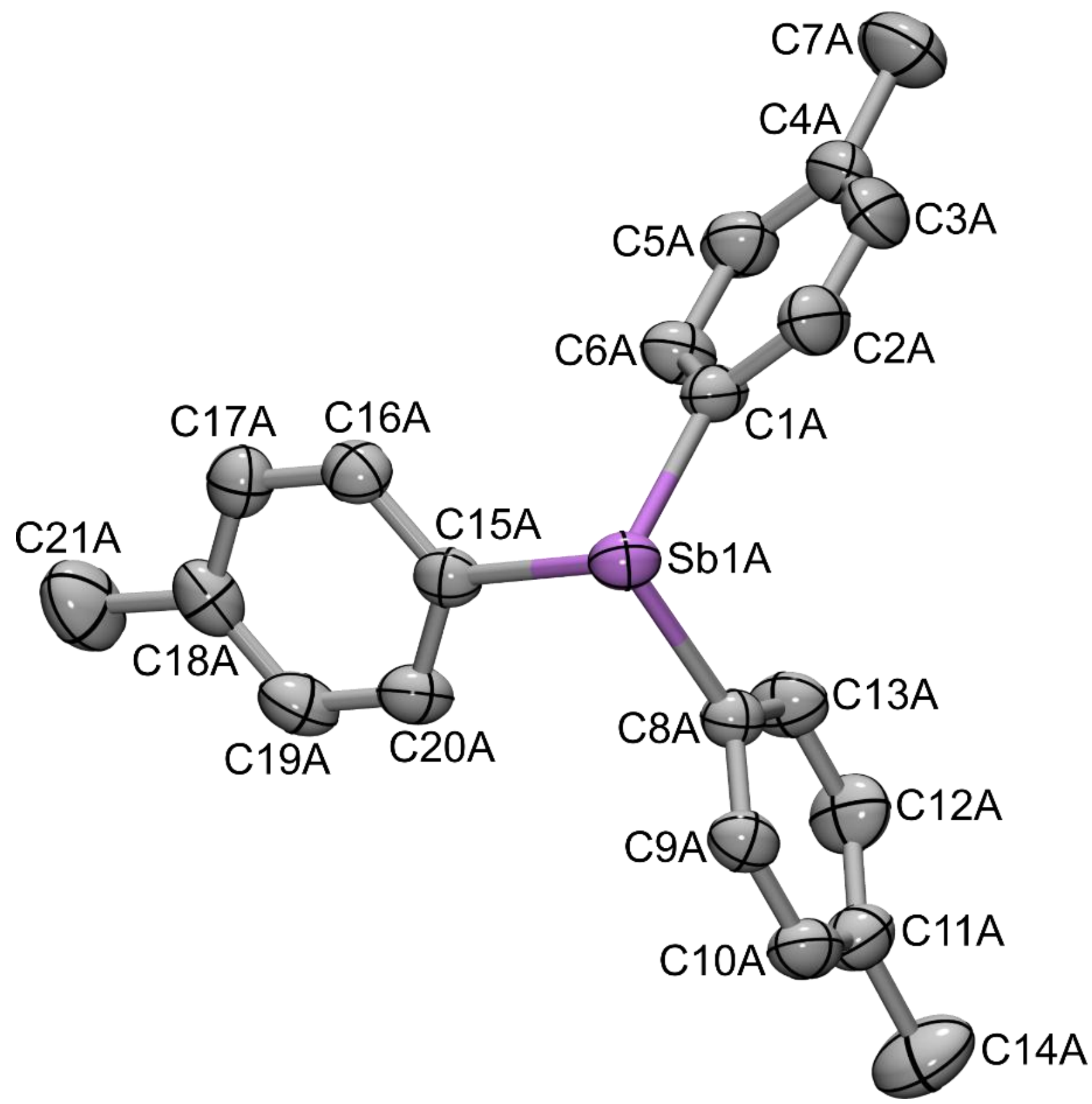

Figure S18. Asymmetric unit of Form II, collected at 253 K, showing the atom labelling scheme. Displacement ellipsoids are drawn at the 50 % probability level and the hydrogen atoms are omitted for clarity.

Hirshfeld Surface Analysis

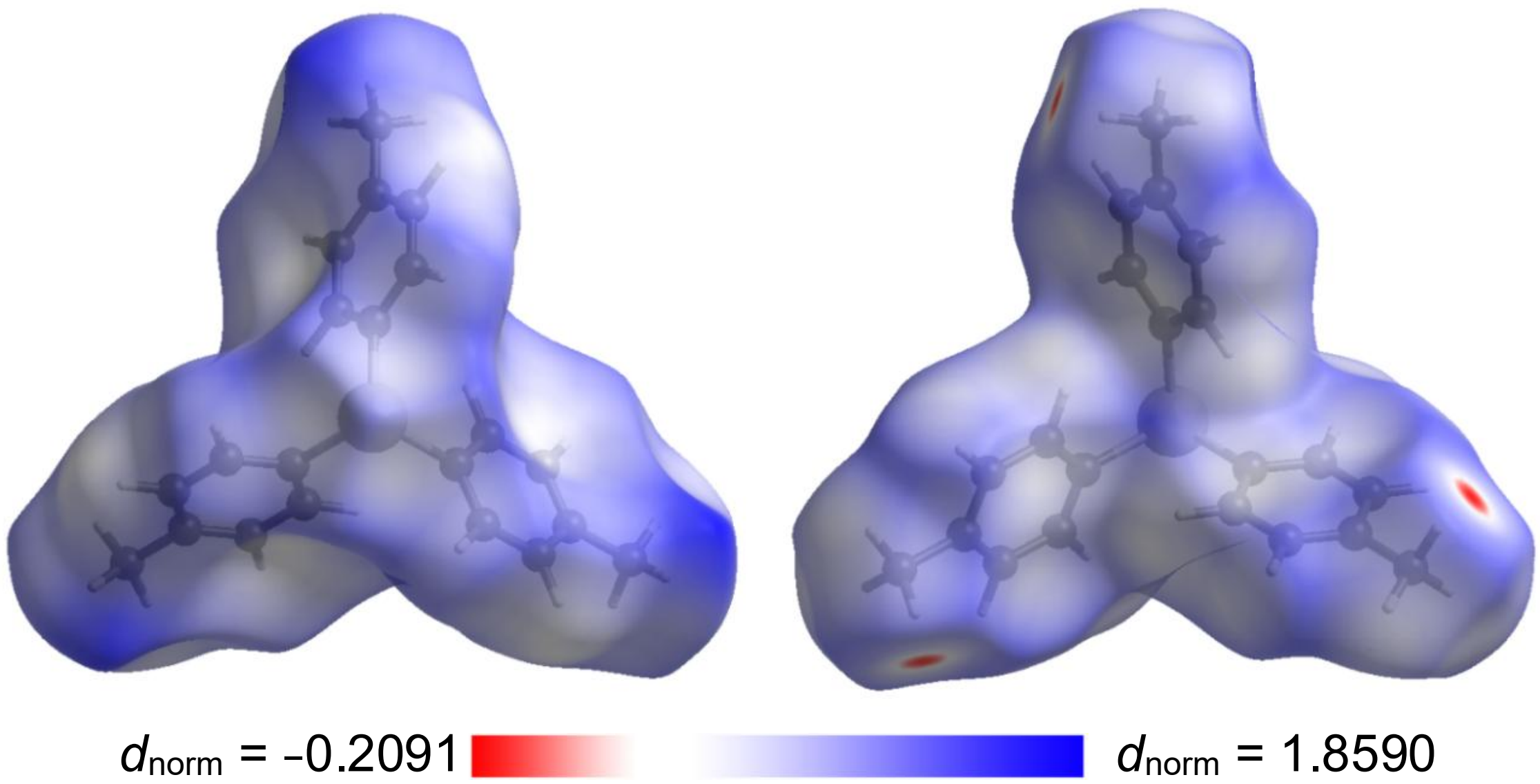

Figure S19. Front and back views (left to right) of $Sb(p\text{-}Tol)_3$ Form I Hirshfeld surface color-mapped with d_{norm} (see Equations S1,2). The only short contact observed is a methyl C-H···H-C contact across an inversion center.

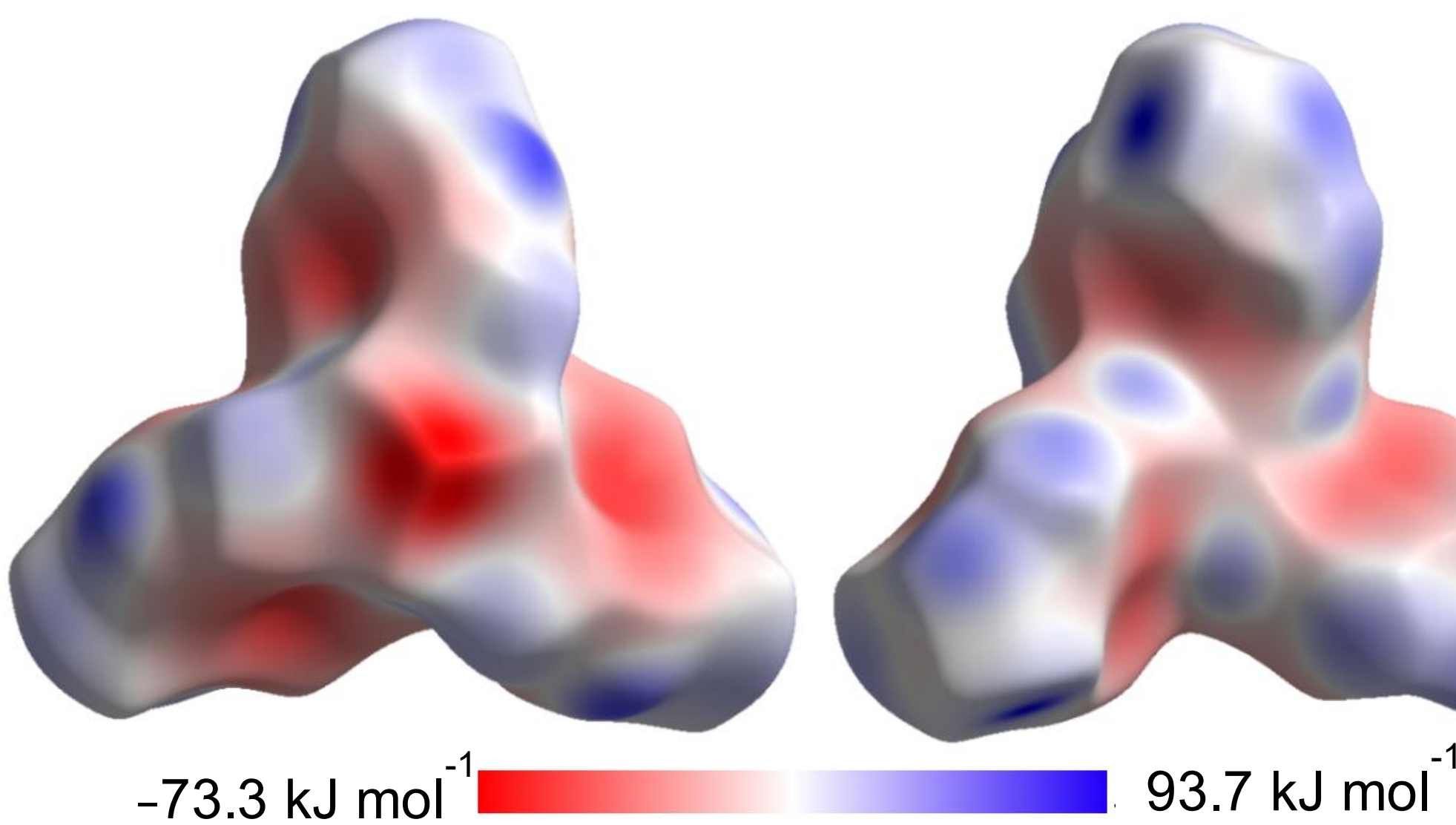

Figure S20. Front and back views (left to right) of $Sb(p\text{-}Tol)_3$ Form I Hirshfeld surface color-mapped with electrostatic potential, calculated with DFT using a B3LYP exchange potential[15-18] and the STO-3G basis[13,14] set in Crystal Explorer.[11,12]

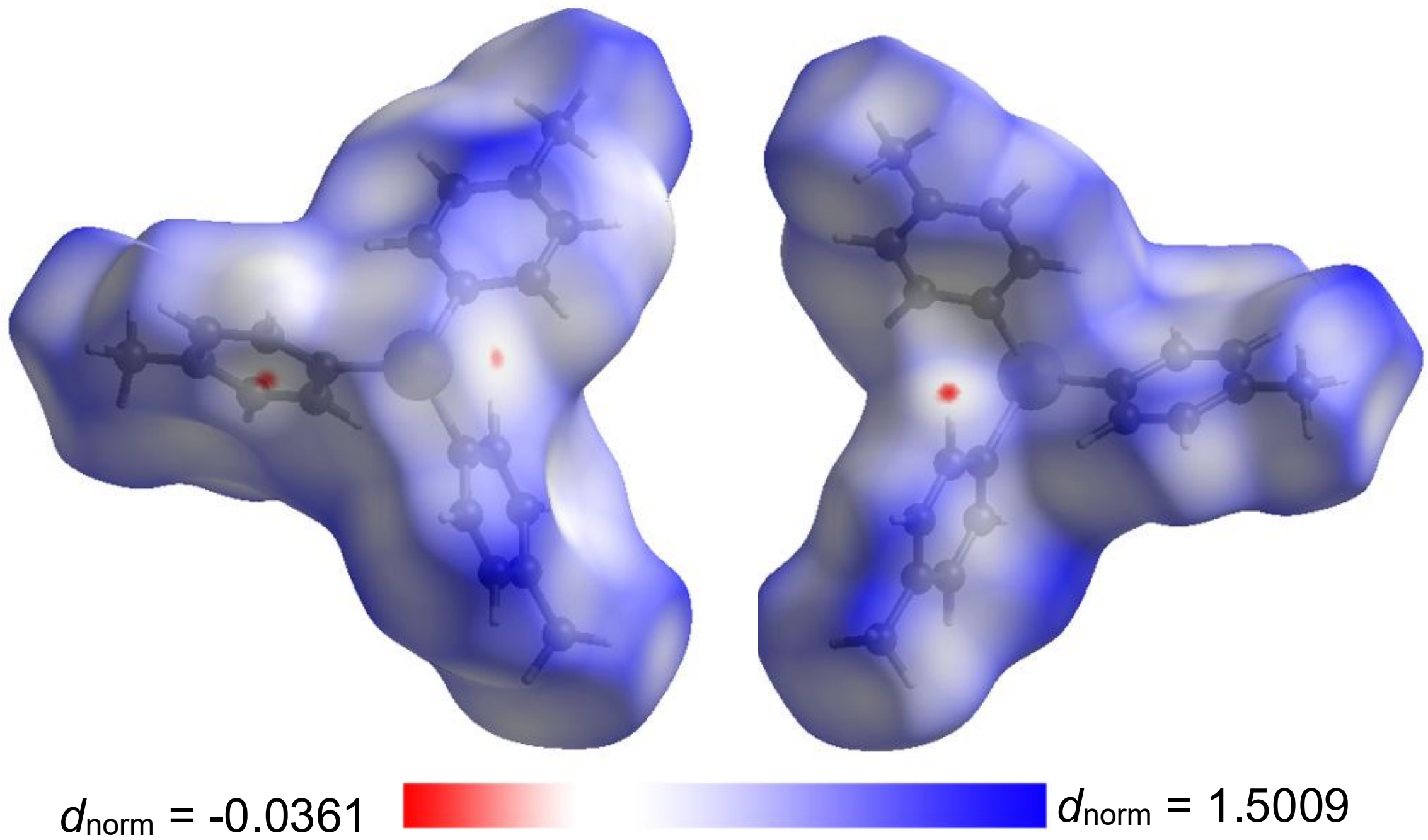

Figure S21. Arranged left to right are the front and back views of Sb(p-Tol)$_3$ Form II Hirshfeld surface color-mapped with d_{norm} (See Equations S1,2). Both ends of a C-H···π short contact, and Sb···Sb interaction across an inversion center can be seen.

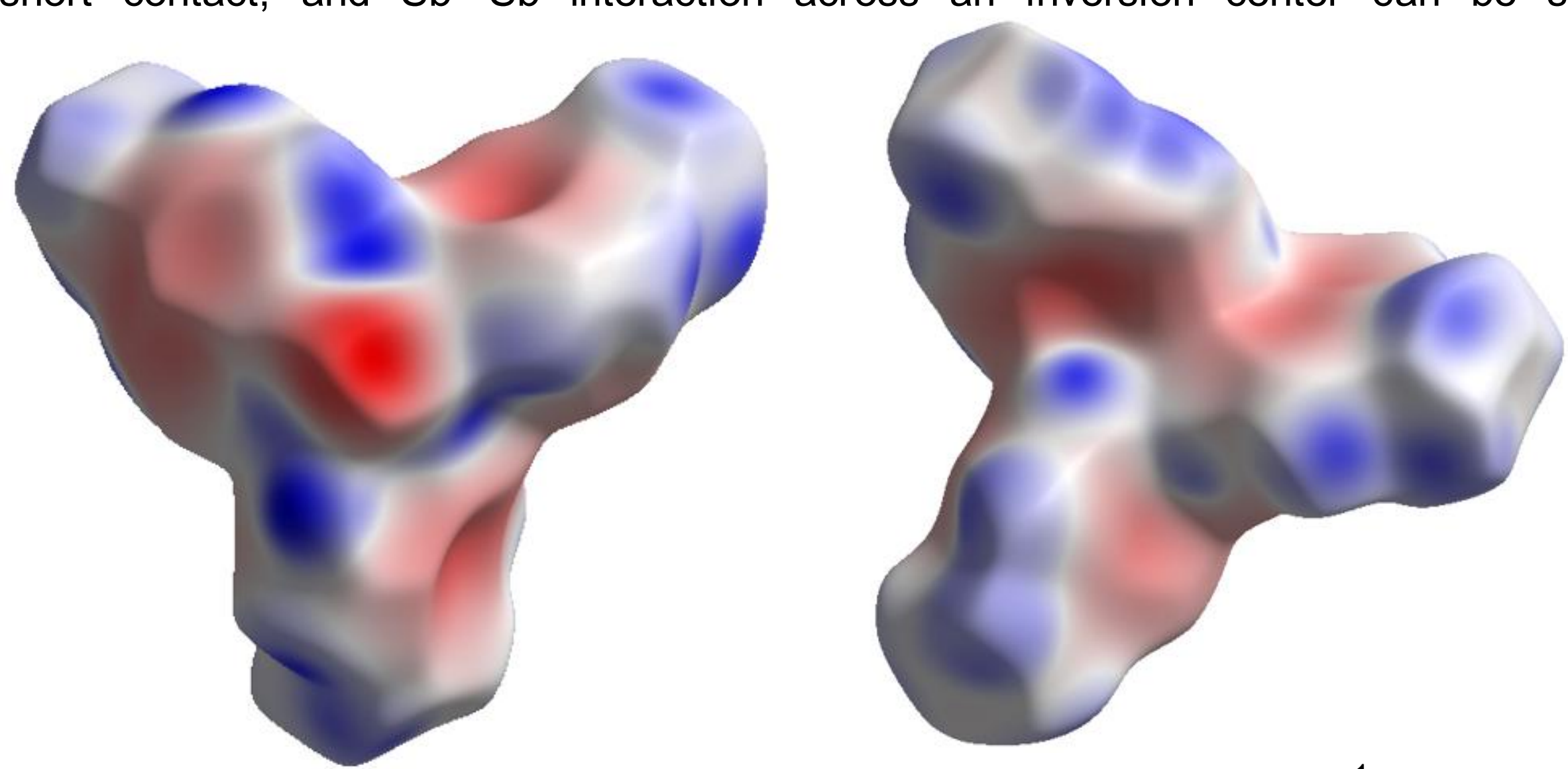

Figure S22. Front and back views (left to right) of Sb(p-Tol)$_3$ Form II Hirshfeld surface color-mapped with electrostatic potential, calculated with DFT using a B3LYP exchange potential[15-18] and the STO-3G basis[13,14] set in Crystal Explorer.[11,12]

Symmetry-reduced interaction energies

Interactions are color-coded and tabulated with parameters: N – number of symmetry-equivalent dimers made by the molecule within the Hirshfeld surface; Symop – symmetry operations which generate neighboring, symmetry-equivalent dimers; Electron Density – a description of the method/basis set used to determine the wave function and calculate energies; and energies of each given interaction, split into four factors from left to right: electrostatic interaction, polarization energy, dispersion forces, and steric repulsion. **Note:** E totals are not determinable, as these summations rely on semi-empirical energy component scale-factors, which have only been developed for MP2 and HF- methods coupled with respective basis sets 6-31G(d,p) and 3-12G.[11,12]

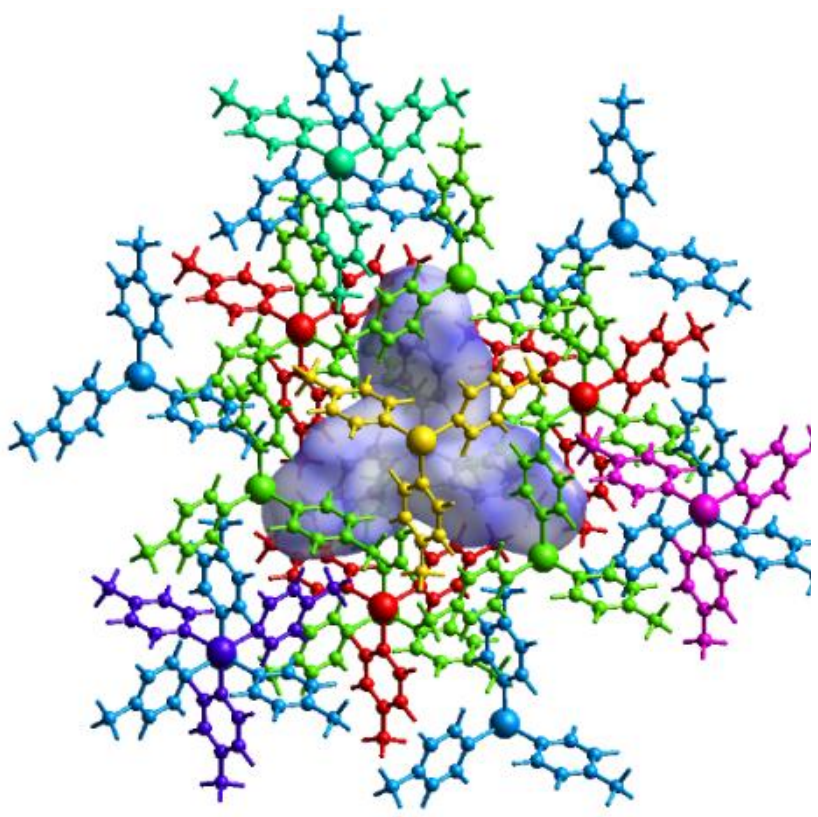

	N	Symop	R	Electron Density	E_ele	E_pol	E_dis	E_rep
	3	-x+1/3, -y+2/3, -z+2/3	7.62	DFT/STO-3G	-3.4	-0.6	-41.3	11.9
	1	-x, -y, -z	4.94	DFT/STO-3G	-5.0	-0.7	-79.5	21.2
	6	x+2/3, y+1/3, z+1/3	10.00	DFT/STO-3G	-0.6	-0.1	-9.3	2.4
	1	-x, -y, -z	13.74	DFT/STO-3G	-0.3	-0.1	-6.4	0.0
	6	x, y, z	12.82	DFT/STO-3G	-1.0	-0.0	-6.3	0.0
	1	-x, -y, -z	13.74	DFT/STO-3G	0.9	-0.1	-6.4	0.0
	1	-x, -y, -z	13.74	DFT/STO-3G	0.7	-0.1	-6.4	0.0

Figure S23. Shell of molecules within 3.8 Å of the molecule inside the HS table of calculated symmetry-reduced interaction energies of Form I. Molecules are color-coded for identification tabulated interaction energy values (in kJ mol^{-1}).

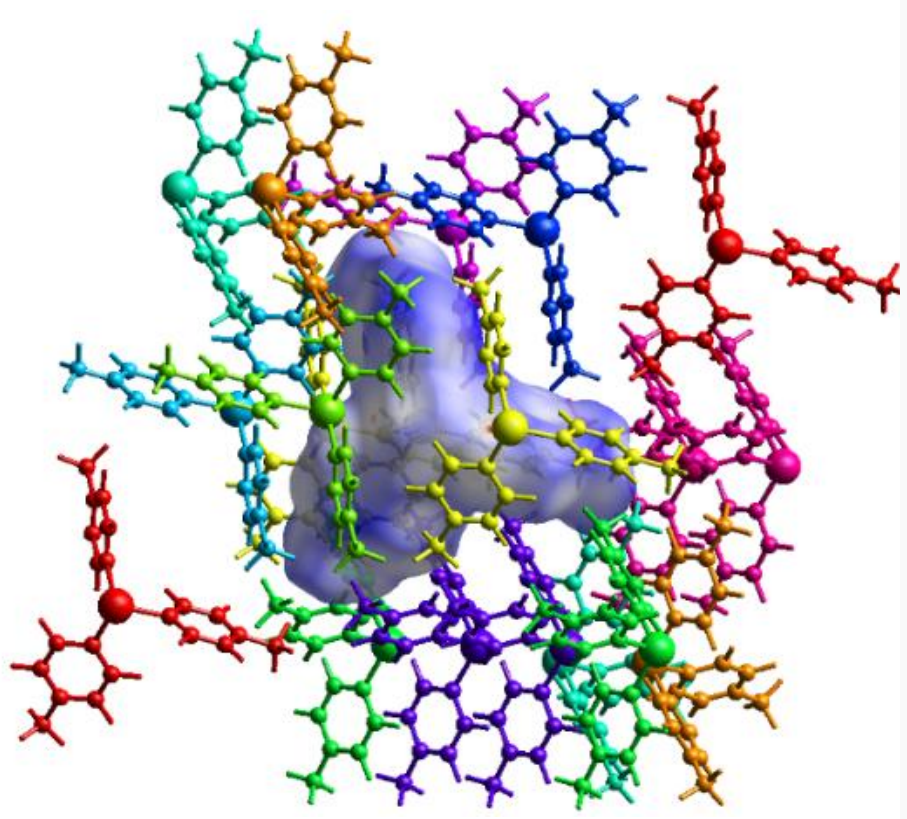

	N	Symop	R	Electron Density	E_ele	E_pol	E_dis	E_rep
	2	x, y, z	13.39	DFT/STO-3G	0.1	-0.0	-2.2	0.0
	2	x, -y+1/2, z+1/2	13.21	DFT/STO-3G	-0.1	-0.0	-1.8	0.0
	2	x, y, z	6.42	DFT/STO-3G	-4.1	-0.7	-57.8	20.9
	1	-x, -y, -z	6.09	DFT/STO-3G	-6.3	-0.8	-37.1	23.1
	2	-x, y+1/2, -z+1/2	12.90	DFT/STO-3G	0.1	-0.0	-6.8	0.0
	2	x, -y+1/2, z+1/2	12.46	DFT/STO-3G	0.2	-0.0	-3.6	0.0
	1	-x, -y, -z	7.70	DFT/STO-3G	-3.2	-0.6	-46.1	15.7
	1	-x, -y, -z	8.62	DFT/STO-3G	-2.0	-0.5	-31.4	8.4
	2	-x, y+1/2, -z+1/2	9.15	DFT/STO-3G	-1.2	-0.3	-26.2	6.6
	1	-x, -y, -z	9.82	DFT/STO-3G	-1.3	-0.2	-28.0	7.2
	2	-x, y+1/2, -z+1/2	12.68	DFT/STO-3G	-1.4	-0.0	-7.5	0.0

Figure S24. Shell of molecules within 3.8 Å of the molecule inside the HS table of calculated symmetry-reduced interaction energies of Form II. Molecules are color-coded for identification tabulated interaction energy values (in kJ mol^{-1}).

VT-PXRD pattern of concurrent Form I/II mixture

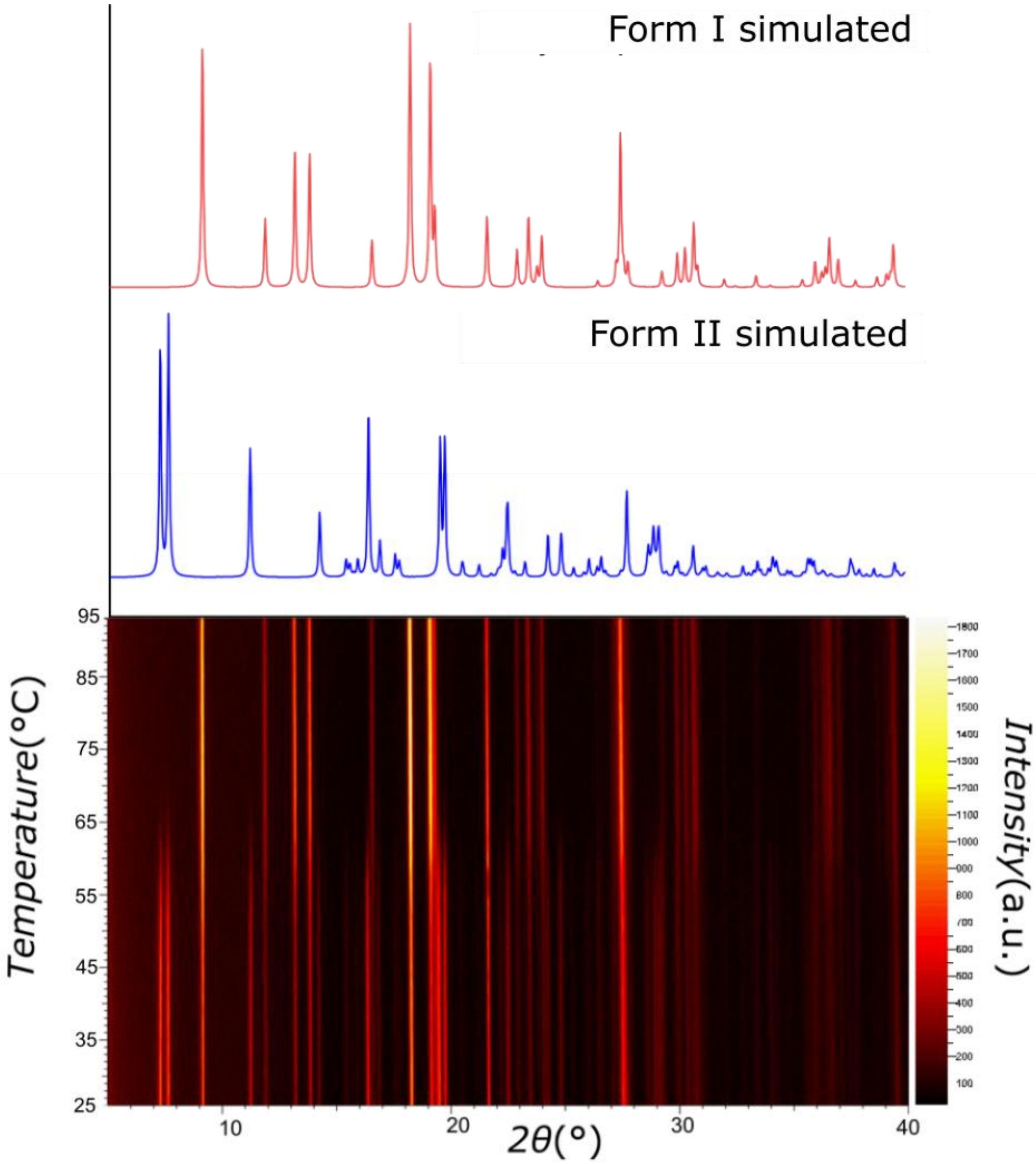

Figure S25. Variable temperature PXRD pattern (from top to bottom): calculated for the herein redetermined structure of Sb(*p*-Tol)$_3$ Form I; calculated for the structure of Sb(*p*-Tol)$_3$ Form II; the temperature-resolved diffractogram measured on a ca. 100 mg sample of a mixture of Form I and II. The temperature was ramped from 25 to 95 ˚C at a rate of 1 ˚C every 6 minutes, scanning with each 1˚C change. The sample for the VT-PXRD experiment was obtained by combining five smaller (ca. 25 mg) batches, each prepared by heating a sample of Sb(*p*-Tol)$_3$ in a vial immersed in an oil bath maintained at 140 ˚C until complete melting was confirmed (based on visual observation), removing to solidify, and repeating the process 10 times.

Percentage composition by temperature plot from Rietveld refinement of VT-PXRD data

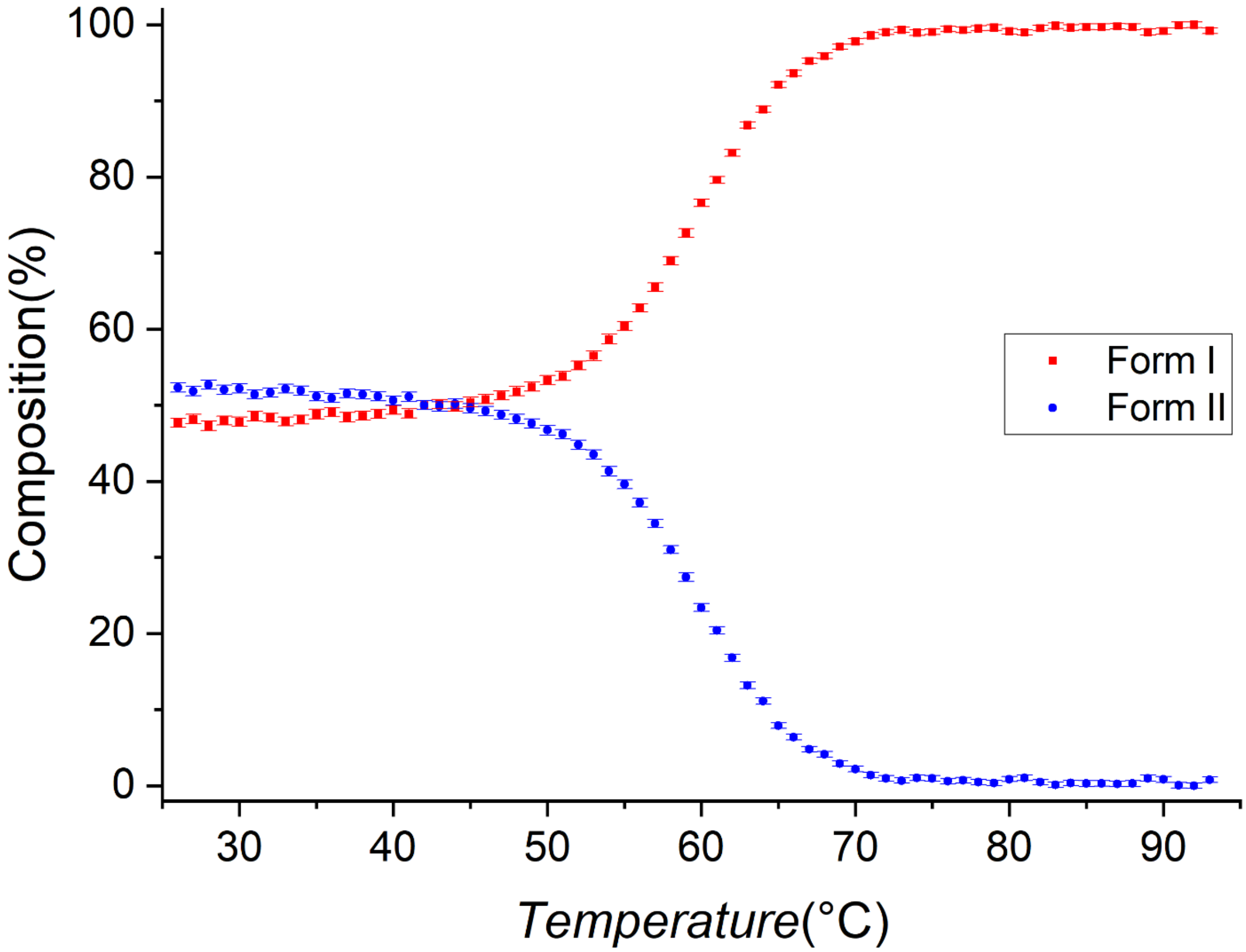

Figure S26. Plot of the change in content of Form I and Form II during heating, based on Rietveld refinement of the VT-PXRD data. Error bars are shown (largest error ±0.63%).

MAS-SSNMR Spectra

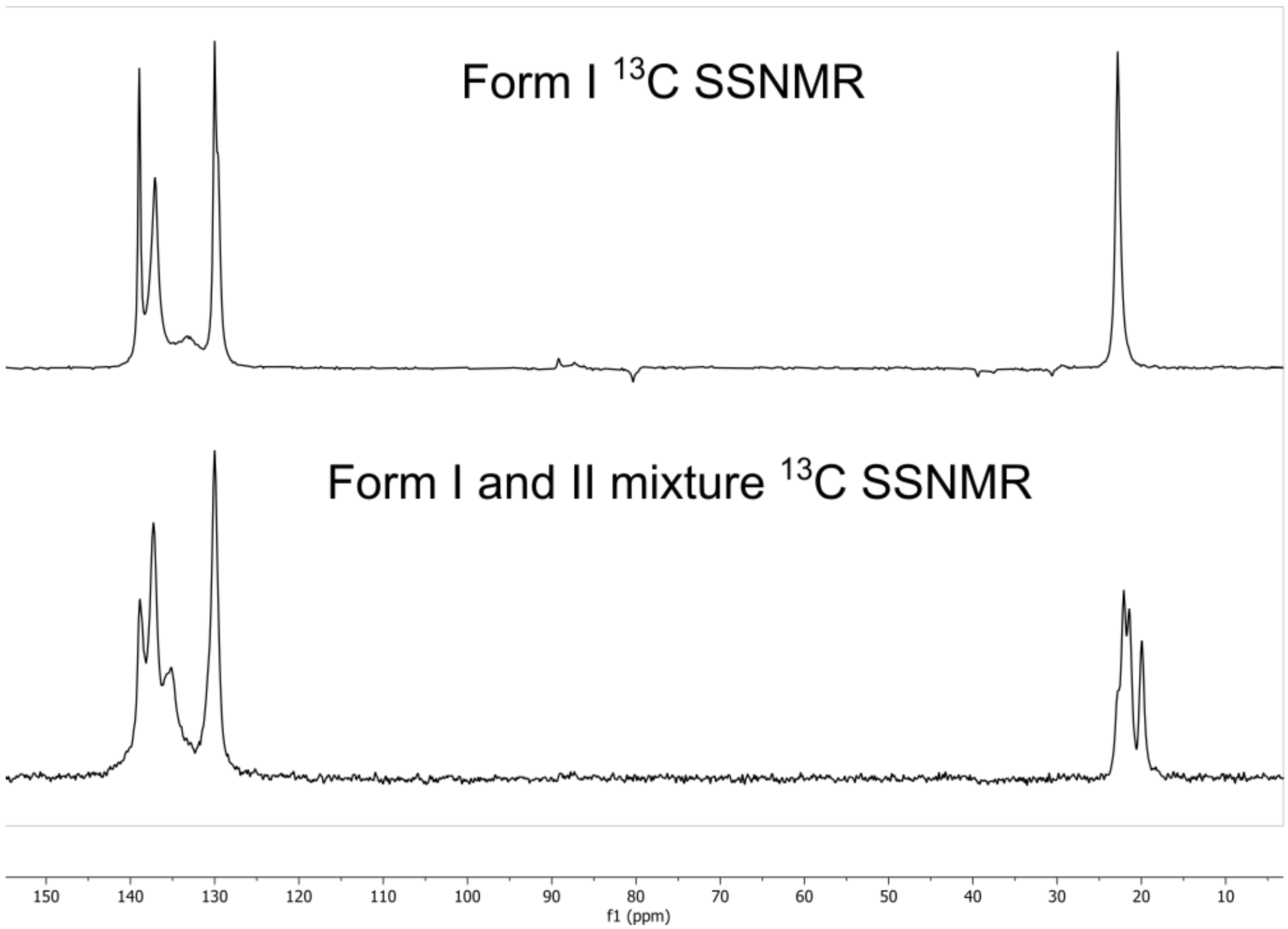

Figure S27. Comparison of $^{13}C\{^{1}H\}$ Magic Angle Spinning Solid-state NMR spectra collected on a Varian 400 MHz instrument operating at 100 MHz at 298 K using a TANCPXTOSS pulse program (from top to bottom): Form I Sb(*p*-Tol)$_3$, Sb(*p*-Tol)$_3$ mixture of forms I and II. Approximately 100 mg of sample was used per measurement, with 512 scans collected in each case.

^{13}C MAS SSNMR Form I: (Varian 400 MHz) δ 139.0, 137.1, 133.2, 130.0, 22.8 ppm.

^{13}C MAS SSNMR Form I and II: (Varian 400 MHz) δ 138.9, 137.3, 135.2, 130.0, 22.8, 22.1, 21.4, 20.0 ppm.

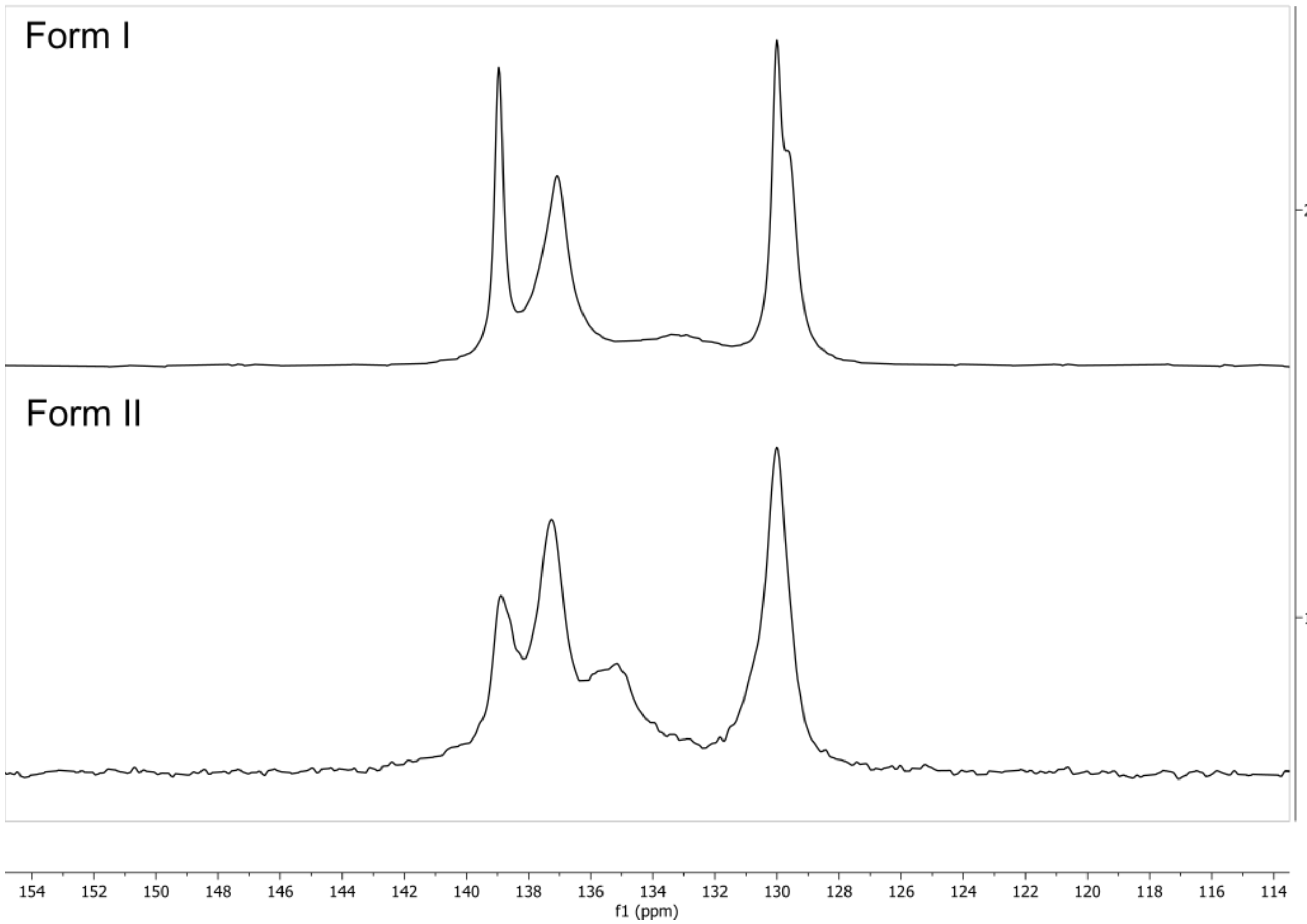

Figure S28. Comparison of $^{13}C\{^1H\}$ Magic Angle Spinning Solid-state NMR spectra collected on a Varian 400 MHz instrument operating at 100 MHz at 298 K using a TANCPXTOSS pulse program, focusing on the aromatic carbon region (from top to bottom): Form I Sb(*p*-Tol)$_3$, Sb(*p*-Tol)$_3$ mixture of Forms I and II.

^{13}C MAS SSNMR Form I: (Varian 400 MHz) δ 139.0, 137.1, 133.2, 130.0 ppm.

^{13}C MAS SSNMR Form I and II: (Varian 400 MHz) δ 138.9, 137.3, 135.2, 130.0 ppm.

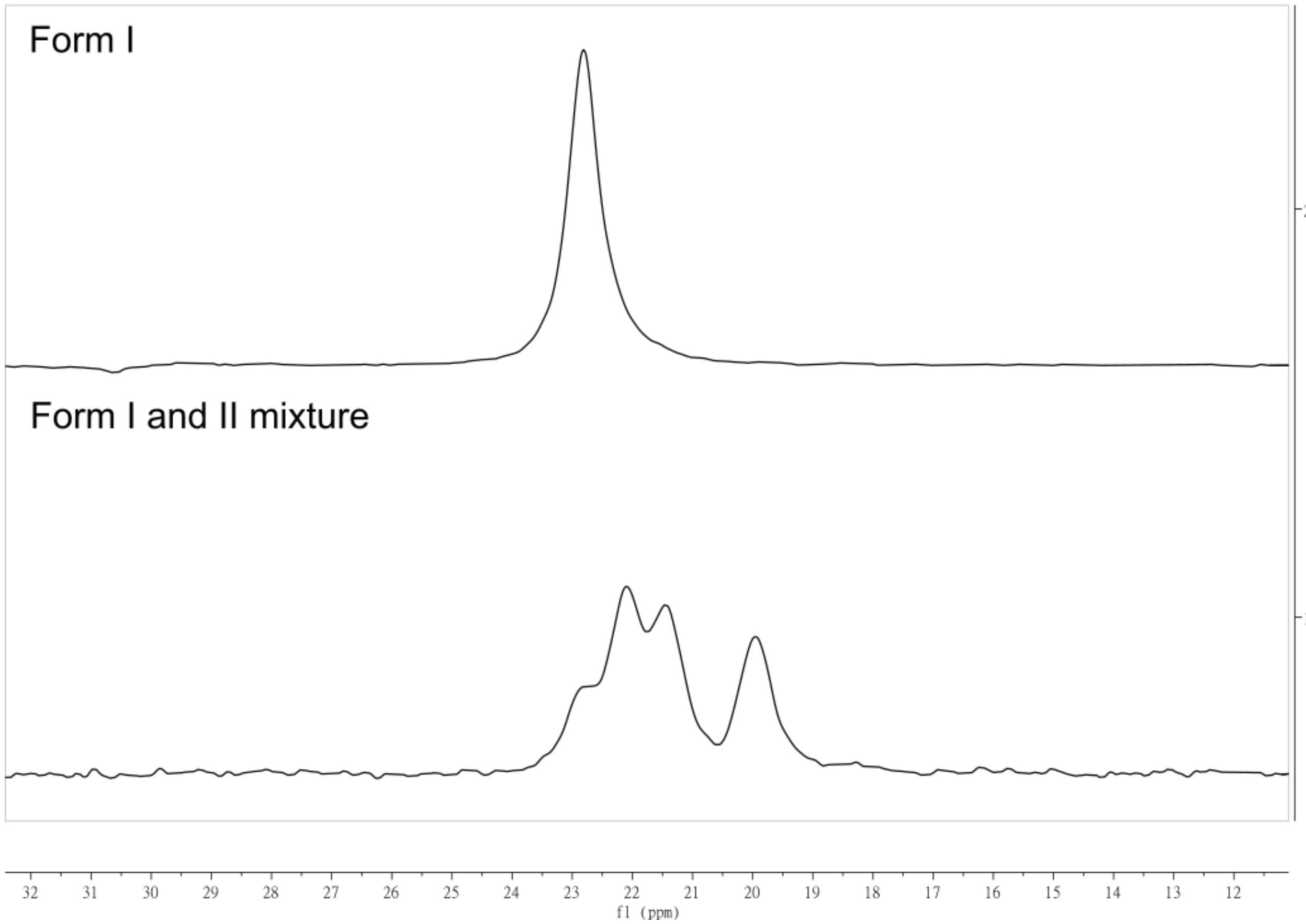

Figure S29. Comparison of $^{13}C\{^{1}H\}$ Magic Angle Spinning Solid-state NMR spectra collected on a Varian 400 MHz instrument operating at 100 MHz at 298 K using a TANCPXTOSS pulse program, focusing on the aliphatic carbon region (from top to bottom): Form I Sb(*p*-Tol)$_3$, Sb(*p*-Tol)$_3$ mixture of Forms I and II.

^{13}C MAS SSNMR Form I: (Varian 400 MHz) δ 22.8 ppm.

^{13}C MAS SSNMR Form I and II: (Varian 400 MHz) δ 22.8, 22.1, 21.4, 20.0 ppm.

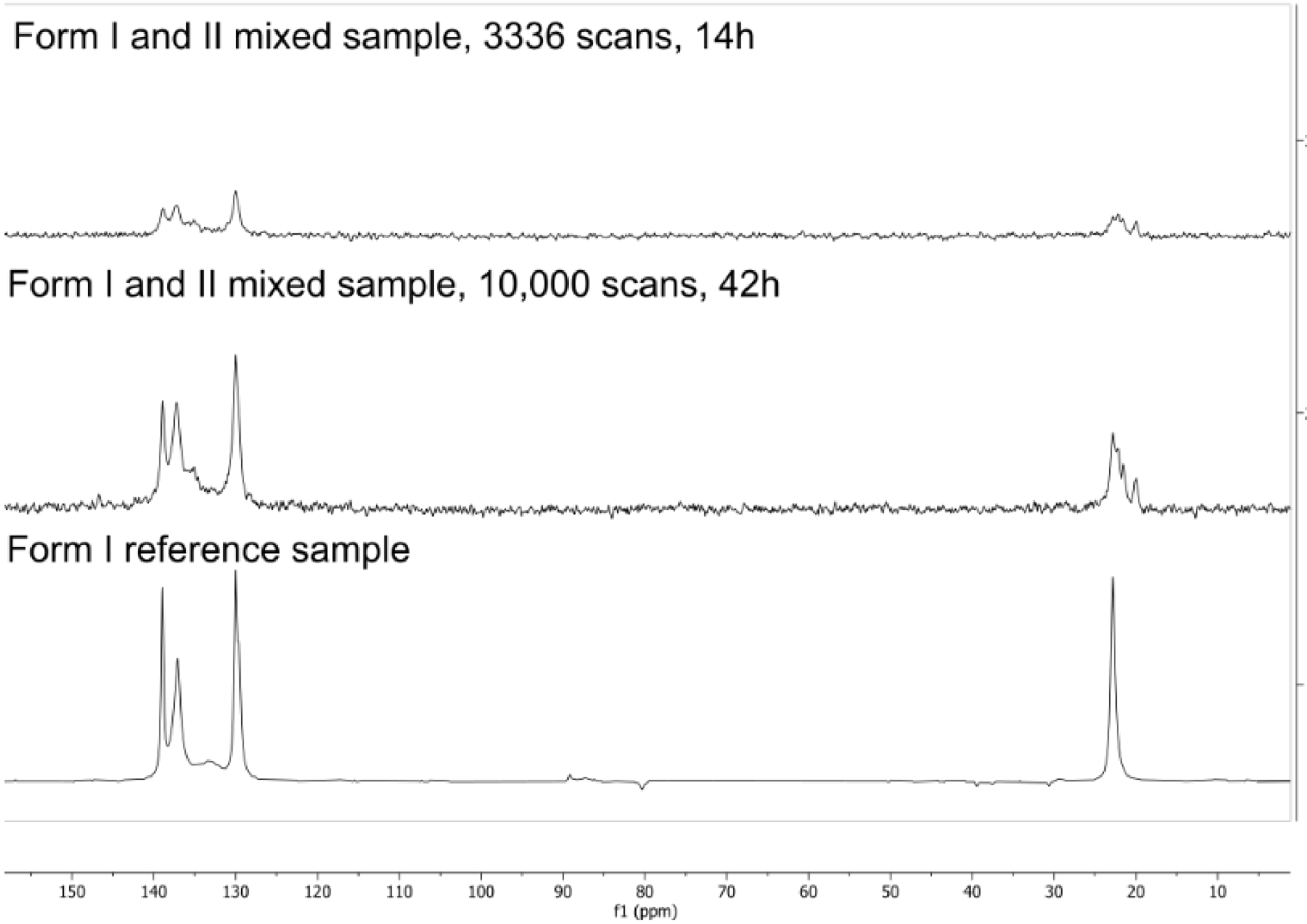

Figure S30. $^{13}C\{^{1}H\}$ Solid-state NMR collected on a Varian 400 MHz instrument operating at 100 MHz at 298 K using a TANCPXTOSS pulse program. Approximately 12 mg of sample was used (from top to bottom): predominantly Form II sample after 14 hours and then 42 hours in the solid-state rotor with 3336 and 10,000 scans being collected at each respective interval. Form I reference scan is a 100 mg sample after 512 scans.

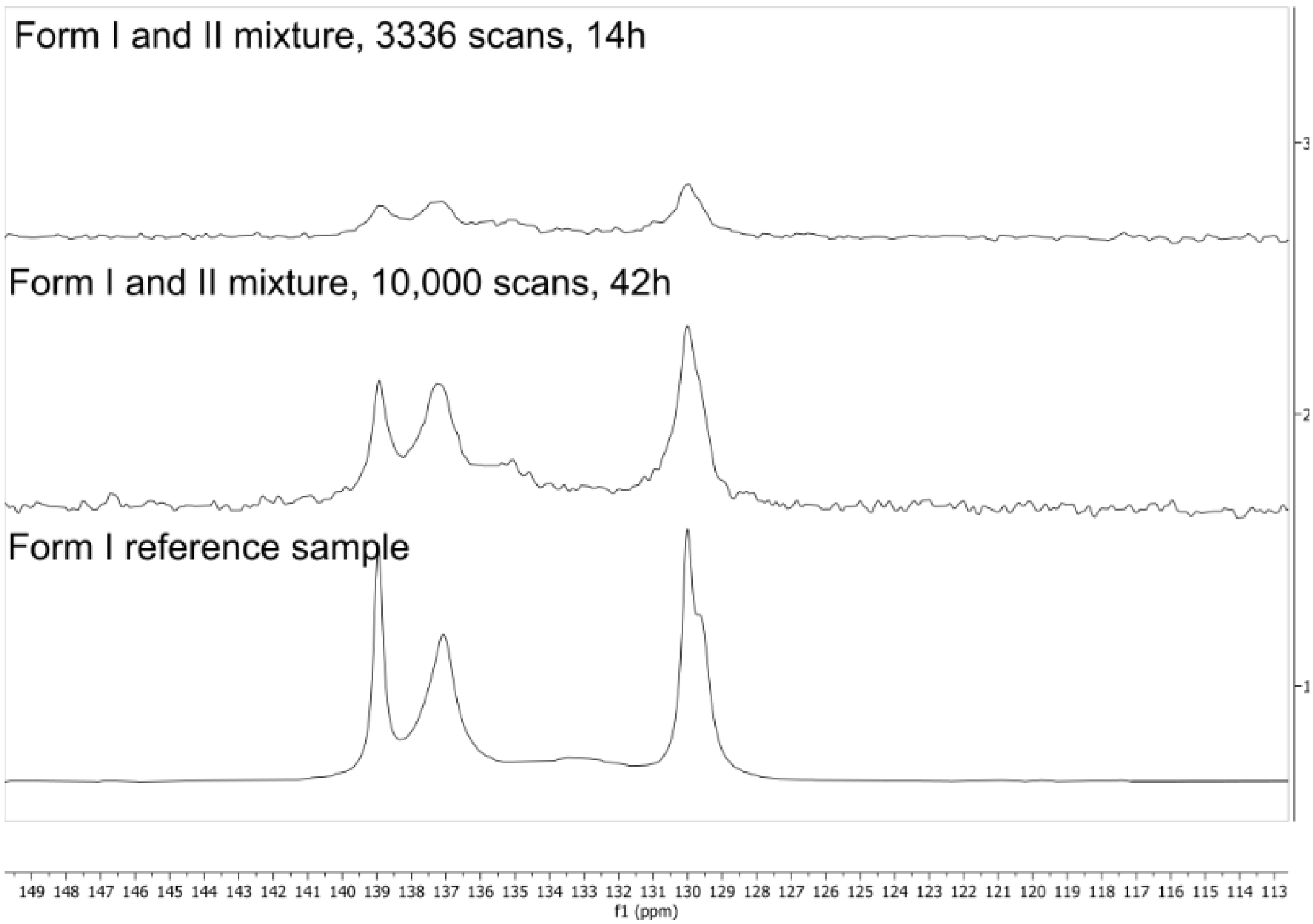

Figure S31. $^{13}C\{^1H\}$ Solid-state NMR collected on a Varian 400 MHz instrument operating at 100 MHz at 298 K using a TANCPXTOSS pulse program. Approximately 12 mg of sample was used (from top to bottom): predominantly Form II sample after 14 hours and then 42 hours in the solid-state rotor with 3336 and 10,000 scans being collected at each respective interval. Enhanced view of aromatic carbon region. Form I reference scan is a 100 mg sample after 512 scans.

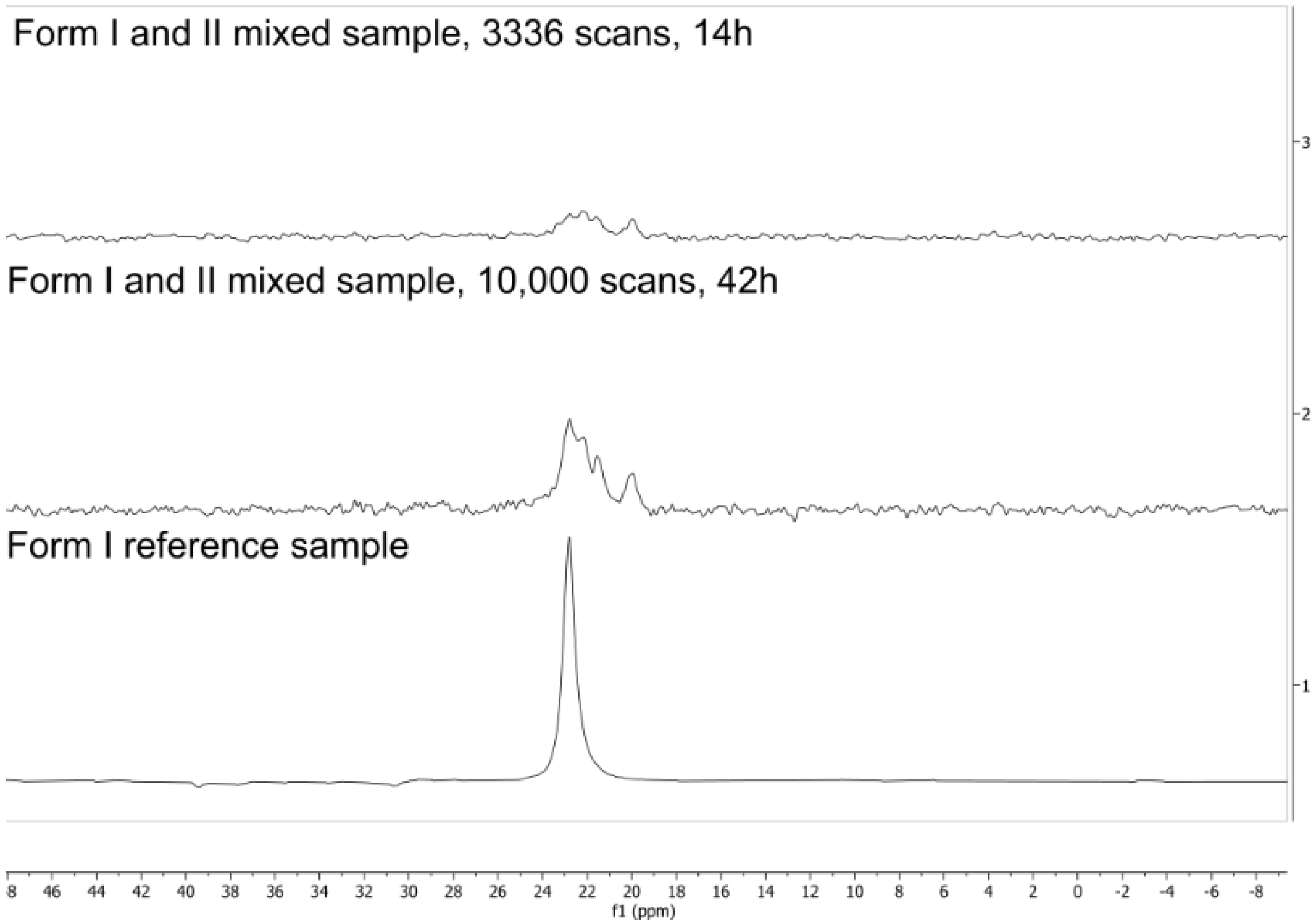

Figure S32. $^{13}C\{^{1}H\}$ Solid-state NMR collected on a Varian 400 MHz instrument operating at 100 MHz at 298 K using a TANCPXTOSS pulse program. Approximately 12 mg of sample was used (from top to bottom): predominantly Form II sample after 14 hours and then 42 hours in the solid-state rotor at with 3336 and 10,000 scans being collected at each respective interval. Enhanced view of aliphatic carbon region. Form I reference scan is a 100 mg sample after 512 scans.

FTIR-ATR spectra

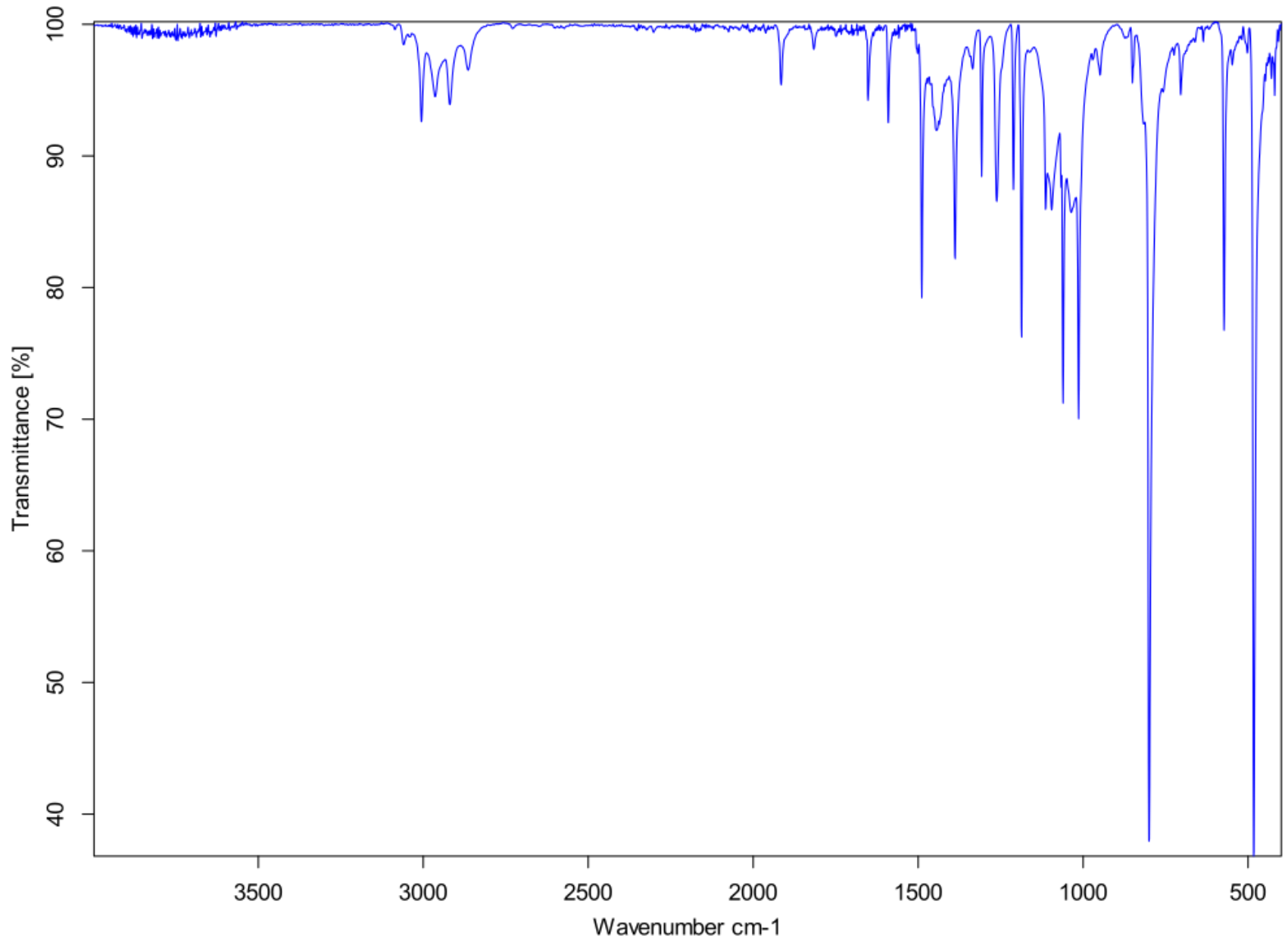

Figure S33. Solid-state FT-IR ATR infrared spectrum of Sb(*p*-Tol)$_3$ Form I.

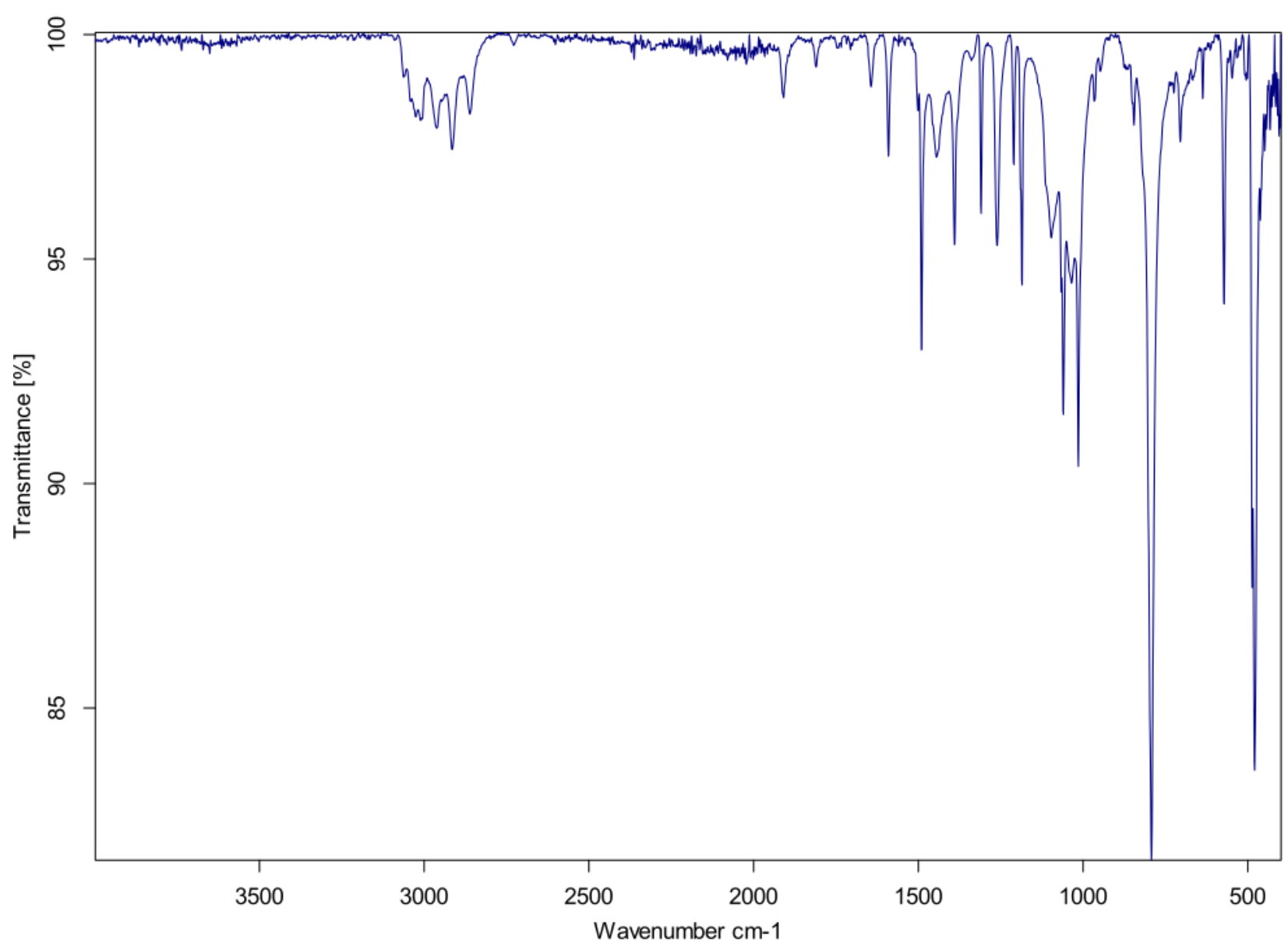

Figure S34. Solid-state FT-IR ATR infrared spectrum of Sb(*p*-Tol)$_3$ Form II.

High Resolution Mass Spectrometry

Acquisition Parameter

Source Type	APCI	Ion Polarity	Positive	Set Nebulizer	4.0 Bar
Focus	Not active	Set Capillary	4000 V	Set Dry Heater	150 °C
Scan Begin	90 m/z	Set End Plate Offset	-500 V	Set Dry Gas	1.5 l/min
Scan End	1250 m/z	Set Charging Voltage	2000 V	Set Divert Valve	Source
		Set Corona	4000 nA	Set APCI Heater	350 °C

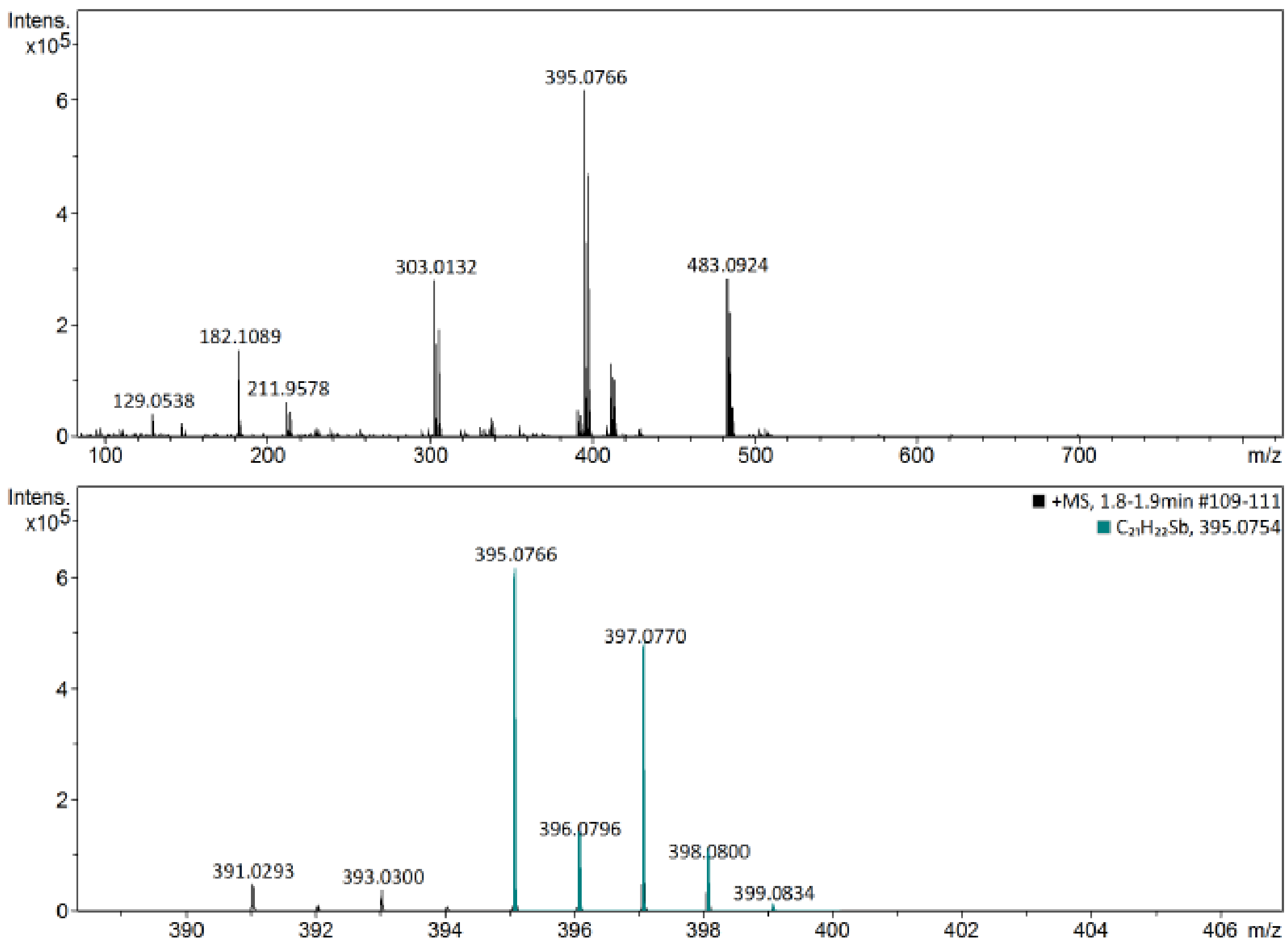

Figure S35. High resolution atmospheric pressure chemical ionization mass spectrum of re-crystallized Sb(*p*-Tol)$_3$. [M+H$^+$] peak corresponding to protonated Sb(*p*-Tol)$_3$ at 395.0766 *M*/*Z* is enhanced to display isotropic splitting pattern (bottom panel). The peak at 483.0924 *M*/*Z* corresponds to the *M*/*Z* ratio of the species [*p*-Tol$_4$Sb$^{\bullet+}$−2H], a species accessible via previously observed triarylpnictogen fragmentation pathways.[53]

Acquisition Parameter

Source Type	APCI	Ion Polarity	Positive	Set Nebulizer	4.0 Bar
Focus	Not active	Set Capillary	4000 V	Set Dry Heater	150 °C
Scan Begin	90 m/z	Set End Plate Offset	-500 V	Set Dry Gas	1.5 l/min
Scan End	1250 m/z	Set Charging Voltage	2000 V	Set Divert Valve	Source
		Set Corona	4000 nA	Set APCI Heater	350 °C

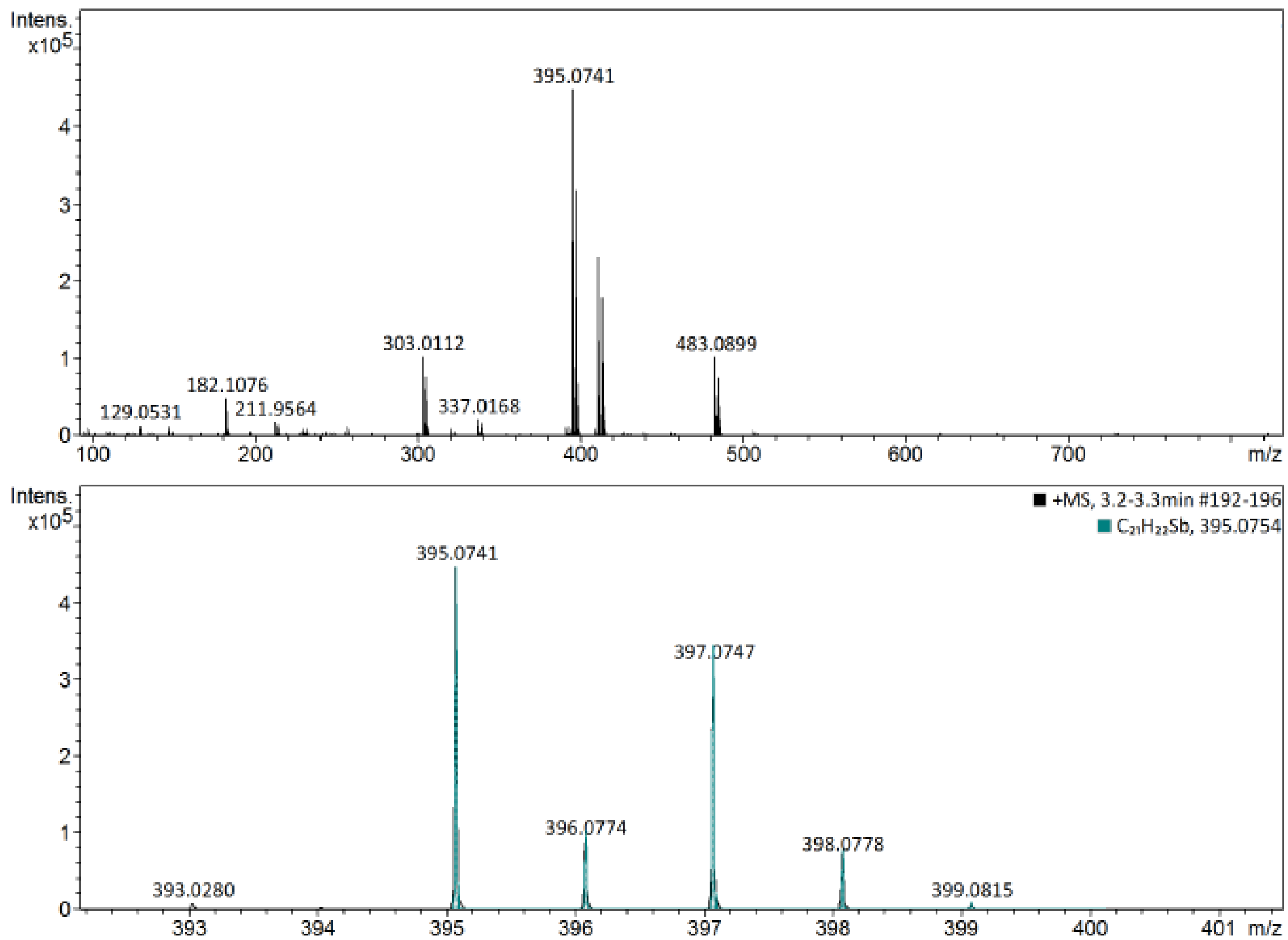

Figure S36. High resolution atmospheric pressure chemical ionization mass spectrum of crude Sb(*p*-Tol)$_3$. The main peak of 395.07 *M*/*Z* is attributed to [M+H$^+$]. The corresponding *M*/*Z* range is enhanced to display isotropic splitting pattern. Peaks at 129.0531 and 211.9564 *M*/*Z* correspond to expected *M*/*Z* for [(Sb(*p*-Tol)$_3$–(C$_7$H$_7$)$_2$)$^{•+}$] and [(Sb(*p*-Tol)$_3$–C$_7$H$_7$)$^{•+}$]. The corresponding coupled bi-aryl species [(C$_7$H$_7$)$_2$$^+$] is observed at 182.1076 M/Z. The peak at 483.0899 *M*/*Z* corresponds to the *M*/*Z* ratio of the species [*p*-Tol$_4$Sb$^{•+}$–2H].[53]

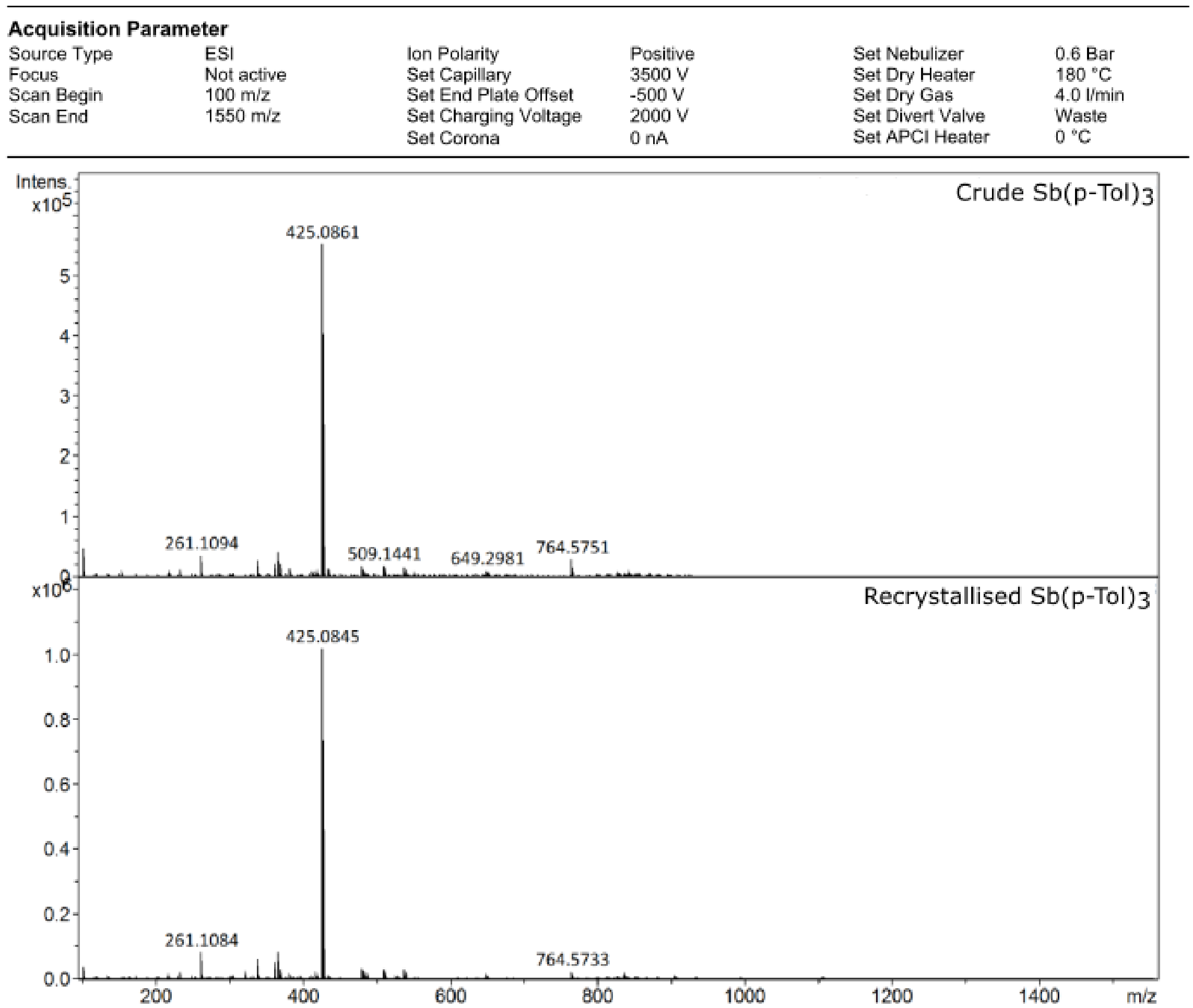

Figure S37. High-resolution electrospray ionization mass spectrums of crude and re-crystallized Sb(*p*-Tol)$_3$ in methanol. The main peak of 425.0861 / 425.0845 *M*/*Z* (crude/re-crystallized) is assigned to [M^{2+}][H_3CO^-]. Peak at *M*/*Z* ≈ 764.57 corresponds to trace impurity species associated with the equipment, and does not exhibit the characteristic isotopic splitting for antimony.

Table S14. Comparison of calculated *M*/*Z* values for the highest intensity peak corresponding to a given chemical species with corresponding experimental *M*/*Z* values from crude and re-crystallized (re-cryst.) Sb(*p*-Tol)$_3$ in ESI HR-MS (Figure S26).

Chemical assignment	Calculated *M*/*Z*	Experimental *M*/*Z* (crude)	Experimental *M*/*Z* (re-cryst.)
[M][Na^+]	417.05792	417.0570	417.0557
[M^{2+}][H_3CO^-]	425.08654	425.0861	425.0845
[M^{2+}][H_3CO^-]$_2$[Na^+]	479.09741	479.0942	479.0927
[M][$H_3C(CH_2)OH$]$_2$[Na^+]	509.14165	509.1441	509.1418
[M][$O(CH_2CH_3)_2$][H_3CH_2OH][Na^+]	537.17295	537.1754	537.1734

Thermal Analysis

As the discovery and ability to characterize Form II were of critical importance for the investigation of the Sb···Sb interactions, it was also important to conduct a systematic study to establish a clearer understanding of parameters affecting the formation of Form II from the melt. To validate whether thermal history was the sole factor affecting the occurrence of Form II, a new batch of Sb(*p*-Tol)$_3$ was synthesized and divided into three parts, based on the method of purification. Specifically, a "crude Sb(*p*-Tol)$_3$" sample was obtained by simple workup of the as-synthesized material by liquid-liquid extraction using Et_2O and water, followed by drying over anhydrous Na_2SO_4. A fraction of the sample termed "normal Sb(*p*-Tol)$_3$" was obtained by re-crystallization of the crude material from *i*-PrOH, which is the procedure that was applied for most samples used in this work. A sample termed "re-crystallized Sb(*p*-Tol)$_3$" was obtained by additional re-crystallization of the normal sample from hot ethanol (EtOH) and then again from hot hexanes, in a sequence that was aimed to help remove impurities that are both more and less polar than Sb(*p*-Tol)$_3$, respectively. The re-crystallized Sb(*p*-Tol)$_3$ was dried under dynamic vacuum over anhydrous P_4O_{10} (see synthetic procedure methods section, SI page 22 for full purification steps). Finally, two fractions of the re-crystallized Sb(*p*-Tol)$_3$ were then purposely contaminated with either vacuum grease or silicon oil to explore whether these impurities, considered likely to be introduced during synthesis, workup, or heating in a silicon oil bath, could have influenced the occurrence of Form II during thermal cycling. The samples were then analyzed by DSC cycling with heating and cooling rates of 5˚C per minute, with experiments involving re-crystallized Sb(*p*-Tol)$_3$ using 20 cycles because little change in behavior was observed in the first 10 cycles.

The DSC cycling experiments revealed the appearance of Form II for the crude sample (SI Figures S56-S60, S85), with Form II apparently already present at the outset of the experiment in quantities undetectable by initial PXRD analysis, but observable in the first DSC cycle by a low-intensity endotherm at ~91°C, consistent with the melting point of Form II. The DSC thermograms for the re-crystallized sample (Figures S51-55, S83), as well as for the re-crystallized sample in the presence of silicon oil (Figures S61-63, S85), also revealed the appearance of Form II upon thermal cycling, but to a significantly lesser extent compared to the crude (Figures S56-60, S84) or normal sample (Figures S39-S50, S82). For re-crystallized Sb(*p*-Tol)$_3$, the appearance of Form II was typically detected through a weak endo- and/or exothermic signal around 90-95˚C, interpreted either as melting of a small amount of Form II and/or Form II → Form I transformation. Notably, endotherms associated with melting or conversion of Form II were never observed upon thermal cycling of the re-crystallized sample in the presence of added vacuum grease contaminant (SI Figures S64-66, S86). Overall, the appearance of Form II upon thermal cycling was much less pronounced for the purified, re-crystallized sample, and appeared completely suppressed by introducing vacuum grease. While the role of vacuum grease in apparently hindering the appearance of Form II is not clear, these results suggest that the formation of this polymorph can be influenced by impurities. In that respect, long-

chain aliphatic molecules have recently been reported to impact polymorphism of molecular crystals,[54,55] and previous studies on crystallization of paracetamol from an amorphous material have shown that thermal history, external surfaces or the presence of polymer impurities can affect polymorph appearance.[56-58]

Analysis of the cooling segments of the DSC cycling experiments reveals exothermic events that were ascribed to sample crystallization. For re-crystallized Sb(*p*-Tol)$_3$ (SI Figures S51-S55, S83), such exotherms were initially observed around 70°C for most cycles, leading to Form I, as indicated by the melting point temperature measured in the subsequent heating segment. After the 8th cycle, however, the temperature of the exothermic crystallization events gradually shifts lower, down to ca. 30°C, which also coincided with observation of Form II upon subsequent heating. Similar behavior was also observed for the re-crystallized sample in the presence of silicon oil (SI Figures S61-S63, S85). In contrast, the crystallization events for a sample of re-crystallized Sb(*p*-Tol)$_3$ contaminated with vacuum grease, which never exhibited the appearance of Form II, all occurred around 90°C (SI Figures S64-S66, S86). For crude Sb(*p*-Tol)$_3$ (Fig. 5a,b, SI Figures S56-S60, S84), exothermic events ascribed to crystallization of the sample are not observed upon cooling, but only upon heating in the next cycle, around 25°C. This suggests that, in these cases, the material forms a supercooled liquid and/or an amorphous solid upon cooling to 25°C, which then crystallizes upon heating. All described observations are consistent with Form II forming from highly supercooled environments.

In one set of 20 experiments performed at Babeș-Bolyai University (SI Figures S67-69), DSC thermograms were fully consistent with the presence of only Form I of Sb(*p*-Tol)$_3$ during the initial 10 heating and cooling cycles. Form II was, however, observed in later cycles either directly through a strong melting point endotherm around 91°C, or indirectly – through a combination of endothermic and exothermic signals around 90°C that were ascribed to either melting of small amounts of Form II, or the transformation of Form II to Form I. Further reliability of this melting re-crystallization route to Form II was evidenced by a subsequent set of 40 DSC experiments (SI Figures 70-73), where Form II first appeared in the 6th heating-cooling cycle, then next in cycle 17 and almost all subsequent ones. Overall, these DSC experiments indicate that the likelihood of obtaining Form II increases by repeated DSC experiments.

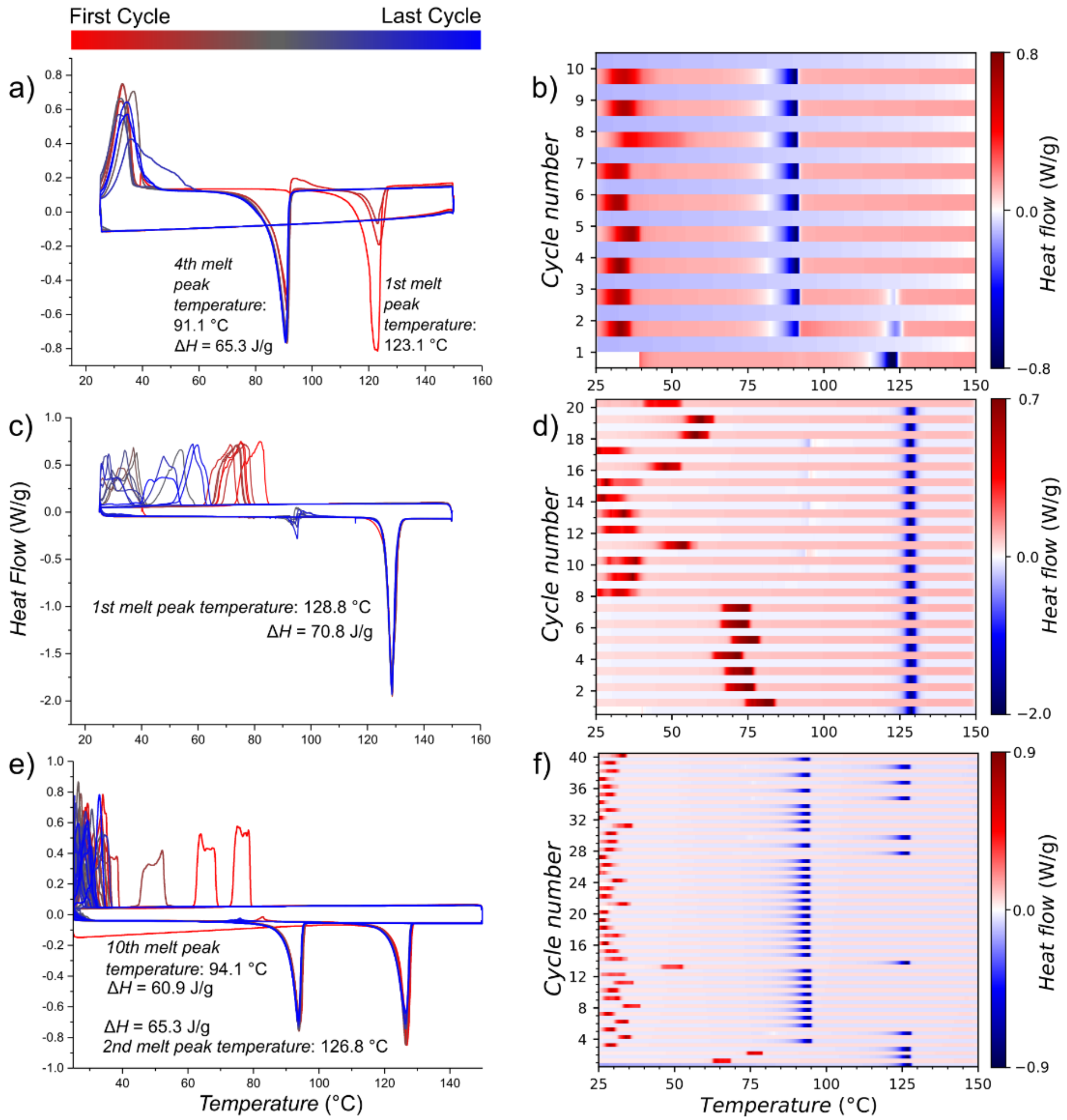

Figure S38. Overview of selected thermograms (left) and corresponding waterfall-style plots (right) for DSC experiments on different Sb(*p*-Tol)$_3$ samples; a) and b) crude Sb(*p*-Tol)$_3$, 10 cycles, 5 ˚C/min; c) and d) re-crystallized Sb(*p*-Tol)$_3$, 20 cycles, 5˚C/min; d) and e) Sb(*p*-Tol)$_3$ synthesized at Babeș-Bolyai University, 40 cycles, 2.5˚C/min. Experiments were performed in the range 25-150°C. Note: the crude Sb(*p*-Tol)$_3$ cycle 1 starts at 40˚C, a precaution against atmospheric moisture adsorbing in the DSC furnace.

Finally, we explored whether changes to the heating/cooling rate, to either 10˚C per minute or 2.5 ˚C per minute, would affect the appearance of Form II (Fig. 5e,f, SI Figures

S74-S81). In the former case, Form II was indirectly detected in cycles 8-12 by a weak transition signal around 90˚C prior to the signal at 126 ˚C assigned to Form I melting. After cycle 15, however, Form II was observable directly in almost all cycles through a clear melting endotherm around 95˚C. When heating at a rate of 2.5˚C per minute, Form II was consistently observed almost each time after the 3rd cycle. Overall, the above outlined experiments show that repeated heating and cooling cycling promotes Form II appearance for at least three heating and cooling rates: 2.5˚C/min, 5˚C/min and 10˚C/min, as well as that the presence of impurities can affect the appearance of Form II. This suggests that the appearance of Form II upon repeated melting and solidification might be promoted by trace impurities generated by degradation of Sb(*p*-Tol)$_3$ during thermal cycling. Alternative explanations might also exist, however, such as persistence of Form I seeds during initial cycling experiments, and a firm conclusion on the role of impurities cannot yet be made.

DSC thermal cycling (10 cycles, 25-150°C, 5°C/min) for normal Sb(*p*-Tol)$_3$

A sample of normal Form I Sb(*p*-Tol)$_3$ (2.78 mg) was placed in a hermetically sealed 40 µL Tzero Alumina Hermetic DSC pan, and subjected to 10 heating and 10 cooling cycles at a heating/cooling rate of 5˚C/min, with each temperature ramp separated by 1-minute isotherm. The initial cycle started at 25 ˚C and all experiments were performed in a nitrogen flow (25 mL/min). Enthalpies are not calculated for cycles indicating presence of both Form I and II.

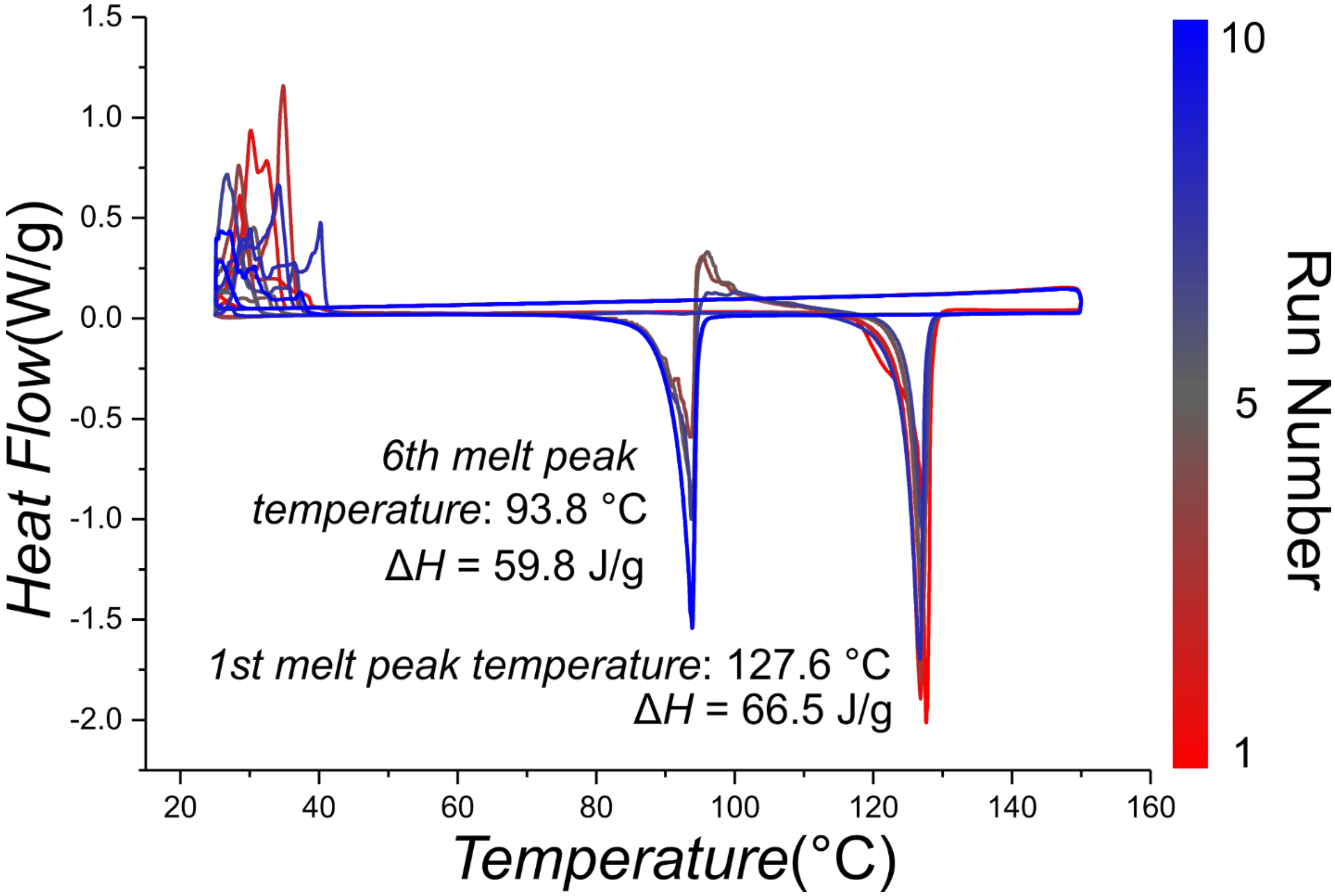

Figure S39. Overlay of thermograms for all ten DSC cycles on normal Sb(*p*-Tol)$_3$, with the most endothermic signal of each phase analysed.

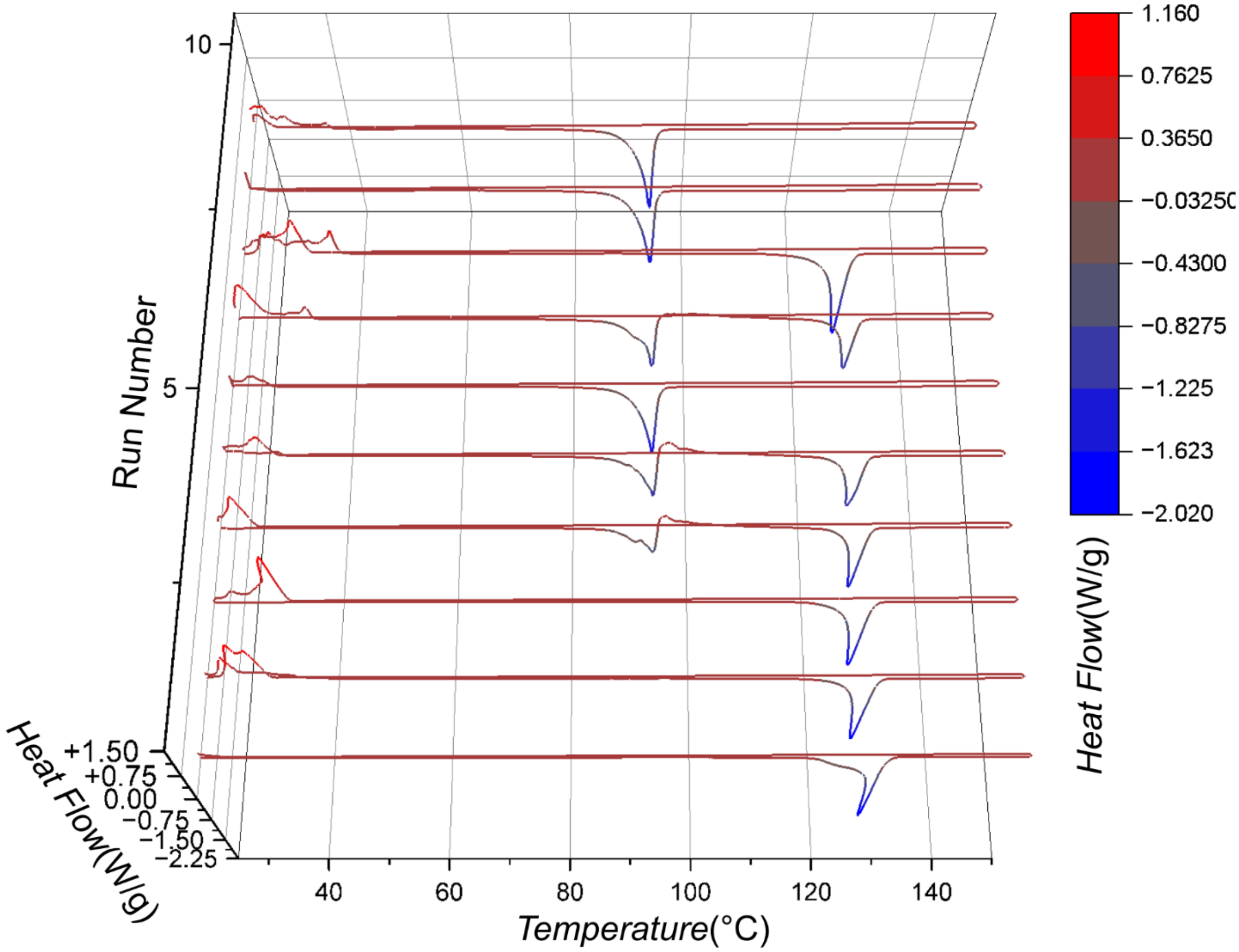

Figure S40. 3D plot of all 10 thermograms on normal Sb(*p*-Tol)$_3$ (Figure S39) in ascending run order front-to-back corresponding to Figure S39. A color gradient is applied to thermograms, spanning from red to grey to blue with respect to heat flow value for additional visual contrast between exo- and endothermic signals.

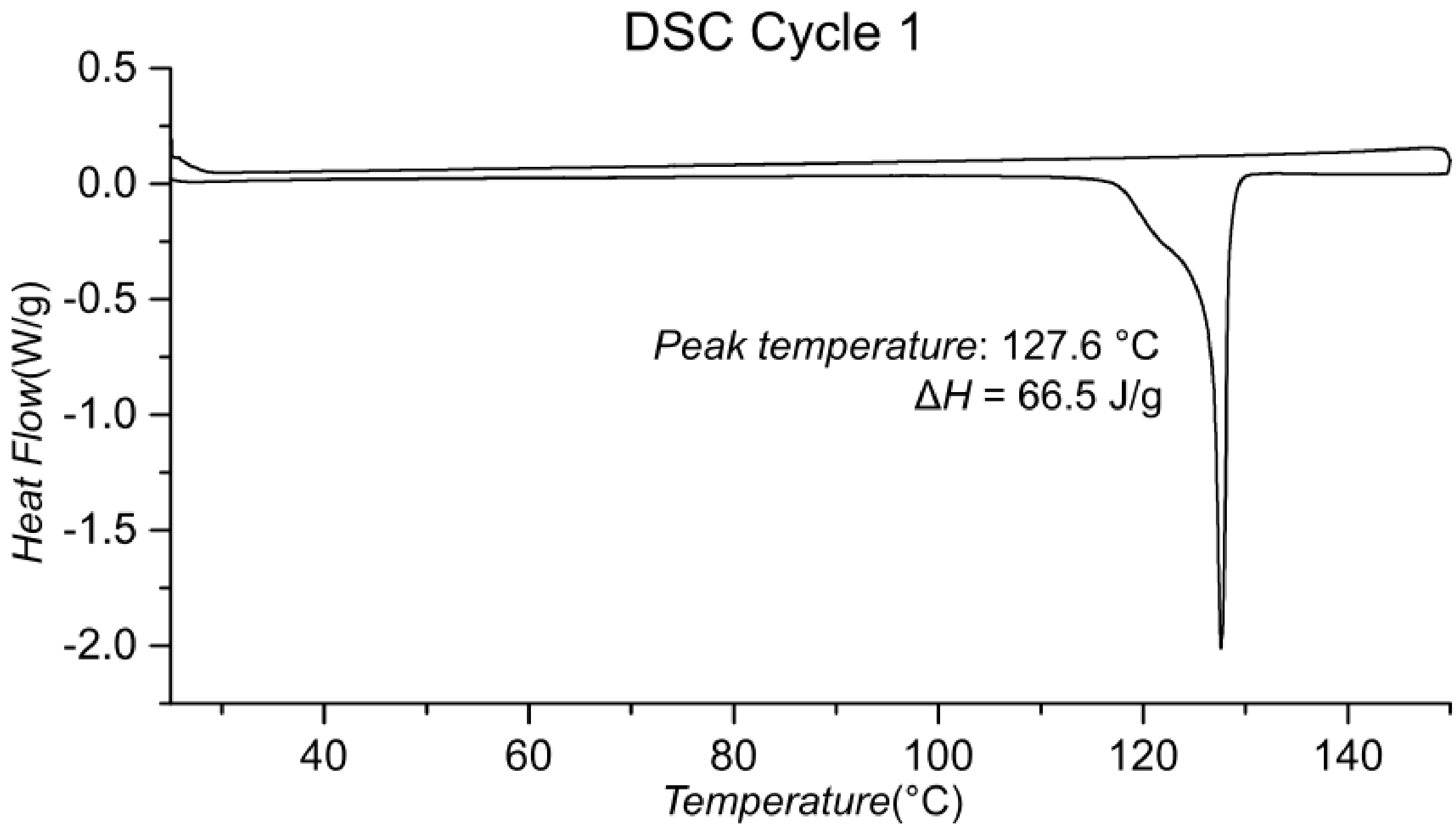

Figure S41. Thermogram for the 1st out of 10 DSC heating/cooling cycles for normal Sb(p-Tol)$_3$ (Figure S39). A minimum observed at 127.6°C, with an onset at 126.6°C. The shoulder peak observed has an onset of 117.7°C.

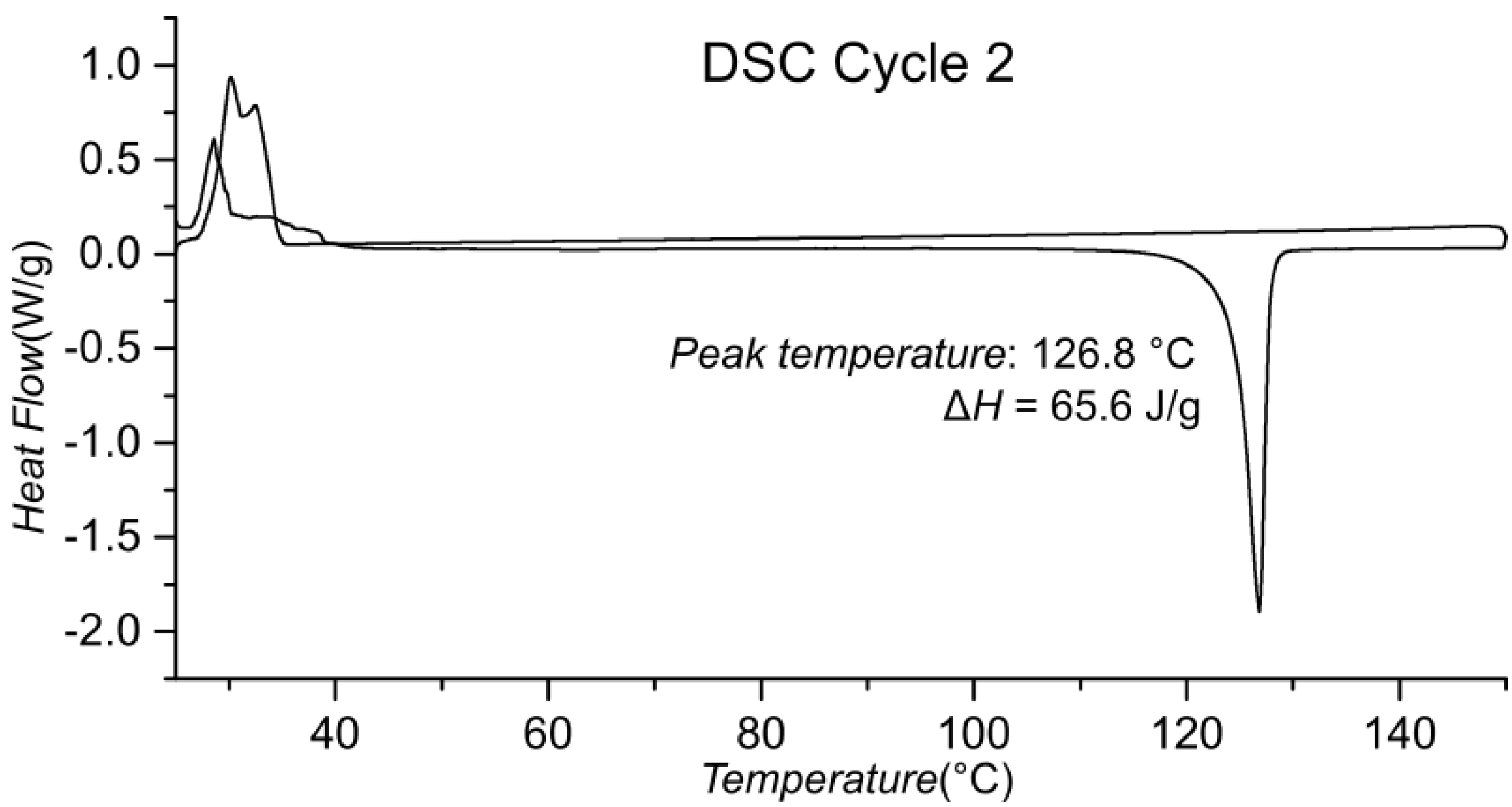

Figure S42. Thermogram for the 2nd out of 10 DSC heating/cooling cycles for normal Sb(p-Tol)$_3$ (Figure S39). A minimum is present at 126.8°C with an onset of 124.5°C

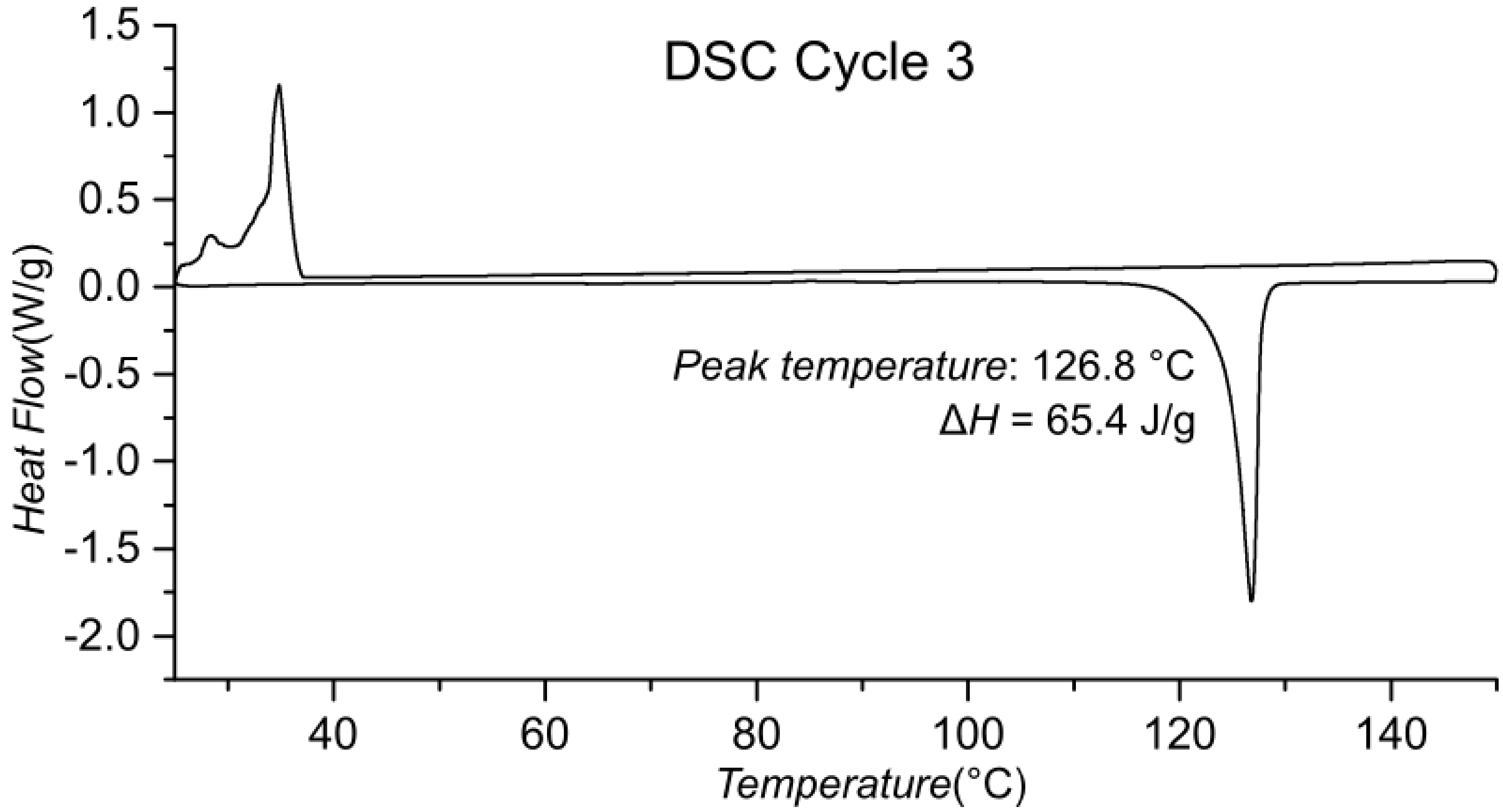

Figure S43. Thermogram for the 3rd out of 10 DSC heating/cooling cycles for normal Sb(*p*-Tol)$_3$ (Figure S39). A minimum is observed at 126.8°C with an onset of 124.3°C.

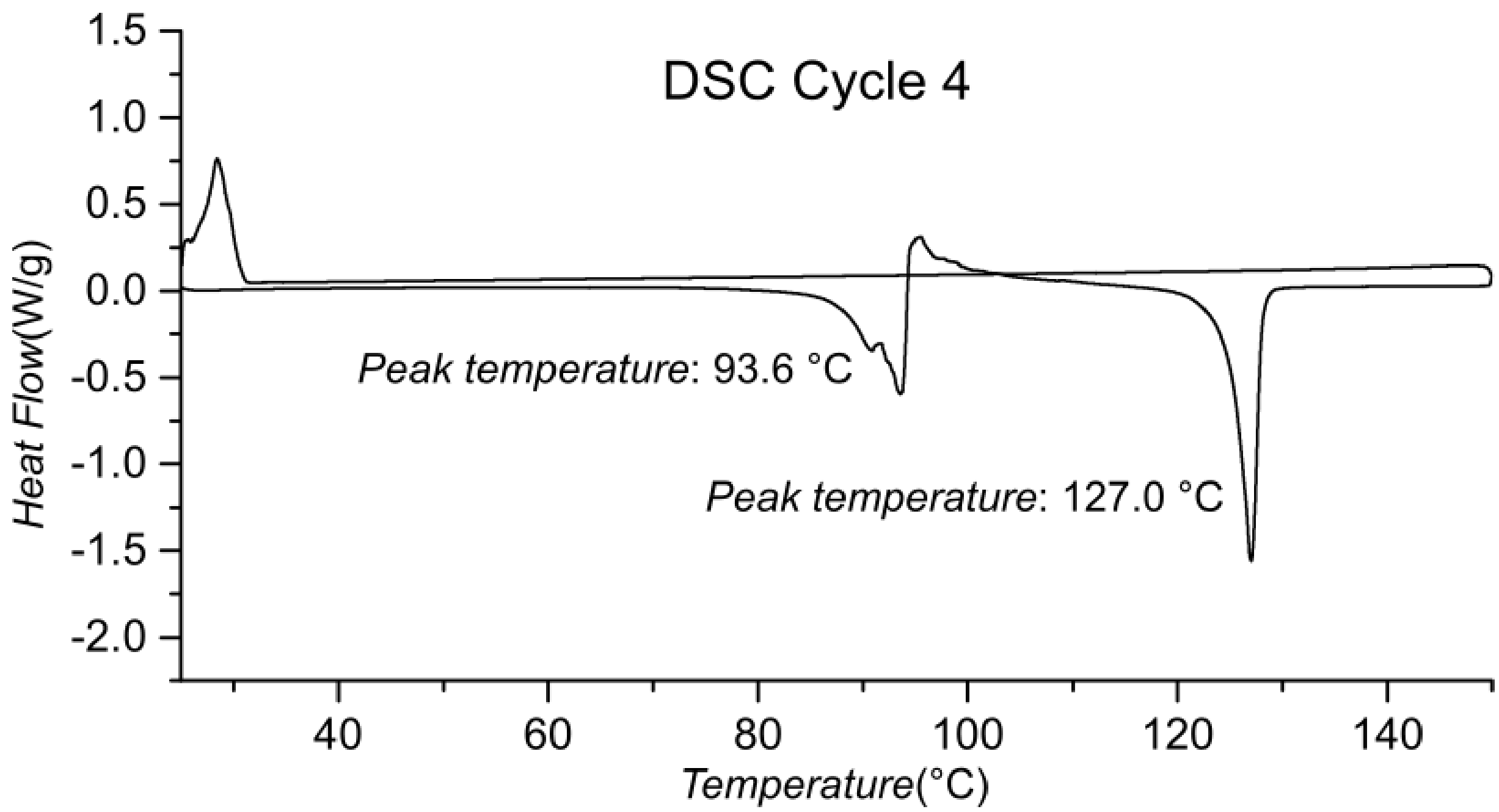

Figure S44. Thermogram for the 4th out of 10 DSC heating/cooling cycles for normal Sb(*p*-Tol)$_3$ (Figure S39). Minima are present at 93.6°C and 127.0°C with onsets of 91.3°C and 125.3°C, respectively.

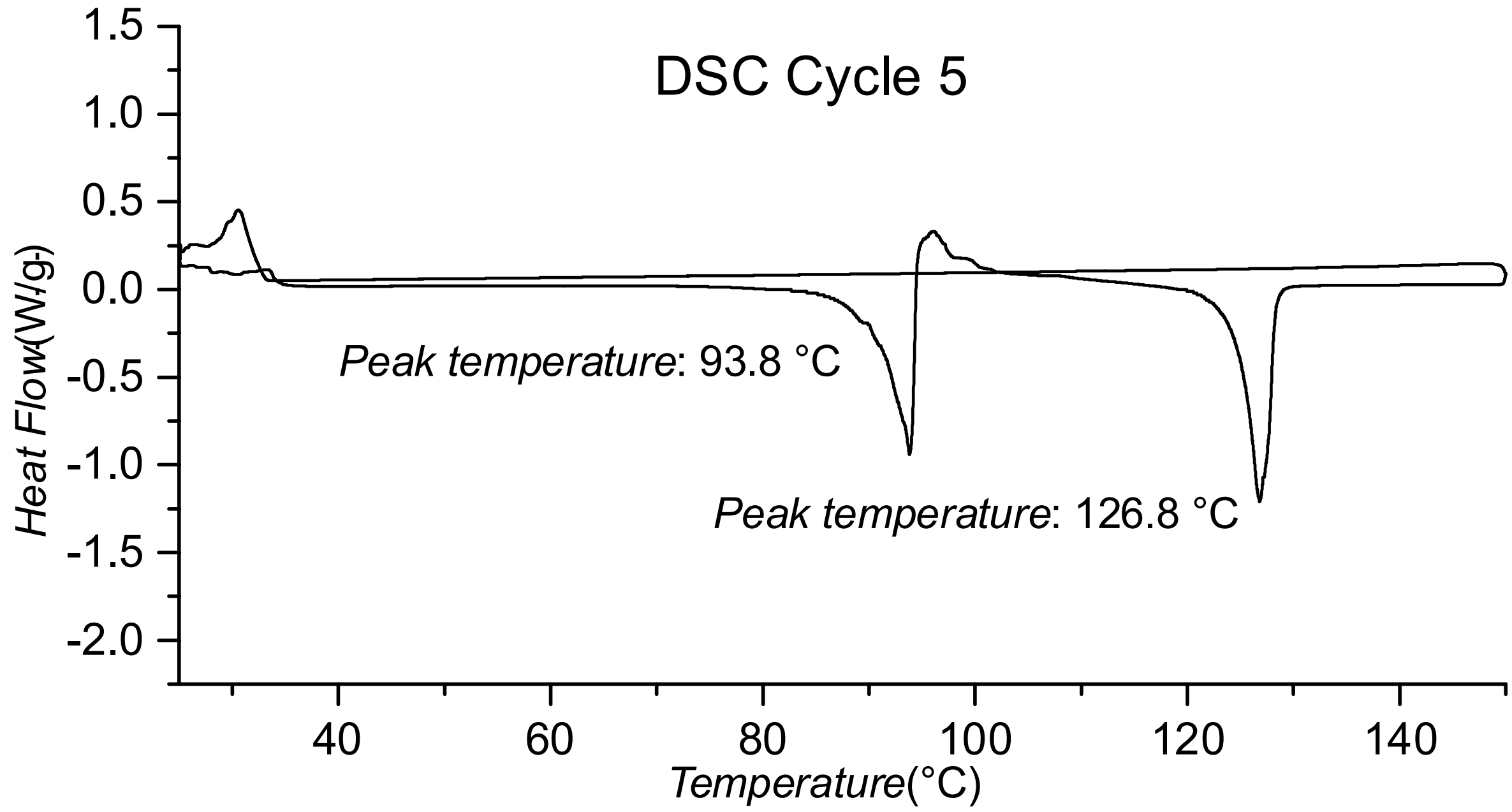

Figure S45. Thermogram for the 5th out of 10 DSC heating/cooling cycles for normal $Sb(p\text{-}Tol)_3$ (Figure S39). Minima are present at 93.8°C and 126.8°C with onsets of 91.7°C and 124.9°C, respectively.

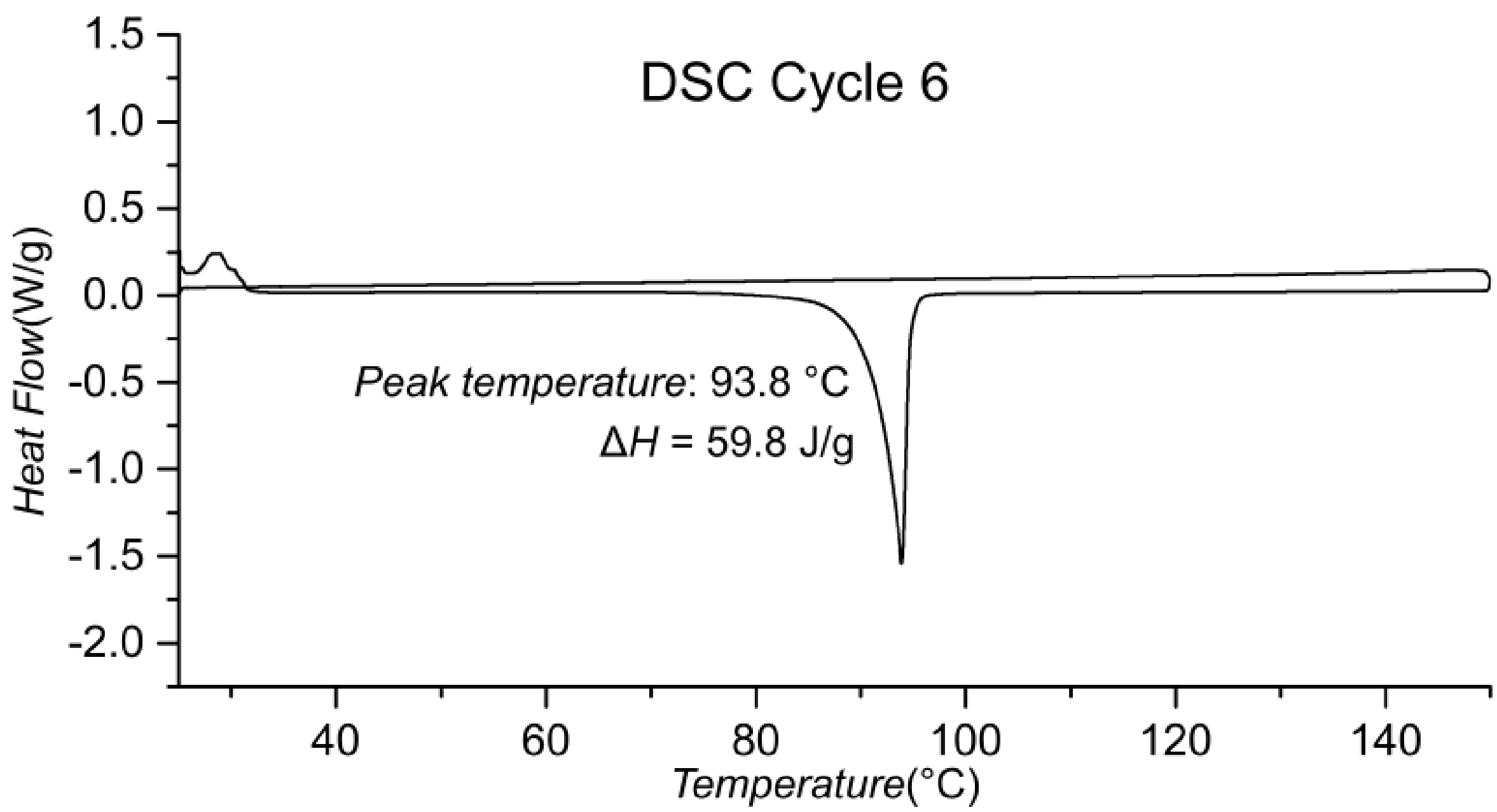

Figure S46. Thermogram for the 6th out of 10 DSC heating/cooling cycles for normal $Sb(p\text{-}Tol)_3$ (Figure S39). A minimum is observed at 93.8°C with an onset of 91.7°C.

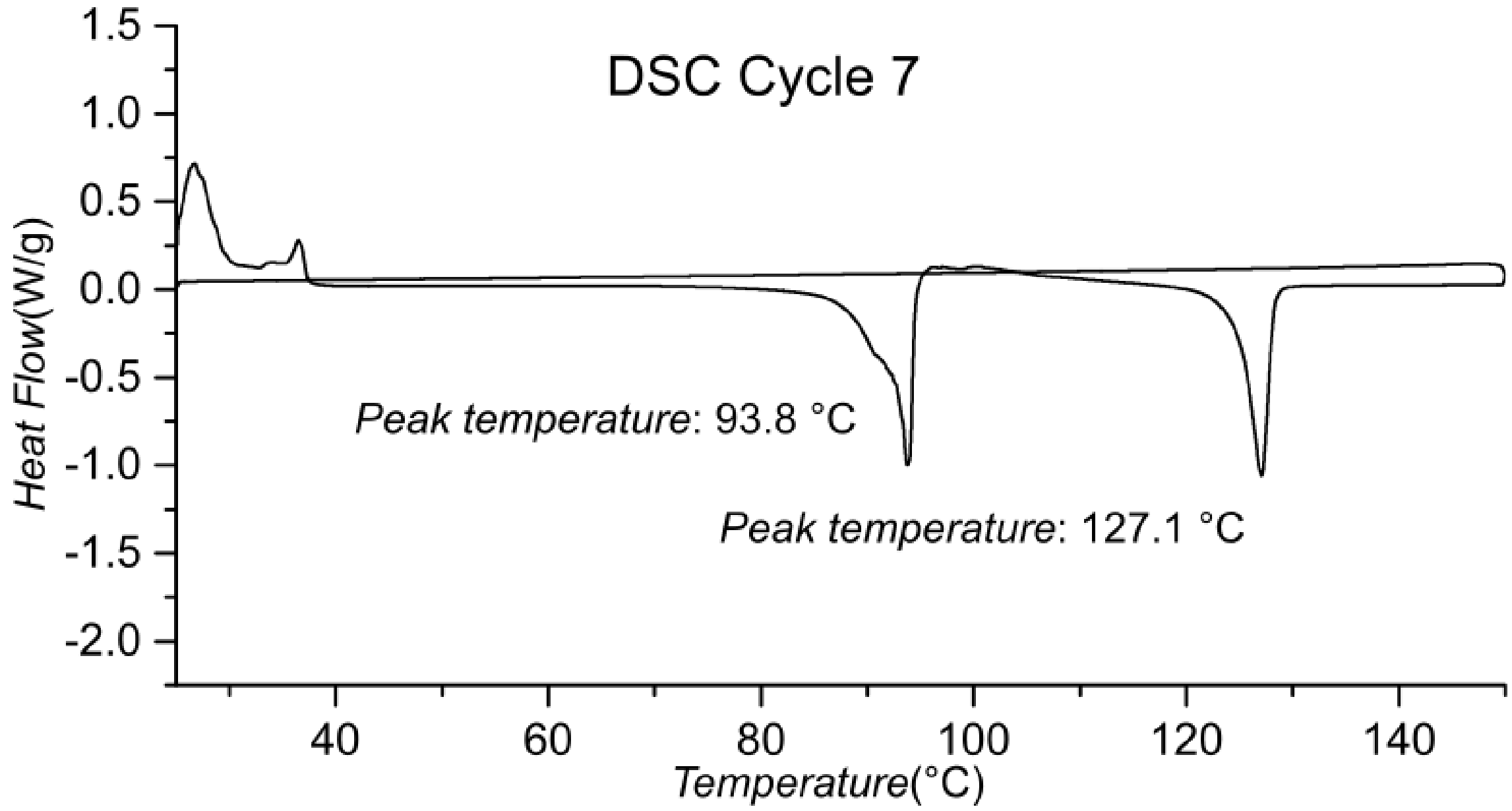

Figure S47. Thermogram for the 7th out of 10 DSC heating/cooling cycles for normal $Sb(p\text{-}Tol)_3$ (Figure S39). Minima are present at 93.8°C and 127.1°C with onsets of 92.5°C and 124.9°C, respectively.

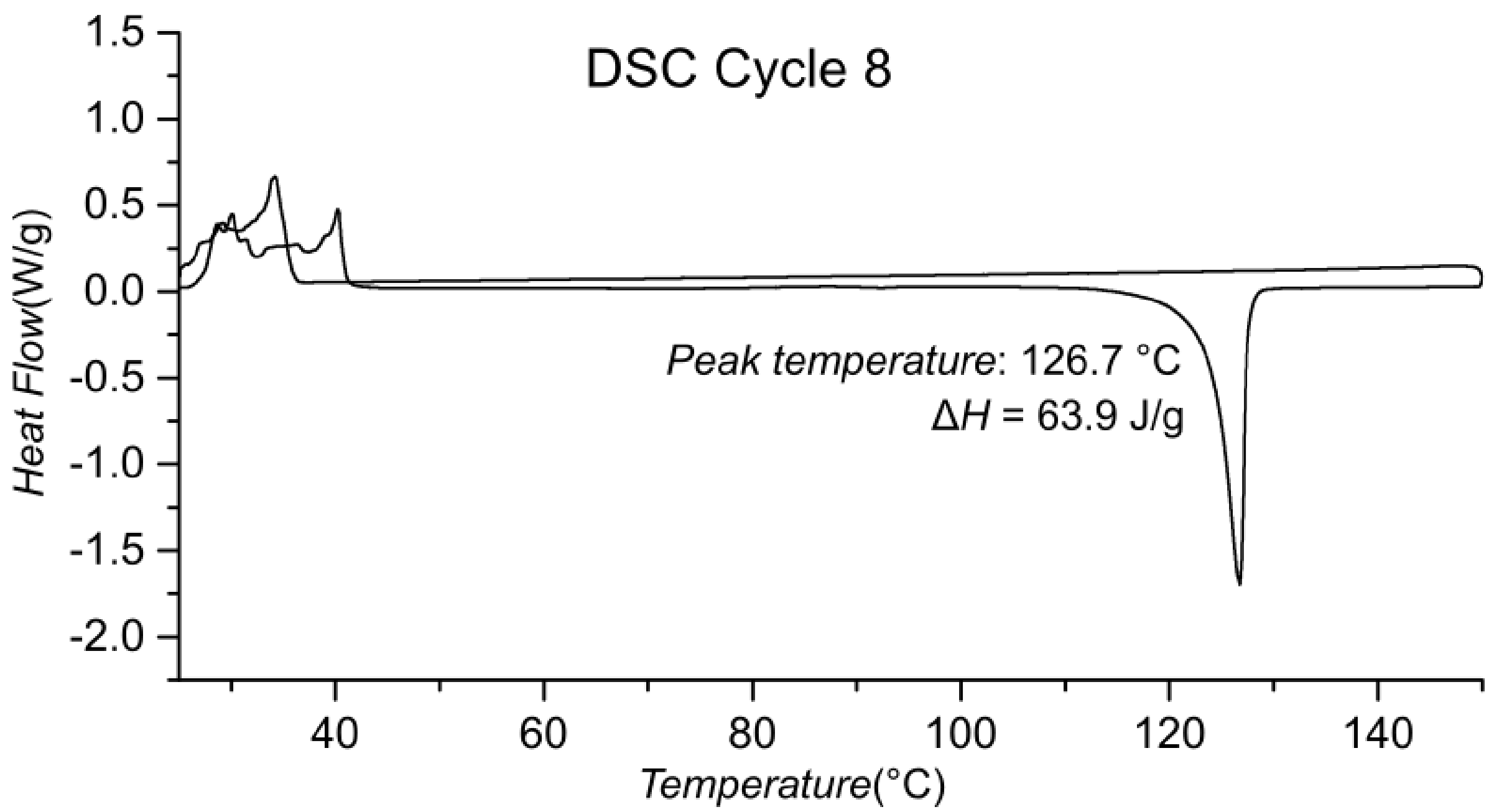

Figure S48. Thermogram for the 8th out of 10 DSC heating/cooling cycles for normal $Sb(p\text{-}Tol)_3$ (Figure S39). A minimum is observed at 126.7°C with an onset of 124.0°C.

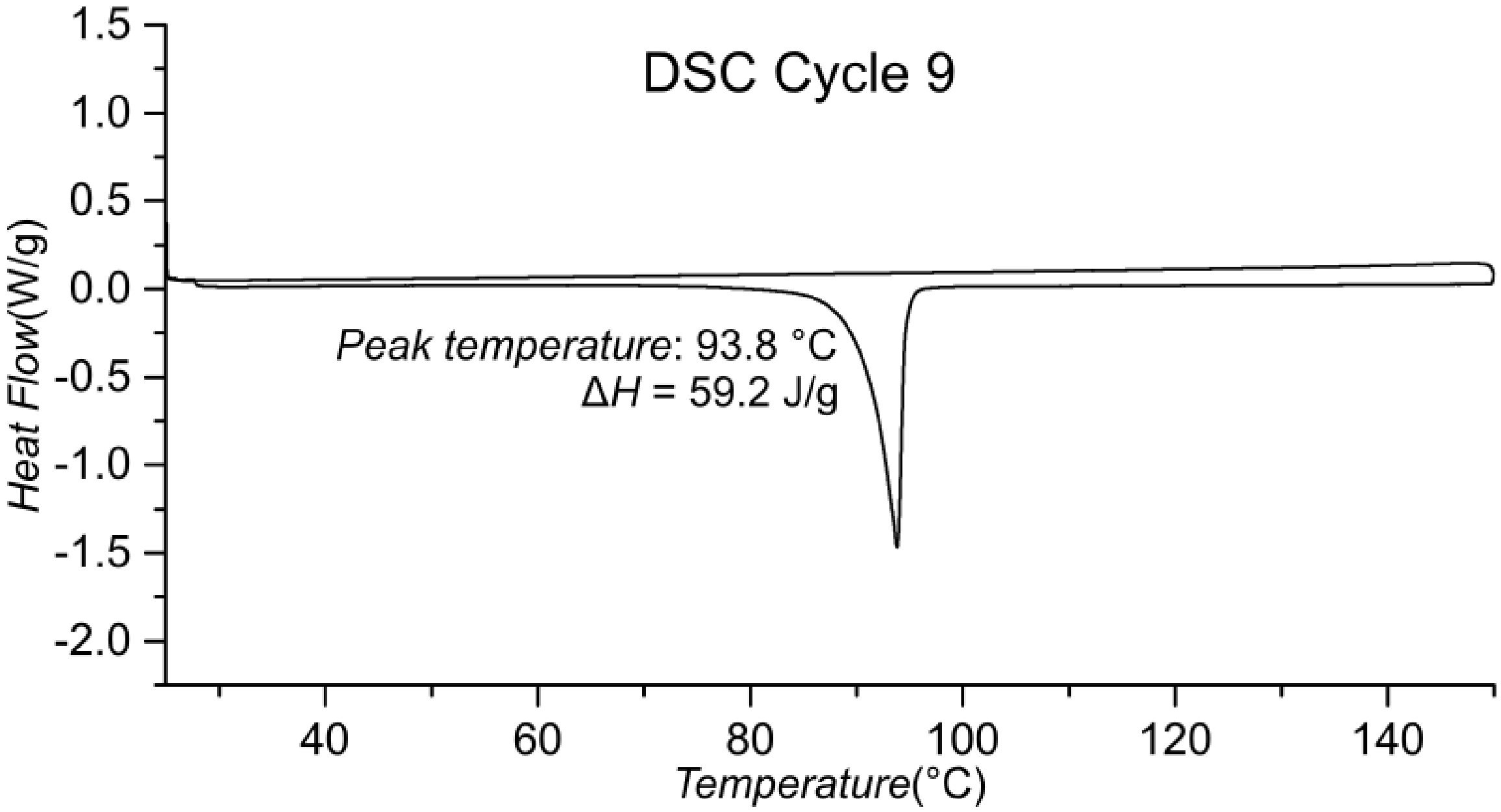

Figure S49. Thermogram for the 9th out of 10 DSC heating/cooling cycles for normal Sb(*p*-Tol)$_3$ (Figure S39). A minimum is observed at 93.8°C with an onset of 91.1°C.

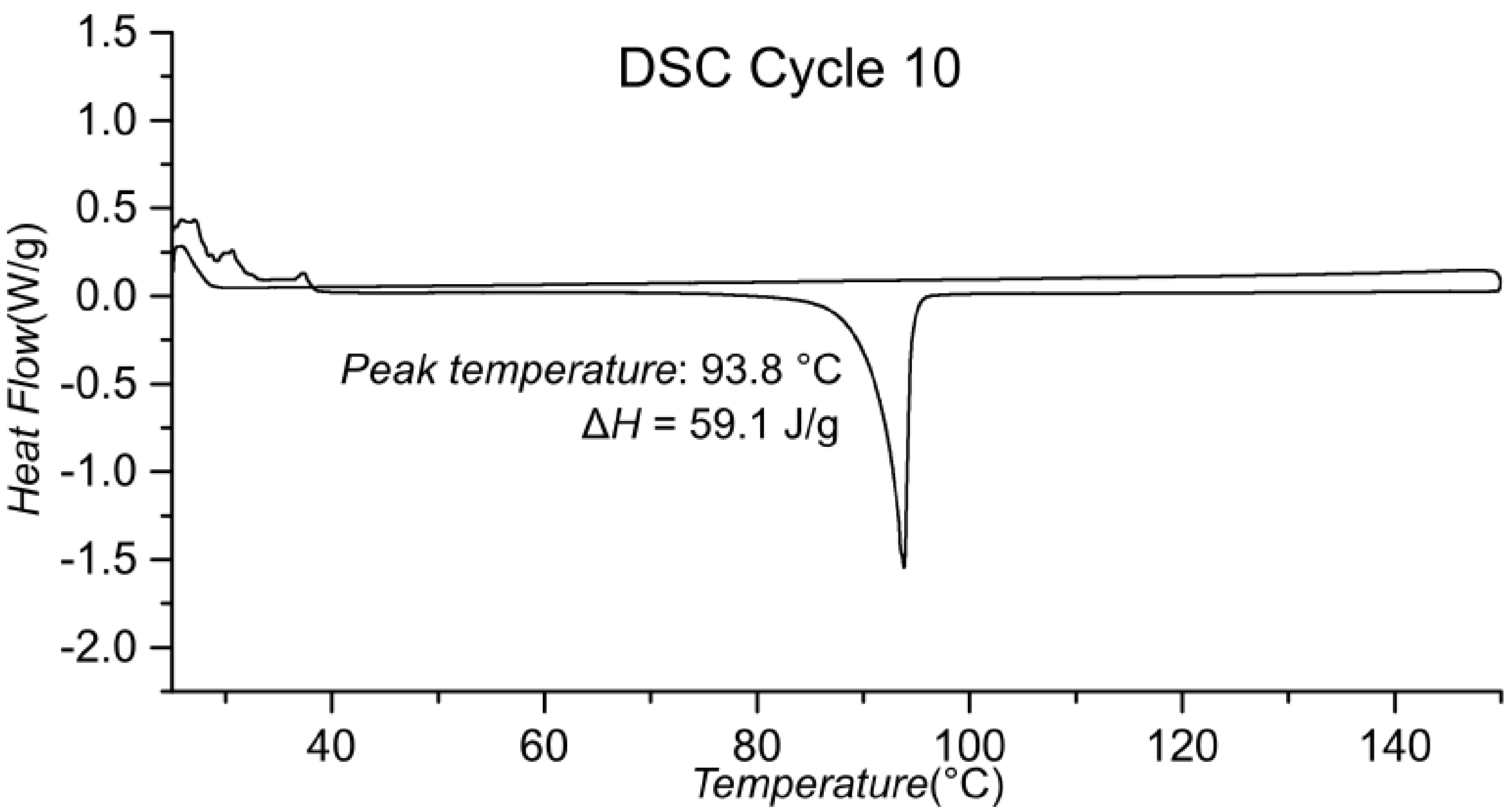

Figure S50. Thermogram for the 10th out of 10 DSC heating/cooling cycles for normal Sb(*p*-Tol)$_3$ (Figure S39). A minimum is observed at 93.8°C with an onset of 91.1°C.

DSC thermal cycling (20 cycles, 25-150°C, 5°C/min) for re-crystallized Sb(*p*-Tol)$_3$

A sample of re-crystallized Form I Sb(*p*-Tol)$_3$ (10.19 mg) of was placed in a hermetically sealed 40 µL Tzero Alumina Hermetic DSC pan and subjected to 20 heating and 20 cooling cycles at a heating/cooling rate of 5˚C/min were used, with each temperature ramp separated by 1-minute isotherm. The initial cycle started at 40˚C and all experiments were performed in a nitrogen flow (25 mL/min). Enthalpies are not calculated for cycles possessing presence of both Form I and II since these would not provide meaningful energy data. Runs selected as noteworthy are shown separately.

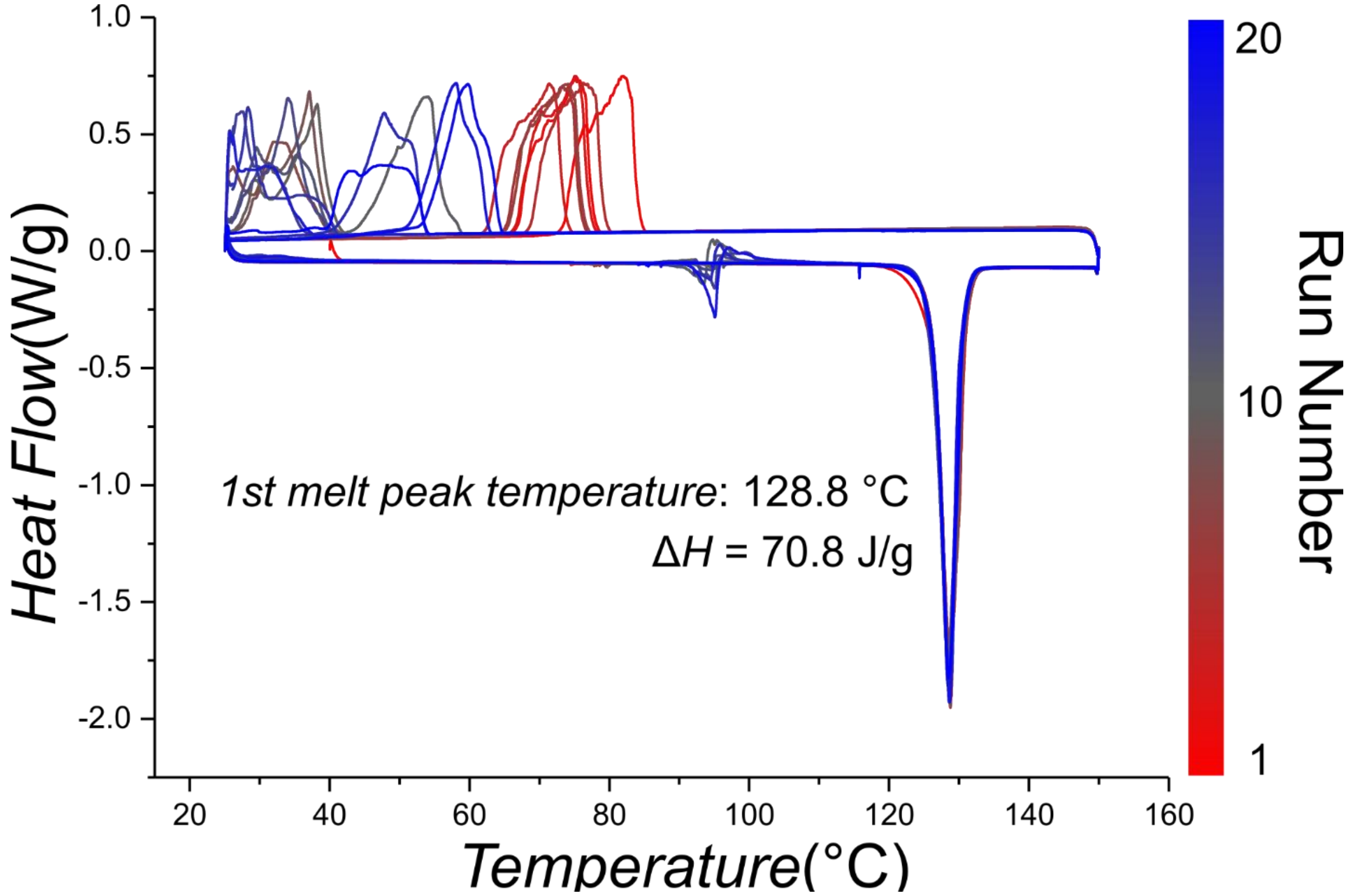

Figure S51. Overlay of all 20 DSC cycles for re-crystallized Sb(*p*-Tol)$_3$, with a gradient colour scheme from red to blue. Temperature and enthalpy for the first and most endothermic melting event are shown. Non-negligible Form II melting endotherms are observed on runs 10-11, 13-14, 16 and 18, but phase-pure Form II was not observed.

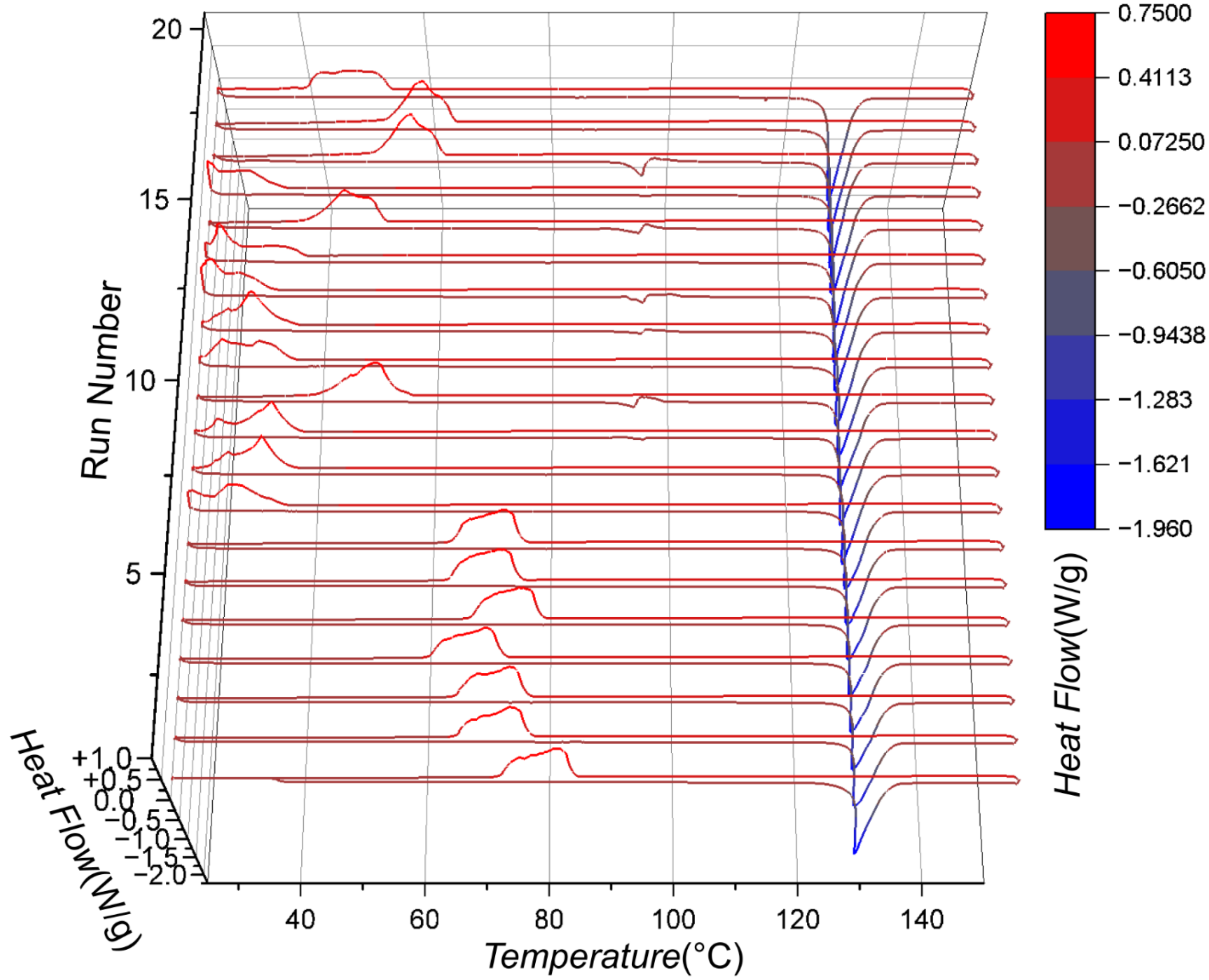

Figure S52. 3D plot of all 20 thermograms on re-crystallized Sb(*p*-Tol)$_3$ (Figure S51.) in ascending run order front-to-back. A color gradient is applied to thermograms, spanning from red to grey to blue with respect to heat flow value for additional visual contrast between exo- and endothermic signals.

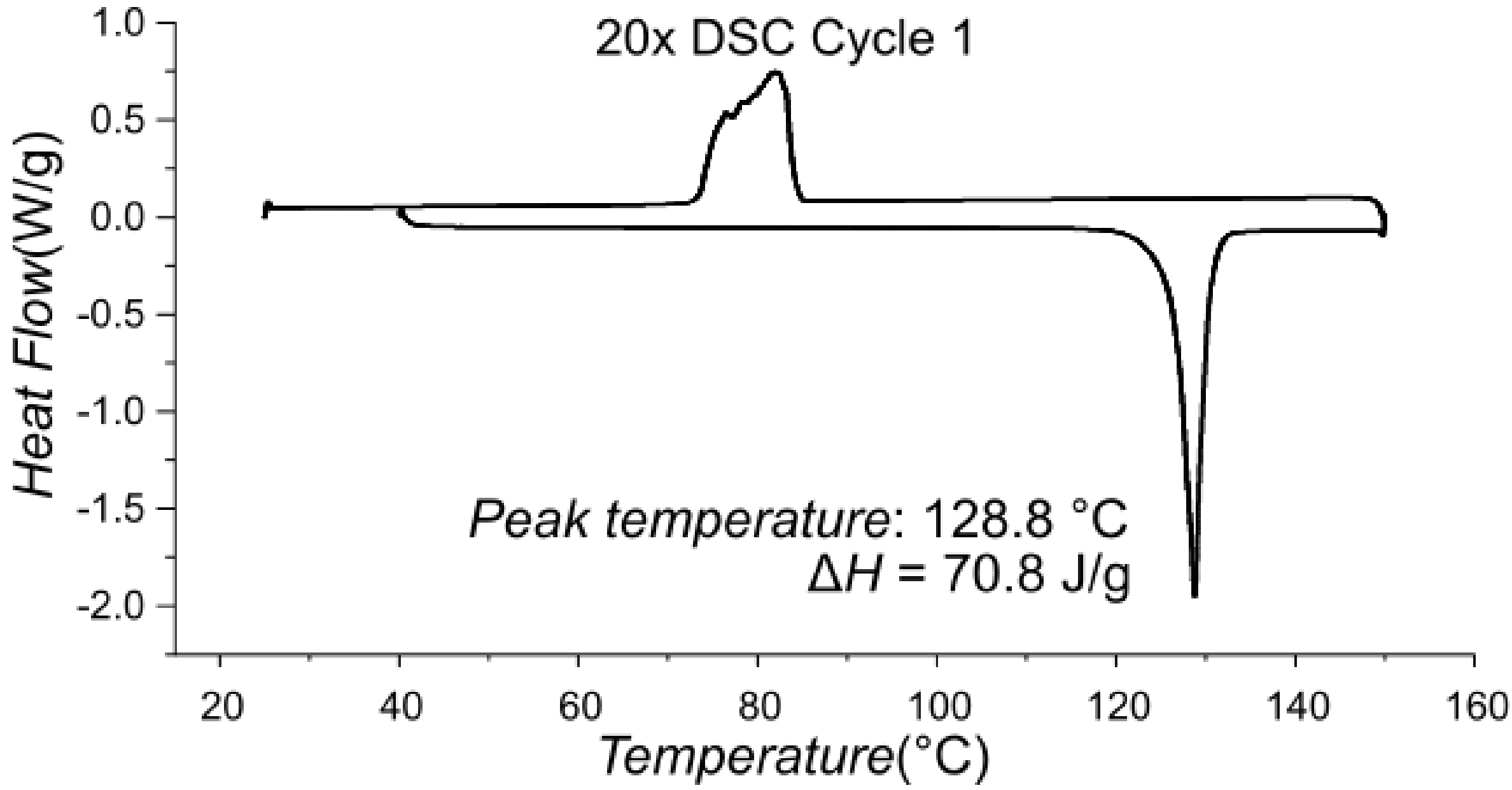

Figure S53. Thermogram for the first out of 20 DSC heating/cooling cycles for re-crystallized Sb(*p*-Tol)$_3$ (Figure S51). This example is the most well defined and endothermic melt for Form I observed. A minimum is observed at 128.8˚C, with an onset of 126.4˚C.

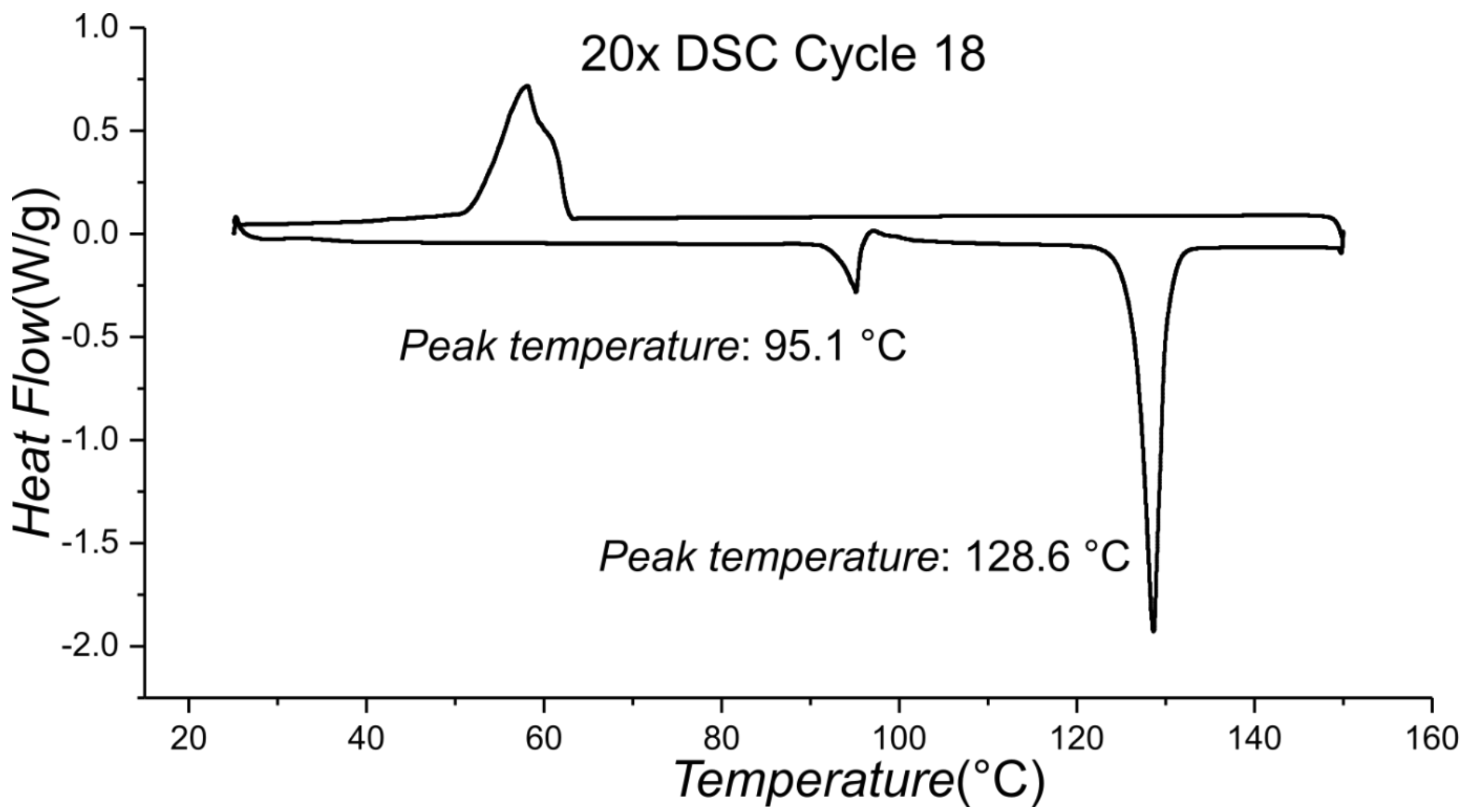

Figure S54. Thermogram for the 18th out of 20 DSC heating/cooling cycles for re-crystallized Sb(*p*-Tol)$_3$ (Figure S51). This run shows the highest peak area for Form II. Minima are observed at 95.1˚C and 128.6˚C with onsets of 95.6˚C and 126.4˚C respectively.

DSC thermal cycling (20 cycles, 25-150°C, 5°C/min) for re-crystallized Sb(*p*-Tol)$_3$: individual experiments

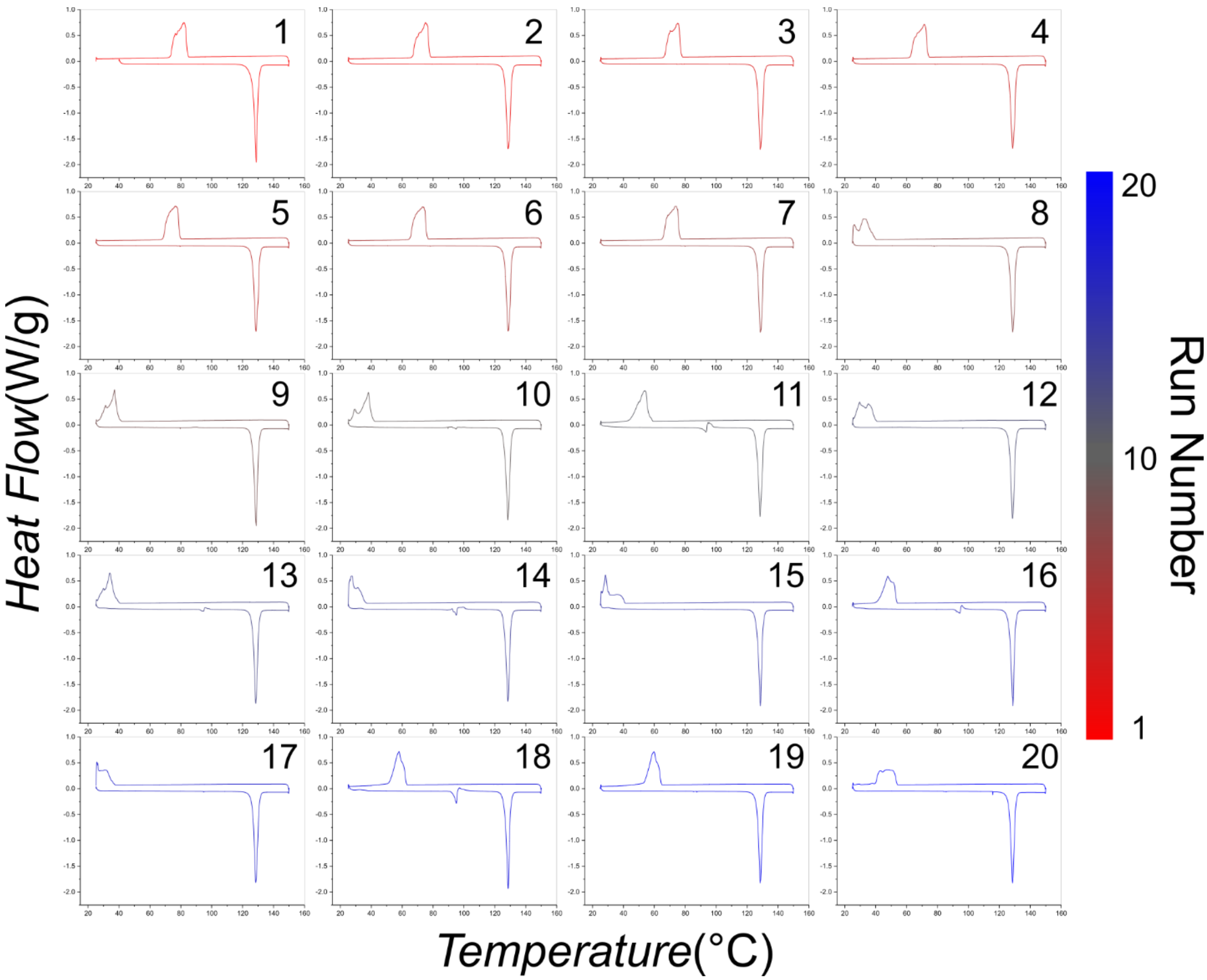

Figure S55. Grid of all thermograms from a set of 20 DSC heating-cooling cycles on re-crystallized Sb(*p*-Tol)$_3$ (Figure S51). Color scheme associated with run number is identical to the associated overlaid figure.

DSC thermal cycling (10 cycles, 25-150°C, 5°C/min) for crude Sb(*p*-Tol)$_3$: overlay

A sample of crude Sb(*p*-Tol)$_3$ (3.47 mg) was placed in a hermetically sealed 40 µL Tzero Alumina Hermetic DSC pan and subjected to 10 heating and 10 cooling cycles at a heating/cooling rate of 5˚C/min, with each temperature ramp separated by 1-minute isotherm. The initial cycle started at 40˚C and all experiments were performed in a nitrogen flow (25 mL/min). Form II is detectable in each cycle, with a weak endotherm in the first cycle suggesting the presence of small amounts of Form II, not detected by PXRD, prior to the DSC experiment.

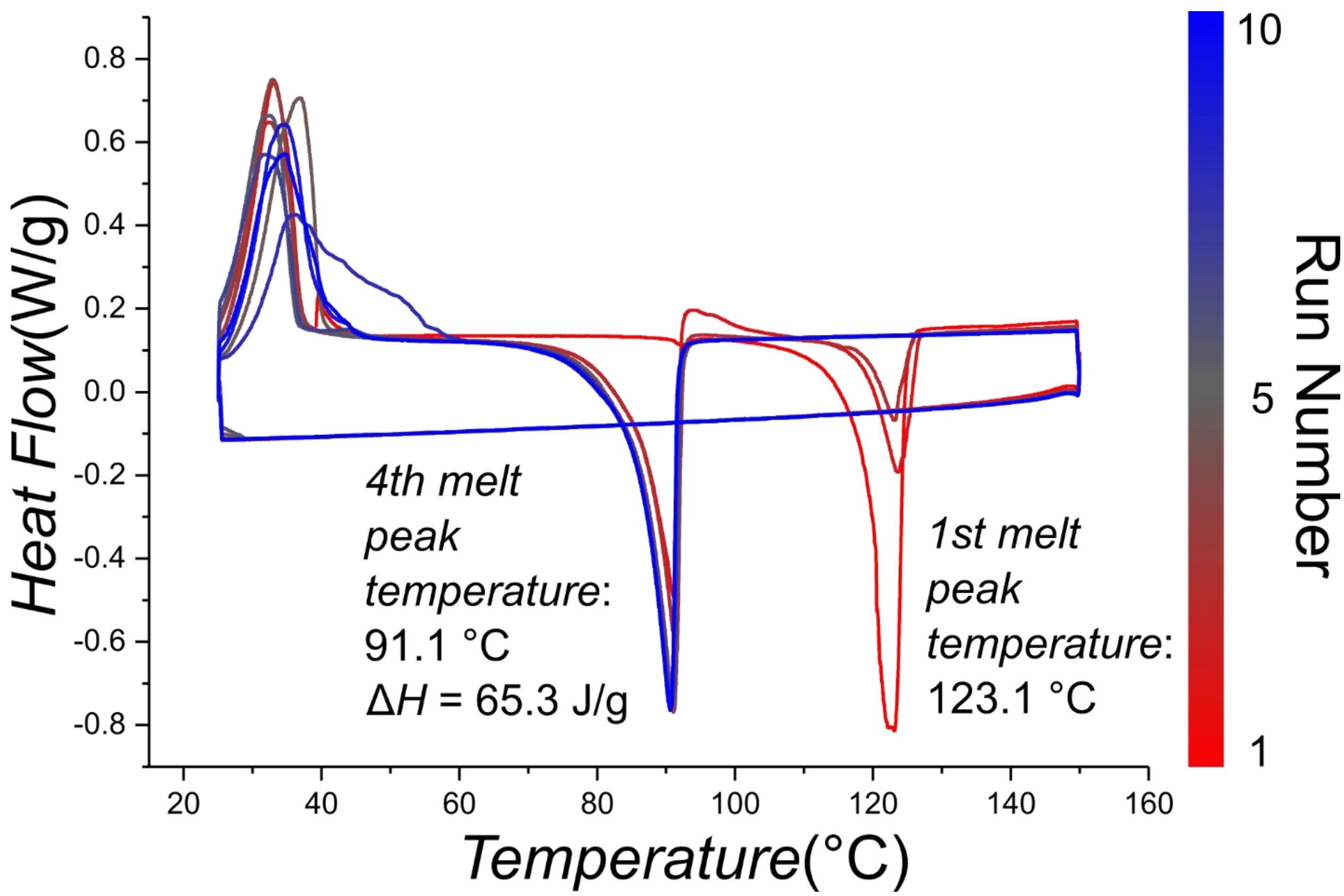

Figure S56. Overlay of all 10 DSC cycles performed on crude Sb(*p*-Tol)$_3$, with a gradient colour scheme from red to blue. Calculated melting temperature and enthalpy are shown for run 4, wherein phase pure Form II melting is observed for the first time in this experiment.

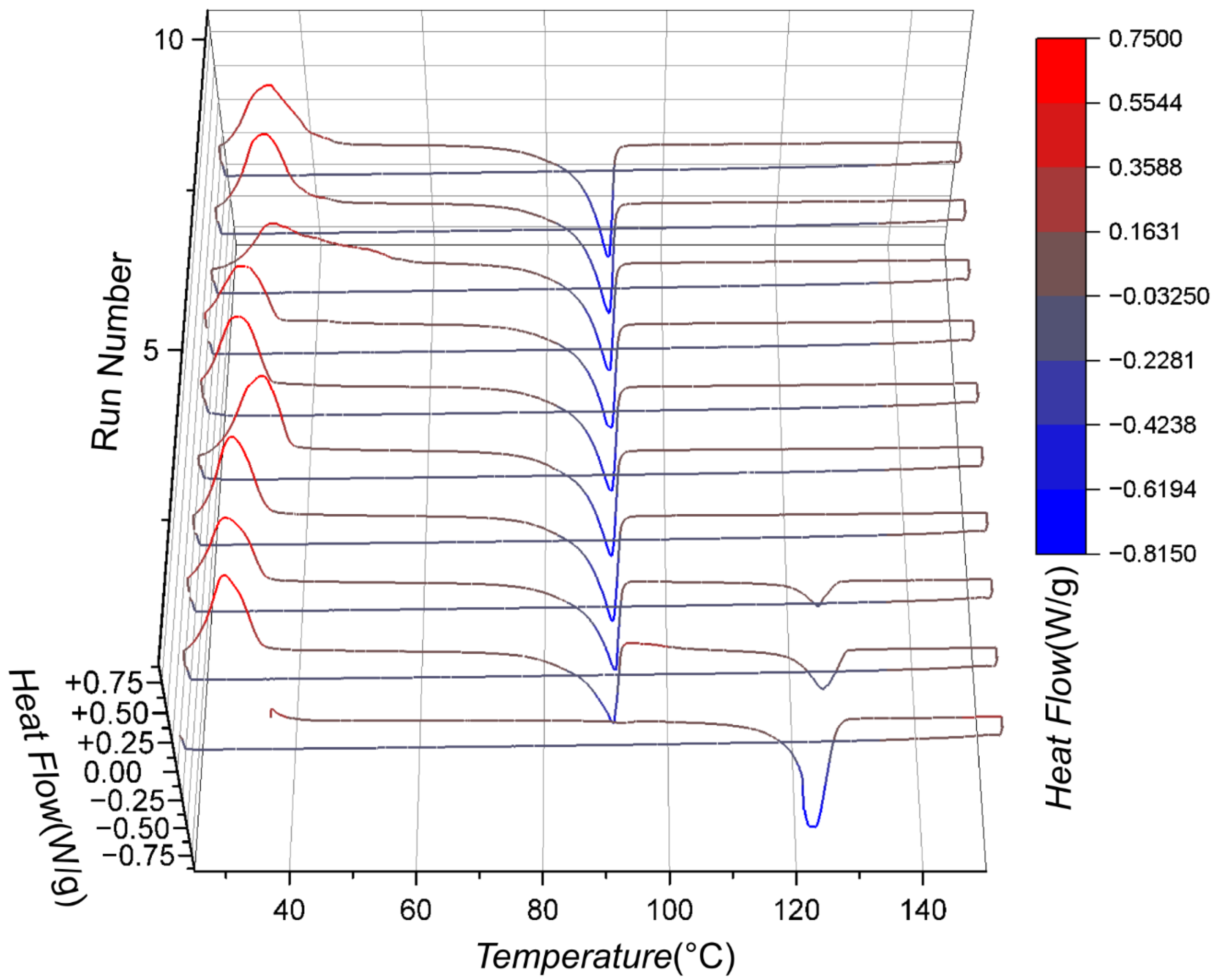

Figure S57. 3D plot of all 10 thermograms on crude Sb(*p*-Tol)$_3$ (Figure S56) in ascending run order front-to-back. A color gradient is applied to thermograms, spanning from red to grey to blue with respect to heat flow value for additional visual contrast between exo- and endothermic signals.

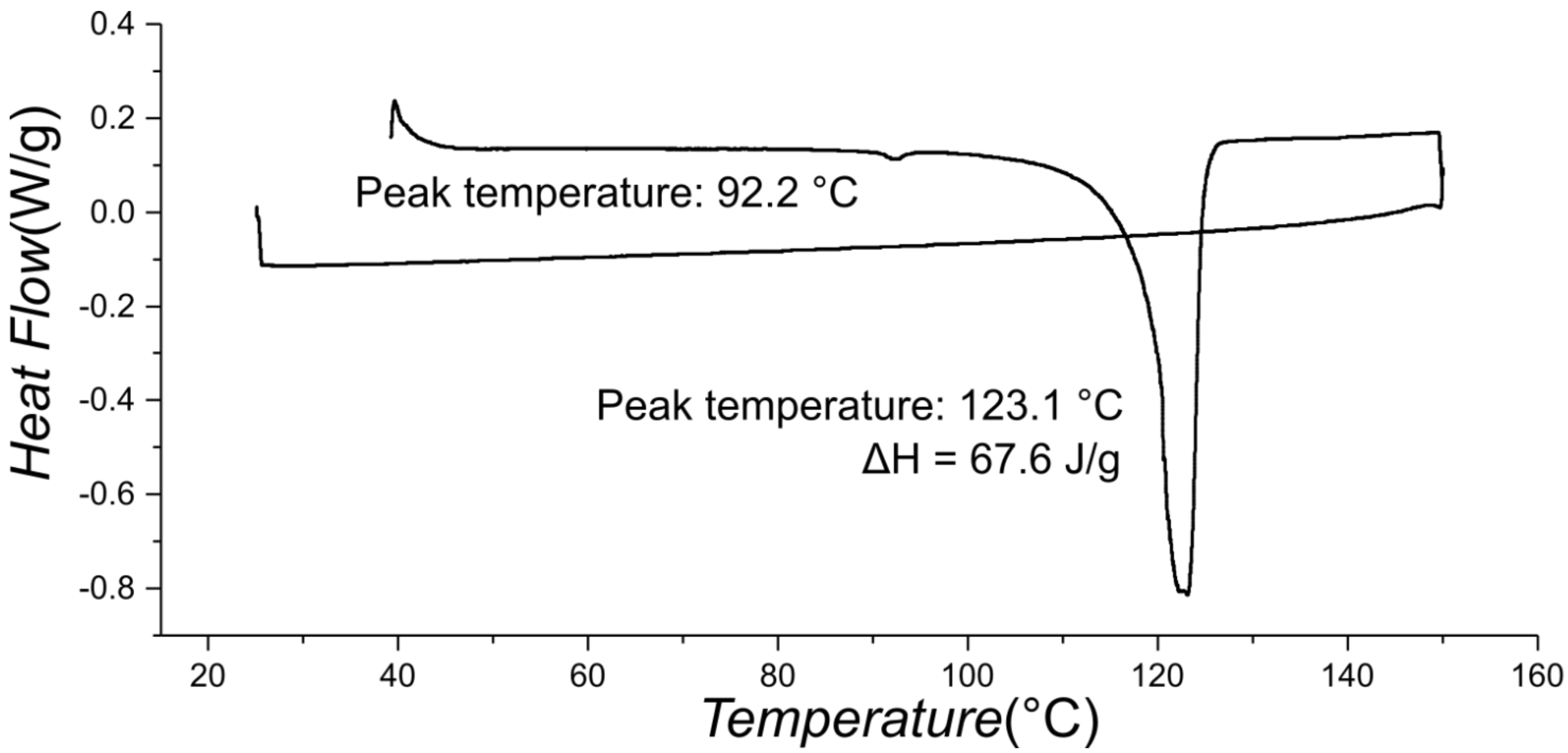

Figure S58. Thermogram for the first out of 10 DSC heating/cooling cycles for crude Sb(*p*-Tol)$_3$ (Figure S56). Minima are observed at 92.2˚C and 123.1˚C with onset points of 90.8˚C and 119.9˚C respectively.

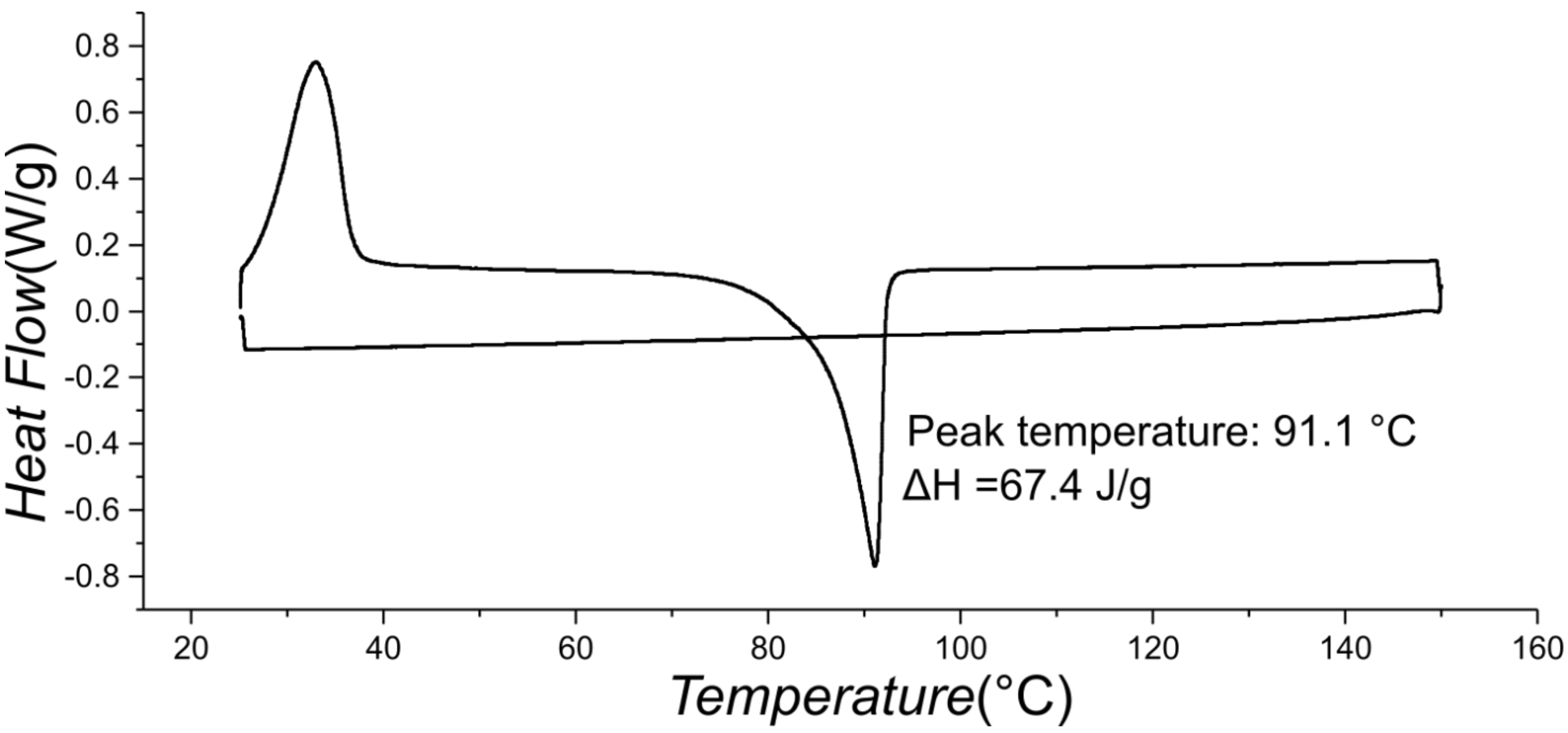

Figure S59. Thermogram for the 4th out of 10 DSC heating/cooling cycles for crude Sb(*p*-Tol)$_3$ (Figure S56). Only Form II is seen. A minimum is observed at 91.1˚C with an onset point of 86.0˚C.

DSC thermal cycling (10 cycles, 25-150°C, 5°C/min) for crude Sb(*p*-Tol)$_3$: individual runs

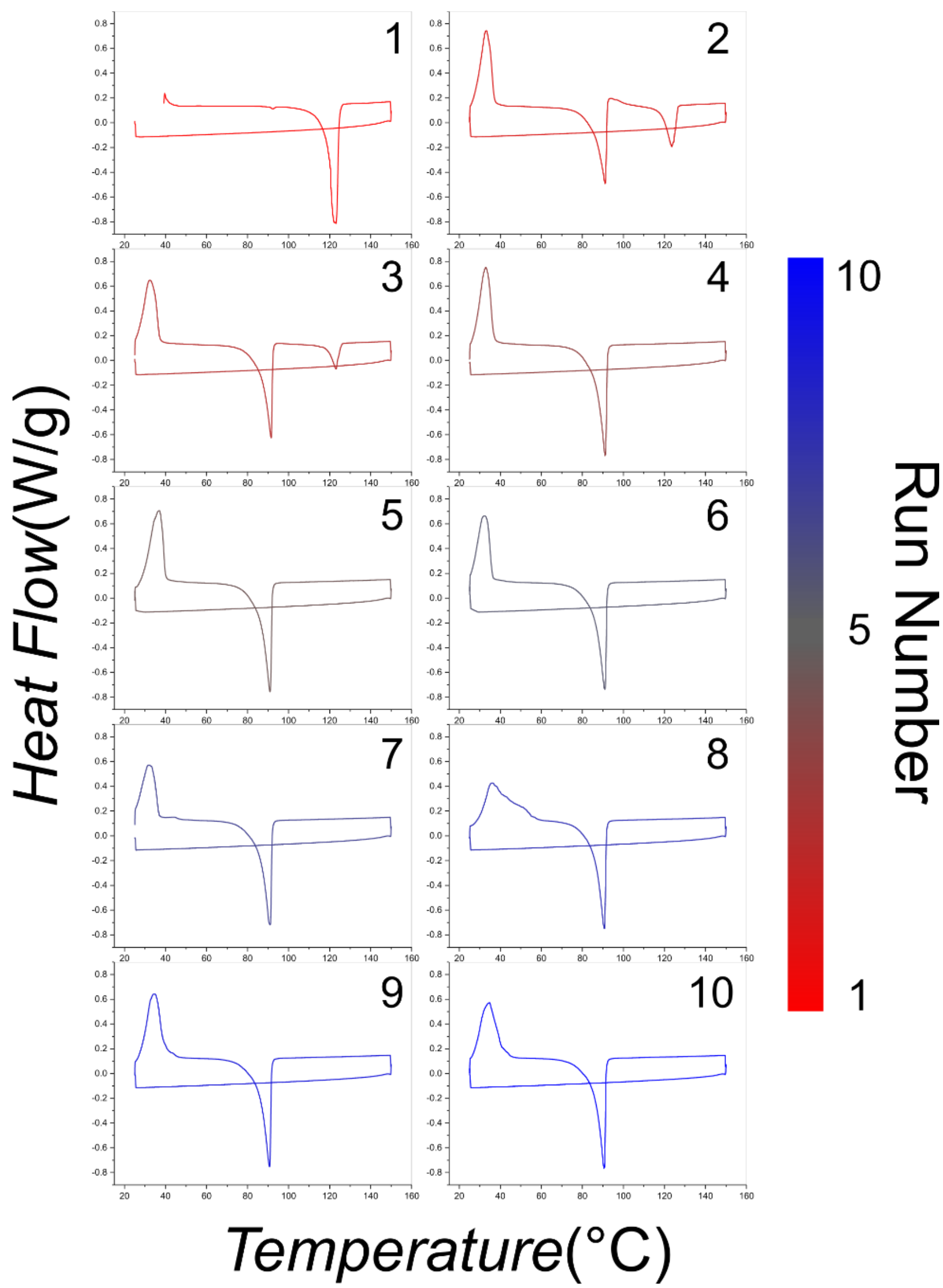

Figure S60. Grid of all thermograms from a set of 10 DSC heating-cooling cycles on crude Sb(*p*-Tol)$_3$ (Figure S56). Color scheme associated with run number is identical to the associated overlaid figure.

DSC thermal cycling (10 cycles, 25-150°C, 5°C/min) for re-crystallized Sb(*p*-Tol)$_3$ with added silicon oil impurity: overlay

A fresh metal needle was used to introduce 0.54 mg of silicon oil to an empty 40 µL Tzero Alumina Hermetic DSC pan, followed by re-crystallized Form I Sb(*p*-Tol)$_3$ (4.97 mg). Form II in this experiment appeared more readily compared to the analogous process in the absence of silicon oil.

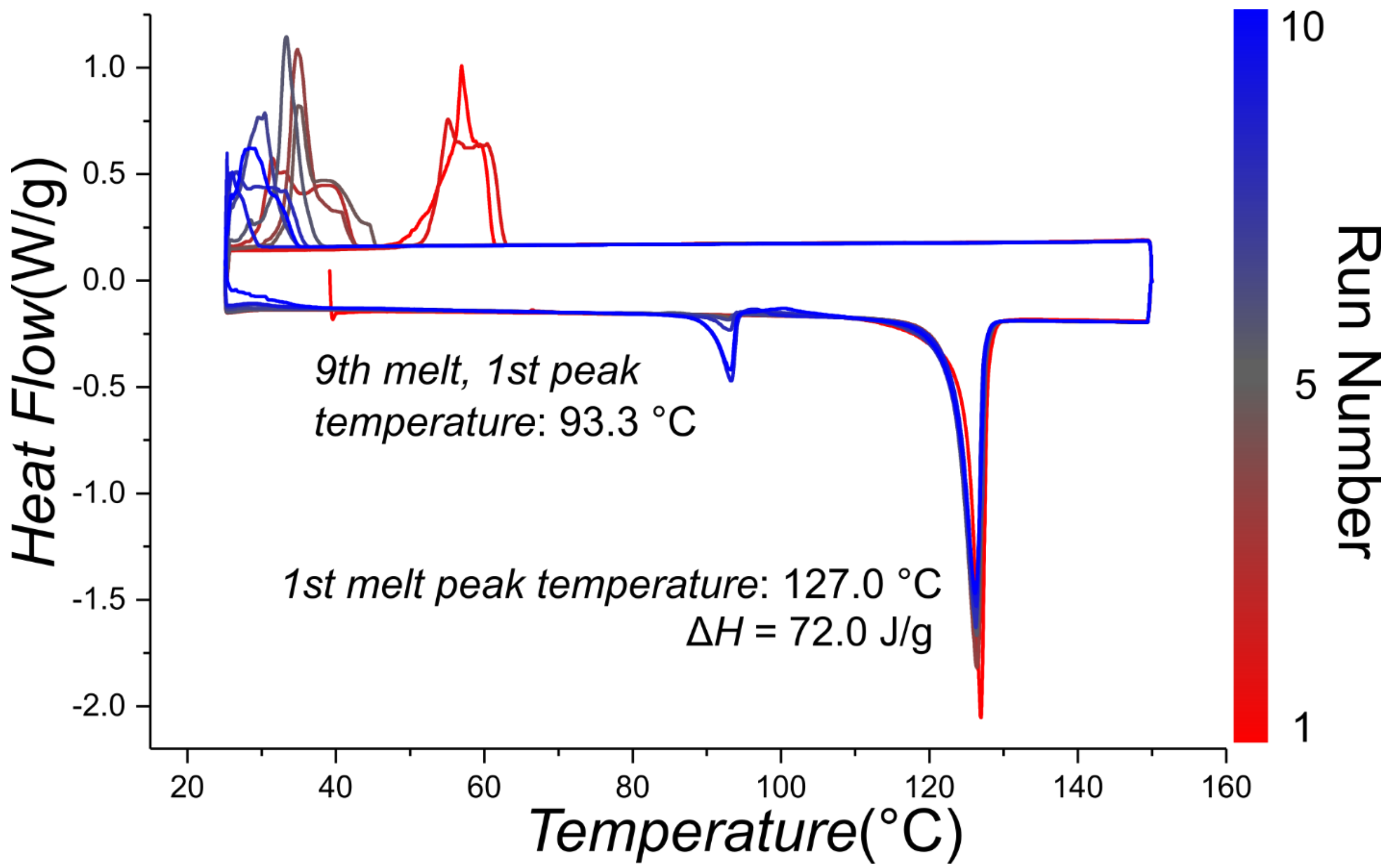

Figure S61. Overlay of all 10 DSC cycles performed on re-crystallized Sb(*p*-Tol)$_3$ in the presence of silicon oil, with a gradient color scheme from red to blue. The melting point temperature and enthalpy for Form I are calculated based on 1st cycle, and the melting point temperature for Form II is given for the 9th cycle. Minima are observed at 93.3˚C and 127.0˚C with onset points of 90.5˚C and 123.5˚C respectively.

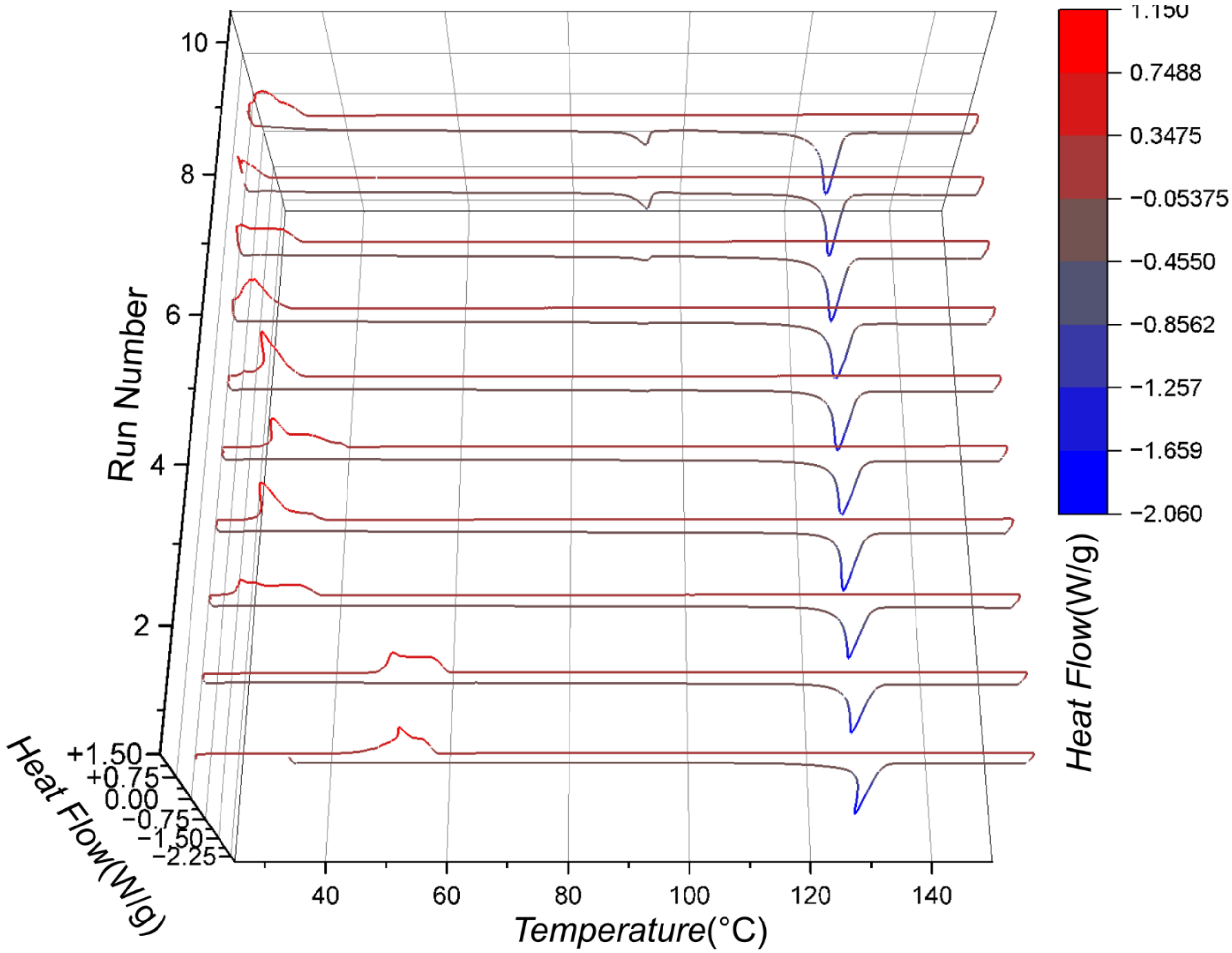

Figure S62. 3D plot of all 10 thermograms on re-crystallized Sb(p-Tol)$_3$ with an added silicon oil impurity (Figure S61) in ascending run order front-to-back. A color gradient is applied to thermograms, spanning from red to grey to blue with respect to heat flow value for additional visual contrast between exo- and endothermic signals.

DSC thermal cycling (10 cycles, 25-150°C, 5°C/min) for re-crystallized Sb(*p*-Tol)$_3$ with added silicon oil impurity: individual runs

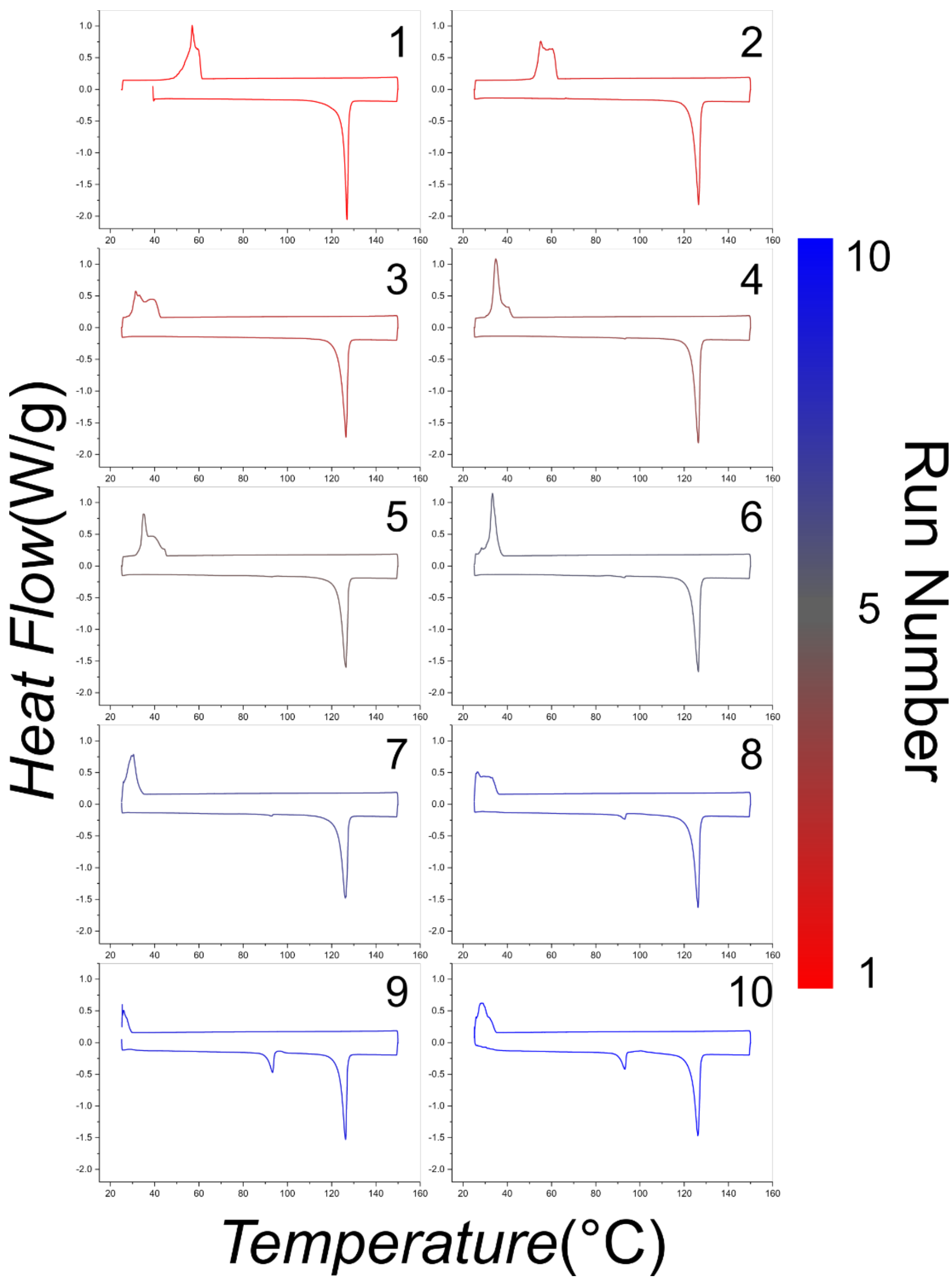

Figure S63. Grid of all thermograms from a set of 10 DSC heating-cooling cycles on re-crystallized Sb(*p*-Tol)$_3$ in the presence of silicon oil (Figure S61). Color scheme associated with run numbering is identical to the associated overlaid figure.

DSC thermal cycling (10 cycles, 25-150°C, 5°C/min) for re-crystallized Sb(*p*-Tol)$_3$ with added vacuum grease impurity: overlay

A fresh metal needle was used to introduce 0.81 mg of vacuum grease to an empty 40 µL Tzero Alumina Hermetic DSC pan, followed by re-crystallized Form I Sb(*p*-Tol)$_3$ (6.45 mg). None of the DSC cycles revealed the appearance of Form II, suggesting that vacuum grease might be inhibiting the formation of Form II.

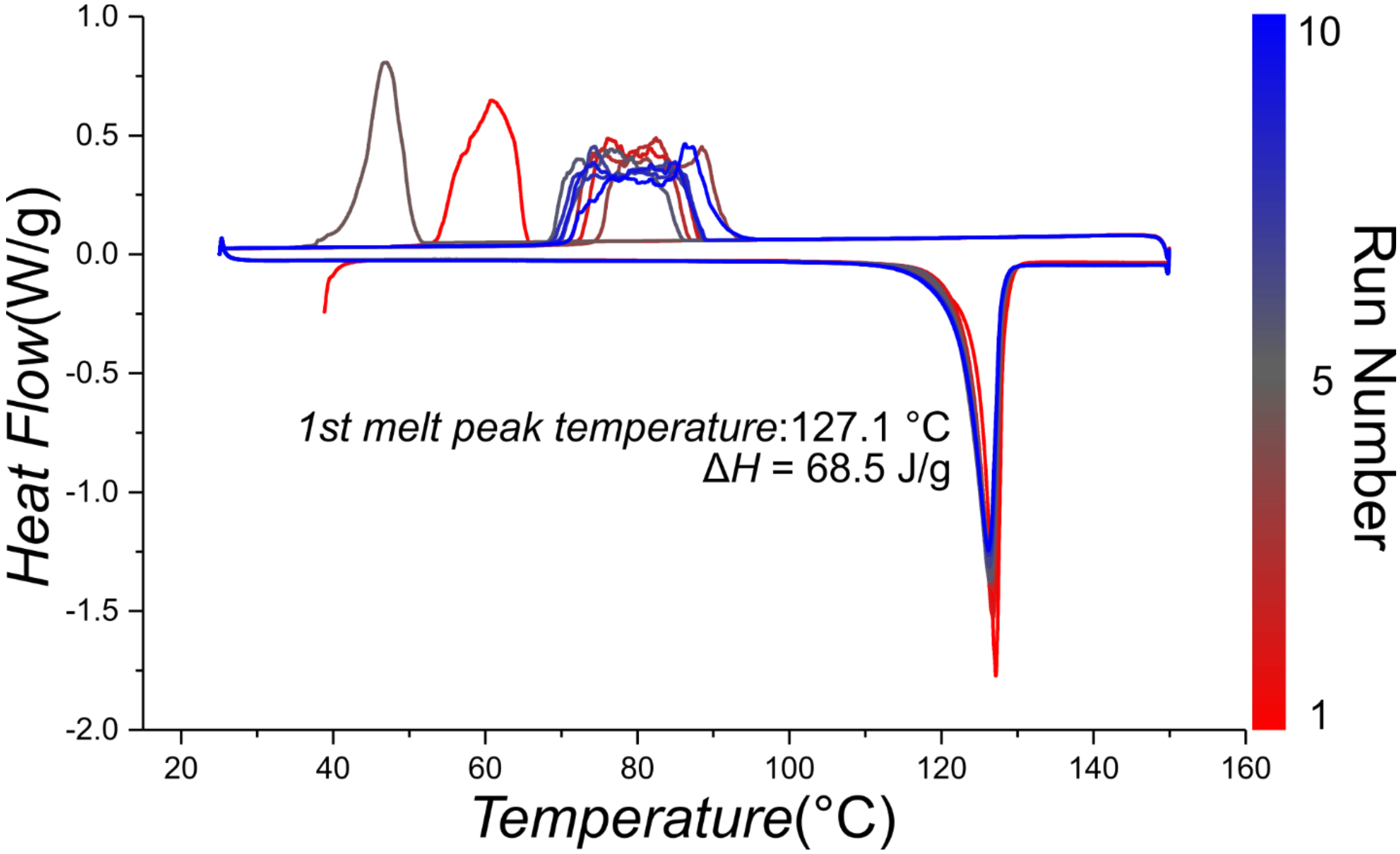

Figure S64. Overlay of all 10 DSC cycles performed on re-crystallized Sb(*p*-Tol)$_3$ in the presence of vacuum grease, with a gradient colour scheme from red to blue. Calculated melting temperature and peak enthalpy of the first and most energetic/well-defined melt is shown. A minimum is observed at 127.1˚C with an onset point of 125.0˚C.

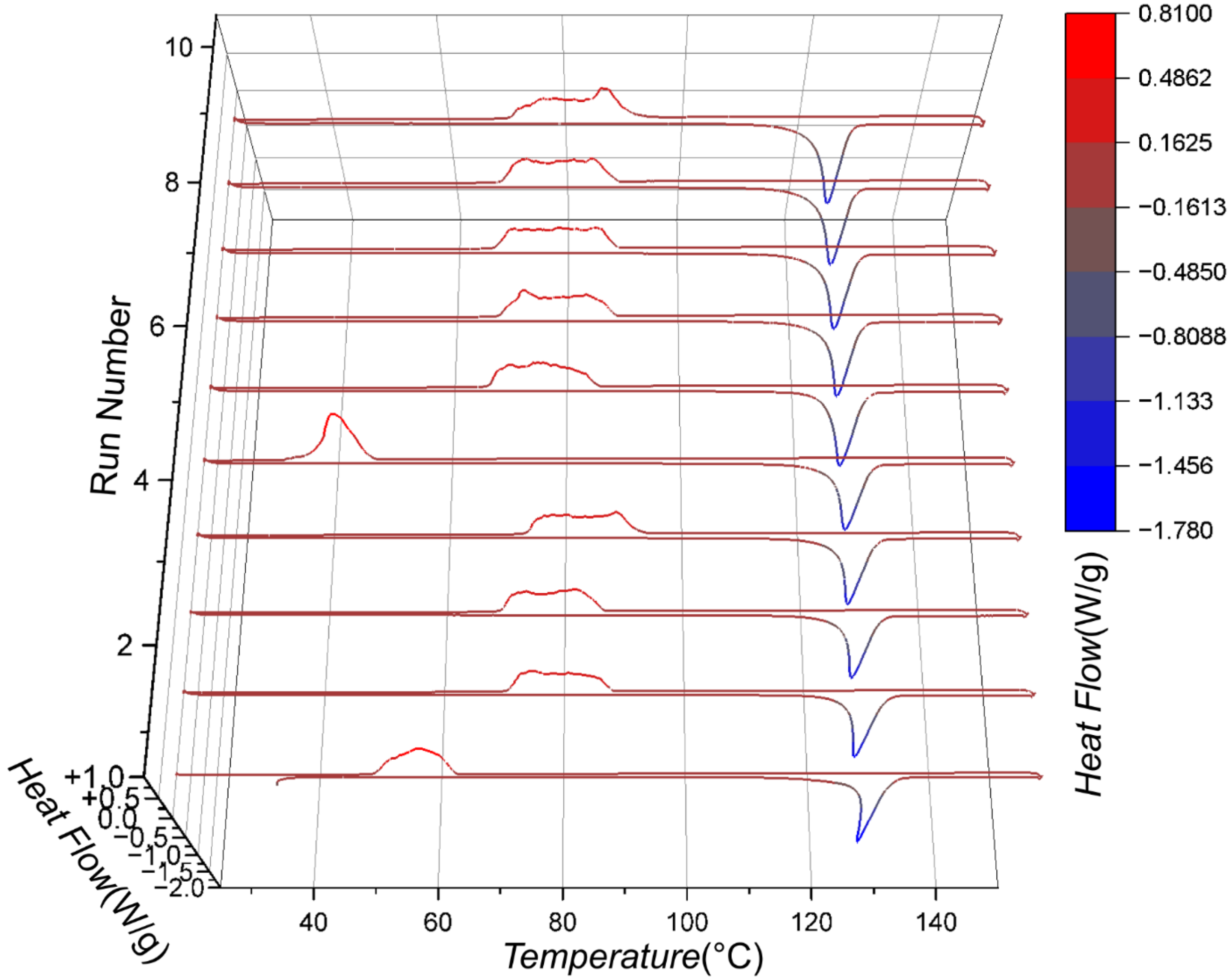

Figure S65. 3D plot of all 10 thermograms on re-crystallized Sb(*p*-Tol)$_3$ with an added vacuum grease impurity (Figure S64) in ascending run order front-to-back. A color gradient is applied to thermograms, spanning from red to grey to blue with respect to heat flow value for additional visual contrast between exo- and endothermic signals.

DSC thermal cycling (10 cycles, 25-150°C, 5°C/min) for re-crystallized Sb(*p*-Tol)$_3$ with added vacuum grease impurity: individual runs

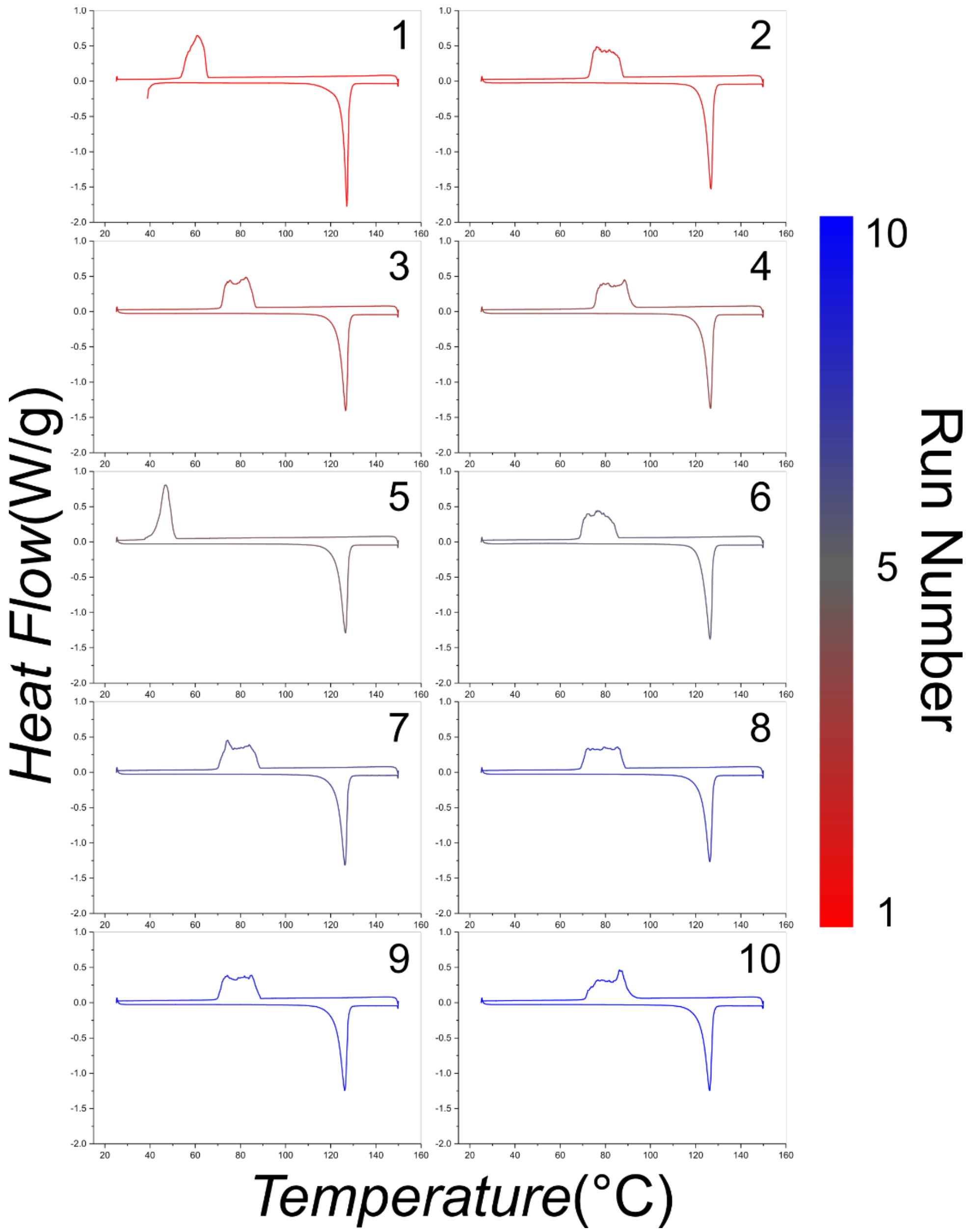

Figure S66. Grid of all thermograms from a set of 10 DSC heating-cooling cycles on re-crystallized Sb(*p*-Tol)$_3$ in the presence of vacuum grease (Figure S64). Colour scheme associated with run number is identical to the associated overlaid figure except for run 6.

DSC thermal cycling (20 cycles, 25–150°C, 5°C/min) for Sb(*p*-Tol)$_3$ synthesized at Babeș-Bolyai University: overlay

A sample of Form I Sb(*p*-Tol)$_3$ (4.348 mg) was placed in a hermetically sealed 40 µL Tzero Alumina Hermetic DSC pan and subjected to 20 heating and 20 cooling cycles at a heating/cooling rate of 5°C/min, with each temperature ramp separated by a 1-minute isotherm. The initial cycle started at 25°C and all experiments were performed in nitrogen flow (50 mL/min).

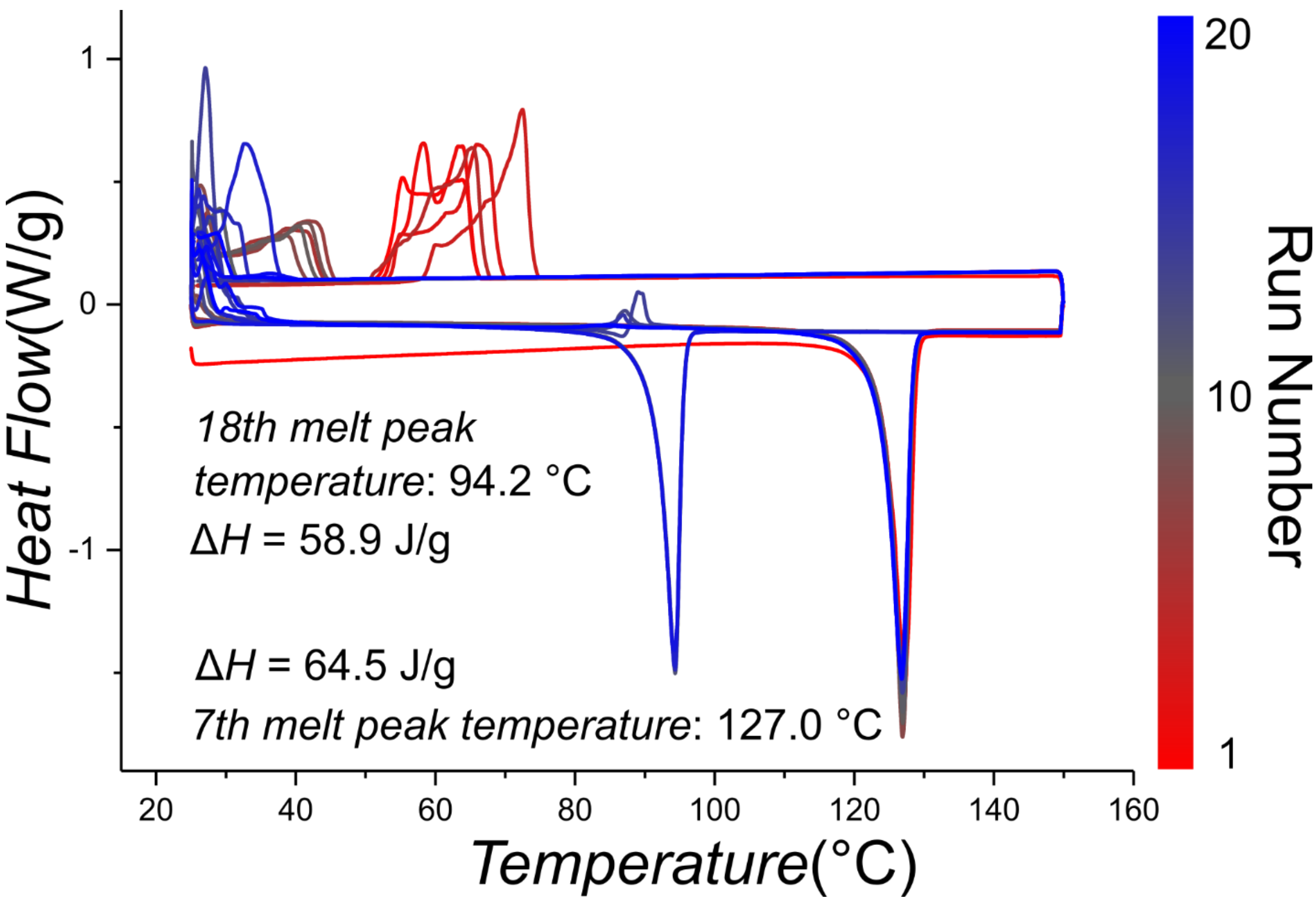

Figure S67. Overlay of all 20 DSC cycles performed on Sb(*p*-Tol)$_3$ synthesized at Babeș-Bolyai University, with a gradient colour scheme from red to blue. Minima are observed at 94.3°C and 127.0°C with onset points of 91.3°C and 124.5°C, respectively.

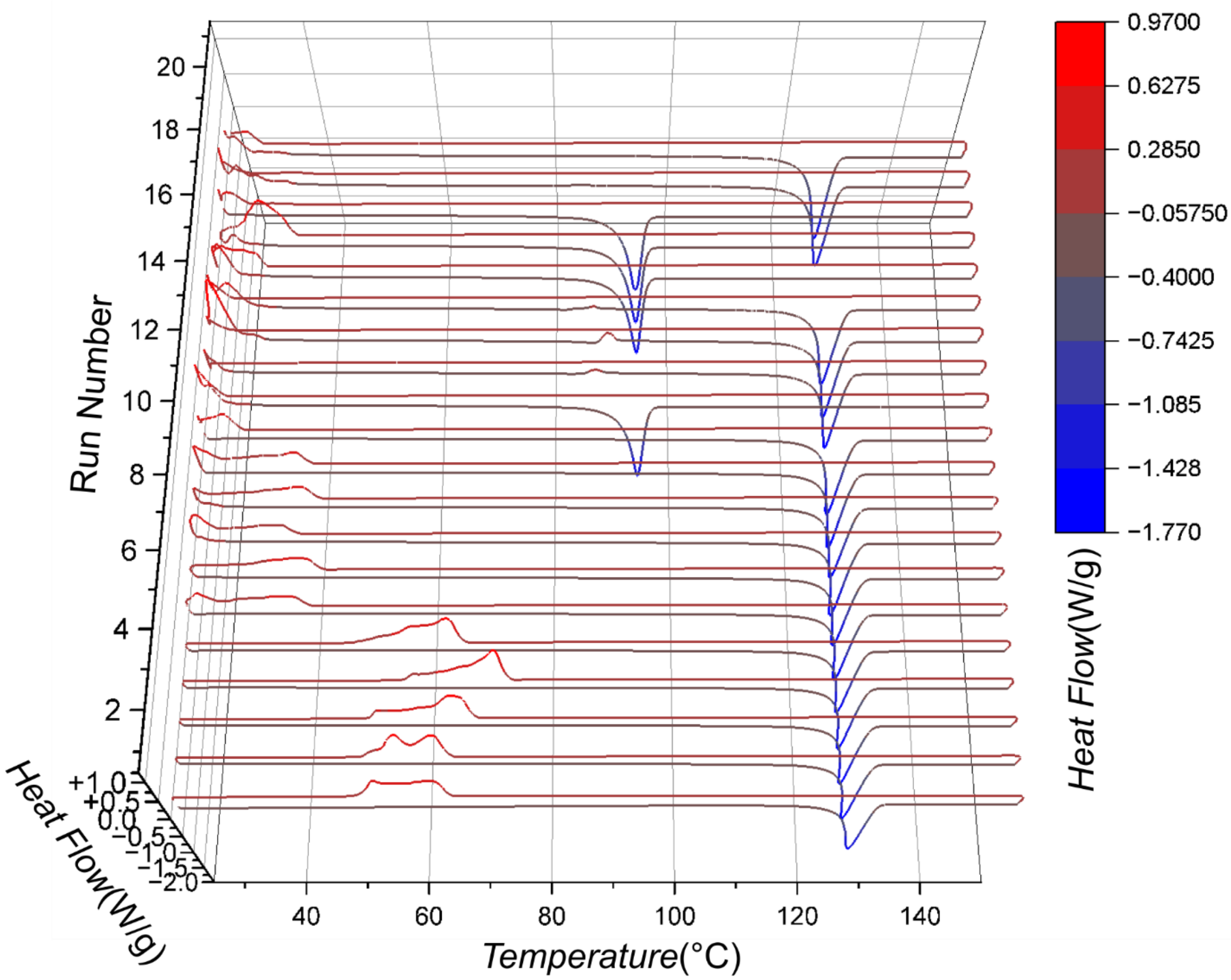

Figure S68. 3D plot of all 20 thermograms on Sb(*p*-Tol)$_3$ synthesized at Babeș-Bolyai University (Figure S67) in ascending order front-to-back. A color gradient is applied to thermograms, spanning from red to grey to blue with respect to heat flow value for additional visual contrast between exo- and endothermic signals.

DSC thermal cycling (20 cycles, 25-150°C, 5°C/min) for Sb(p-Tol)$_3$ synthesized at Babeș-Bolyai University: individual runs

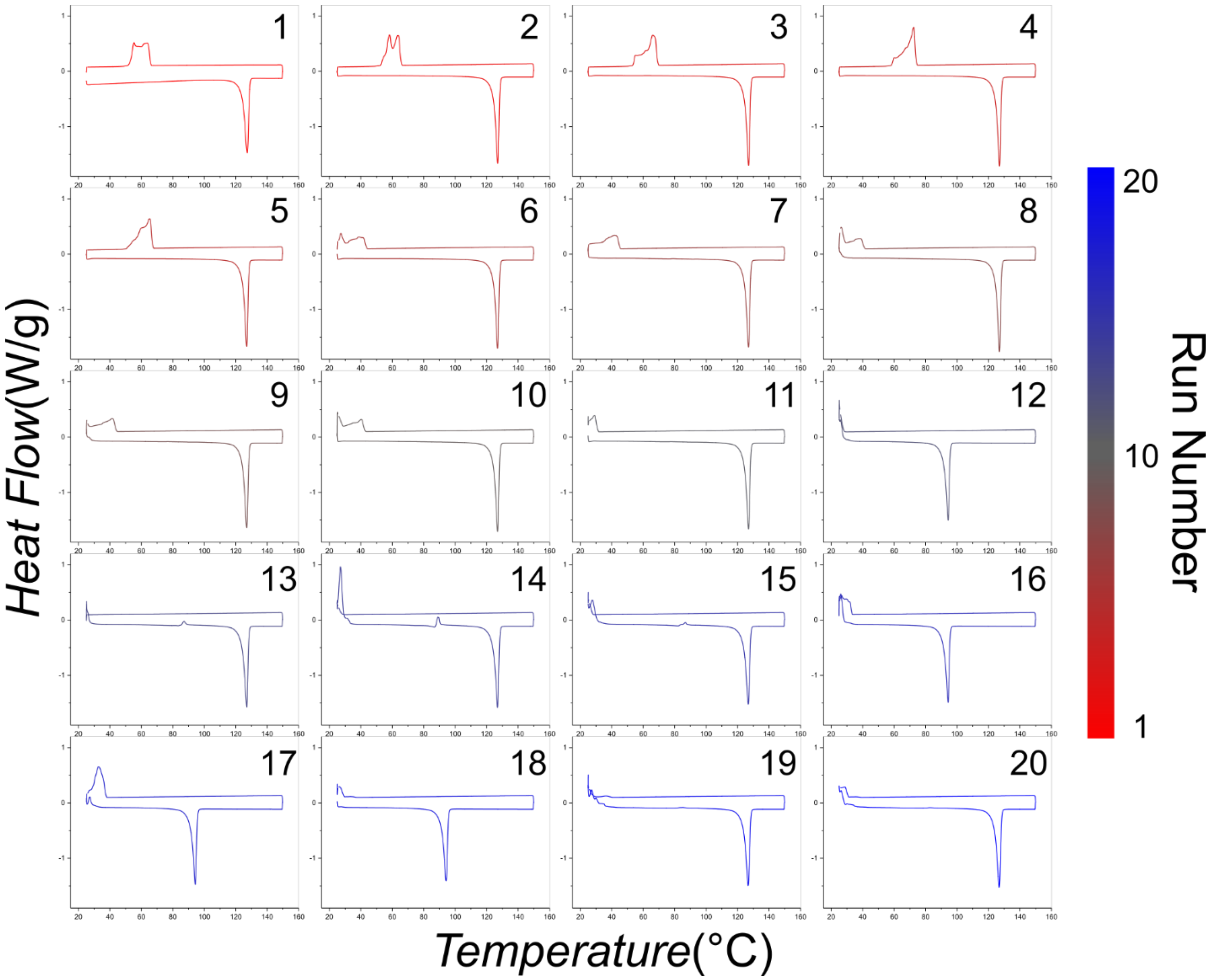

Figure S69. Grid of all thermograms from a set of 20 DSC heating-cooling cycles on Form I Sb(p-Tol)$_3$ (Figure S67). Color scheme associated with run number is identical to the associated overlaid figure.

DSC thermal cycling (40 cycles, 25-150°C, 5°C/min) for Sb(*p*-Tol)$_3$ synthesized at Babeș-Bolyai University: overlay

A sample of Form I Sb(*p*-Tol)$_3$ (7.764 mg) was placed in a hermetically sealed 40 µL Tzero Alumina Hermetic DSC pan and subjected to 40 heating and 40 cooling cycles at a heating/cooling rate of 5°C/min, with each temperature ramp separated by a 1-minute isotherm. The initial cycle started at 25°C and all experiments were performed in nitrogen flow (50 mL/min).

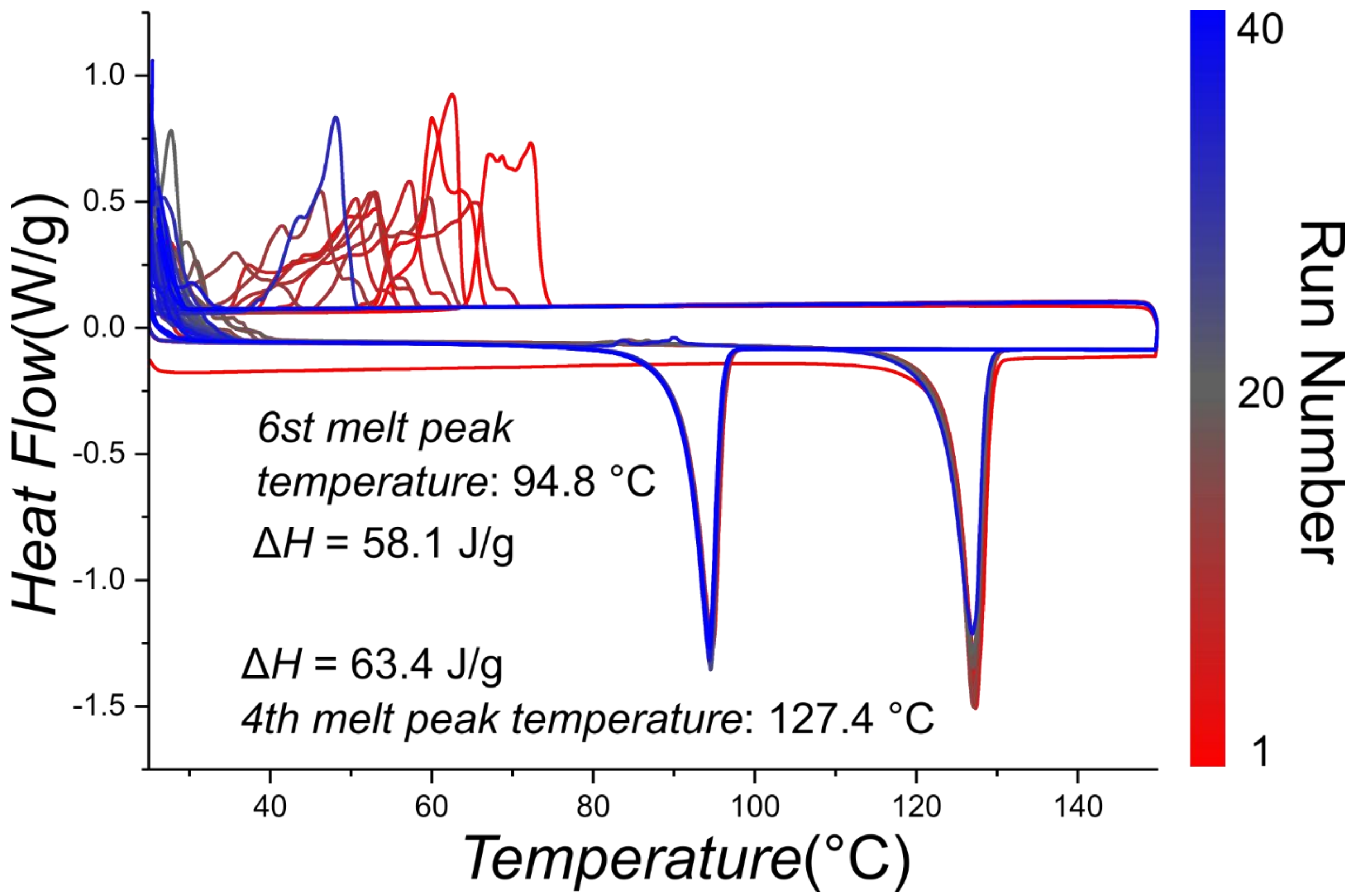

Figure S70. Overlay of all 40 DSC cycles performed on Sb(*p*-Tol)$_3$ synthesized at Babeș-Bolyai University, with a gradient color scheme from red to blue. Minima are observed at 94.8°C and 127.4°C with onset points of 91.1°C and 124.3°C, respectively.

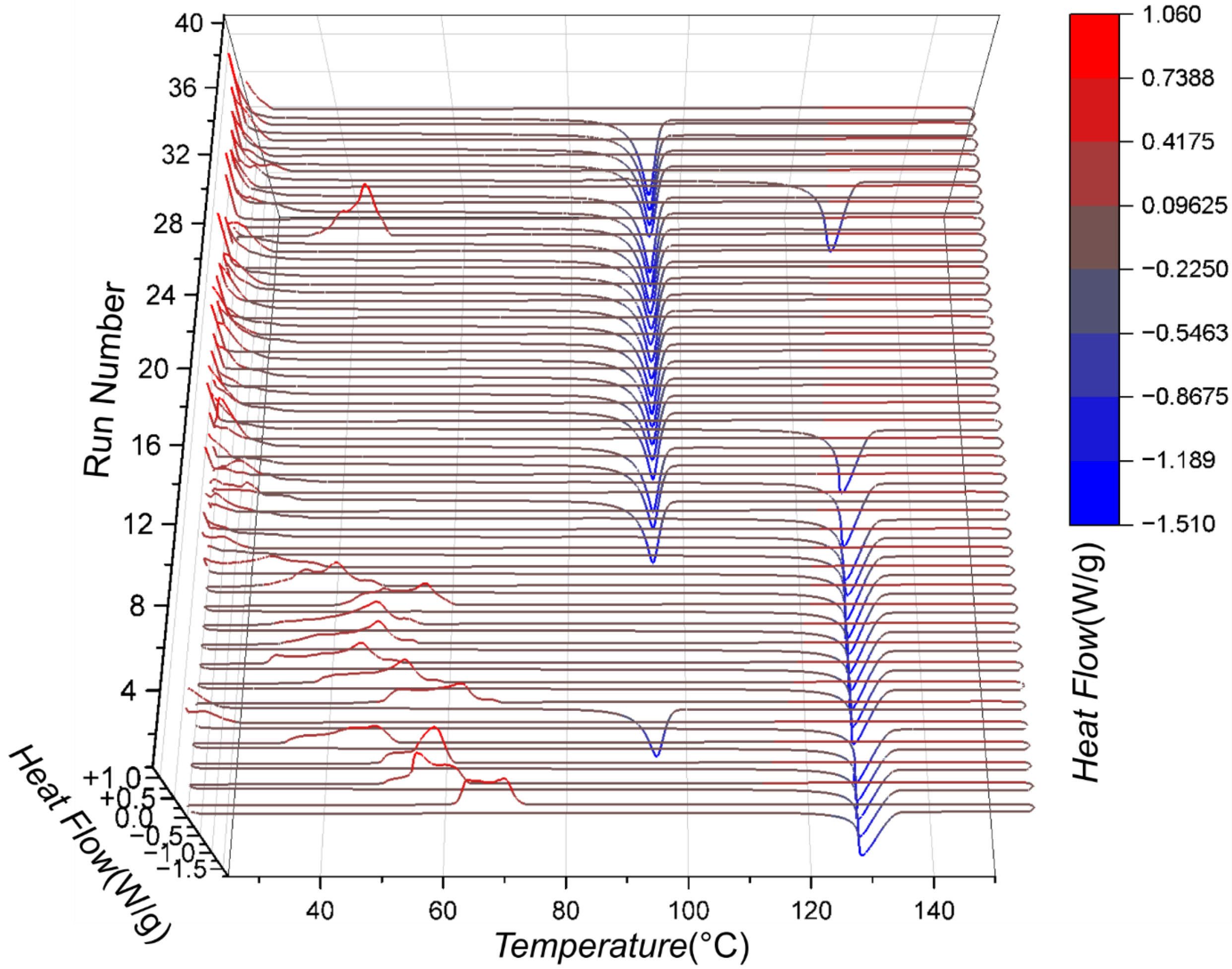

Figure S71. 3D plot of all thermograms on Sb(*p*-Tol)$_3$ synthesized at Babeș-Bolyai University (Figure S70) in ascending run order front-to-back. A color gradient is applied to thermograms, spanning from red to grey to blue with respect to heat flow value for additional visual contrast between exo- and endothermic signals.

DSC thermal cycling (40 cycles, 25-150°C, 5°C/min) for Sb(p-Tol)$_3$ synthesized at Babeș-Bolyai University: individual runs 1–20

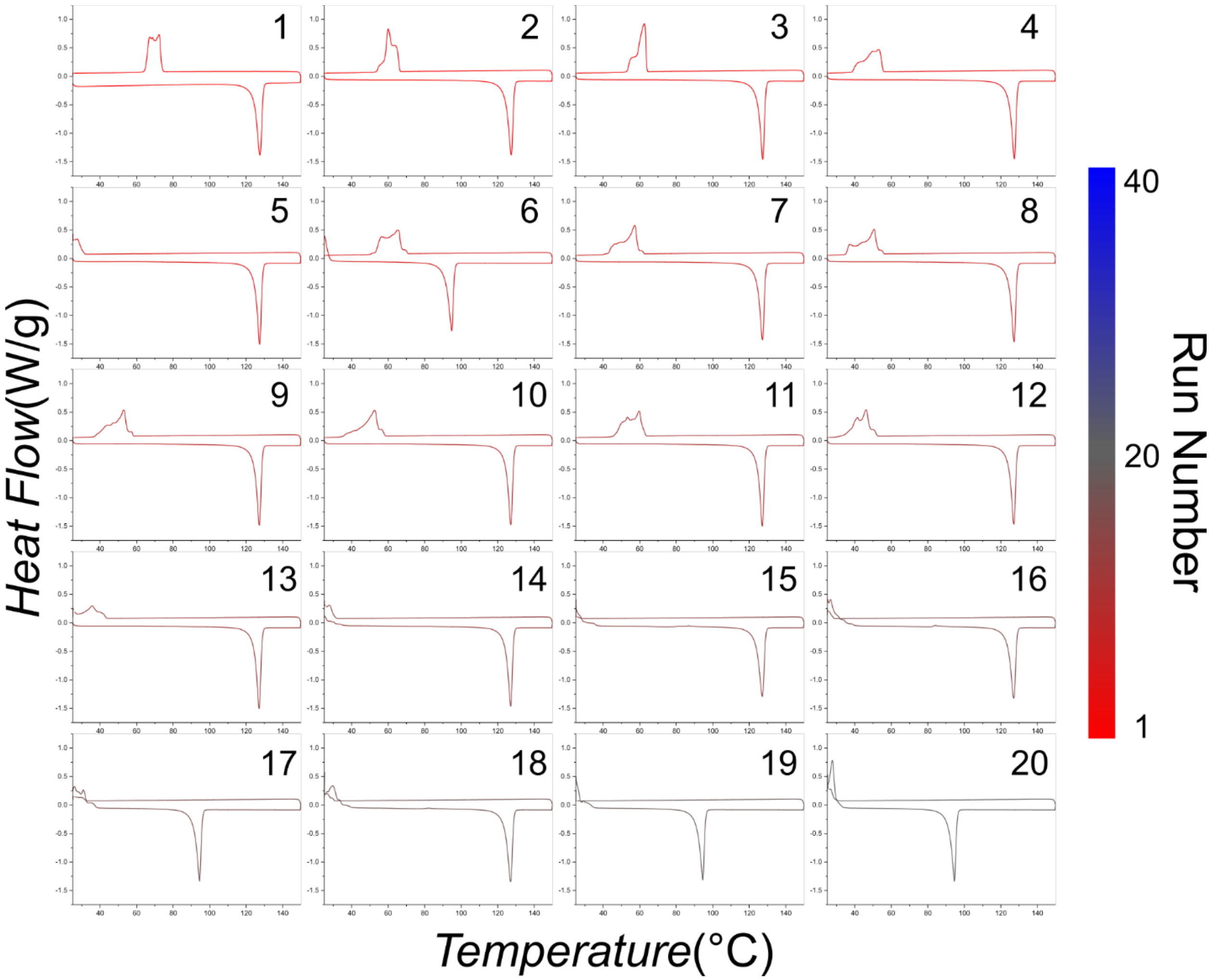

Figure S72. Grid of the first 20 thermograms from a set of 40 DSC heating-cooling cycles on Form I Sb(p-Tol)$_3$ (Figure S70). Color scheme associated with run number is identical to the associated overlaid figure.

DSC thermal cycling (40 cycles, 25-150°C, 5°C/min) for Sb(*p*-Tol)$_3$ synthesized at Babeș-Bolyai University: individual runs 21–40

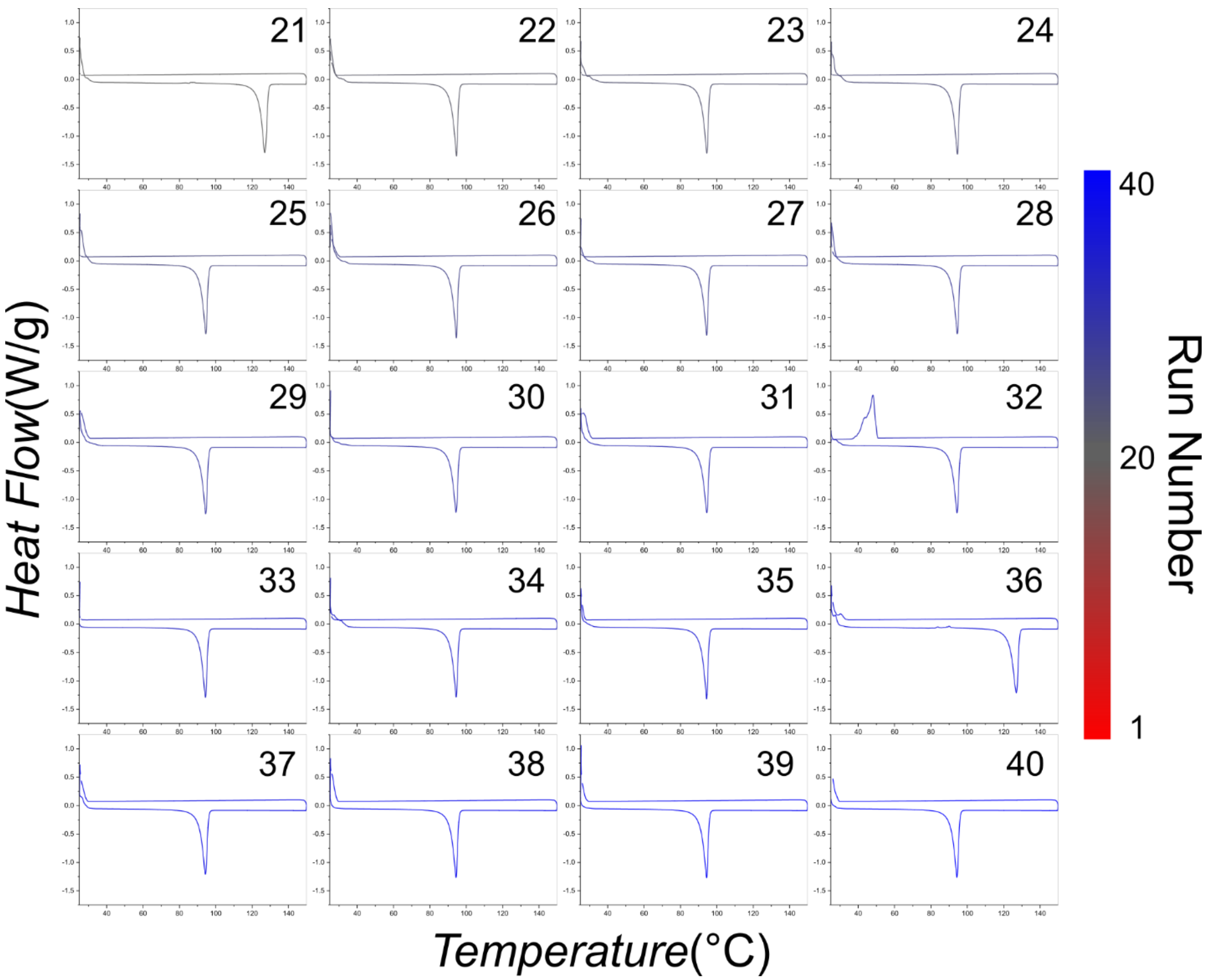

Figure S73. Grid of the last 20 thermograms from a set of 40 DSC heating-cooling cycles on Form I Sb(*p*-Tol)$_3$ (Figure S70). Color scheme associated with run number is identical to the associated overlaid figure.

DSC thermal cycling (40 cycles, 25-150°C, 10°C/min) for Sb(*p*-Tol)$_3$ synthesized at Babeș-Bolyai University: overlay

A sample of Form I Sb(*p*-Tol)$_3$ (8.842 mg) was placed in a hermetically sealed 40 µL Tzero Alumina Hermetic DSC pan and subjected to 40 heating and 40 cooling cycles at a heating/cooling rate of 10°C/min, with each temperature ramp separated by a 1-minute isotherm. The initial cycle started at 25°C and all experiments were performed in nitrogen flow (50 mL/min).

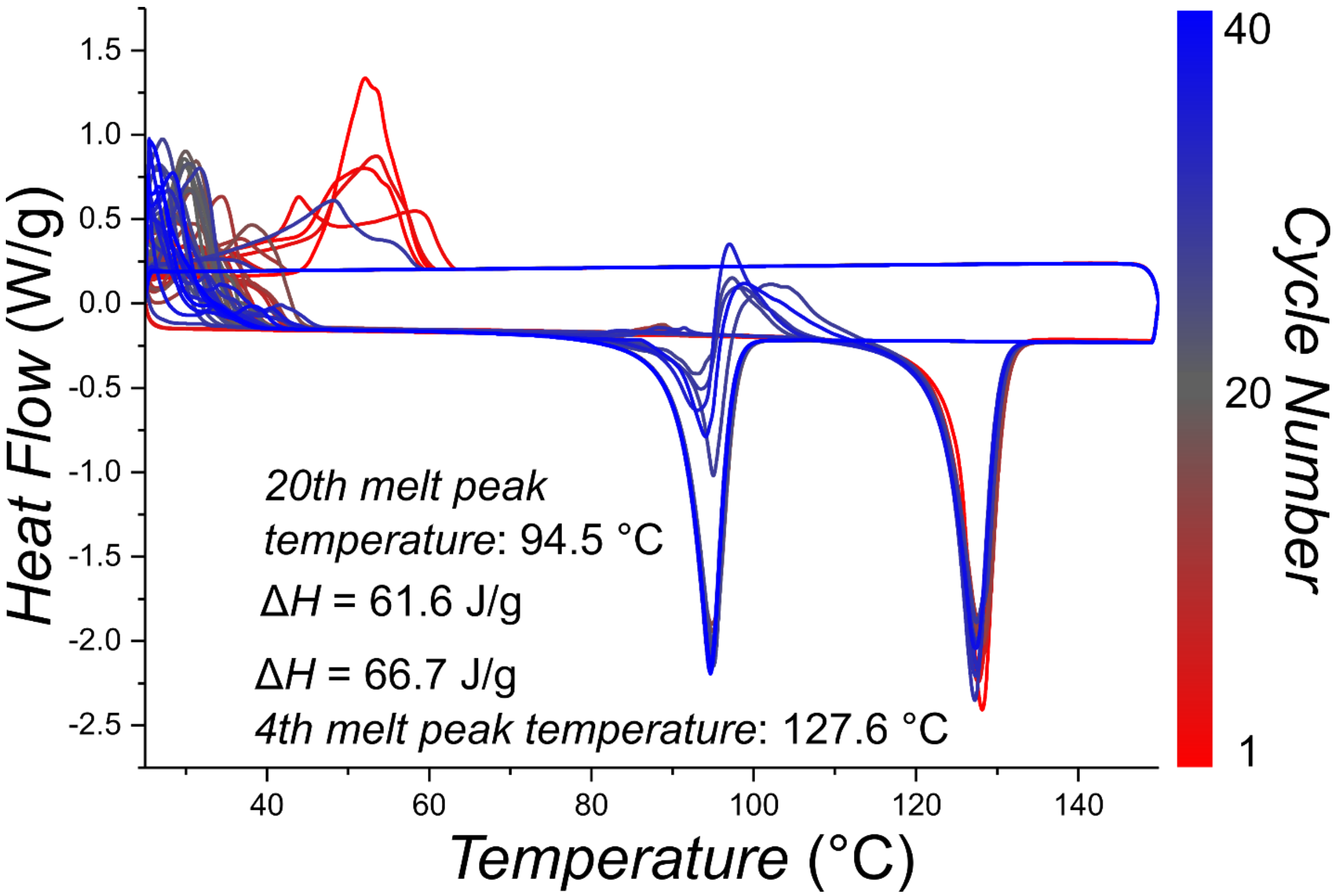

Figure S74. Overlay of all 40 DSC cycles performed on Sb(*p*-Tol)$_3$ synthesized at Babeș-Bolyai University, with a gradient color scheme from red to blue. Minima are observed at 94.5°C and 127.6°C with onset points of 90.5°C and 123.6°C, respectively.

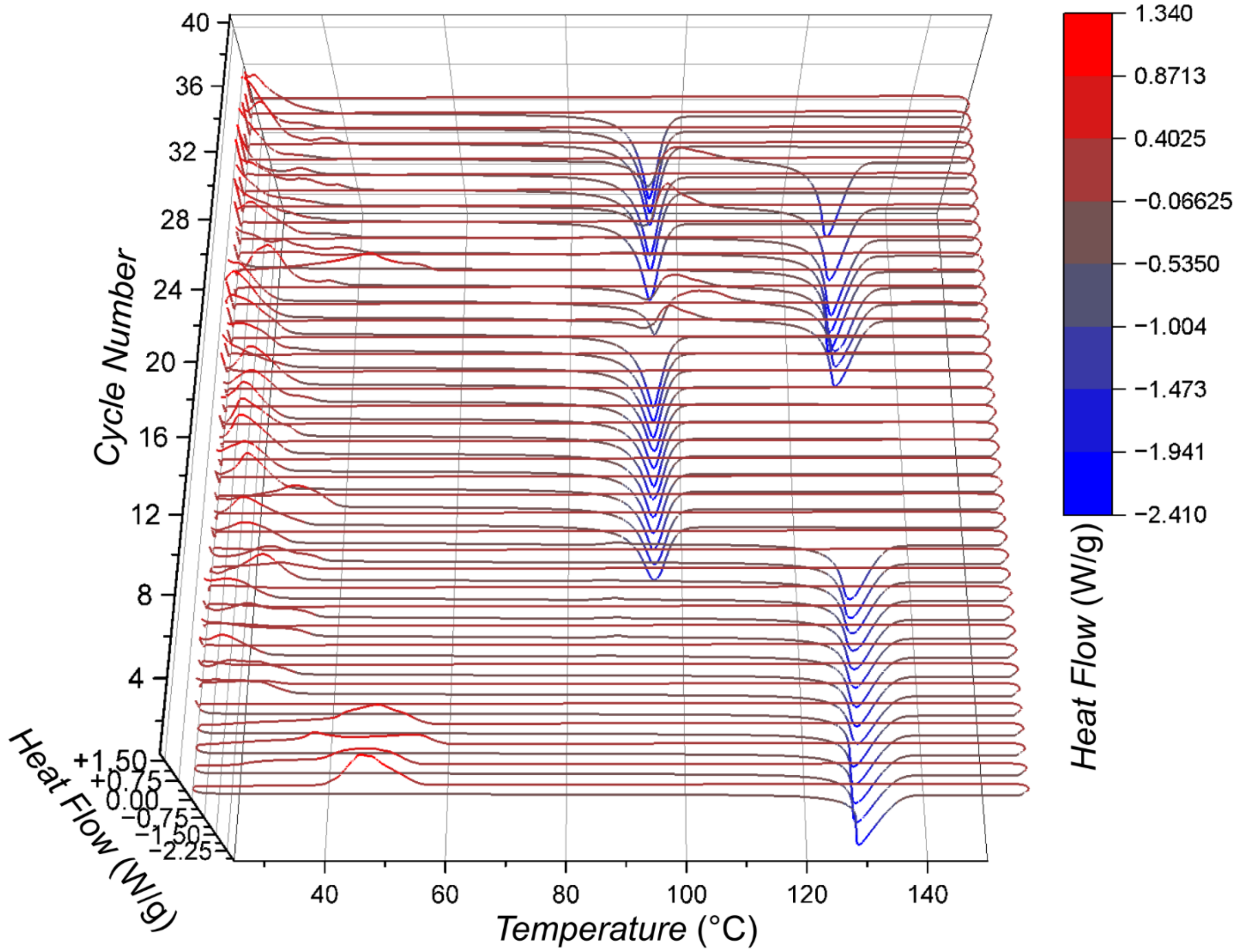

Figure S75. 3D plot of all 40 thermograms on Sb(p-Tol)$_3$ synthesized at Babeș-Bolyai University, run at 10˚C/min (Figure S74) in ascending run order front-to-back. A color gradient is applied to thermograms, spanning from red to grey to blue with respect to heat flow value for additional visual contrast between exo- and endothermic signals.

DSC thermal cycling (40 cycles, 25-150°C, 10°C/min) for Sb(*p*-Tol)$_3$ synthesized at Babeș-Bolyai University: individual runs 1–20

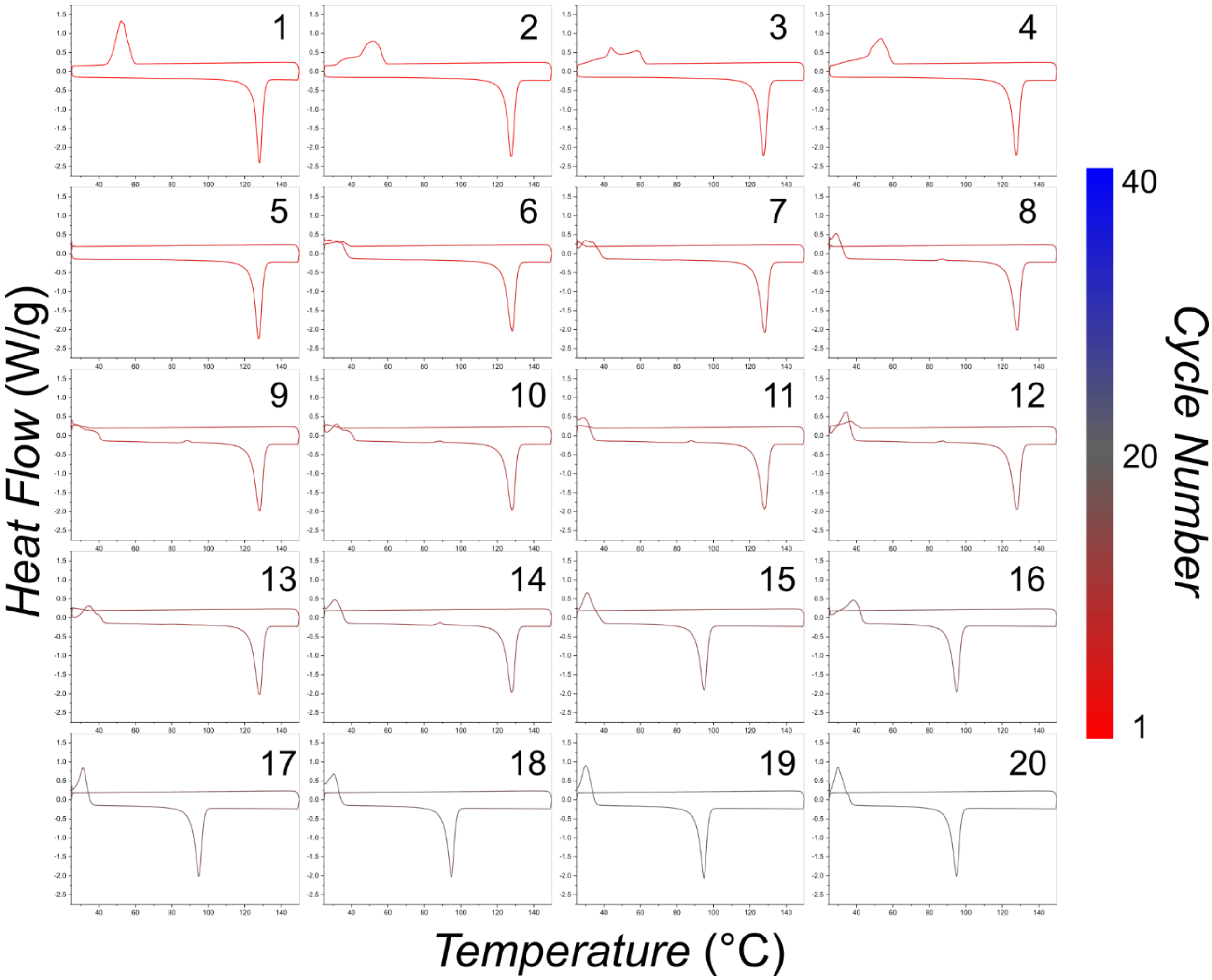

Figure S76. Grid of the first 20 thermograms from a set of 40 DSC heating-cooling cycles on Form I Sb(*p*-Tol)$_3$ (Figure S74). Color scheme associated with run number is identical to the associated overlaid figure.

DSC thermal cycling (40 cycles, 25-150°C, 10°C/min) for Sb(*p*-Tol)$_3$ synthesized at Babeș-Bolyai University: individual runs 21–40

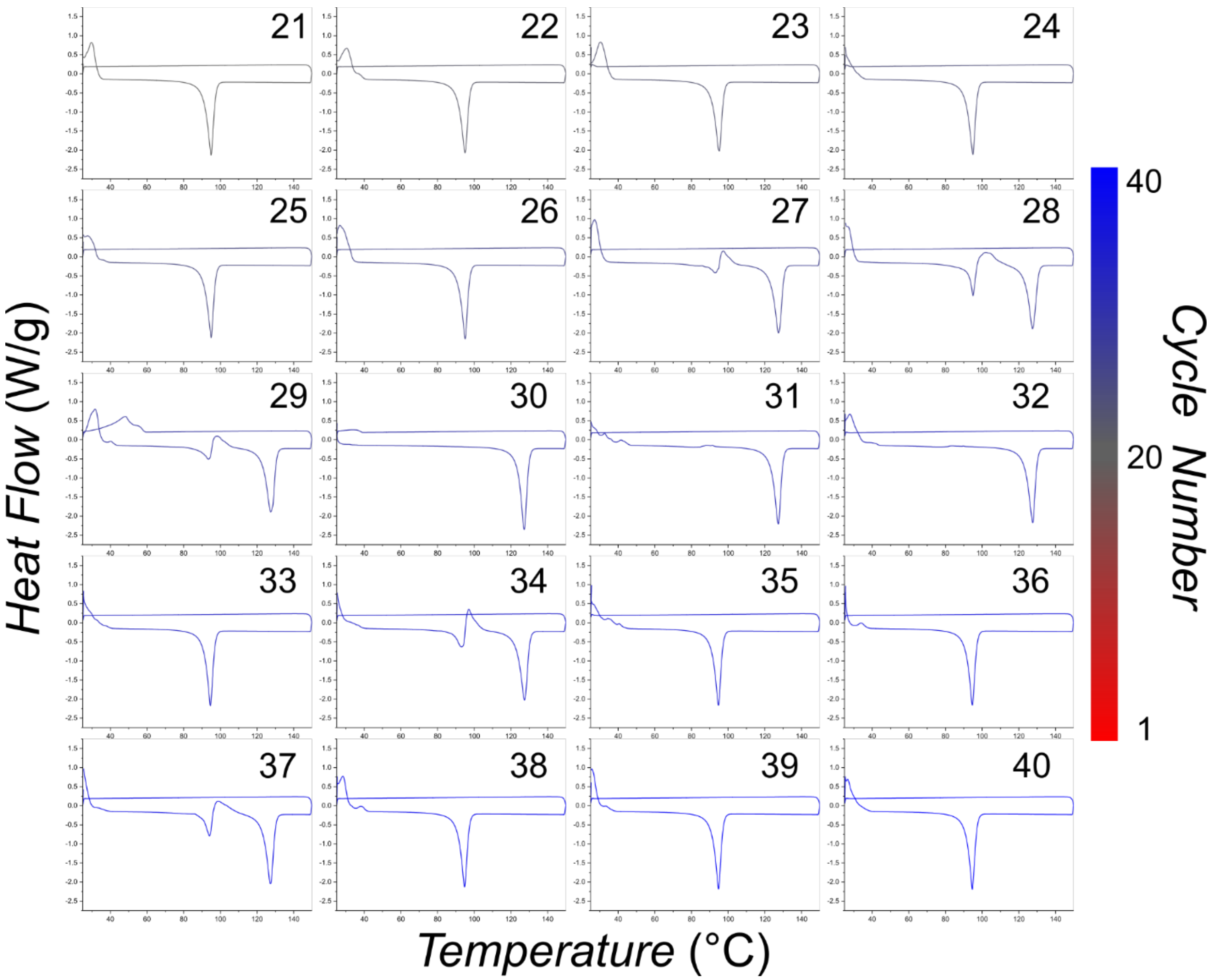

Figure S77. Grid of the last 20 thermograms from a set of 40 DSC heating-cooling cycles on Form I Sb(*p*-Tol)$_3$ (Figure S74). Color scheme associated with run number is identical to the associated overlaid figure.

DSC thermal cycling (40 cycles, 25-150°C, 2.5°C/min) for Sb(*p*-Tol)$_3$ synthesized at Babeș-Bolyai University: overlay

A sample of Form I Sb(*p*-Tol)$_3$ (9.981 mg) was placed in a hermetically sealed 40 μL Tzero Alumina Hermetic DSC pan and subjected to 40 heating and 40 cooling cycles at a heating/cooling rate of 2.5°C/min, with each temperature ramp separated by a 1-minute isotherm. The initial cycle started at 25°C and all experiments were performed in nitrogen flow (50 mL/min).

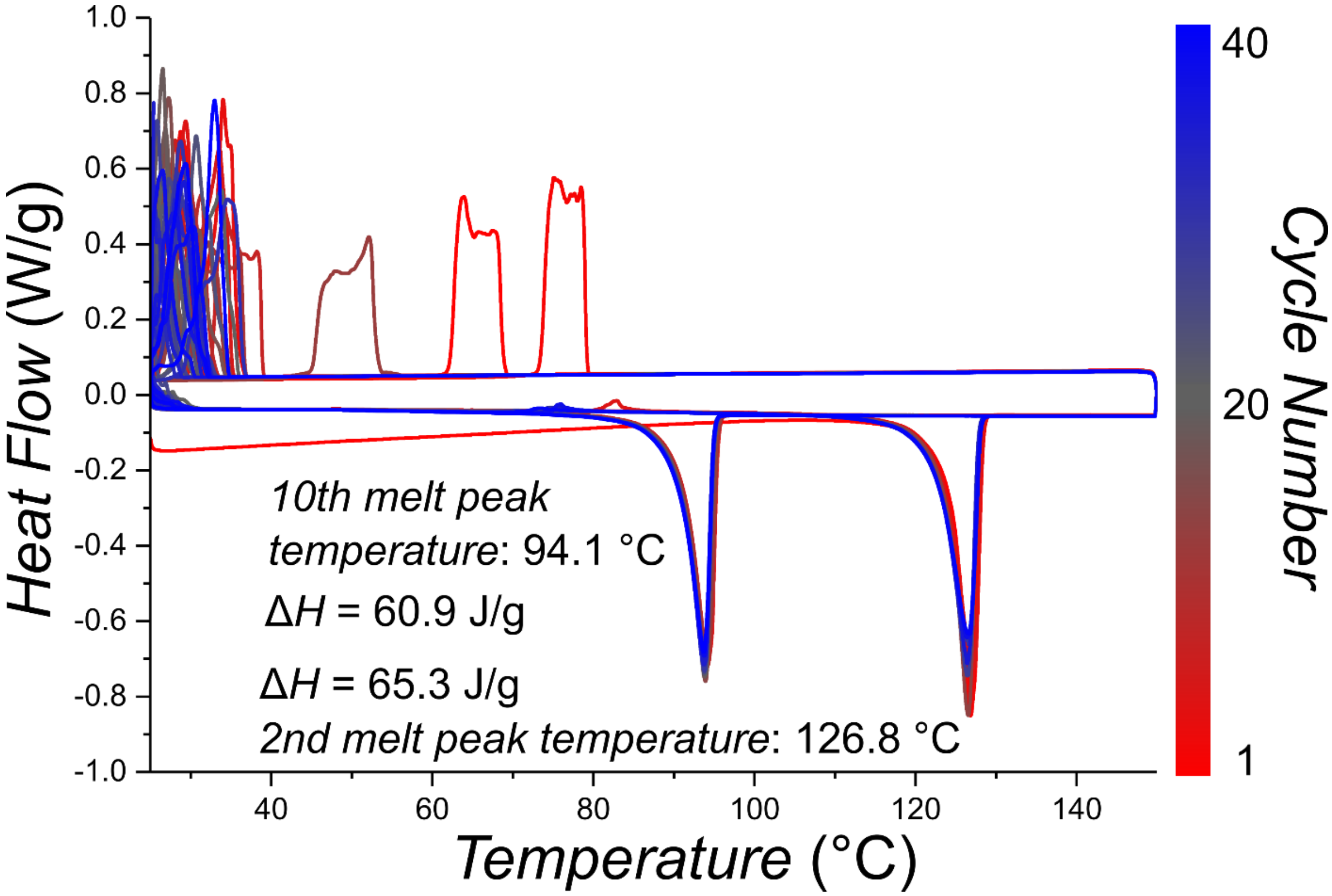

Figure S78. Overlay of all 40 DSC cycles performed on Sb(*p*-Tol)$_3$ synthesized at Babeș-Bolyai University, with a gradient color scheme from red to blue. Minima are observed at 94.1°C and 126.8°C with onset points of 90.7°C and 123.9°C, respectively.

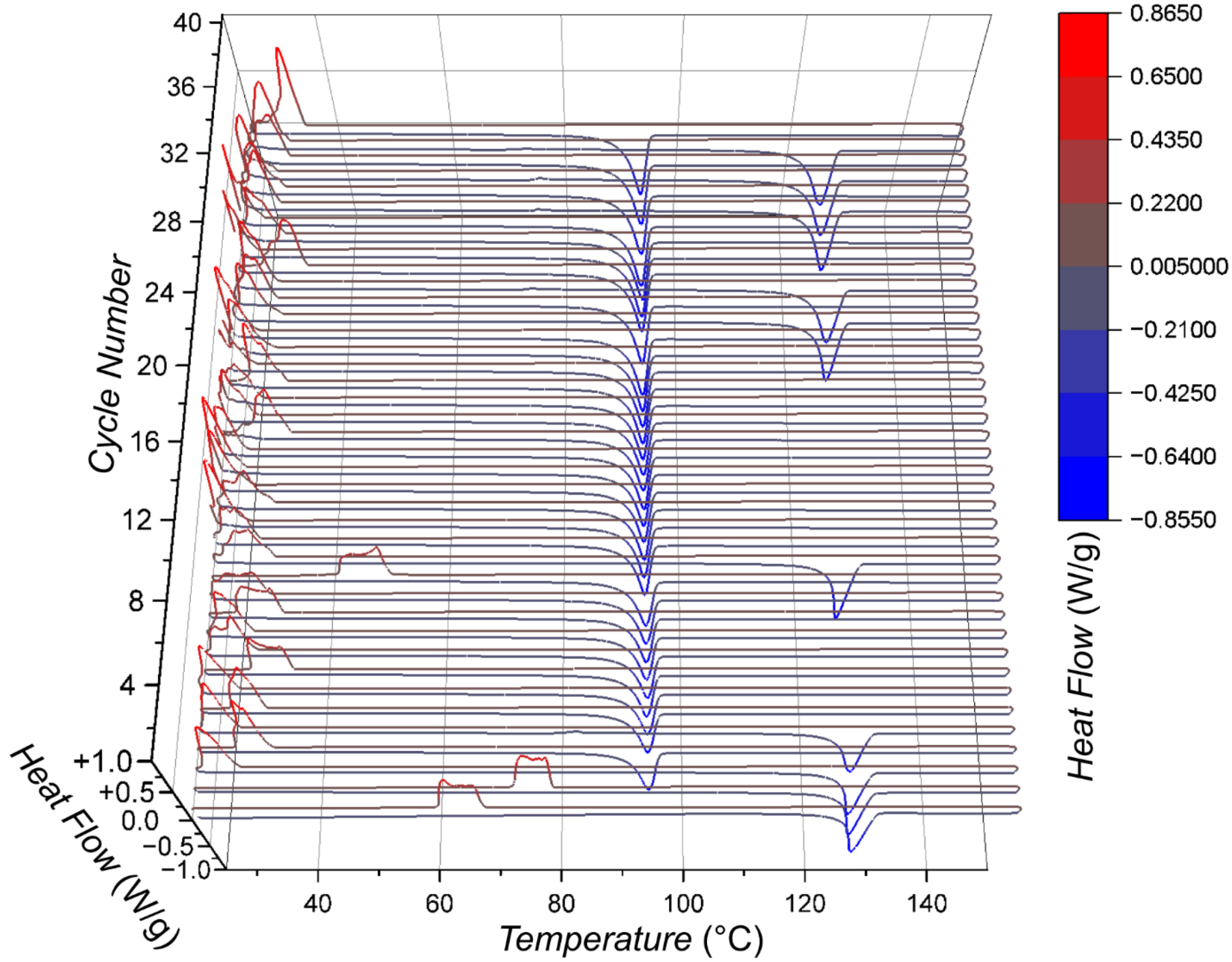

Figure S79. 3D plot of all 40 thermograms on Sb(*p*-Tol)$_3$ synthesized at Babeș-Bolyai University, run at 2.5˚C/min (Figure S78) in ascending run order front-to-back. A color gradient is applied to thermograms, spanning from red to grey to blue with respect to heat flow value for additional visual contrast between exo- and endothermic signals.

DSC thermal cycling (40 cycles, 25-150°C, 2.5°C/min) for Sb(*p*-Tol)$_3$ synthesized at Babeș-Bolyai University: individual runs 1–20

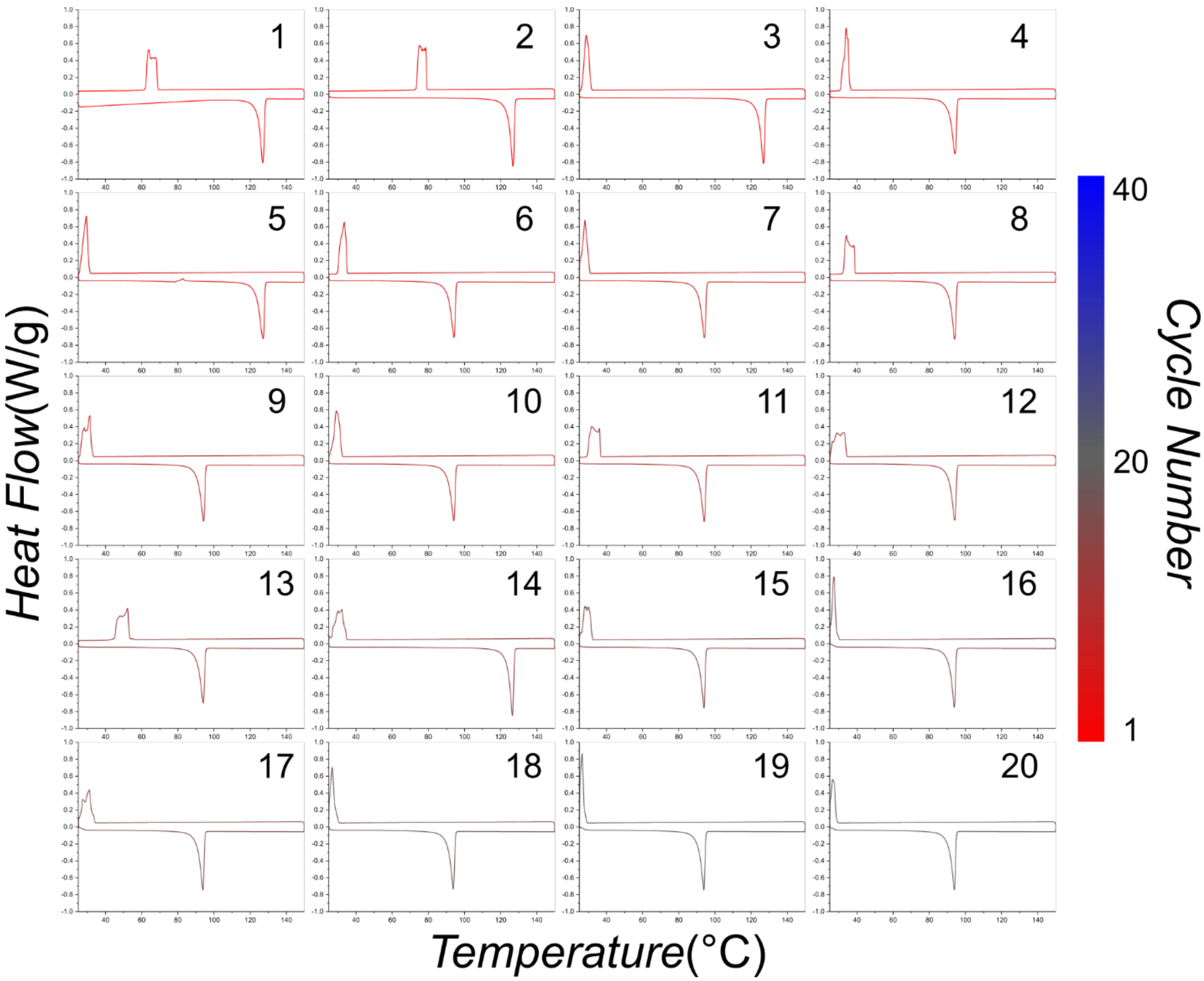

Figure S80. Grid of the first 20 thermograms from a set of 40 DSC heating-cooling cycles on Form I Sb(*p*-Tol)$_3$ (Figure S78). Color scheme associated with run number is identical to the associated overlaid figure.

DSC thermal cycling (40 cycles, 25-150°C, 2.5°C/min) for Sb(*p*-Tol)$_3$ synthesized at Babeș-Bolyai University: individual runs 21–40

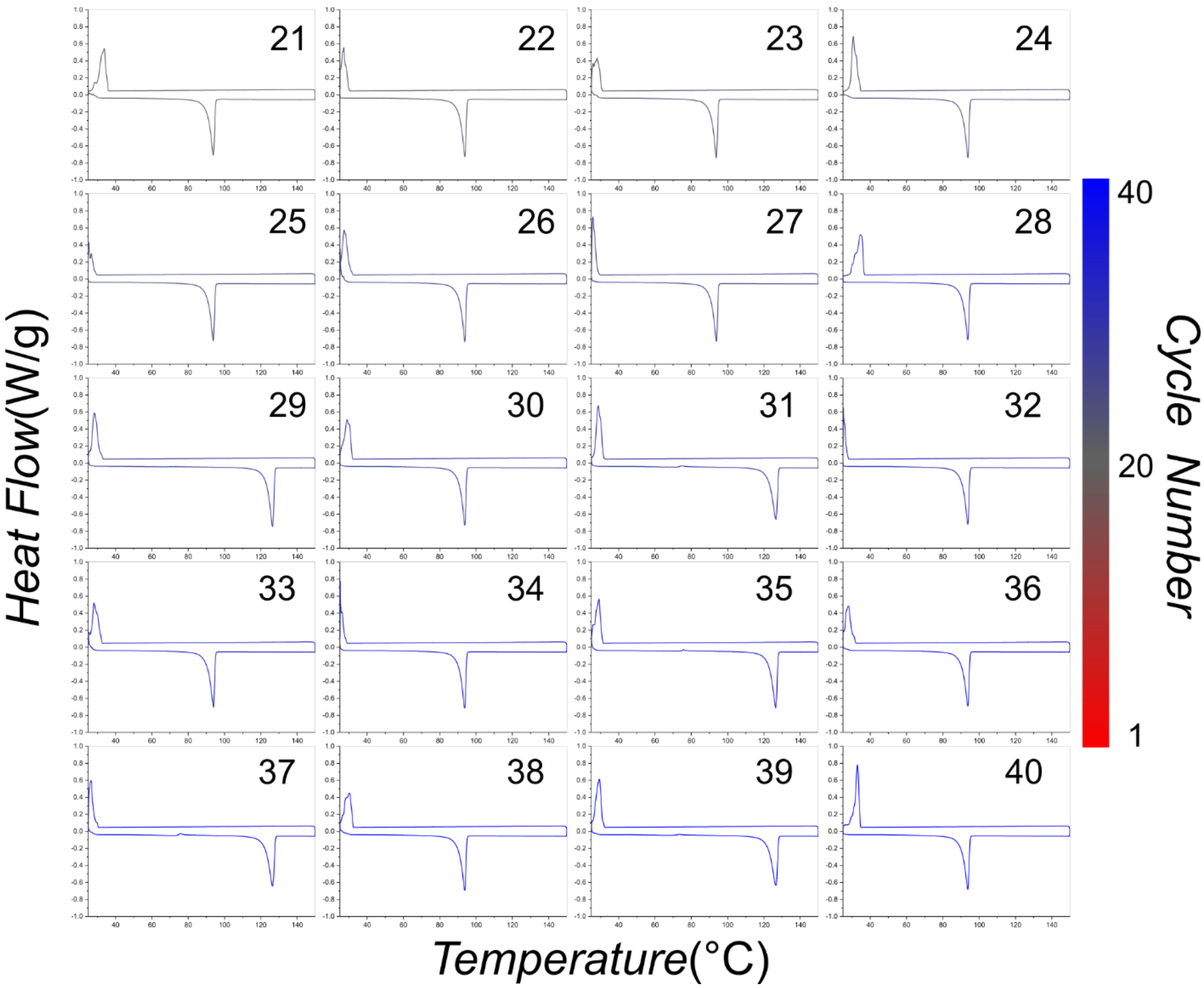

Figure S81. Grid of the last 20 thermograms from a set of 40 DSC heating-cooling cycles on Form I Sb(*p*-Tol)$_3$ (Figure S78). Color scheme associated with run number is identical to the associated overlaid figure.

PXRD patterns of Sb(p-Tol)$_3$ samples measured before and after DSC cycling

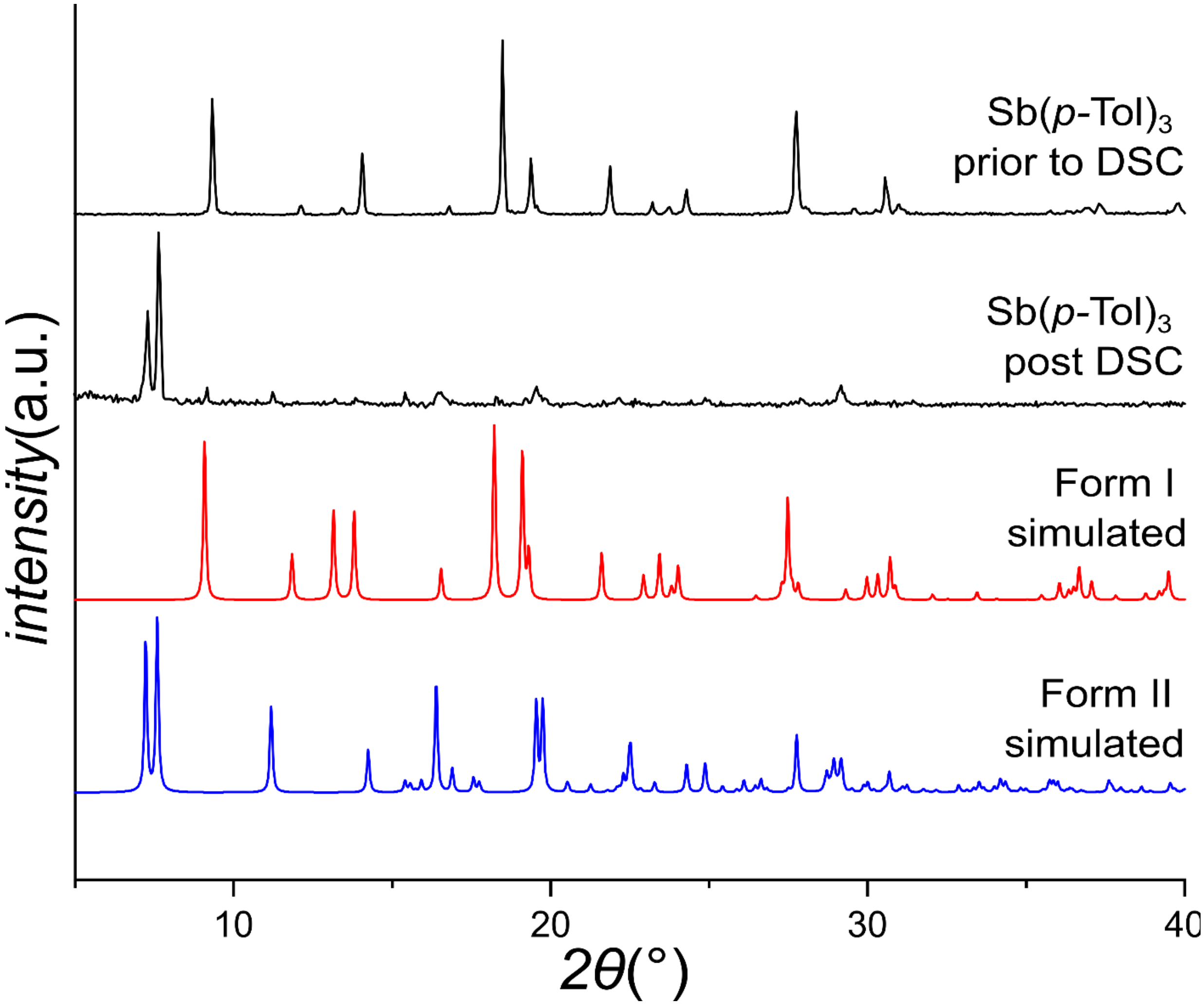

Figure S82. Comparison of PXRD patterns corresponding to a sample of normal Sb(p-Tol)$_3$ before and after 10 DSC cycles (corresponding to Figure S39): the sample before DSC cycling; after DSC cycling; simulated for the crystal structure of Form I and simulated for the crystal structure of Form II.

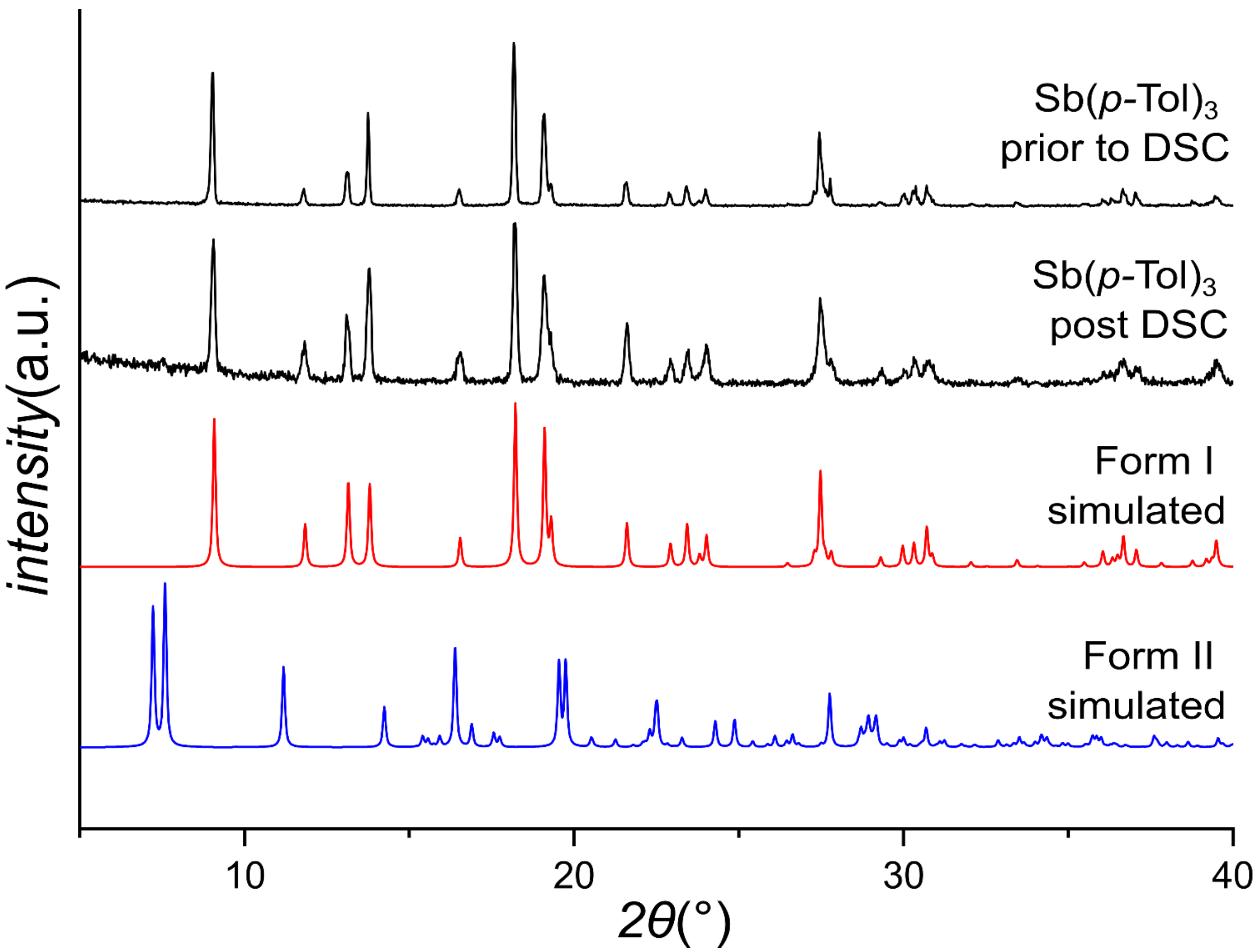

Figure S83. Comparison of PXRD patterns corresponding to a sample of re-crystallized Sb(*p*-Tol)$_3$ before and after 20 DSC cycles (corresponding to Figure S51): the sample before DSC cycling; after DSC cycling; simulated for the crystal structure of Form I and simulated for the crystal structure of Form II.

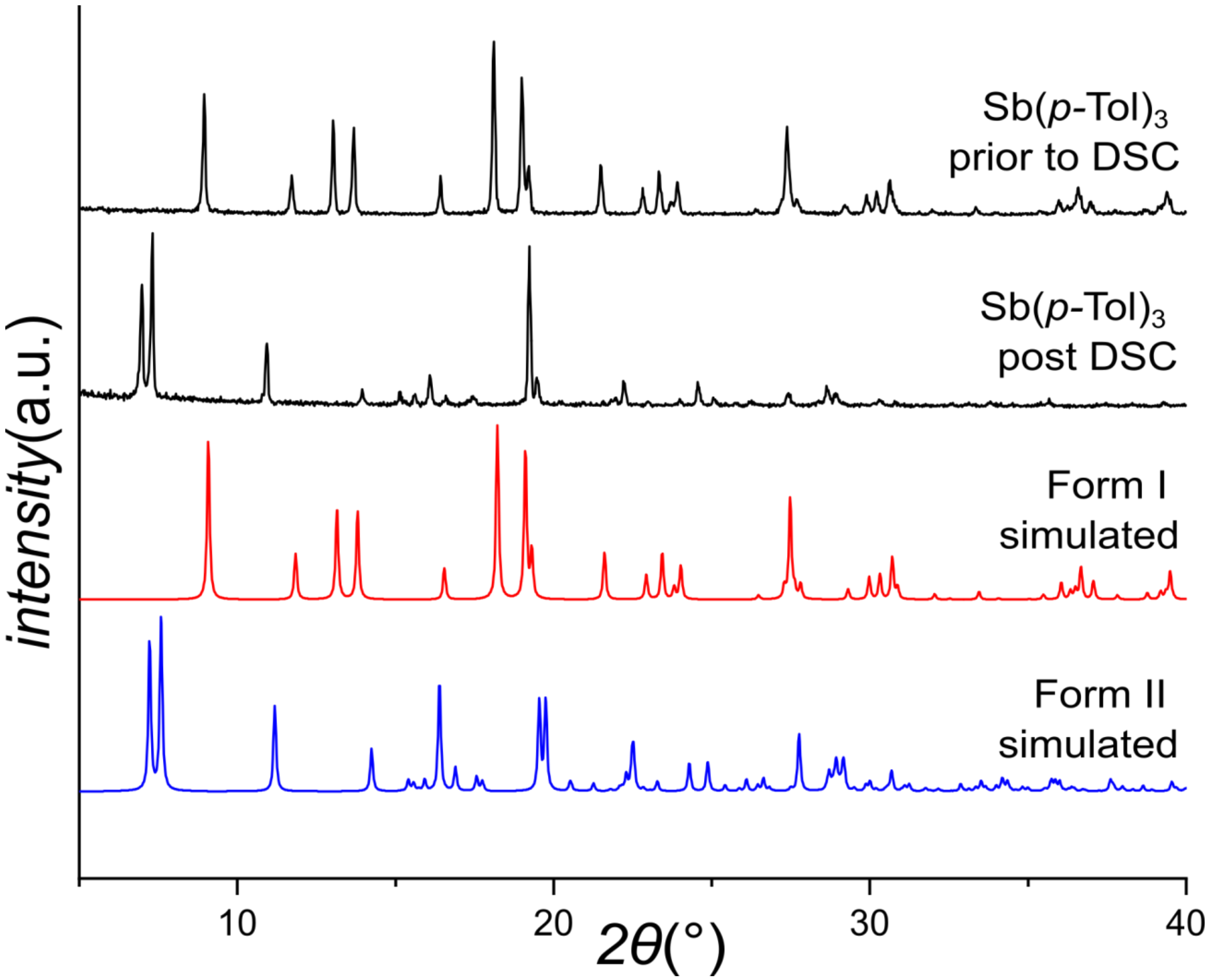

Figure S84. Comparison of PXRD patterns corresponding to a sample of crude Sb(*p*-Tol)$_3$ before and after 10 DSC cycles (corresponding to Figure S56): the sample before DSC cycling; after DSC cycling; simulated for the crystal structure of Form I and simulated for the crystal structure of Form II.

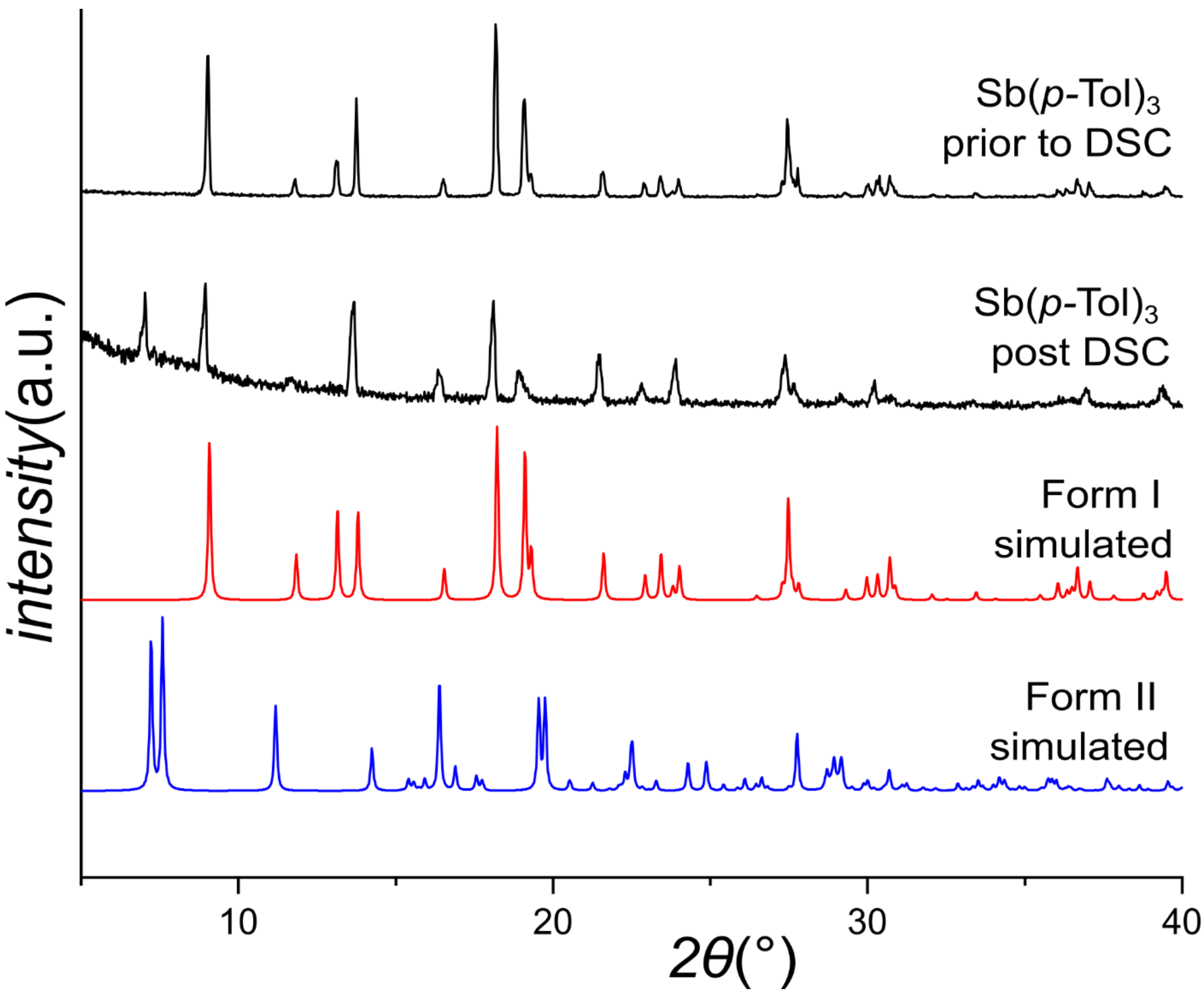

Figure S85. Comparison of PXRD patterns corresponding to a sample of re-crystallized Sb(*p*-Tol)$_3$ in the presence of introduced silicon oil impurity before and after 10 DSC cycles (corresponding to Figure S61): the sample before DSC cycling; after DSC cycling; simulated for the crystal structure of Form I and simulated for the crystal structure of Form II.

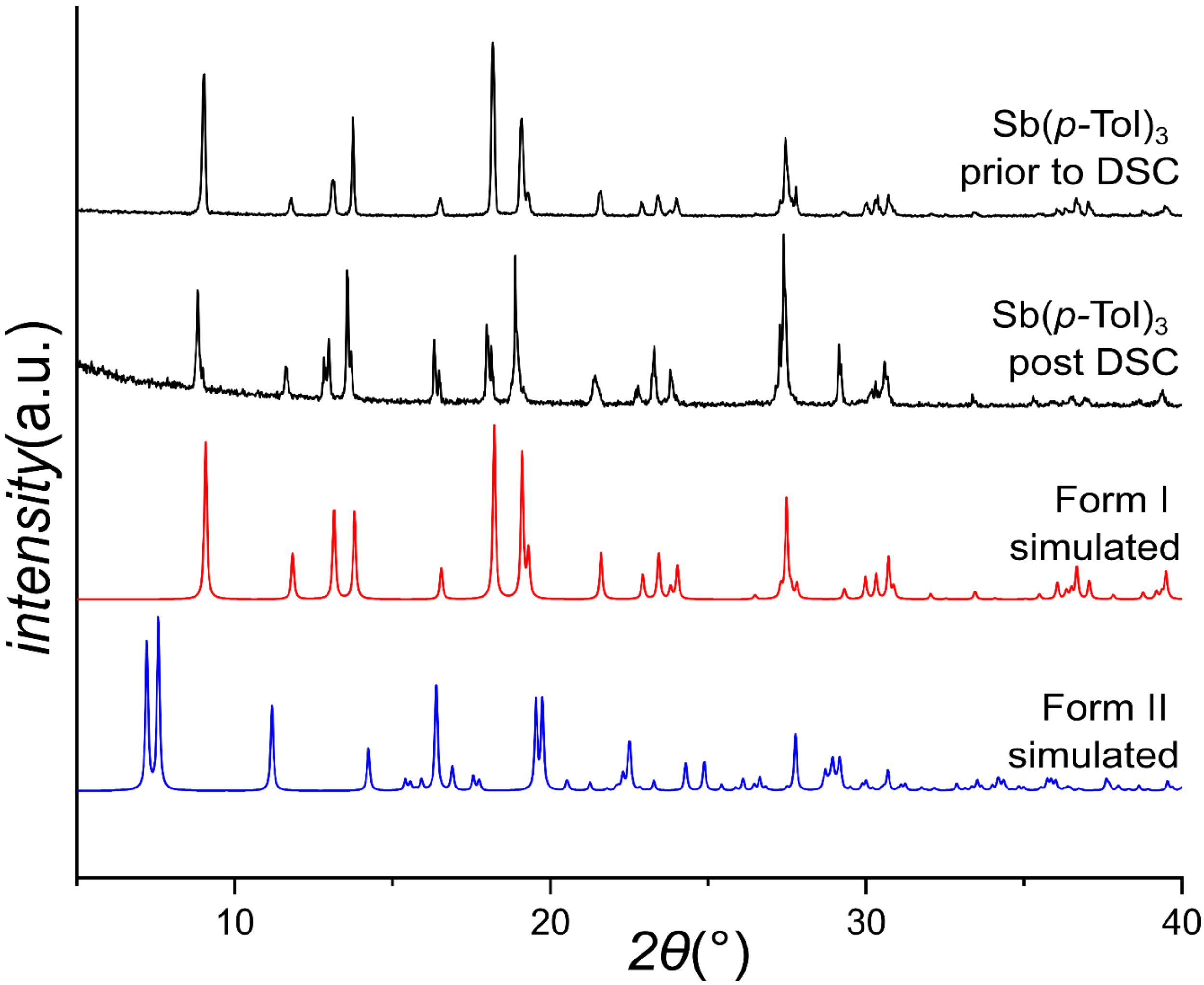

Figure S86. Comparison of PXRD patterns corresponding to a sample of re-crystallized Sb(*p*-Tol)$_3$ in the presence of a vacuum grease impurity before and after 10 DSC cycles (corresponding to Figure S64): the sample before DSC cycling; after DSC cycling; simulated for the crystal structure of Form I and simulated for the crystal structure of Form II.

Before and after PXRDs of slurry experiments

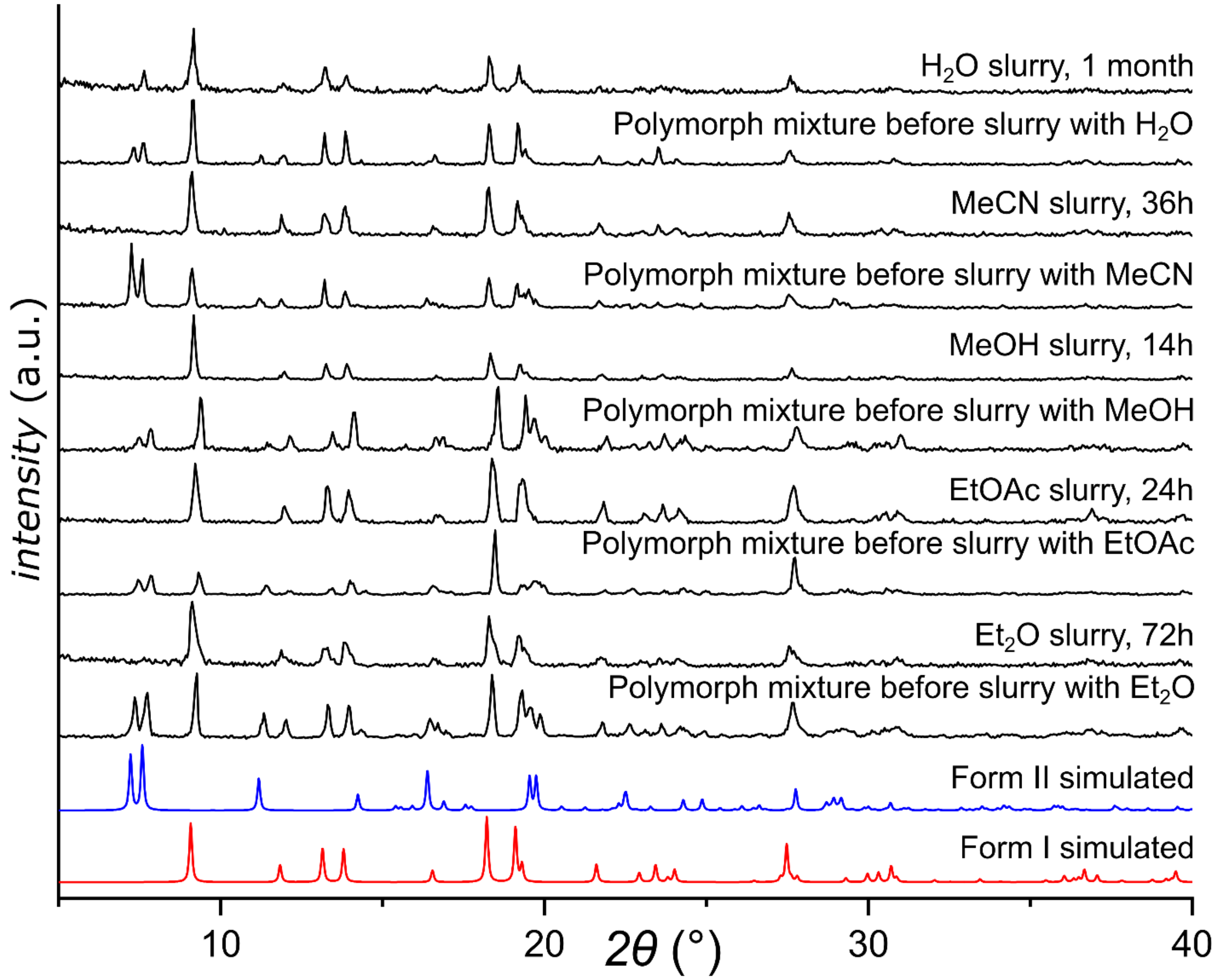

Figure S87. Comparison of PXRD patterns for Sb(*p*-Tol)$_3$ Form I and II mixtures before and after slurrying with a solvent. Slurrying samples were prepared by putting ca. 15 mg of Sb(*p*-Tol)$_3$ in a small vial, heating the material to 140˚C *via* submersion in an oil bath, removing the sample upon complete melting (based on visual observation), and repeating this process ten times. Slurries were prepared using ca. 75 µL of a given solvent, with no samples showing complete dissolution at any point.

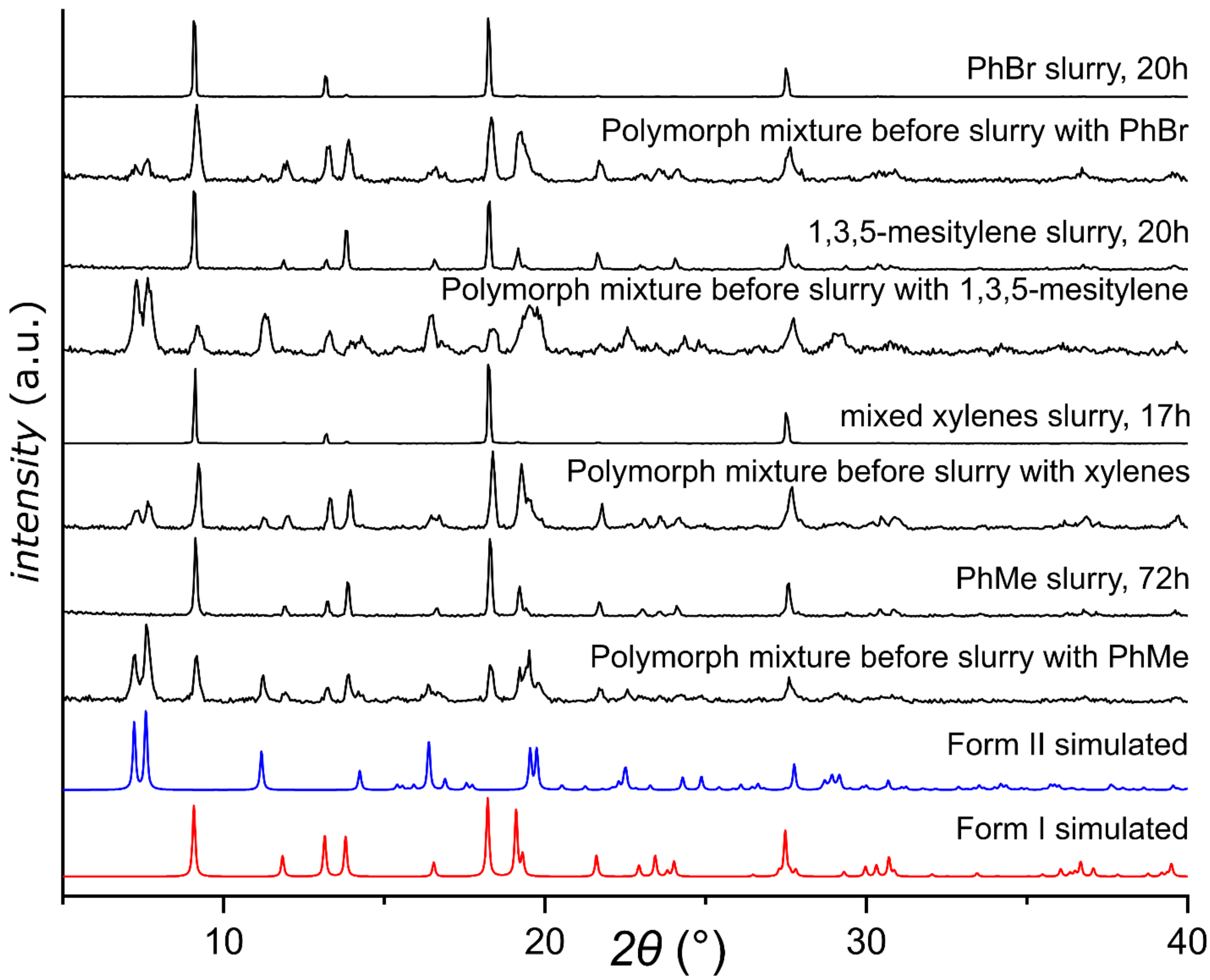

Figure S88. Powder patterns of Sb(*p*-Tol)$_3$ Form I and II mixtures before and after slurrying with an array of solvents. Slurrying samples were prepared by putting ca. 15 mg of Sb(*p*-Tol)$_3$ in a small vial, heating the material to 140˚C *via* submersion in an oil bath, removing the sample upon complete melting (based on visual observation), and repeating this process ten times. Slurries were prepared using ca. 75 µL of a given solvent, with no samples showing complete dissolution at any point.

Cambridge Structural Database Type I structures, I

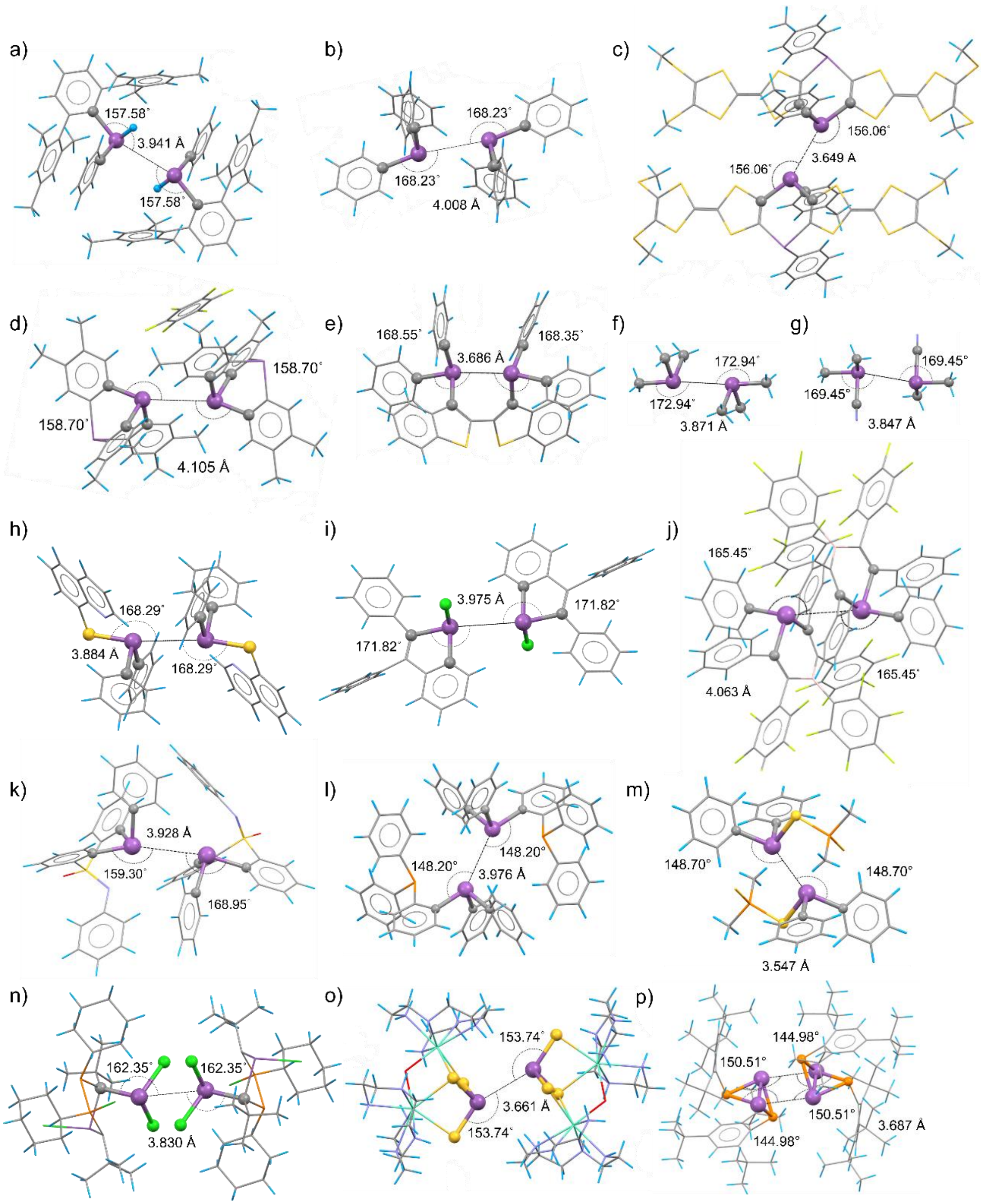

Figure S89. Overview of CSD entries showing dimeric Sb···Sb contacts between trivalent Sb centers. Entries are ordered roughly by atomic mass and complexity of substituents. CCDC reference codes: a) XOGZUP,[59] b) ZZZEHA01,[60] c) KIRKAA,[61] d) KATTIJ,[62] e) YIXPUU,[63] f) GALMIT,[34] g) AFUTAW,[64] h) DURCEY,[65] j) GEHXID,[66] j) JUBQUW,[67] k) BUNWOA,[68] l) VAPNAE,[69] m) RAZPAJ,[70] n) WUJNUK,[71] o) TAMJUO.[72] p) VESCAX.[73]

Cambridge Structural Database Type I structures, II

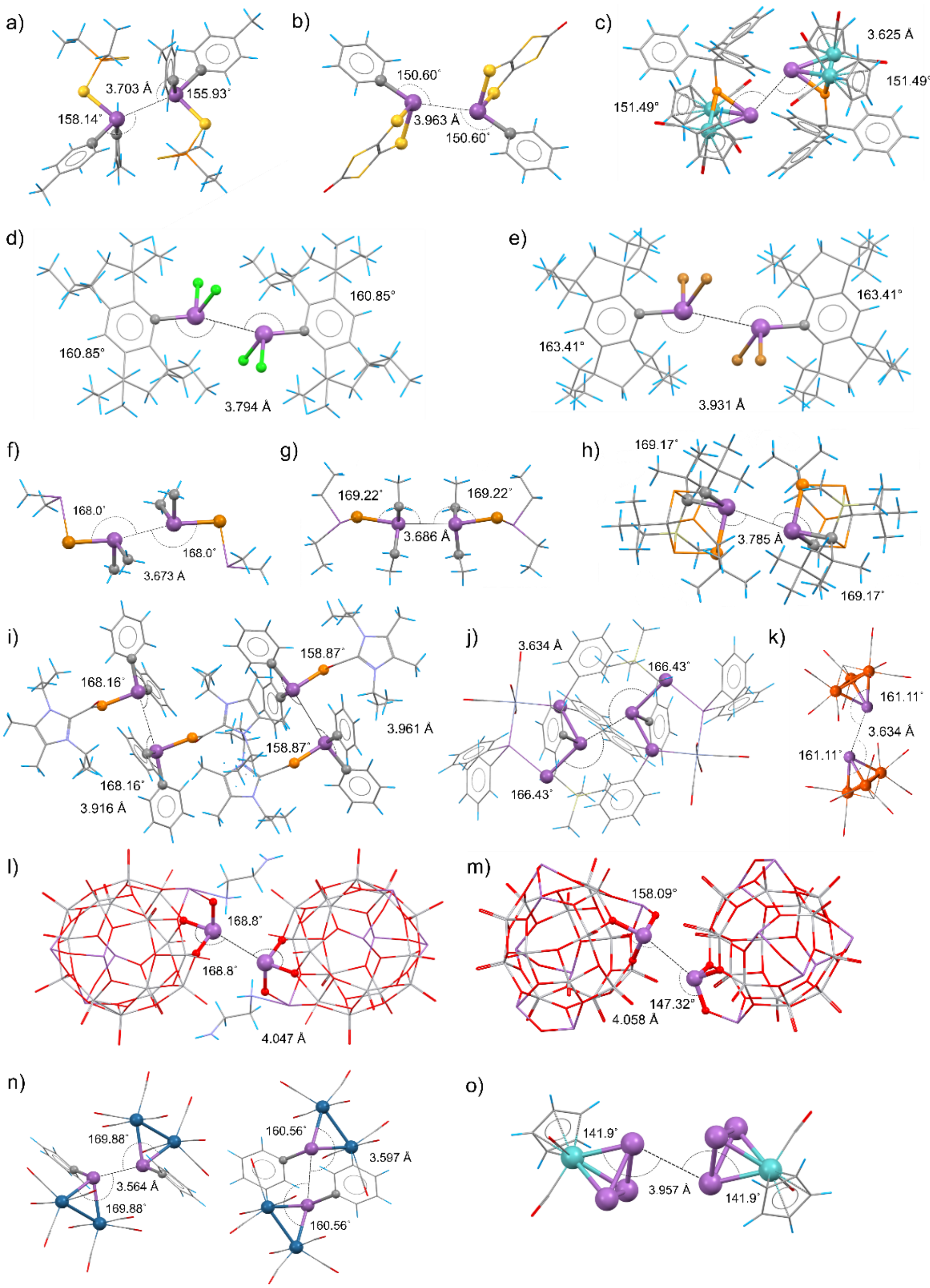

Figure S90. Overview of CSD entries showing dimeric Sb···Sb contacts between trivalent Sb centers. Entries are ordered roughly by atomic mass number and complexity of substituents. CCDC reference codes: a) HAZWOU,[74] b) IHOVEG,[75] c) GIVBAS,[76] d) ELINIA[77], e) ELIROK[77], f) ZIDSUC,[78] g) ZIDTEN,[78] h) QOXMUL,[79] i) INAQUM,[80] j) KAGZIC,[81] k) KEMVON,[82] l) VUVXOD,[83] m) ASEMIU,[84] n) CARVUP.[85] o) NAXJUR.[86]

Cambridge Structural Database Type I structures, III

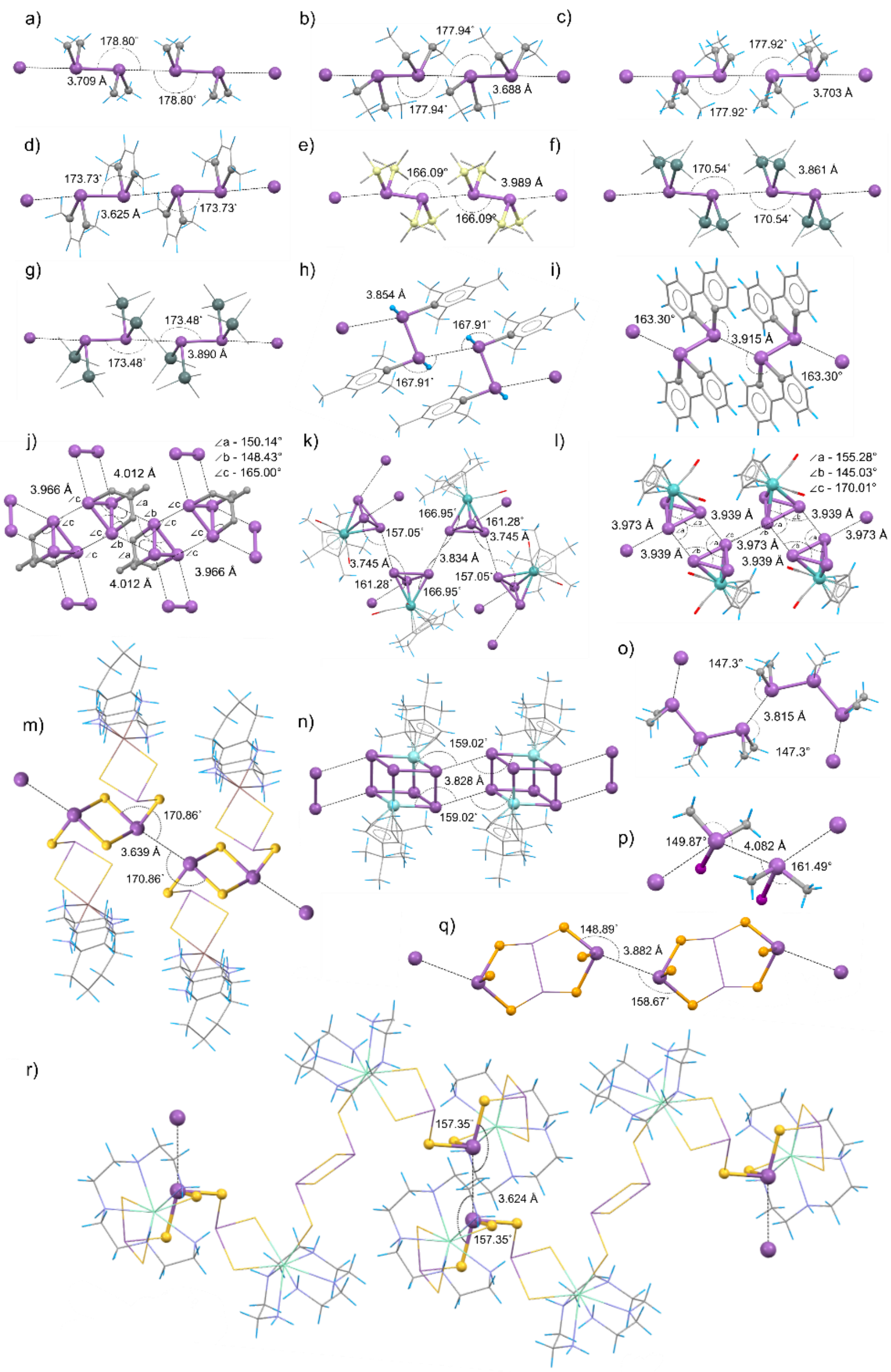

Figure S91. Overview of CSD entries showing chains, nets or other extended bonding arrangements involving short Sb···Sb contacts between trivalent Sb centers. Entries are ordered roughly by atomic mass number of direct substituents. CCDC codes: a) CEPDAC,[87] b) GAVKOF,[88] c) GAVKOF01,[89] d) TMEDSB,[90] e) BOJFAH,[91] f) SEHPAW,[92] g) FOFVAX,[93] h) YAFSOQ,[94] i) GIHWED,[95] j) DEMXOI,[96] k) NAXKAY,[86] l) NAXJUR,[86] m) MUVSIG,[97] n) EWACIR,[98] o) TAXVIX,[99] p) DOQWEL,[100] q) ZAQRUE,[101] r) NIDGUE.[102]

Cambridge Structural Database Type II Structures

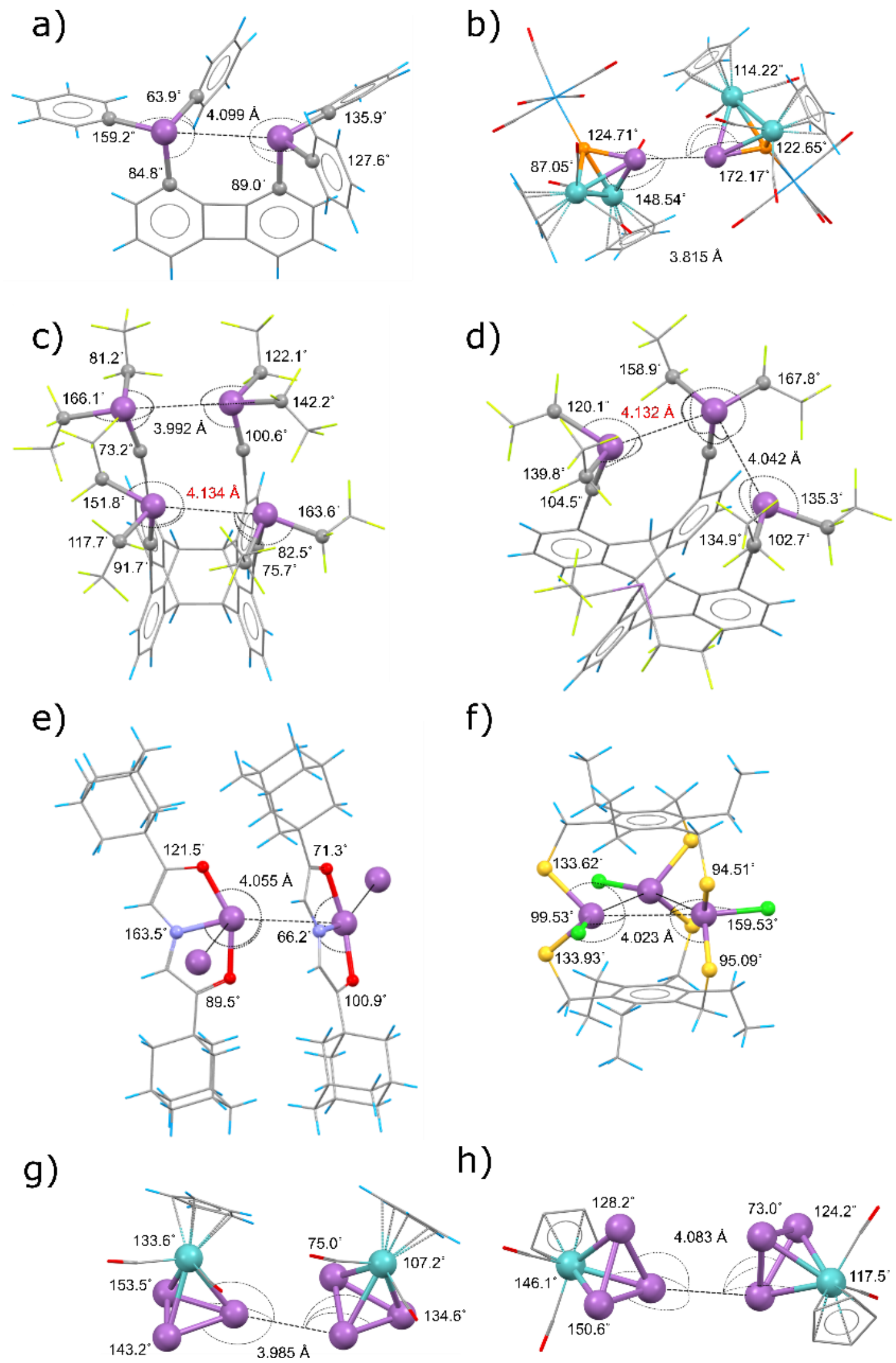

Figure S92. Overview of CSD entries showing Sb···Sb contacts categorized as Type II. Entries c) and d) each notably possess two symmetrically distinct Sb···Sb contacts, as well as g) and h) which are two separate views from one entry. Entries c) and d) each possess a contact longer than the sum of van der Waals radii of two antimony atoms used in the CSD search (4.12 Å),[103] but shorter than the sum of radii recommended by Alvarez (4.94 Å).[104] This pair of longer contacts, highlighted in red, still aligns with the geometrical categorization for a Type II Sb···Sb interaction. Entry e) is planar, which means that angular constraints are distorted when compared to a trigonal pyramidal geometry. Entry f) possesses three sets of identical contacts, for clarity, only one instance is fully labelled. CCDC codes: a) IFUSOU,[105] b) MEFQUJ,[106] c) QOBDEU,[107] d) QOBDAQ,[107] e) FEDMIK,[108] f) YUBZEC.[109] g) and h) NAXJUR[86].

Cambridge Structural Database Miscellaneous Structures

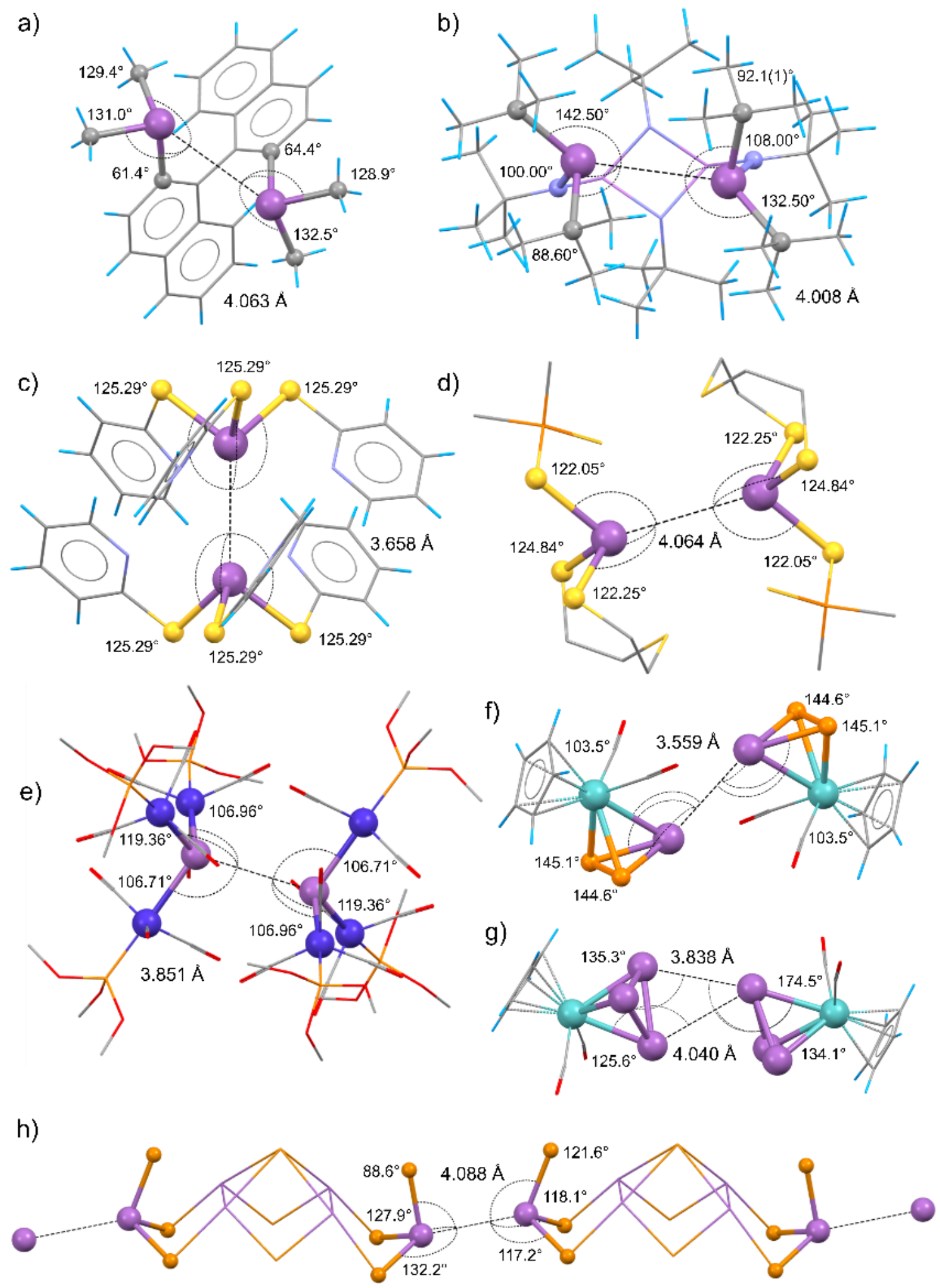

Figure S93. Eight hits categorized as "miscellaneous", as they present a Sb···Sb pair closer than 4.12 Å but with angles that do not fit either of the proposed Type I or II geometries. CCDC codes: a) VOLWID;[110] b) YATMAL[111], c) CIDWIV,[112] d) ROLTIV,[113] e) KUJNEI,[114] f) TUYFAY[115], g) NAXJUR[86], and h) LEXSUC.[116]

References

1. Bruker, *APEX3*, Bruker AXS Inc., Madison, Wisconsin, USA, **2012**.
2. L. Krause, R. Herbst-Irmer, G. M. Sheldrick, D. Stalke, Comparison of silver and molybdenum microfocus X-ray sources for single-crystal structure determination. *J. Appl. Cryst.* **2015**, *48*, 3–10.
3. G. M. Sheldrick, SHELXT – Integrated space-group and crystal-structure determination. *Acta. Cryst.* **2015**, *A71*, 3–8.
4. G. M. Sheldrick, Crystal structure refinement with SHELXL. *Acta. Cryst.* **2015**, *C71*, 3–8.
5. O. V. Dolomanov, L. J. Bourhis, R. J. Gildea, J. A. K. Howard, H. Puschmann, OLEX2: a complete structure solution, refinement and analysis program. *J. Appl. Cryst.* **2009**, *42*, 339–341.
6. L. J. Farrugia, WinGX and ORTEP for Windows: an update. *J. Appl. Cryst.* **2012**, *45*, 849–854.
7. C. F. Macrae, I. Sovago, S. J. Cottrell, P. T. A. Galek, P. McCabe, E. Pidcock, M. Platings, G. P. Shields, J. S. Stevens, M. Towler, P. A. Wood, "*Mercury 4.0* : from visualization to analysis, design and prediction", *J. Appl. Cryst.* **2020**, *53*, 226–235.
8. Persistence of Vision Pty. Ltd. *Persistence of Vision Raytracer*. (Persistence of Vision Pty. Ltd., **2018**).
9. H. M. A. Rietveld, A profile refinement method for nuclear and magnetic structures. *J. Appl. Crystallogr.*, **1969**, *2*, 65–71.
10. A. A. Coelho, *TOPAS* and *TOPAS-Academic*: an optimization program integrating computer algebra and crystallographic objects written in C++. *J. Appl. Crystallogr.* **2018**, *51*, 210-218.
11. P. R. Spackman, M. J. Turner, J. J. McKinnon, S. K. Wolff, D. J. Grimwood, D. Jayatilaka, M. A. Spackman, *CrystalExplorer*: a program for Hirshfeld surface analysis, visualization and quantitative analysis of molecular crystals. *J. Appl. Cryst.* **2021**, *54*, 1006-1011.
12. C. F. Mackenzie, P. R. Spackman, D. Jayatilaka, M. A. Spackman, *CrystalExplorer* model energies and energy frameworks: extension to metal coordination compounds, organic salts, solvates and open-shell systems *IUCrJ*, **2017**, *4*, 575-587.
13. W. J. Hehre, R. F. Stewart, J. A. Pople, Self-Consistent Molecular-Orbital Methods. I. Use of Gaussian Expansions of Slater-Type Atomic Orbitals. *J. Chem. Phys.*, **1969**, *51*, 2657-2664.
14. J. B. Collins, P. von R. Schleyer, J. S. Binkley, J. A. Pople, Self-Consistent Molecular-Orbital Methods. XVII. Geometries and binding energies of second-row molecules. A comparison of three basis sets. **1976**, *64*, *J. Chem. Phys*, 5142-5151.
15. A. D. Becke, Density-Functional Thermochemistry. III. The Role of Exact Exchange. *J. Chem. Phys.*, **1993**, *98*, 5648 – 5652.
16. C. Lee, W. Yang, R. G. Parr, Development of the Colle-Salvetti correlation-energy formula into a functional of the electron density. *Phys. Rev. B. Condens. Matter.*, **1988**, *37*, 785-789.
17. S. H. Vosko, L. Wilk, M. Nusair, Accurate spin-dependent electron liquid correlation energies for local spin density calculations: a critical analysis. *Can. J. Phys.*, **1980**, *58*, 1200 – 1211.

18. P. J. Stephens, F. J. Devlin, C. F. Chabalowski, M. J. Frisch, Ab Initio Calculation of Vibrational Absorption and Circular Dichroism Spectra Using Density Functional Force Fields. *J. Phys. Chem.*, **1994**, *98*, 11623 – 11627.
19. D. Jayatilaka, D. J. Grimwood, Tonto: A Fortran Based Object-Oriented System for Quantum Chemistry and Crystallography. In: P. M. A. Sloot, D. Abramson, Y. E. Gorbachev, J. J. Dongarra, A. Y. Zomaya, (eds) Computational Science – ICCS 2003. **2003**, *2660*, Springer, Berlin, Heidelberg.
20. S. Grimme, Semiempirical GGA-type density functional constructed with a long-range dispersion correction. *J. Comp. Chem.*, **2006**, *27*, 1787 – 1799.
21. C. R. Groom, I. J. Bruno, M. P. Lightfoot, S. C. Ward, The Cambridge Structural Database. *Acta Cryst.* **2016**, *B72*, 171–179.
22. B. Lindquist-Kleissler, T. C. Johnstone, Models of the Putative Antimony(v)–Diolate Motifs in Antileishmanial Pentavalent Antimonial Drugs. *Dalton Trans.* **2023**, *52*, 9229–9237.
23. S. J. Clark, *et al.* First principles methods using CASTEP. *Z. Kristallogr.* **2005**, *220*, 567–570.
24. T. Björkman, CIF2Cell: Generating geometries for electronic structure programs. *Comput. Phys. Commun.* **2011**, *182*, 1183–1186.
25. H. J. Monkhorst, J. D. Pack, Special points for Brillouin-zone integrations. *Phys. Rev. B* **1976**, *13*, 5188–5192.
26. J. P. Perdew, K. Burke, M. Ernzerhof, Generalized Gradient Approximation Made Simple. *Phys. Rev. Lett.* **1996**, *77*, 3865–3868.
27. S. Grimme, J. Antony, S. Ehrlich, H. A. Krieg, Consistent and Accurate Ab Initio Parametrization of Density Functional Dispersion Correction (DFT-D) for the 94 Elements H-Pu. *J. Chem. Phys.* **2010**, *132*, 154104.
28. S. Grimme, S. Ehrlich, L. Goerigk, Effect of the Damping Function in Dispersion Corrected Density Functional Theory. *J. Comput. Chem.* **2011**, *32*, 1456–1465.
29. E. Caldeweyher, C. Bannwarth, S. Grimme, Extension of the D3 Dispersion Coefficient Model. *J. Chem. Phys.* **2017**, *147*, 034112.
30. E. Caldeweyher, S. Ehlert, A. Hansen, H. Neugebauer, S. Spicher, C. Bannwarth, S. Grimme, A Generally Applicable Atomic-Charge Dependent London Dispersion Correction. *J. Chem. Phys.* **2019**, *150*, 154122.
31. E. Caldeweyher, J,-M. Mewes, S. Ehlert, S. Grimme, Extension and Evaluation of the D4 London-Dispersion Model for Periodic Systems. *Phys. Chem. Chem. Phys.* **2020**, *22*, 8499–8512.
32. W. Kohn, L. J. Sham, 'Self-Consistent Equations Including Exchange and Correlation Effects' *Phys. Rev.* **1965**, *140*, A1133–A1138.
33. Wagner, J. P.; Schreiner, P. R. London Dispersion in Molecular Chemistry—Reconsidering Steric Effects. *Angew. Chem. Int. Ed.* **2015**, 54, 12274–12296.
34. Bujak, M.; Stammler, H.-G.; Vishnevskiy, Y. V.; Mitzel, N. W. Very Close I···As and I···Sb Interactions in Trimethylpnictogen-Pentafluoroiodobenzene Cocrystals. *CrystEngComm* **2022**, 24, 70–76.
35. Zahn, S.; Frank, R.; Hey-Hawkins, E.; Kirchner, B. Pnicogen Bonds: A New Molecular Linker? *Chem. Eur. J.* **2011**, 17, 6034–6038.
36. R. F. W. Bader, A quantum theory of molecular structure and its applications. *Chem. Rev.* **1991**, *91*, 893-928.

37. E. Espinosa, M. Souhassou, H. Lachekar, C. Lecomte, Topological analysis of the electron density in hydrogen bonds. *Struct. Sci.* **1999**, *55*, 563-572.
38. E. R. Johnson, S. Keinan, P. Mori-Sánchez, J. Contreras-García, A. J. Cohen, W. Yang, Revealing noncovalent interactions. *J. Am. Chem. Soc.* **2010**, *132*, 6498-6506.
39. L. Zhang, Q. H. Zhu, Y. R. Zhou, S. L. Wang, J. Fu, J. Y. Liu, G. H. Zhang, L. Ma, G. Tao, G. H. Tao, L. He, Hydrogen-bonding and π-π interaction promoted solution-processable covalent organic frameworks. *Nat. Commun.* **2023**, *14*, 8181.
40. M. J. Rutter, C2x: A tool for visualisation and input preparation for Castep and other electronic structure codes. *Comput. Phys. Commun.* **2018**, *225*, 174-179.
41. A. Otero-de-la-Roza, M. A. Blanco, A. M. Pendás, V. Luaña, Critic: A New Program for the Topological Analysis of Solid-State Electron Densities. *Comput. Phys. Commun.* **2009**, *180*, 157–166.
42. A. Otero-de-la-Roza, E. R. Johnson, V. Luaña, Critic2: A Program for Real-Space Analysis of Quantum Chemical Interactions in Solids. *Comput. Phys. Commun.* **2014**, *185*, 1007–1018.
43. A. Otero-de-la-Roza, Finding critical points and reconstruction of electron densities on grids. *J. Chem. Phys.* **2022**, *156*, 224116.
44. Neese, F. The ORCA Program System. *WIREs Comput. Mol. Sci.* **2011**, 2, 73–78.
45. Neese, F. Software Update: The ORCA Program System—Version 6.0. *WIREs Comput. Mol. Sci.* **2025**, 15, e70019
46. Neese, F. The SHARK Integral Generation and Digestion System. *J. Comput. Chem.* **2022**, 44, 381–396.
47. Weigend, F.; Ahlrichs, R. Balanced Basis Sets of Split Valence, Triple Zeta Valence and Quadruple Zeta Valence Quality for H to Rn: Design and Assessment of Accuracy. *Phys. Chem. Chem. Phys.* **2005**, 7, 3297-3305.
48. Weigend, F. Accurate Coulomb-Fitting Basis Sets for H to Rn. *Phys. Chem. Chem. Phys.* **2006**, 8, 1057-1065.
49. Metz, B.; Stoll, H.; Dolg, M. Small-Core Multiconfiguration-Dirac–Hartree–Fock-Adjusted Pseudopotentials for Post- d Main Group Elements: Application to PbH and PbO. *J. Chem. Phys.* **2000**, 113, 2563–2569.
50. Humphrey, W.; Dalke, A.; Schulten, K. VMD: Visual Molecular Dynamics. *J. Mol. Graphics* **1996**, 14, 33–38.
51. Connolly, M. L. Analytical Molecular Surface Calculation. *J. Appl. Crystallogr.* **1983**, 16, 548–558.
52. M. Wada, S. Natsume, S. Suzuki, U. Akira, M. Nakamura, S. Hayase and T. Erabi, Two-step synthesis of triarylmetals (As, Sb, Bi) starting from the metal oxides and 2,6-dimethoxybenzenethiol, *J. Organomet. Chem.*, **1997**, *548*, 223–227.
53. H. F. Grützmacher, D. Kirchhoff, Trends in the Unimolecular Reactions of Triaryl Pnictogen Radical Cations $Ar_3E^{\bullet +}$ (E = N, P, As, Sb, Bi): Reductive Elimination versus E-C Bond Cleavage. *Organometallics*, **2001**, *20*, 3738–3744.
54. D. Hasa, E. Carlino, W. Jones, Polymer-Assisted Grinding, a Versatile Method for Polymorph Control of Cocrystallization, *Cryst. Growth Des.* **2016**, *16*, 1772-1779.
55. D. Zheltikova, E. Losev, E. Boldyreva, To touch or not to touch? Fingerprint-assisted grinding of carbamazepine form III, *CrystEngComm* **2023**, *25*, 4879-4888.

56. A. Rivalta, T. Salzillo, E. Venuti, R. G. Della Valle, B. Sokolovič, O. Werzer, A. Brillante, Bulk and Surface-Stabilized Structures of Paracetamol Revisited by Raman Confocal Microscopy, *ACS Omega* **2018**, *3*, 9564-9571.
57. P. Di Martino, P. Conflant, M. Drache, J. P. Huvenne, A. M. Guyot-Hermann, Preparation and physical characterization of forms II and III of paracetamol, *J. Therm. Anal.* **1997**, *48*, 447-458.
58. J. C. Burley, M. J. Duer, R. S. Stein, R. M. Vrcelj, Enforcing Ostwald's rule of stages: Isolation of paracetamol forms III and II, *Eur. J. Pharm. Sci.* **2007**, *31*, 271-276.
59. H. J. Breunig, E. Lork, O. Moldovan, C. I. Raţ, Syntheses of a stable tristibine and of related antimony compounds with the 2,6-dimesitylphenyl (Dmp) substituent. *J. Organomet. Chem.* **2008**, *693*, 2527-2534.
60. E. A. Adams, J. W. Kolis, W. T. Pennington, Structure of triphenylstibine. *Acta Cryst* **1990**, *C46*, 917-919.
61. M. Hasegawa, M. Shirai, Y. Mazaki, Tetrathiafulvalene Dimer Merged with a Binuclear Ring of Sn and Sb: Synthesis and Molecular Structures Induced by Heteroatoms. *Eur. J. Inorg. Chem.* **2018**, 4084-4092.
62. Y. Uchiyama, G. A. Yamamoto, Bromine Adduct of 2,3,6,7,14,15-Hexamethyl-9,10-distibatriptycene. The First Stibonium–Stiborate Zwitterion. *Chem. Lett.* **2005**, *34*, 966-967.
63. C.-H. Wang, A. Sumida, Y. Adachi, H. Imoto, K. Naka, J. Ohshita, Group 15 Element (As, Sb, Bi)-Substituted Bibenzofurans with Noncovalent Conformational Locks for Enhanced Planarity. *Organometallics* **2023**, *42*, 3397-3404.
64. D. Britton, V. G. Young, E. O. Schlemper, Intermolecular interactions in cyanodimethylarsine and cyanodimethylstibine. *Acta Cryst.* **2002**, *C58*, m307-m309.
65. H. Preut, U. Praeckel, F. Huber, F. Diphenyl[8-quinolinethiolato(1-)-S]antimony(III). *Acta Cryst.* **1986**, *C42*, 1138-1140.
66. A. M. Christianson, F. P. Gabbaï, A Lewis Acidic, π-Conjugated Stibaindole with a Colorimetric Response to Anion Binding at Sb(III). *Organometallics* **2017**, *36*, 3013-3015.
67. J. Culvyhouse, D. K. Unruh, H. Lischka, A. J. A. Aquino, C. Krempner, Facile Access to Organostibines via Selective Organic Superbase Catalyzed Antimony-Carbon Protonolysis. *Angew. Chem., Int. Ed.* **2024**, *63*, e202407822.
68. K. Mizuta, H. Shimoji, T. Fujii, S. Yamamoto, H. Ohkita, K. Naka, H. Imoto, Pnictogen-Bridged Sulfoximines: Effects of Element and N-Substituent on Excited-State Dynamics. *Chem. Photo. Chem.* **2025**, *9*, e202400336.
69. J. S. Jones, F. P. Gabbai, Activation of an Au−Cl Bond by a Pendent Sb^{III} Lewis Acid: Impact on Structure and Catalytic Activity. *Chem. Eur. J.* **2017**, *23*, 1136-1144.
70. M. N. Gibbons, D. B. Sowerby, C. Silvestru, I. Haiduc, Reduction of antimony(V) by dithiophosphinates and the crystal structure of dimeric diphenylantimony(III) dimethyldithiophosphinate. *Polyhedron* **1996**, *15*, 4573-4578.
71. C. Jones, P. C. Junk, A. F. Richards, M. Waugh, The interaction of phosphavinyl Grignard reagents with group 15 halides: synthesis and structural characterisation of novel heterocyclic and heterocage compounds. *New J. Chem.* **2002**, *26*, 1209-1215.
72. J. Zhou, L. An, X. Liu, H. Zou, F. Hu, C. Liu, One unprecedented 1-D europium thioindate-thioantimonate based on heterometallic mixed nitro-thioclusters with photoluminescent properties. *Chem. Commun.* **2012**, *48*, 2537-2539.

73. P. Jutzi, U. Meyer, S. Opiela, M. M. Olmstead, P. P. Power, Syntheses and x-ray crystal structures of the compounds $[ArPAs]_2$ and $[ArPSb]_2$ (Ar = 2,4,6-(t-Bu)$_3C_6H_2$. P_2As_2- and P_2Sb_2-substituted analogs of bicyclo[1.1.0]butane. *Organometallics*, **1990**, *9*, 1459-1463.
74. C. Silvestru, I. Haiduc, R. Kaller, K. H. Ebert, H. J. Breunig, Synthesis, spectroscopic characterization and molecular structure of dimeric (diethyldithiophosphinato)DI(*para*-tolyl)antimonyl(III), $[(p\text{-}CH_3C_6H_4)_2SbS_2P(C_2H_5)_2]_2$, containing a novel monocyclic $P_2S_4Sb_2$ inorganic ring system, formed through Sb…S semibonding interactions. *Polyhedron* **1993**, *12*, 2611-2617.
75. N. Avarvari, M. Fourmigué, Neutral and Dianionic Organoantimony(III) Dithiolene Complexes: Syntheses, X-ray Crystal Structures, and Unexpected Reactivity. *Organometallics* **2003**, *22*, 2042-2049.
76. L. Zimmermann, C. Riesinger, G. Balázs, M. Scheer, Synthesis and Reactivity of Hetero-Pnictogen Diazonium Analogs Stabilized by Transition Metal Units. *Chem. Eur. J.* **2023**, *29*, e202301974.
77. Y. Kawase, K. Ota, S. Ikoma, K. Sugahara, T. Nakahodo, Y. Mizuhata, T. Matsuo, Organoantimony(I) Compounds Incorporating the Bulky Rind Groups: Selective Synthesis and Characterization of Distibene and Cyclotetrastibane. *Organometallics* **2025**, *44*, 2240-2249.
78. S. Heimann, S. Schulz, D. Bläser, C. Wölper, Syntheses and Solid-State Structures of Bis(dialkylstibanyl)sulfanes and -telluranes. *Eur. J. Inorg. Chem.* **2013**, 4909-4915.
79. C. Jones, R. C. Thomas, The synthesis and structural characterisation of a diphosphastibolyl potassium complex, $[\{[K(DME)][1,4,2\text{-}P_2SbC_2Bu^t{}_2]\}_\infty]$, and a novel hetero-cage compound. *J. Organomet. Chem.* **2001**, *622*, 61-65.
80. J. E. Walley, L. S. Warring, E. Kertesz, G. Wang, D. A. Dickie, Z. Benko, R. J. Gilliard Jr. Indirect Access to Carbene Adducts of Bismuth- and Antimony-Substituted Phosphaketene and Their Unusual Thermal Transformation to Dipnictines and $[(NHC)_2OCP][OCP]$. *Inorg. Chem.* **2021**, *60*, 4733-4743.
81. H. J. Breunig, I. Ghesner, M. E. Ghesner, E. Lork, Syntheses and coordination chemistry of di-, tri-, and tetrastibanes, $R_2Sb(SbR')_nSbR_2$ (n=0, 1, 2). *J. Organomet. Chem.* **2003**, *677*, 15-20.
82. C. Caballero, B. Nuber, M. L. Ziegler, Darstellung und charakterisierung von trigonal bipyramidalen methylidin-clustern des typs $EFe_3(CO)_9CH$ (E = As, Sb, Bi) durch Umsetzung des Carbidoclusters $Fe_5C(CO)_{15}$ mit metallischem Arsen und Wismut bzw. $SbCl_5$. *J. Organomet. Chem.* **1990**, *386*, 209-223.
83. P. Yi, K.-W. Tong, C.-Q. Chen, Y. Zhang, J. Du, and P. Yang,Switchable Iodine Adsorption Management in Polyoxovanadate−Cucurbit[6]uril-Based Supramolecular Framework Materials. *Chem. Mater.* **2025**, *37*, 3542-3554.
84. X.-X. Hu, J.-Q. Xu, X.-B. Cui, J.-F. Song, T.-G. Wang, A novel one-dimensional framework material constructed from antimony-substituted polyoxovanadium clusters: $[(C_2N_2H_{10})_2\beta\text{-}\{Sb^{III}{}_8V^{IV}{}_{14}O_{42}(H_2O)\}](C_2N_2H_8)\cdot 4H_2O$. *Inorg. Chem. Comm.* **2004**, *7*, 264-267.
85. Y.-Z. Li, R. Ganguly, W. K. Leong, Expedient Synthesis of a Metallostibine $Os_2(CO)_8(\mu\text{-}SbPh)$: An Unusual and Strong Two-Electron-Donor Ligand. *Eur. J. Inorg. Chem.* **2017**, *2017*, 2541-2546.

86. H. J. Breunig, R. Rösler, E. Lork, Complexes with Sb_2 and cyclo-Sb_3 Ligands: The Tetrahedranes [{$C_5H_5(CO)_2Mo$}$_2Sb_2$], [$C_5H_5(CO)_2MoSb_3$], and [$C_5Me_5(CO)_2MoSb_3$]. *Angew. Chem. Int. Ed.* **1997**, *36*, 2819-2821.
87. O. Mundt, H. Riffel, G. Becker, A. Simon, Element—Element-Bindungen, III [1]. Intermolekulare Sb···Sb-Wechselwirkungen im kristallinen Tetramethyldistiban[1] / Element—Element Bonds, III [1]. Intermolecular Sb···Sb Interactions in Crystalline Tetramethyldistibane. *Z. Naturforsch. B* **1984**, *39*, 317-322.
88. A. Kuczkowski, S. Heimann, A. Weber, S. Schulz, D. Bläser, C. Wölper, Structural Characterization of Et_4Sb_2 and Et_4Bi_2. *Organometallics* **2011**, *30*, 4730-4735.
89. S. Schulz, S. Heimann, A. Kuczkowski, D. Bläser, C. Wölper, The Origin of Thermochromic Behavior in Distibines: Still an Open Question. *Organometallics* **2013**, *32*, 3391−3394.
90. A. J. Ashe, W. Butler III, T. R. Diephouse, 2,2',5,5'-Tetramethyldistibolyl. A thermochromic distibine. *J. Am. Chem. Soc.* **1981**, *103*, 207-209.
91. G. Becker, H. Freudenblum, C. Witthauer, Trimethylsilylverbindungen der Vb-Elemente. VI. Synthese, Molekül- und Kristallstruktur des Tetrakis(trimethylsilyl)distibans im Vergleich mit Tetraphenyldistiban. *Z. Anorg. Allg. Chem.* **1982**, *492*, 37-51.
92. S. Roller, M. Dräger, H. J. Breunig, M. Ateş, S. Güleş, Kristallstruktur, thermische Ausdehnung und Farbwechsel von $(Me_3Ge)_2Sb$-$Sb(GeMe_3)_2$ und anderen Distibanen. *J. Organomet. Chem.* **1989**, *378*, 327-337.
93. S. Roller, M. Dräger, H. J. Breunig, M. Ates, S. Gülec, $(Me_3Sn)_2Sb$–$Sb(SnMe_3)_2$,$^1{}_\infty$-verknüpfte Sb–Sb-Hanteln in einem thermochromen Distiban mit Schweratomgerüst. *J. Organomet. Chem.* **1987**, *329*, 319-326.
94. T. Pugh, N. F. Chilton, R. A. Layfield, Antimony-ligated dysprosium single-molecule magnets as catalysts for stibine dehydrocoupling. *Chem. Sci.* **2017**, *8*, 2073-2080.
95. D. Zhang, T. Tang, Z. Zhang, L. Le, Z. Xu, H. Lu, Z. Tong, D. Zeng, W.Y. Wong, S.-F. Yin, *et al.* Nickel- and Palladium-Catalyzed Cross-Coupling of Stibines with Organic Halides: Site-Selective Sequential Reactions with Polyhalogenated Arenes. *ACS Catal.* **2022**, *12*, 854-867.
96. J. Ellermann, E. Kock, H. Burzlaff, Structure of 4-methyl-1,2,6-tristibatricyclo[2.2.1.0^{2,6}]heptane, $C_5H_9Sb_3$. *Acta Cryst.* **1985**, *C41*, 1437-1439.
97. E. Quiroga-González, C. Näther, W. Bensch, The thioantimonate anion $[Sb_4S_8]^{4-}$ acting as a tetradentate ligand: Solvothermal synthesis, crystal structure and properties of {$[In(C_6H_{14}N_2)_2]_2Sb_4S_8$}$Cl_2$ exhibiting unusual uniaxial negative and biaxial positive thermal expansion. *Solid State Sci.* **2010**, *12*, 1235-1241.
98. V. Heinl, A. E. Seitz, G. Balázs, M. Seidl, M. Scheer, Transfer of polyantimony units. *Chem. Sci.* **2021**, *12*, 9726-9732.
99. Breunig, H. J.; Denker, M.; Lork, E. A Trinuclear Organoantimony Cation: Structure of [Me_2Sb–$Sb(Me_2)$–$SbMe_2$][Me_2SbBr_2]. *Angew. Chem. Int. Ed.* **1996**, *35*, 1005-1006.
100. H. J. Breunig, H. Althaus, R. Rösler, E. Lork, Neue Synthesen von Me_2SbX (X=Cl, I) und Kristallstrukturen von Me_2SbI und $[(Me_3Si)_2CH]_2SbCl$. *Z. Anorg. Allg. Chemie* **2000**, *626*, 1137-1140.

101. S. Sportouch, J. Thumim, M. Tillard-Charbonnel, C. Belin, Heteroatomic polyanions of post-transition elements. Synthesis and structure of a salt containing the novel tetra antimony hexaselenate (2-) anion $[Sb_4Se_6]^{2-}$. *Nouv. J. Chim.* **1995**, *19*, 133-135.
102. J. Zhou, F. Hu, L. An, X. Liu, C.-Y. Meng, Novel lanthanoid thioantimonates: the first coexistence of different types of thioantimonate anions in the same framework. *Dalton Trans.* **2012**, *41*, 11760-11764.
103. M. Mantina, A. C. Chamberlin, R. Valero, C. J. Cramer, D. G. Truhlar, Consistent van der Waals Radii for the Whole Main Group, *J. Phys. Chem. A* **2009**, *113*, 5806−5812.
104. S. Alvarez, A cartography of the van der Waals territories, *Dalton Trans.* **2013**, *42*, 8617−8636.
105. C.-H. Chen, F. P. Gabbaï, Coordination of a stibine oxide to a Lewis acidic stiborane at the upper rim of the biphenylene backbone, *Dalton Trans.* **2018**, *47*, 12075-12078.
106. J. E. Davies, M. J. Mays, P. R. Raithby, G. P. Shields, P. K. Tompkin, A. D. Woods, The preparation of $[M_2Cp_2(CO)_4(\mu\text{-}\eta^2\text{-PE})]$ (E = As or Sb), the first complexes with naked heterodiatomic Group 15 ligands, and of their adducts with $W(CO)_5$, *Dalton Trans.* **2000**, *29*, 1925-1930.
107. J. L. Beckmann, J. Krieft, Y. V. Vishnevskiy, B. Neumann, H.-G. Stammler, N. W. Mitzel, Poly-pnictogen bonding: trapping halide ions by a tetradentate antimony(iii) Lewis acid, *Chem. Sci.* **2023**, *14*, 13551-13559.
108. A. J. Arduengo, III, C. A. Stewart, F. Davidson, D. A. Dixon, J. Y. Becker, S. A. Culley, M. B. Mizen, The synthesis, structure, and chemistry of 10-Pn-3 systems: tricoordinate hypervalent pnicogen compounds, *J. Am. Chem. Soc.* **1987**, *109*, 627-647.
109. M. S. Collins, R. Y. Choi, L. N. Zakharov, L. A. Watson, B. P. Hay, D. W. Johnson, Self-assembled trinuclear arsenic and antimony macrobicycles, *Chem. Sci.* **2015**, *6*, 2444-2448.
110. M. Jura, W. Levason, G. Reid, M. Webster, Preparation and properties of sterically demanding and chiral distibine ligands. *Dalton Trans.* **2008**, *37*, 5774-5782.
111. C. Ritter, J. Bergunde, F. Weigend, C. von Hänisch, Structure and Reactivity of Alkaline Metal Bis(amido)diazadiarsetidinides. *Z. Anorg. Allg. Chem.* **2021**, *647*, 1830-1839.
112. A. P. Bozopoulos, S. C. Kokkou, P. J. Rentzeperis, P. Karagiannidis, Structure of tris(2-pyridinethiolato)antimony(III) (TPTA), $[Sb(C_5H_4NS)_3]$. *Acta Cryst.* **1984**, *C40*, 944-946.
113. M.-A. Muñoz-Hernández, R. Cea-Olivares, R.-A. Toscano, S. Hernández-Ortega, Conformational Trends and Intermolecular Associations in Dialkyldithiophosphinate stibocanes $X(CH_2CH_2S)_2SbS_2PR_2$ (X = O, S; R = Me, Et). *Z. Anorg. Allg. Chem.* **1997**, *623*, 642-648.
114. N. C. Norman, P. M. Webster, L. J. Farrugia, Further synthetic and structural studies on cobalt carbonyl containing antimony complexes. *J. Organomet. Chem.* **1992**, *430*, 205-219.
115. L. Zimmermann, M. A. Land, C. Riesinger, C. L. B. Macdonald, M. Scheer, Synthesis of heteropnictogen ligands via P^I transfer. *Chem. Commun.* **2025**, *61*, 6973-6976.

116. C. J. Warren, S. S. Dhingra, D. M. Ho, R. C. Haushalter, A. B. Bocarsly, Electrochemical Synthesis of New Sb-Te Zintl Anions by Cathodic Dissolution of Sb_2Te_3 Electrodes: Structures of $Sb_2Te_5^{4-}$ and $Sb_6Te_9^{4-}$, *Inorg. Chem.* **1994**, *33*, 2709-2710.
